# Ancient Cytokine Interleukin 15-Like (IL-15L) Induces a Type 2 Immune Response

**DOI:** 10.3389/fimmu.2020.549319

**Published:** 2020-10-29

**Authors:** Takuya Yamaguchi, Chia Jung Chang, Axel Karger, Markus Keller, Florian Pfaff, Eakapol Wangkahart, Tiehui Wang, Christopher J. Secombes, Azusa Kimoto, Mitsuru Furihata, Keiichiro Hashimoto, Uwe Fischer, Johannes M. Dijkstra

**Affiliations:** ^1^Institute of Infectology, Friedrich-Loeffler-Institut, Greifswald-Insel Riems, Germany; ^2^Institute of Molecular Virology and Cell Biology, Friedrich-Loeffler-Institut, Greifswald-Insel Riems, Germany; ^3^Institute of Novel and Emerging Infectious Diseases, Friedrich-Loeffler-Institut, Greifswald-Insel Riems, Germany; ^4^Institute of Diagnostic Virology, Friedrich-Loeffler-Institut, Greifswald-Insel Riems, Germany; ^5^Scottish Fish Immunology Research Centre, School of Biological Sciences, University of Aberdeen, Aberdeen, United Kingdom; ^6^Institute for Comprehensive Medical Science, Fujita Health University, Toyoake, Japan; ^7^Nagano Prefectural Fisheries Experimental Station, Nagano, Japan

**Keywords:** evolution, IL-15-like, IL-15, IL-2, IL-15Rα, type 2 immunity, fish

## Abstract

Related interleukin-2, -15, and -15-like (IL-2, -15, and -15L) are ancient cytokines, with all three genes surviving in extant fish and some mammals. The present study is the first to identify IL-15L functions, namely in rainbow trout. In isolated trout splenocytes, and *in vivo*, purified recombinant IL-15L+IL-15Rα molecules induced expression of *IL-4* and *IL-13* homologs, which are markers of type 2 immunity. In contrast, trout IL-15 stimulated type 1 immunity markers, thus IL-15 and IL-15L can have opposing functions. Trout IL-15L was more dependent on “in *trans*” presentation by the receptor chain IL-15Rα than IL-15, and stimulated CD4^−^CD8^−^(IgM^−^) lymphocytes from thymus and spleen. We propose an important role for IL-15L early in the type 2 immunity cytokine cascade. Trout IL-2 and IL-15 exhibited features reminiscent of their mechanistic and functional dichotomy observed in mammals; for example, IL-15 but not IL-2 required a receptor alpha chain (only IL-15Rα in the case of fish) for its stability, and only IL-15 was efficient in stimulating lymphocytes from mucosal tissues. Data suggest that IL-15L and IL-15 may be particularly effective in stimulating innate lymphocyte type 2 cells (ILC2) and natural killer (NK) cells, respectively, but further identification of the cell types is needed. An interesting finding different from in mammals was the efficient stimulation of CD4^+^CD8^+^ thymocytes by IL-2. In short, this study presents fundamental information on the evolution of the IL-2/15/15L cytokine family.

## Introduction

Interleukin 2 (IL-2) was one of the first cytokines to be characterized. This was due to the remarkable power of IL-2 to induce and sustain T lymphocyte proliferation *in vitro*, and IL-2 was originally named “T cell growth factor” (TCGF) (1–3). Many years later, IL-15, a molecule closely related to IL-2, was discovered (4), and it took even longer to realize that IL-15 was especially potent/stable in combination with its “heterodimer partner” IL-15Rα (5–7). Nowadays, recombinant IL-2 and IL-15 (with or without IL-15Rα), or antibodies blocking their action, provide important tools for *in vitro* culturing of lymphocytes and for treating disease in the clinic or in preclinical models [reviewed in (8)]. How mammalian IL-2 vs. IL-15 functions and mechanisms are organized is only partially understood, and analysis of this cytokine family in non-mammalian species may provide additional insights.

*IL-15-like* (*IL-15L*) gene was originally discovered in teleost fish (9–11), but later the gene was also discovered in cartilaginous fish (12), reptiles, non-eutherian mammals, and some eutherian mammals including cattle, horse, pig, cat, mouse lemur, rabbit, and hedgehog (13). In rodents and higher primates only an *IL-15L* pseudogene was found, and IL-15L function is not expected in those species (13).

The cytokines IL-2, IL-15, and IL-15L are close relatives within a larger subfamily of cytokines that also includes IL-4, IL-7, IL-9, IL-13, IL-21, and thymic stromal lymphopoietin (TSLP), most of which bind receptors that contain an IL-2Rγ chain (aka “common cytokine-receptor γ-chain” or “γ_c_”) (13–15).

The following describes the IL-2 and IL-15 functions as discovered for mammals. IL-2 and IL-15 signal through the heterodimer type I receptor IL-2Rβ·IL-2Rγ and can induce very similar transcription profiles (16). Both IL-2 and IL-15 activate the transcription factor STAT5 (15, 17). Whereas, free IL-2 and IL-15 molecules can bind with low efficiency to IL-2Rβ·IL-2Rγ heterodimers, the cytokine-specific and efficient receptor complexes are formed by the heterotrimers IL-2Rα·IL-2Rβ·IL-2Rγ and IL-15Rα·IL-2Rβ·IL-2Rγ, respectively (16, 18–21). The IL-2Rα and IL-15Rα chains do not belong to the type I receptor chain family, but important parts of their ectodomains belong to the complement control protein (CCP) domain family (aka “sushi” or “SRC” domains). IL-2 is secreted predominantly by activated T cells, while IL-2Rα is constitutively highly expressed on the surface of regulatory T cells (T_regs_) and is enhanced on several leukocyte populations after their activation, most notably on effector T cells (22–24). IL-2 interacts primarily in free, secreted form with membrane-bound IL-2Rα·IL-2Rβ·IL-2Rγ complexes, and in this situation the IL-2Rα chain is said to be provided “in *cis*.” IL-2 secretion by activated T cells forms part of a self-stimulatory loop for these cells, but also provides a negative feedback loop through the stimulation of T_regs_ (25). In contrast to IL-2, the IL-15 protein is predominantly expressed together with IL-15Rα by antigen presenting cells such as monocytes and dendritic cells (23). Membrane-bound or shed/secreted IL-15·IL-15Rα complexes can stimulate other cells that express IL-2Rβ·IL-2Rγ, and in this situation the IL-15Rα chain is said to be provided “in *trans*” (26–28). The IL-15 to IL-15Rα binding mode is characterized by unusually high affinity in the picomolar range (5, 20), and, although in experiments IL-15 was shown to be able to function as an independent secreted cytokine, it was calculated that in human serum all IL-15 may be bound to soluble forms of IL-15Rα (28). Both IL-2 and IL-15 can stimulate a variety of lymphocytes, but whereas a dominant effect of IL-2 concerns the above mentioned T_reg_ stimulation (29, 30), IL-15 is particularly important for stimulation of natural killer (NK) cells, intra-epithelial lymphocytes (IELs), and CD8^+^ T cells (7, 31, 32).

Mammalian *IL-2R*α and *IL-15R*α genes were derived from a gene duplication event (21), probably early in tetrapod species evolution from an *IL-15R*α type gene, after which the IL-2Rα to IL-2 binding mode substantially diverged (13). In contrast, sequence comparisons suggest that the binding mode of IL-15 and IL-15L to IL-15Rα, as elucidated for mammalian IL-15 (33, 34), did not change during evolution of jawed vertebrates (13). In teleost (modern bony) fish, consistent with sequence motif conservation (13), and in the absence of an IL-2Rα molecule (13, 35), both IL-2 and IL-15 were found to bind with IL-15Rα, although IL-15 with a higher affinity (35).

In teleost fish, the *IL-2* and *IL-15* loci are well-conserved (13), and some studies have been done on the recombinant cytokines [reviewed in (36)]. Importantly, reminiscent of the proliferation functions in mammals, rainbow trout IL-2 and IL-15 in the supernatants of transfected cells were both able to sustain long term culturing of lymphocytes from trout head kidney (a fish lymphoid organ) that expressed markers of CD4^+^ T cells (37, 38).

Hitherto, the only functional property determined for IL-15L was its interaction with IL-15Rα, which we showed using recombinant bovine proteins (13). In contrast to the situation in mammals, bona fide *IL-15L* genes are well-conserved throughout fishes (13), so we speculated that fish IL-15L might have a more robust and easier to identify function. In the present study, we started with analyses of both rainbow trout and cattle, after which we concentrated on the rainbow trout model because only for that species we were able to detect IL-15L function. Functions of the recombinant trout cytokines were investigated using both supernatants of transfected mammalian cells and isolated proteins after expression in insect cells. Comparisons between rainbow trout IL-2, IL-15, and IL-15L functions, and their different dependencies on IL-15Rα, revealed ancient similarities of this cytokine system with the mammalian situation. Unexpected were the very different, and even opposing, immune effects that rainbow trout IL-15 and IL-15L could have on some lymphocyte populations.

## Results

### Identification, Expression Analysis, and Sequence Comparisons of Rainbow Trout IL-15La and -b

**Figure 1:**
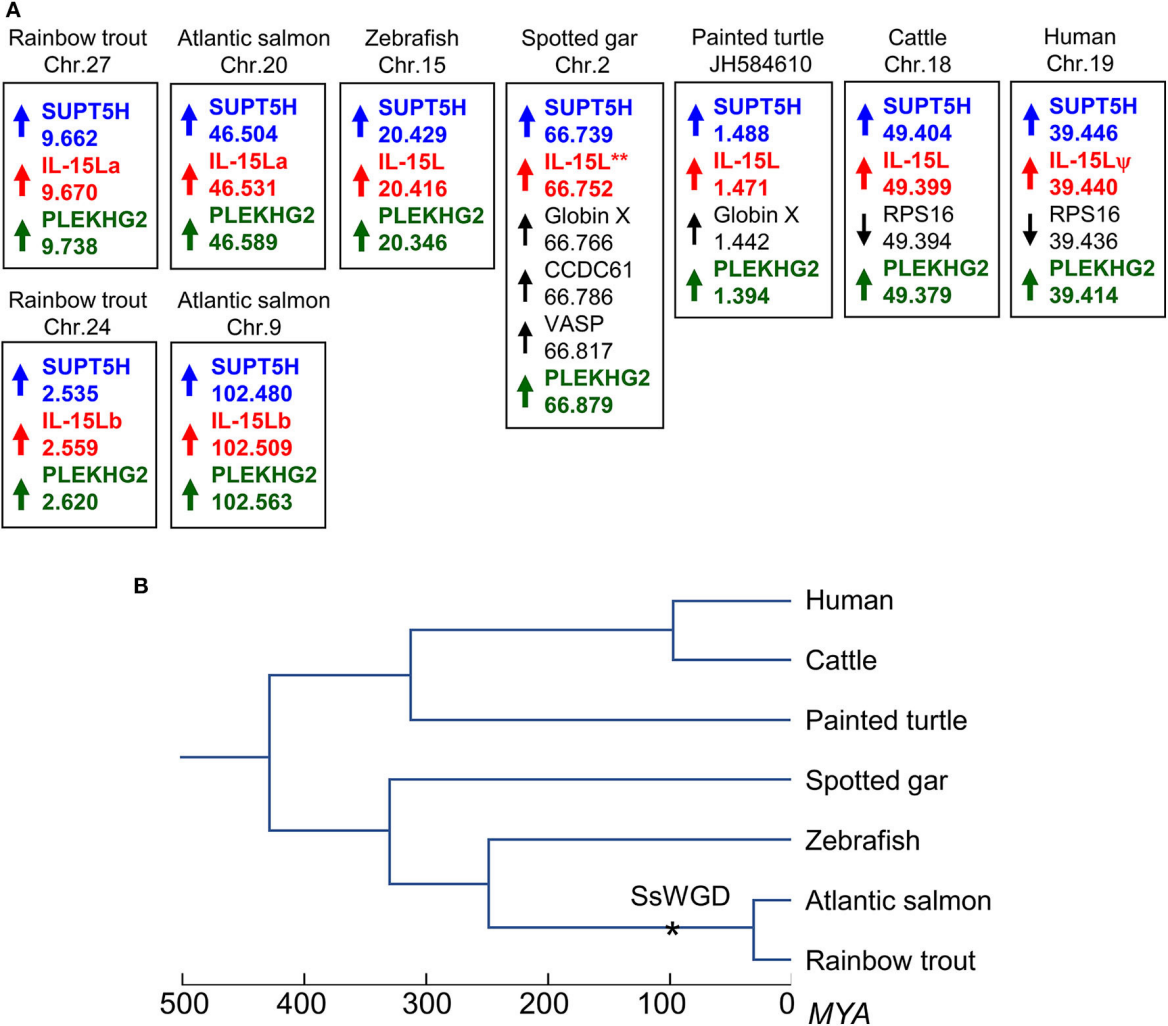
**(A)** The *IL-15L* gene is located in similar genomic regions in different species. Arrows indicate gene orientations, numbers indicate the nucleotide positions in megabase, and shared orthologous genes are highlighted by identically colored non-black font. Orientation between scaffolds was adapted to match gene orientations. **(B)** Phylogeny of the species shown in **(A)** (39); SsWGD, salmonid-specific whole genome duplication (40). (Details in **A**) In rainbow trout, Atlantic salmon (*Salmo salar*), zebrafish (*Danio rerio*), spotted gar (*Lepisosteus oculatus*), painted turtle (*Chrysemys picta*), cattle, and human, *IL-15L* loci are found close to suppressor of *Ty 5 homolog* (*SUPT5H*) and pleckstrin homology domain containing family G member 2 (PLEKHG2) genes. Human *IL-15L* is a pseudogene (IL-15Lψ) (13). Rainbow trout *IL-15La* and *IL-15Lb* were found in the rainbow trout reference genome sequence (NCBI database Omyk_1.0) chromosomes 27 and 24, respectively. Atlantic salmon *IL-15La* and *IL-15Lb* were found in the Atlantic salmon reference genome sequence (NCBI database ICSASG_v2) chromosomes 20 and 9, respectively. *For spotted gar, full-length *IL-15L* consensus gene sequence could not be found in the determined genomic sequence (13), but full-length cDNA information is available in the NCBI transcript shotgun assembly (TSA) database (see Figure 3A). Depicted zebrafish, spotted gar, turtle, cattle, and human data are based on (Pre-) Ensembl datasets GRC.z10, LepOcu1, ChrPicBel3.0.1, UMB 3.1 and GRCh38.p12, respectively. Indicated positions refer to probable ORF start codons (for all genes in trout, salmon, turtle, and human, the IL-15L genes in the other species, and PLEKHG2 in spotted gar) or the gene 5′ ends according to annotations in the Ensembl database.

**Figure 2:**
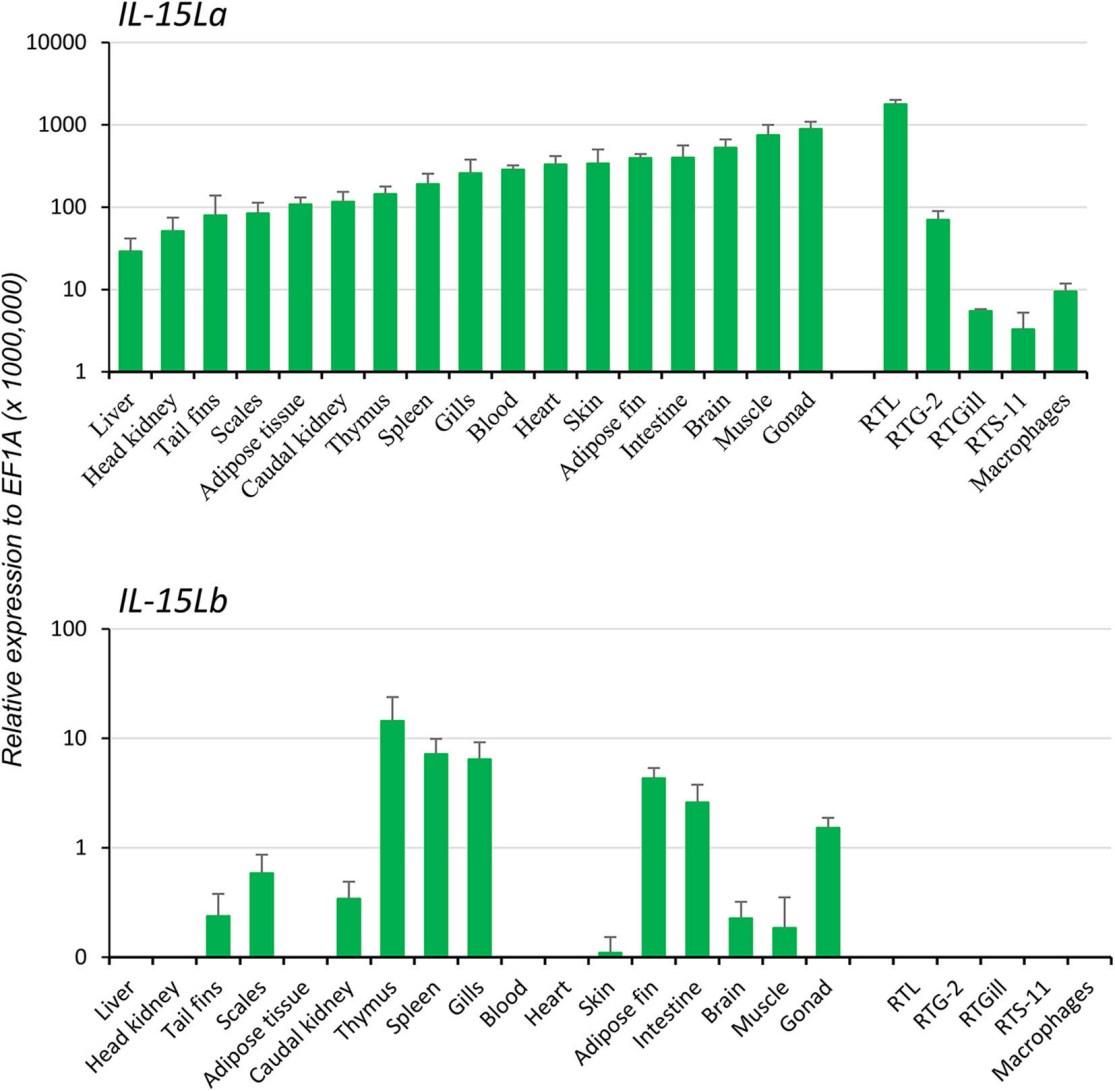
In rainbow trout, ubiquitous expression of *IL-15La* vs. a more restricted expression of *IL-15Lb* was found by RT-qPCR analysis of tissues, cell lines, and primary head kidney (HK) macrophages. Seventeen tissues from six rainbow trout individuals were sampled: blood, tail fins, scales, skin, muscle, adipose fin, thymus, gills, brain, adipose tissues, spleen, liver, heart, gonad, HK, caudal kidney, and intestine. The relative expression of each *IL-15L* gene was normalized against the expression level of the housekeeping gene *EF1A*. The expression levels in four trout cell lines—a monocyte/macrophage-like cell line RTS-11 from spleen, an epithelial cell line RTL from liver, a fibroblastic cell line RTG-2 from gonad and an epithelial cell line RTGill from gills—and in primary HK macrophages were determined in a similar way. The figure shows the means+SEM values, with *n* = 6 for the trout tissues and *n* = 4 for the cell lines and primary macrophage cultures. A table with the values per individual sample is shown in Supplementary File 1C(a).

Two rainbow trout *IL-15L* genes, *IL-15La*, and *IL-15Lb*, could be identified in genomic sequence databases (Figure 1) and were amplified from cDNA (Supplementary Files 1A,B). They map to the rainbow trout reference genome chromosomes 27 and 24, which have been recognized as a pair of chromosomes sharing ohnologous regions derived from a whole genome duplication early in the evolution of salmonid fishes (40). By 5′-RACE analysis and database comparisons a number of AUG triplets in 5′ untranslated regions (5′UTRs) of both trout *IL-15La* and *IL-15Lb* were found (Supplementary Files 1A,B, and Table 1), as reported for *IL-15L* of other fish species (9, 11) (Table 1), for mammalian *IL-15L* (13) (Table 1) and for fish and mammalian *IL-15* (9, 10, 41–43). These additional *AUG* triplets suggest that efficient translation may need some special conditions and that the transcript amounts may not be directly representative of the protein amounts (41, 42). *IL-15La* was found constitutively expressed in many tissues of healthy trout, whereas *IL-15Lb* showed a more restricted expression pattern (Figure 2 and Supplementary File 1C). Figure 2 [plus Supplementary File 1C(a)] and Supplementary File 1C(b) show our experimental RT-qPCR and semi-quantitative RT-PCR data, respectively, while Supplementary File 1C(c) shows the relative numbers of matches in tissue-specific single read archive (SRA) datasets of the NCBI database. Despite variation between trout individuals, rather consistent findings were that trout *IL-15Lb* expression was relatively high in gill, and both trout *IL-15La* and *IL-15Lb* expression were relatively low in head kidney (Figure 2 and Supplementary File 1C). In genomic sequence databases of a related salmonid fish, Atlantic salmon (*Salmo salar*), *IL-15La* and *IL-15Lb* could also be found (Figure 1), and comparison of these sequences with tissue-specific RNA-based SRA datasets indicated that *IL-15La* and *IL-15Lb* expression in Atlantic salmon agree with the above summary for trout [Supplementary File 1C(c)]. Figure 2 shows that *IL-15La* transcripts were also found in trout macrophages, and epithelial and fibroblast cell lines.

**Table 1:** Number of AUG codons in the 5′UTR of reported *IL-15L*transcript sequences.

**Species**		**Length of 5'UTR (in nt)**	**Number of AUGs in 5'UTR**		
**clade**	**Species**			**NCBI accession**	**Article**
Teleost fish	(*IL-15La*) Rainbow trout (*Oncorhynchus mykiss*)	820	15	(TSA) GBTD01057175	
	Idem	59	2	(TSA) GDKP01008169	
	Idem	635	12	MK619679	The current study
	(*IL-15La*) Brown trout (*Salmo trutta*)	639	9	(TSA) GFIS01054003	
	(*IL-15La*) Atlantic salmon (*Salmo salar*)	662	15	(TSA) GBRB01011922	
	Idem	634	9	(EST) DW573175*	
	Idem	650	9	(EST) EG765561*	
	(*IL-15Lb*) Rainbow trout (*Oncorhynchus mykiss*)	315	7	MK619680	The current study
	(*IL-15Lb*) Lake whitefish (*Coregonus clupeaformis*)	433	9	(TSA) GFGX01037271	
	(*IL-15Lb*) Grayling (*Thymallus thymallus*)	1109	17	(TSA) GFIZ01047949	
	Northern snakehead (*Channa argus*)	722	12	(TSA) GEGU01032800	
	Idem	800	12	(TSA) GEML01050890	
	Idem	1000	14	(TSA) GEGU01032799	
	Idem	1078	14	(TSA) GEML01050892	
	Tetraodon (*Tetraodon nigrovirides*)	250	5	AY374511	(10)
	Largemouth bass (*Micropterus salmoides*)	572	13	(TSA) GBFO01032257	
	Barramundi perch (*Lates calcarifer*)	181	5	(TSA) GAQL01246981	
	Idem	721	12	(TSA) GAQL01163692	
	Idem	363	7	(EST) EX468503*	
	Channel catfish (*Ictalurus punctatus*)	214	2	(TSA) JT467338	
	Idem	401	7	(TSA) GELA01014758	
	Dojo (*Misgurnus anguillicaudatus*)	178	3	(EST) BJ837638	
	Idem	354	10	(EST) BJ826350*	
	Zebrafish (*Danio rerio*)	440	13	(TSA) GFIL01006389	
	Idem	136	3	(EST) CV484401	
	Idem	250	8	(EST) EH568567	
	Idem	540	16	(EST) EH547439*	
	Fathead minnow (*Pimephales promelas*)	245	10	(TSA) GCVQ01032974	
Reptiles	Green anole (*Anolis carolinensis*)	524	9	(TSA) GBDW01152911	
	Idem	993	14	(TSA) GADO01152734	
	Idem	114	1	(TSA) GBDE01207268	
Marsupials	Long-nosed bandicoot (*Perameles nasuta*)	314	3	(TSA) GFSS01117967	
Eutherian mammals	Cattle (*Bos taurus*)	225	3	**Supplementary File 3** in (13)	(13)
	Idem	571	6	**Supplementary File 3** in (13)	(13)
	Idem	647	6	JX271582	(13)
	Rabbit (*Oryctolagus cuniculus*)	347	3	(TSA) GBCT01158384	
	Idem	91	0	**Supplementary File 3** in (13)	(13)
	Idem	375	1	JX271583	(13)

For teleost fish all IL-15L sequences present in the NCBI expressed sequence tag (EST) database that (potentially*) encode canonical IL-15L protein are listed, whereas for teleost fish IL-15L sequences in the NCBI TSA sequence database only representative sequences are listed.

*In-frame AUGs at non-consensus positions were considered as start codons if favorable for encoding signal peptides, and included in the 5'UTR AUG number if unfavorable*.

*Because of low expression levels, for intact mammalian and reptilian IL-15L transcripts little information is available (13)*.

* *Some of the listed sequences end within the IL-15L open reading frame and additional analysis should clarify whether they are derived from RNAs encoding full-length canonical IL-15L protein*.

**Figure 3:**
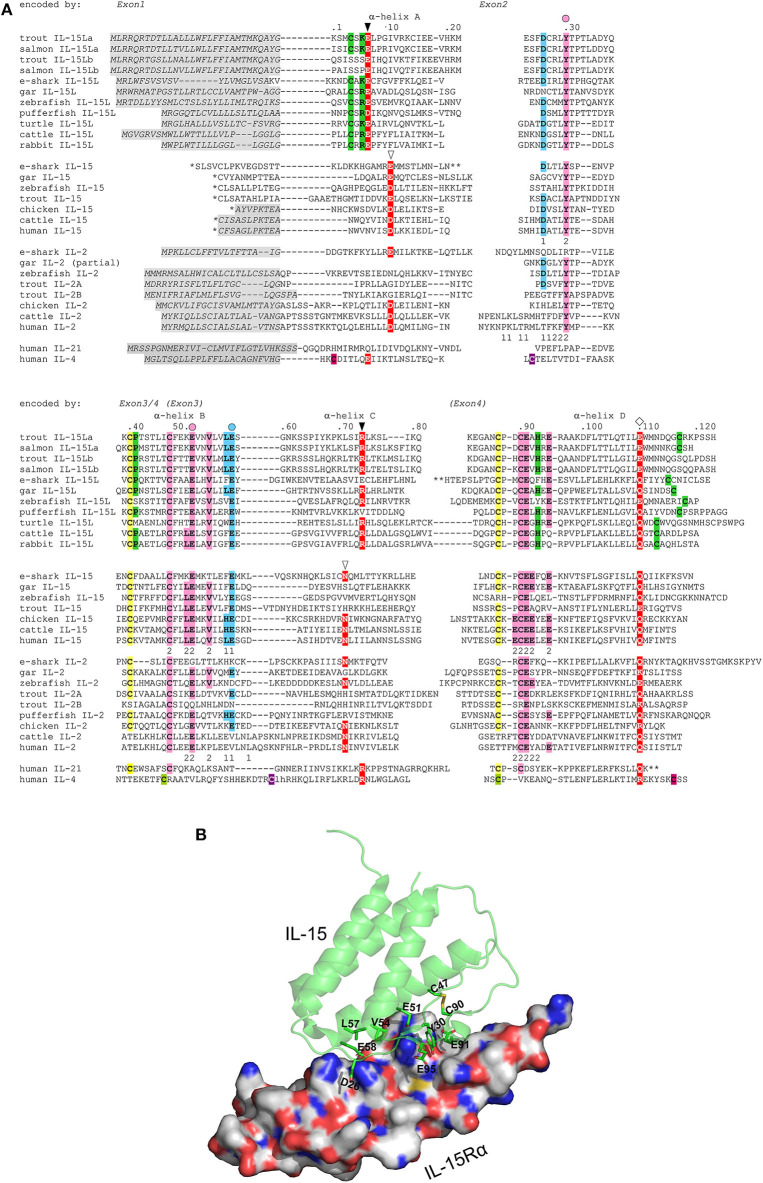
**(A)** Rainbow trout IL-15La and IL-15Lb possess sequence motifs characteristic for IL-15L and for the IL-2/15/15L-family, but trout IL-15Lb lost some IL-15L-concensus residues. Identical colored shading of cysteines refers to known or expected disulfide bridges. Residues rather characteristic for IL-15L (13), including a cysteine pair, are shaded green. In the interaction of mammalian IL-15 and IL-2 with their respective receptor chains IL-15Rα or IL-2Rα, two binding patches “1” and “2” were distinguished, with patch 2 quite similar and patch 1 quite different between IL-15·IL-15Rα and IL-2·IL-2Rα (33, 34); the participating residues in human IL-15 and IL-2 are indicated with the respective number 1 or 2 below them. To highlight the conservation of the IL-15 to IL-15Rα binding mode, residues in the alignment that are identical to the human or murine IL-15 patch 1 residues are shaded blue, and residues that are identical to the human or murine IL-15 patch 2 are shaded pink; the most important residues for IL-15 to IL-15Rα binding (34) are indicated by a colored circle above the alignment. Residues which are known or are expected to be of importance for interaction with type I receptors are shaded red (13, 16, 44–48); the open triangles indicate residues important for interaction of mammalian IL-15 and IL-2 with the IL-2Rβ chain, the closed triangles indicate positions which in IL-4 are important for interaction with IL-4Rα chain, and the diamonds are indicated above a glutamine or arginine which in mammalian IL-2, IL-15, or IL-4 is important for binding IL-2Rγ chain (16, 46–48). *, for IL-15 sequences the leader peptide amino acid sequences encoded by exons upstream from family consensus exon 1 are not shown. **, for elephant shark IL-15L, elephant shark IL-15 and human IL-21 a stretch is not shown for lay-out reasons. Residue numbering follows trout IL-15La. Italic font and gray shading, (predicted) leader peptides. Gaps, open spaces relate to exon borders whereas hyphens connect residues encoded by the same exon. For comparisons with additional cytokines see reference (13). Names of species are: trout, rainbow trout (*Oncorhynchus mykiss*); salmon, Atlantic salmon (*Salmo salar*); e-shark, elephant shark (*Callorhinchus milii*); gar, spotted gar (*Lepisosteus oculatus*); zebrafish (*Danio rerio*); pufferfish, green spotted pufferfish (*Tetraodon nigroviridis*); turtle, painted turtle (*Chrysemys picta*); cattle (*Bos taurus*); human (*Homo sapiens*). Database accessions for the sequences are [for the sequences also see references (1, 10)]: e-shark IL-15L, GenBank KA353649; gar IL-15L, GenBank GFIM01029449; zebrafish IL-15L, GenBank NP_001009558; rainbow trout IL-15La, GenBank MK619679; rainbow trout IL-15Lb, GenBank MK619680; Atlantic salmon IL-15La, GenBank GBRB01011922; Atlantic salmon IL-15Lb, predicted from GenBank AGKD04000049; pufferfish IL-15L, predicted from Ensembl “TETRAODON8” and described by Fang et al. (10); turtle IL-15L, GenBank XP_008171403; cattle IL-15L, NP_001288142; rabbit IL-15L, NP_001288189; e-shark IL-15, JW878023; gar IL-15, Ensembl “LepOcu1”; zebrafish IL-15, GenBank AAZ43090; trout IL-15, GenBank AJ555868; chicken IL-15, GenBank AAD38392; cattle IL-15, AAA85130; human IL-15, AAA21551; e-shark IL-2 (alias IL-2-like), predicted from the elephant genome project sequence which has GenBank accession AAVX02000000; gar IL-2, predicted from Ensembl “LepOcu1”; zebrafish IL-2, predicted from Ensembl “Zv9”; trout IL-2A, GenBank NM_001164065; trout IL-2B, GenBank HE805273; chicken IL-2, GenBank AAC96064; cattle IL-2, GenBank AAA30586; human IL-2, GenBank 0904306A; human IL-21, Genbank AAG29348; human IL-4, GenBank AAA59149. For comparisons with additional cytokines see (13). **(B)** The ancient IL-2/15/15L-family patch 1 and patch 2 residues that are conserved in trout IL-15L are (expected to be) situated at the cytokine-to-IL-15Rα interface. The structure shown is of human IL-15 with human IL-15Rα ectodomain (PDB accession 2Z3Q) (33). The figure was made using PyMOL software. IL-15 is depicted in green semi-transparant cartoon format with highlighting, in sticks format, of sidechains of IL-15Rα-binding residues that are conserved in trout IL-15L. IL-15Rα is depicted in white surface format. Blue is used for N atoms, red for O, gold for S, and C atoms are in molecule-specific colors.

The deduced amino acid sequences of trout IL-15La and IL-15Lb are aligned in Figure 3A together with related cytokines. Residues that are rather typical for IL-15L (13) are shaded green. Phylogenetic tree analysis comparing these highly diverged cytokines does not provide conclusive information on their evolution (13), but when such analysis is performed on only the IL-15L sequences the result (Supplementary File 1D) is consistent with the location-based assumption (see above) that rainbow trout *IL-15La* and *IL-15Lb* are paralogues which were generated by the whole genome duplication in an ancestor of salmonids (40). As we discussed previously (13), although the conservation of the overall sequences is poor, residues of mammalian IL-15 that are known to bind IL-15Rα are well-conserved throughout IL-15, IL-15L, and fish IL-2. In Figure 3A, blue and pink shading mark residues of binding patches 1 and 2 determined for mammalian IL-15 to IL-15Rα binding (33, 34), with the most important residues (34) indicated with a circle above; this impressively conserved set of residues is shown at the structural level in a human IL-15·IL-15Rγα, complex (Figure 3B) as determined previously (33). Residues of mammalian IL-2, IL-15, and IL-4 which are known to be of major importance for interaction with their respective type I receptors (16, 44–48) are shaded red in Figure 3A, and so are residues of the other cytokines for which a similar importance may be expected (13). Although some of the alignments of the highly diverged α-helix A and C regions in Figure 3A are quite speculative [for a better discussion of the alignment see (13)], among IL-15L sequences an acidic residue (D/E) in α-helix A, an arginine in α-helix C, and a glutamine or glutamic acid (E/Q) in α-helix D that may participate in type I receptor binding are rather well-conserved.

Very recently, it was described that rainbow trout has two quite different IL-2 molecules, IL-2A and IL-2B, which have overlapping but distinct functions (49). Figure 3A shows that compared to fish IL-2 consensus the rainbow trout IL-2B molecule lost cysteines and some residues for IL-15Rα binding, a topic for future studies. In the present study, we only analyze rainbow trout IL-2A, which for simplicity we call “IL-2.” Since trout IL-15Lb lost an IL-15L consensus cysteine pair (the green shaded cysteines in Figure 3A), we speculated that trout IL-15La function would be more representative of canonical IL-15L function, and therefore most research in the present study was dedicated to this protein version.

### Trout IL-15La Can Be N-glycosylated

**Figure 4:**
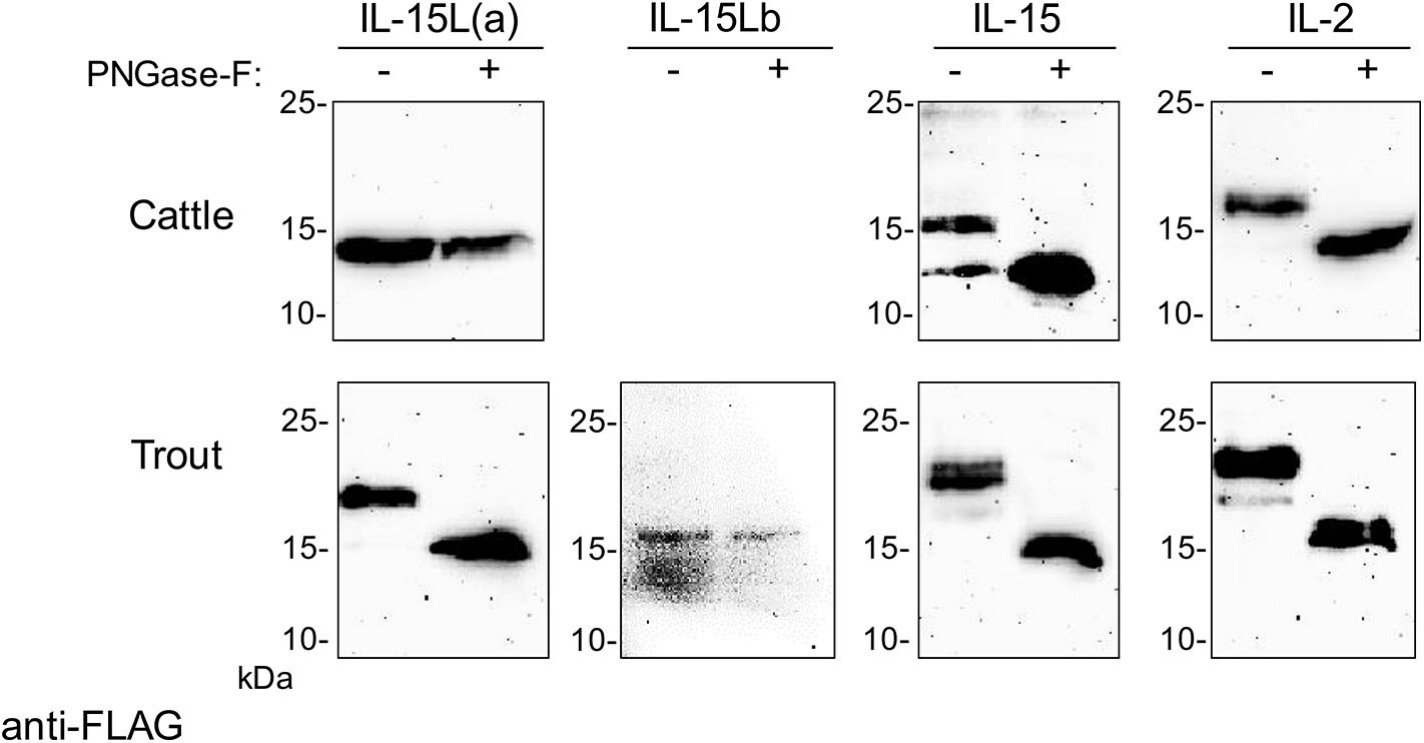
Bovine IL-2 and IL-15, and rainbow trout IL-2, IL-15, and IL-15La in transfected HEK293T cells are N-glycosylated. Lysates of HEK293T cells transfected for the indicated bovine or trout cytokine were digested with PNGase-F (+) or mock treated (–) and analyzed by Western blotting using anti-FLAG mAb. All analyzed molecules except bovine IL-15L and trout IL-15Lb showed a shift in apparent molecular weight consistent with removal of N-linked oligosaccharides. For full-size blots and additional controls see Supplementary File 5A.

Trout IL-15La has a single N-glycosylation motif [NxS/T (50)] at position 61 (Figure 3A). Human HEK293T cells were transfected with DNA plasmid expression vectors encoding FLAG-tagged versions of bovine IL-2, IL-15, and IL-15L, and trout IL-2, IL-15, IL-15La, and IL-15Lb (for sequences of expression vectors see Supplementary File 2). After 24 h, cell lysates were prepared and treated with PNGase-F or without (mock treatment), and then the samples were subjected to anti-FLAG Western blot analysis. This revealed shifts in apparent molecular weight indicative of N-glycosylation for most investigated cytokines but not for bovine IL-15L and trout IL-15Lb (Figure 4 and Supplementary File 5A). The results are consistent with these latter two cytokines not having an N-glycosylation motif (Figure 3A).

### Cross-Reactivities Between Trout and Bovine Cytokines and IL-15Rα

**Figure 5:**
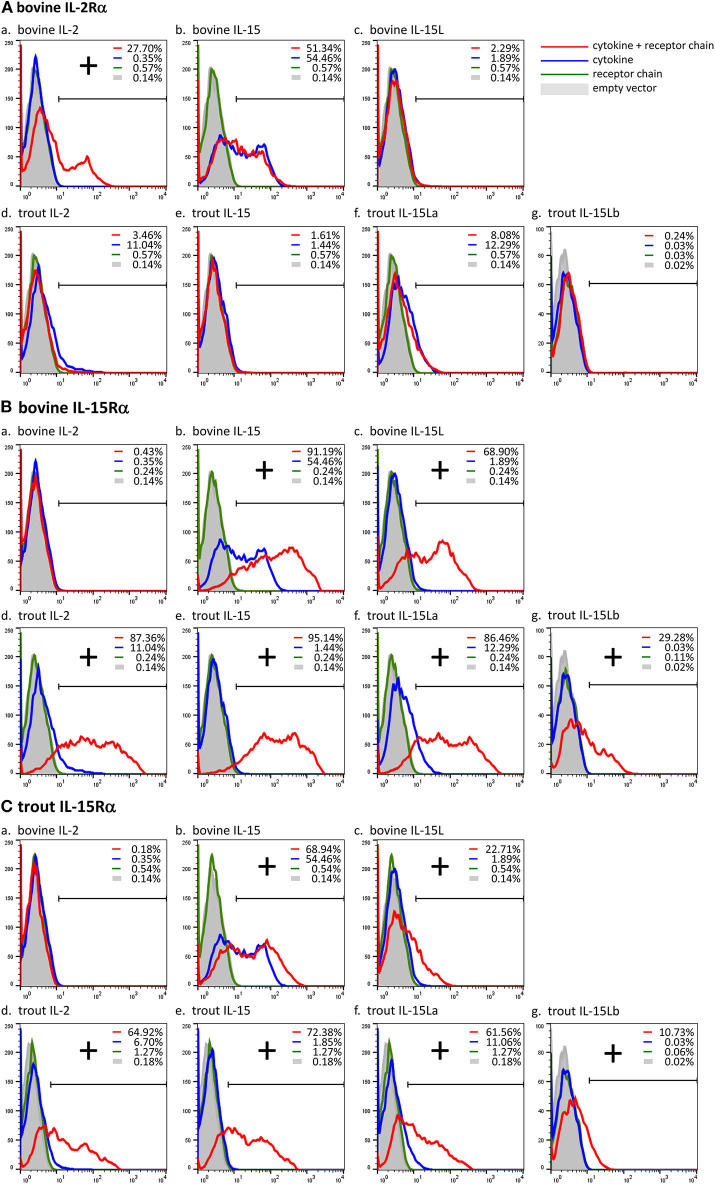
Cross-species interactions between receptor chain IL-15Rα of cattle and trout, and the cytokines IL-15 and IL-15L of both species, and IL-2 of trout, were revealed by cytokine presence at the cell surface after co-transfection of encoding plasmids. In contrast, the interaction between bovine IL-2 and bovine IL-2Rα was found to be mutually exclusive. Live transfected human HEK293T cells were analyzed by flow cytometry using anti-FLAG mAb to detect the presence of the FLAG-tagged cytokines on the cell surface. The percentages of anti-FLAG-labeled cells were compared between cells transfected for cytokine alone (blue line), cells co-transfected for cytokine plus receptor chain (red line), cells transfected for the receptor chain alone (green line) and cells transfected with empty vector (gray). Bovine IL-2Rα **(A)**, bovine IL-15Rα **(B)**, and trout IL-15Rα **(C)** were tested in combination with each cytokine (a-g). If the percentage of anti-FLAG-labeled cells was higher in the cells co-transfected for the receptor chain than in the cells transfected for the cytokine alone, the cytokine was considered to be bound to the receptor chain (shown as “+”). For a summary of experiment repeat results see Supplementary File 7.

Previously, we showed, by a combination of DNA plasmid transfection and anti-FLAG flow cytometry experiments, that FLAG-tagged bovine IL-15L could be found on the surface of HEK293T cells if they were co-transfected for bovine IL-15Rα but not if co-transfected for bovine IL-2Rα (13). In the present study we repeated this analysis, but in addition included recombinant expression of trout IL-15Rα, and of FLAG-tagged bovine IL-2 and IL-15, and trout IL-2, IL-15, IL-15La, and IL-15Lb. The results of representative experiments are shown in Figure 5, while the table in Supplementary File 7 summarizes the results of all experiment repeats that were done. The interaction between bovine IL-2Rα chain and bovine IL-2 was mutually specific (Figure 5A). Bovine IL-15 and IL-15L, and trout IL-2, IL-15, IL-15La, and IL-15Lb, could only be detected, or were detected at higher amounts, at the cell surface, if the cells were co-transfected for either bovine or trout IL-15Rα (Figures 5B,C). The cross-species interactions appeared to be especially efficient for bovine IL-15Rα co-expressed with trout IL-2, IL-15, and IL-15L (Figure 5B), but were also observed for trout IL-15Rα co-expressed with bovine IL-15 and IL-15L (Figure 5C). For unknown reasons, recombinant bovine IL-15 (in which the leader sequence had been replaced for that of IL-2; see Supplementary File 2) was also detectable at the cell surface in the absence of co-transfected receptor chains (Figures 5Ab,Bb,Cb).

### Dependency on Soluble IL-15Rα for Efficient Stable Secretion of Bovine and Trout IL-15 and IL-15L by Transfected HEK293T Cells

**Figure 6:**
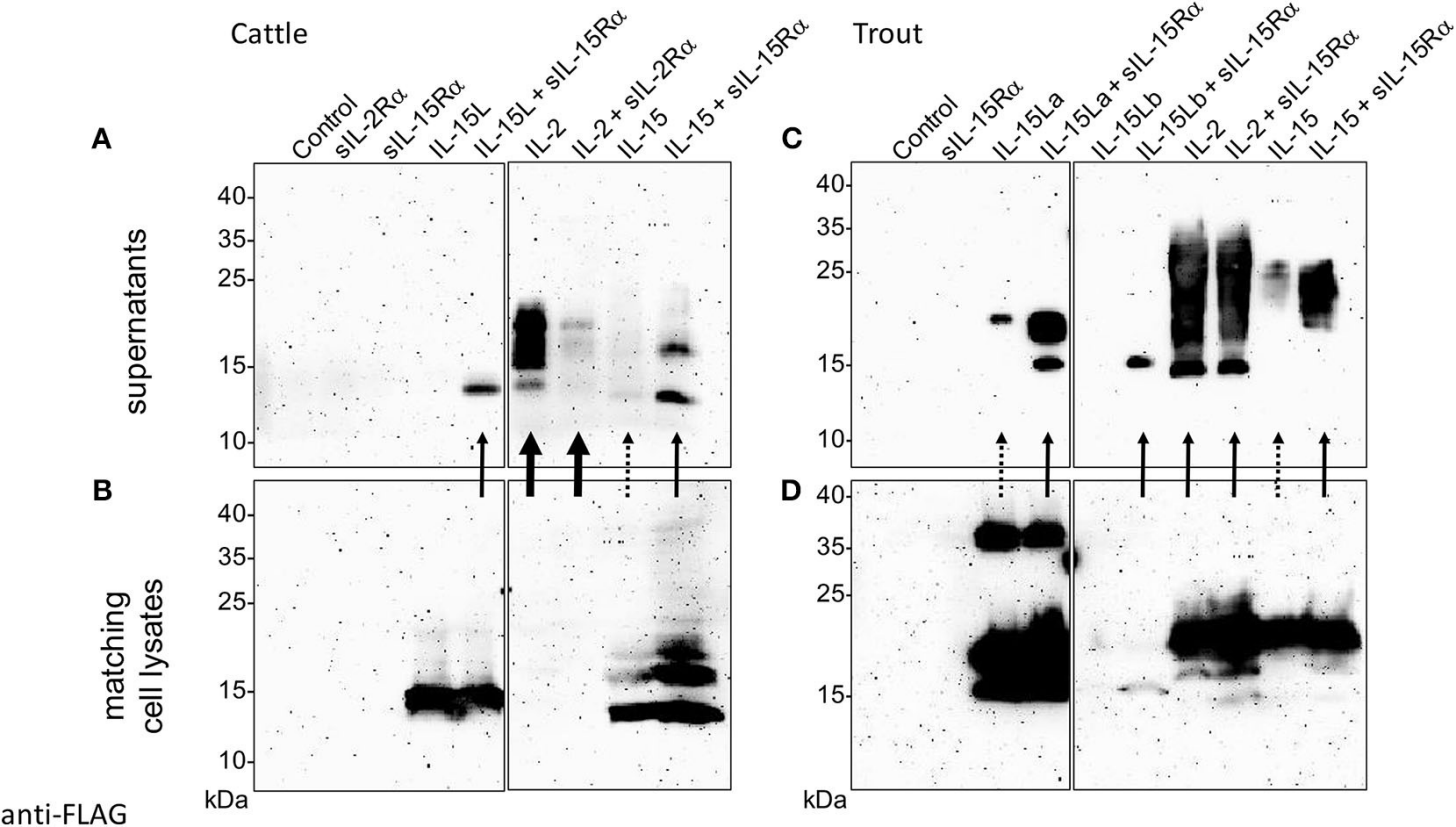
Co-expression with soluble IL-15Rα enhances presence of bovine and trout IL-15 and IL-15L in the supernatant of transfected cells. HEK293T cells were transfected for FLAG-tagged cytokines and/or species-specific Myc-tagged soluble IL-2Rα or IL-15Rα, and the matching supernatants (**A**, cattle; **C**, trout) and cell lysates (**B**, cattle; **D**, trout) were compared by Western blot analysis using anti-FLAG mAb. Thick, thin and dashed arrows highlight very efficient secretion, intermediate efficient secretion, and poor secretion, respectively, as they are deduced from comparison between the supernatant and cell lysate results. For experiment repeats and additional information see Supplementary File 5B.

Previously, we found that recombinant bovine IL-15L could only be found in the supernatant of transfected cells if co-transfected for soluble IL-15Rα (sIL-15Rα) (13). In the present study that research was extended by also investigating the effect of co-transfection for species-specific sIL-15Rα on the stable secretion of bovine IL-15 and of trout IL-2, IL-15, and IL-15L, and that of co-transfection for bovine sIL-2Rα on bovine IL-2. Figures 6A–D, show the Western blot results of representative experiments in which the bovine and trout molecules were expressed, respectively, comparing the cytokines present in the supernatant (Figures 6A,C) to those present in the matching cell lysates (Figures 6B,D). Supplementary File 5B shows experiment repeats and the uncropped blot results, and in addition includes the Western blot analyses for detection of the receptor chains. The data in Figure 6 and Supplementary File 5B consistently indicate that bovine and trout IL-15 and IL-15L are dependent for their abundance in the supernatant on the co-expression with, or fusion to sIL-15Rα (Supplementary File 5B). As reported before (13), no IL-15L was detectable in supernatants of cells transfected for bovine IL-15L alone (Figure 6A). When transfected for only trout IL-15La, small amounts of the cytokines could be detected in the supernatant, but these increased markedly upon co-transfection for, or genetic fusion to, sIL-15Rα (Figure 6C and Supplementary File 5B). Trout IL-15Lb was consistently found in lower amounts than the other cytokines, even in the transfected cell lysates, especially in the absence of sIL-15Rα (Figure 6D), which seems to have a stabilizing role and to be necessary for finding any trout IL-15Lb in the cell supernatant (Figure 6C). Similar to IL-15L, the presence of bovine and trout IL-15 in the supernatant was considerably boosted by the co-transfection for sIL-15Rα (Figures 6A,C). Stable secretion of IL-2 of cattle and trout did not depend on receptor chain co-expression (Figures 6A,C), and especially bovine IL-2 was efficiently released from the cells (compare Figure 6A with Figure 6B). In Figure 6, although somewhat arbitrarily, very efficient secretion, intermediate efficient secretion, and poor secretion are highlighted with arrows, estimated from comparison of Figure 6A with Figure 6B, and of Figure 6C with Figure 6D. Whether the increased amounts of IL-15 and IL-15L in the supernatants in the presence of sIL-15Rα were caused by enhanced secretion, improved stability, or by both, needs further investigation. We interpret the band of ~37 kDa observed for the cell lysate samples containing trout IL-15La as a possible IL-15La homodimer (Figure 6D); similar sized trout IL-15La protein complexes can also be seen in additional Western blot figures in Supplementary Files 5A,B, and were also observed for purified IL-2 (see below).

### Trout IL-2, IL-15, and IL-15L in Supernatants of Transfected HEK293T Cells Induce STAT5 Phosphorylation in Distinct Lymphocyte Populations; Trout IL-15La and IL-15Lb Stimulate CD4^−^CD8^−^ (Double Negative, DN) Thymocytes

**Figure 7:**
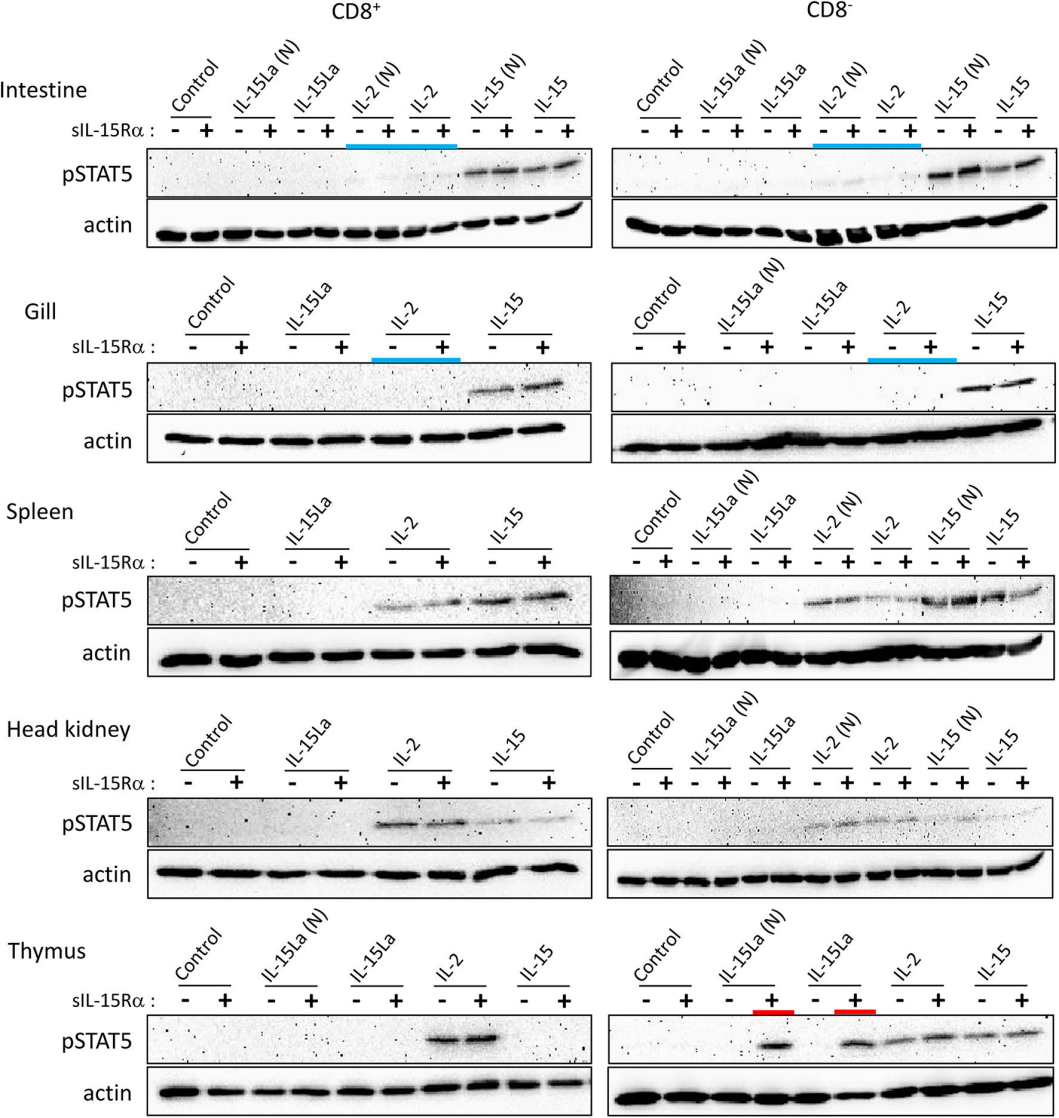
Phosphorylation of STAT5 in CD8^+^ and CD8^−^ fractions of trout lymphocytes isolated from several tissues, induced after incubation for 15 min with recombinant trout cytokine containing HEK293T cell supernatants. Western blot analysis of cell lysates, using anti-pSTAT5 mAb. Symbols “–” and “+” indicate whether cytokines were co-expressed with trout sIL-15Rα. “Control” refers to incubation with supernatants of cells transfected with empty vector in the place of a cytokine encoding vector. Highlighted with colors are the ability of IL-15La plus sIL-15Rα to stimulate DN thymocytes (red) and the (relative) inefficiency of IL-2 to stimulate lymphocytes from the mucosal tissues intestine and gill (blue). An addition “(N)” indicates that no tag was added to the recombinant cytokine (see Supplementary File 2). See Supplementary File 5C for experiment repeats and additional information.

Preliminary experiments in which total leukocytes of different rainbow trout tissues were stimulated with cytokine-containing supernatants of transfected cells did not reveal induction of phosphorylated STAT5 (pSTAT5) by IL-15L. Therefore, we tried to increase the sensitivity by first sorting the CD8α-positive and -negative (CD8^+^ and CD8^−^) morphological lymphocyte fractions (FSC^low^/SSC^low^ in flow cytometry; mostly called “lymphocytes” from here) using an established monoclonal antibody (51) (Supplementary File 3). These cells were incubated with supernatants of HEK293T cells transfected for trout cytokines and/or for trout sIL-15Rα, or with control supernatant, and then pSTAT5 amounts were compared by Western blot analysis. Results are shown in Figure 7 and Supplementary File 5C. In several experiments, but not consistently in all experiments, non-tagged versions of the cytokines were included [named IL-2(N), IL-15(N), IL-15La(N) and IL-15Lb(N)], to exclude the possibility that a FLAG-tag effect was responsible for the experimental outcome. Trout IL-2 efficiently stimulated both CD8^+^ and CD8^−^ lymphocytes from the systemic lymphoid tissues spleen and head kidney, and also from the thymus (Figure 7). However, IL-2 was not found to stimulate lymphocytes from gill, and only had a weak stimulatory effect on CD8^+^ and CD8^−^ populations isolated from intestine (highlighted by blue bars in Figure 7). That IL-15 was more efficient than IL-2 in the stimulation of lymphocytes from intestine and gill was evident because the induced pSTAT5 amounts were higher while the amounts of recombinant cytokine used for stimulation were smaller (Supplementary File 5C). Trout IL-15La and IL-15Lb containing supernatants did not detectably induce pSTAT5 in any of the investigated cell populations, except for CD8^−^ thymocytes (Figure 7 and Supplementary File 5C; highlighted by red bars in Figure 7; IL-15Lb data are only shown in Supplementary File 5C). The stimulation by IL-15La and IL-15Lb appeared to be fully dependent on the co-presence of sIL-15Rα (Figure 7 and Supplementary File 5C), although it should be realized that in absence of sIL-15Rα the concentrations of IL-15La and IL-15Lb in the supernatant were very low or absent (see Figure 6).

**Figure 8:**
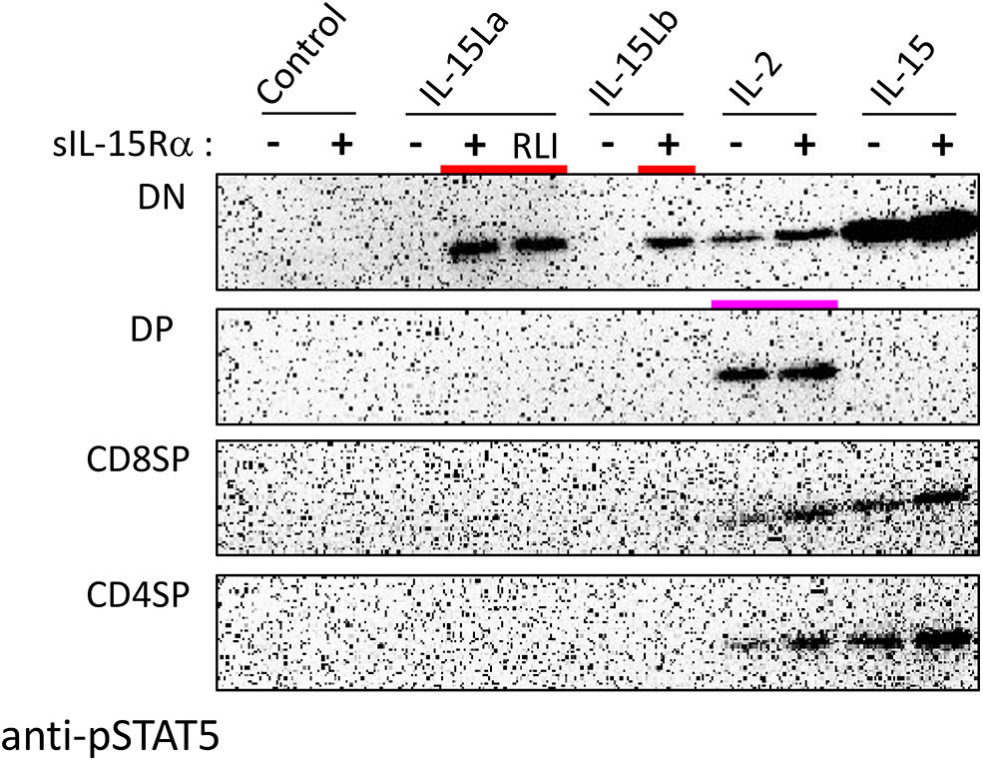
Phosphorylation of STAT5 in CD8^−^CD4^−^ (DN), CD8^+^CD4^+^ (DP), CD8^+^CD4^−^ (CD8SP), and CD8^−^CD4^+^ (CD4SP) fractions of trout thymocytes, induced after incubation for 15 min with recombinant trout cytokine containing HEK293T cell supernatants. Western blot analysis of cell lysates using anti-pSTAT5 mAb. Symbols “–” and “+” indicate whether cytokines were co-expressed with trout sIL-15Rα. “Control” refers to incubation with supernatants of cells transfected with empty vector in the place of a cytokine encoding vector. Highlighted with colors are the ability of IL-15La plus sIL-15Rα, IL-15Lb plus sIL-15Rα, and a fusion of trout IL-15La to, in this case, human sIL-15Rα (RLI), to stimulate DN thymocytes (red) and the ability of IL-2 to stimulate DP thymocytes (magenta). See Supplementary File 5D for experiment repeats and additional information.

During our studies, monoclonal antibodies against rainbow trout CD4-1 and CD4-2 became available (52); whether CD4-1 and CD4-2 have similar or different functions is not known, but in CD4-positive lymphocytes they commonly are co-expressed (52). To further investigate which thymocytes of trout were stimulated by IL-15La and IL-15Lb, thymocytes were labeled with an anti-CD8α monoclonal antibody with a different isotype (see Supplementary File 3A) than the above-mentioned (51) and additionally labeled for CD4 (using a mixture of anti-CD4-1 and anti-CD4-2; Supplementary File 3). Upon stimulation with supernatants of cells transfected for the various trout cytokines, it was found that IL-15La and IL-15Lb induced STAT5 phosphorylation in only unstained thymocytes (i.e., double negative or DN thymocytes; Figure 8 and Supplementary File 5D; highlighted with red bars in Figure 8). As observed for the CD8^−^ thymocytes (Figure 7 and Supplementary File 5C), this stimulation was dependent on co-presence of, or fusion to, sIL-15Rα (Figure 8 and Supplementary File 5D; the “RLI” protein is a fusion version). A notable observation is that cells stained for both CD4 and CD8 molecules (double positive or DP thymocytes) were only sensitive to IL-2 and not to IL-15 or to IL-15L (Figure 8 and Supplementary File 5D; highlighted with a magenta bar in Figure 8).

### Expression of Trout IL-2, IL-15, and IL-15L in Insect Cells

To enable experiments under quantitatively controlled conditions, trout FLAG-tagged IL-2, IL-15 and IL-15La, and trout Myc-tagged sIL-15Rα were expressed in insect cells using a baculovirus system. Expression of the cytokines was undertaken with or without co-expression of trout sIL-15Rα, and in the case of IL-15 and IL-15La also as genetic fusions with trout sIL-15Rα; the fusion products were named IL-15-RLI and IL-15La-RLI. Recombinant proteins were isolated from the supernatant using anti-FLAG agarose and the resulting preparations were analyzed by size exclusion chromatography and by Coomassie staining and Western blotting after SDS-PAGE. Western blot analyses revealed that the sIL-15Rα proteins could only be isolated by anti-FLAG agarose when co-expressed with FLAG-tagged IL-2, IL-15, or IL-15La (Supplementary Files 4A–C), confirming the interaction of all three cytokines with IL-15Rα as already shown with different experiments in Figure 5. Size exclusion chromatography results indicated that IL-15, IL-15La, sIL-15Rα and IL-2+sIL-15Rα preparations may be unstable and prone to aggregation (not shown), and these preparations were not used for functional assays. For functional studies of IL-2 a preparation was used which mainly behaved as an apparent homodimer during size exclusion chromatography (Supplementary File 4F) as described for mammalian IL-2 preparations (53); Western blot analysis of the purified trout IL-2 also suggested the ability to form homodimers (Supplementary File 4F). Since initial analyses indicated functional similarity between the noncovalent associations and genetically linked forms of IL-15 or IL-15La with sIL-15Rα [Supplementary Files 5F(a,b); see also Figure 8], and because of the convenience and apparent stability, the preparations of the genetic fusion products IL-15-RLI and IL-15La-RLI (Supplementary Files 4D,E) were selected over the noncovalent associations for further functional studies. When using sensitive cells, the trout IL-2, IL-15-RLI, and IL-15La-RLI proteins were found to induce pSTAT5 from concentrations of 40 pM or less (Supplementary File 5F), which is reminiscent of the working concentrations found for recombinant IL-2 and complexes of IL-15 with sIL-15Rα in human systems (16).

### High Concentrations of Trout IL-15La-RLI Induce STAT5 Phosphorylation in Trout Splenocytes

**Figure 9:**
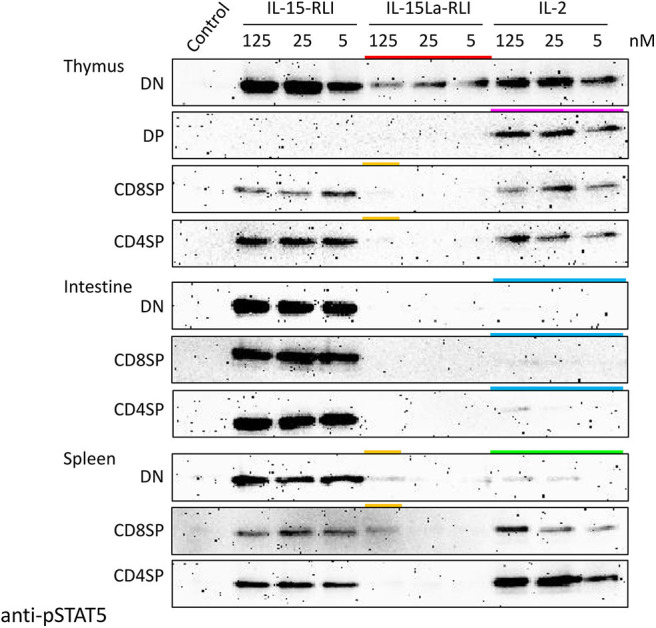
Phosphorylation of STAT5 in lymphocyte fractions of trout thymus, intestine, and spleen, induced after incubation for 15 min with purified recombinant trout cytokines (produced in insect cells) IL-2, IL-15-RLI, and IL-15La-RLI at 5, 25, and 125 nM. Western blot analysis of cell lysates, using anti-pSTAT5 mAb. DN, CD8^−^CD4^−^; DP, CD8^+^CD4^+^; CD8SP, CD8^+^CD4^−^; CD4SP, CD8^−^CD4^+^. Lysates of mock-treated cells were loaded as “Control.” Highlighted with colors are the ability of IL-15La-RLI to stimulate DN thymocytes (red), the ability of IL-2 to stimulate DP thymocytes (magenta), the (relative) inefficiencies of IL-2 to stimulate intestinal lymphocytes (blue) and DN splenocytes (green), and the weak ability of IL-15La-RLI to stimulate DN and CD8SP splenocytes and CD8SP and CD4SP thymocytes (orange). See Supplementary File 5E for additional information.

Three different concentrations (5, 25, and 125 nM) of recombinant IL-2, IL-15-RLI, and IL-15La-RLI proteins isolated from insect cells were used to stimulate CD4^+^CD8^−^ (CD4SP [single positive]), CD4^−^CD8^+^ (CD8SP), and CD4^−^CD8^−^ (DN) lymphocyte fractions of thymus, intestine, and spleen, while for the thymus this analysis also included the CD4^+^CD8^+^ (DP) fraction [which is only abundant in that tissue; (52)]. Even when using high concentrations of purified cytokines, important findings obtained by using supernatants of HEK293T cells (Figures 7, 8, Supplementary Files 5C,D) were confirmed; for example, intestinal lymphocytes were hardly responsive to IL-2, and DP thymocytes were stimulated only by IL-2 (Figure 9 and Supplementary File 5E; highlighted by blue and magenta bars, respectively, in Figure 9). Also, the sensitivity of DN thymocytes to IL-15La+sIL-15Rα (in this case as RLI fusion form) was confirmed (highlighted by a red bar in Figure 9). However, now, at the highest tested concentration of purified IL-15La-RLI, also preparations of DN and CD8SP splenocytes, and CD4SP and CD8SP thymocytes, were detectably stimulated [highlighted by orange bars in Figure 9; more visible for thymocytes in Supplementary File 5E(b)]. An additional observation was that pSTAT5 levels in DN splenocytes were not very responsive to IL-2 treatment [highlighted with a green bar in Figure 9; see also Supplementary File 5C(f)].

### Trout IL-15 (+sIL-15Rα) Induces Expression of Type 1 Immunity Marker Genes in Trout Total Splenocytes but Trout IL-15L+sIL-15Rα Induces Expression of Type 2 Immunity Marker Genes

**Figure 10:**
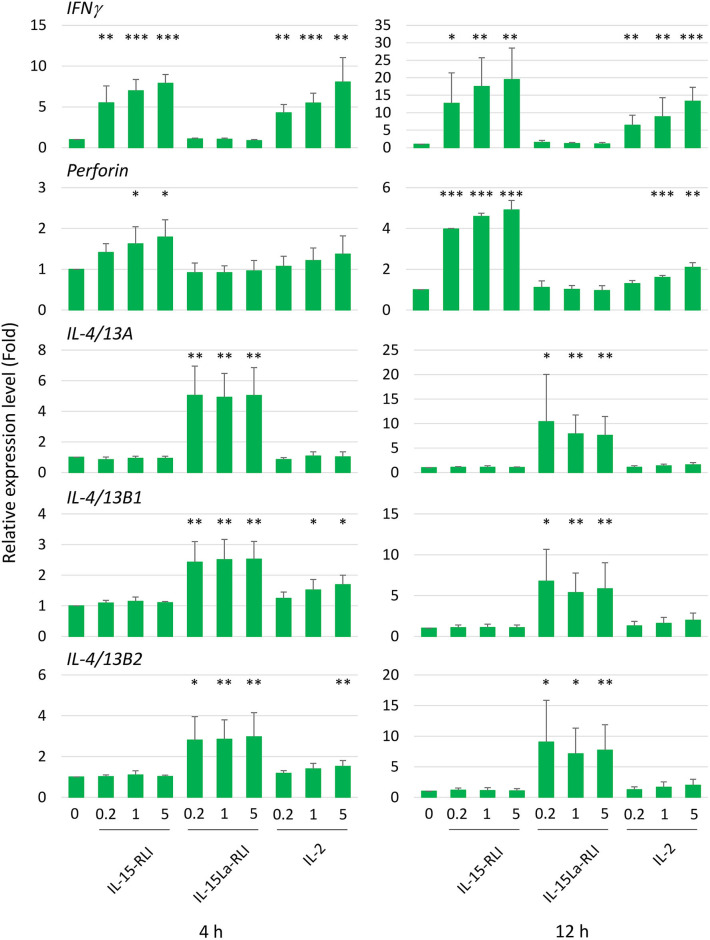
In trout total splenocytes, trout IL-15-RLI and IL-15La-RLI selectively enhanced expression of signature genes for type 1 and type 2 immunity, respectively; trout IL-2 enhanced genes of either signature. Relative expression levels of *IFN*γ, *perforin, IL-4/13A, IL-4/13B1*, and *IL-4/13B2* in trout total splenocytes were measured by RT-qPCR after incubation for 4 and 12 h with purified recombinant trout cytokines IL-2, IL-15-RLI, and IL-15La-RLI (produced in insect cells) at 0.2, 1, and 5 nM. Expression levels were normalized to *EF1A* expression and the values for the mock-treated control were set to 1 in each experimental panel. The average values of four biological experiments are shown together with error bars representing SD. In cases in which the average value was more than 1.5-fold higher than in the matching controls, one, two, or three asterisks indicate *p*-values smaller than 0.05, 0.01, or 0.001, respectively, based on paired samples T-test for the log-adjusted values of relative expression levels in samples and matching controls (54). A table with the underlying Ct values is shown in Supplementary File 6A.

After preliminary experiments, judging the technical feasibility and reproducibility of experiments and results, and the fact that splenocytes were sensitive to IL-15L+sIL-15Rα as shown by the pSTAT5 analysis (Figure 9), we decided to concentrate on trout splenocytes for further RT-qPCR analysis after cytokine stimulation. Purified trout IL-2, IL-15-RLI, and IL-15La-RLI were incubated at 0.2, 1, and 5 nM concentrations with total splenocytes, and after 4 h and 12 h incubation the RNA of the cells was isolated and subjected to RT-qPCR analysis to assess the expression levels of type 1 immunity marker genes *interferon* γ (*IFN*γ) and *perforin*, and type 2 immunity marker genes *IL-4/13A, IL-4/13B1*, and *IL-4/13B2*. IL-2 significantly enhanced *IFN*γ, *perforin, IL-4/13B1*, and *IL-4/13B2*; IL-15-RLI significantly enhanced *IFN*γ and *perforin*; and IL-15La-RLI significantly enhanced *IL-4/13A, IL-4/13B1*, and *IL-4/13B2* (Figure 10). To ensure that the observations were not caused by preparation artifacts, similar experiments were performed with supernatants of transfected HEK293T cells. The results (Supplementary File 6B) are comparable to those in Figure 10, and provide the important additional observations that non-covalent complexes between IL-15La and sIL-15Rα, and between IL-15Lb and sIL-15Rα, also specifically enhanced expression of *IL-4/13A, IL-4/13B1*, and *IL-4/13B2*. A further finding, consistent with the pSTAT5 assay results (Figures 7, 8, Supplementary Files 5C,D), was that IL-15 with and without sIL-15Rα seemed to have similar potencies in enhancing *IFN*γ and *perforin* expression, but that IL-15La and IL-15Lb fully depended on co-expression with sIL-15Rα for function (Supplementary File 6B).

### Trout IL-15La-RLI Efficiently Induces Type 2 Immunity Marker Gene Expression in CD4^−^CD8^−^IgM^−^ Splenocytes

**Figure 11:**
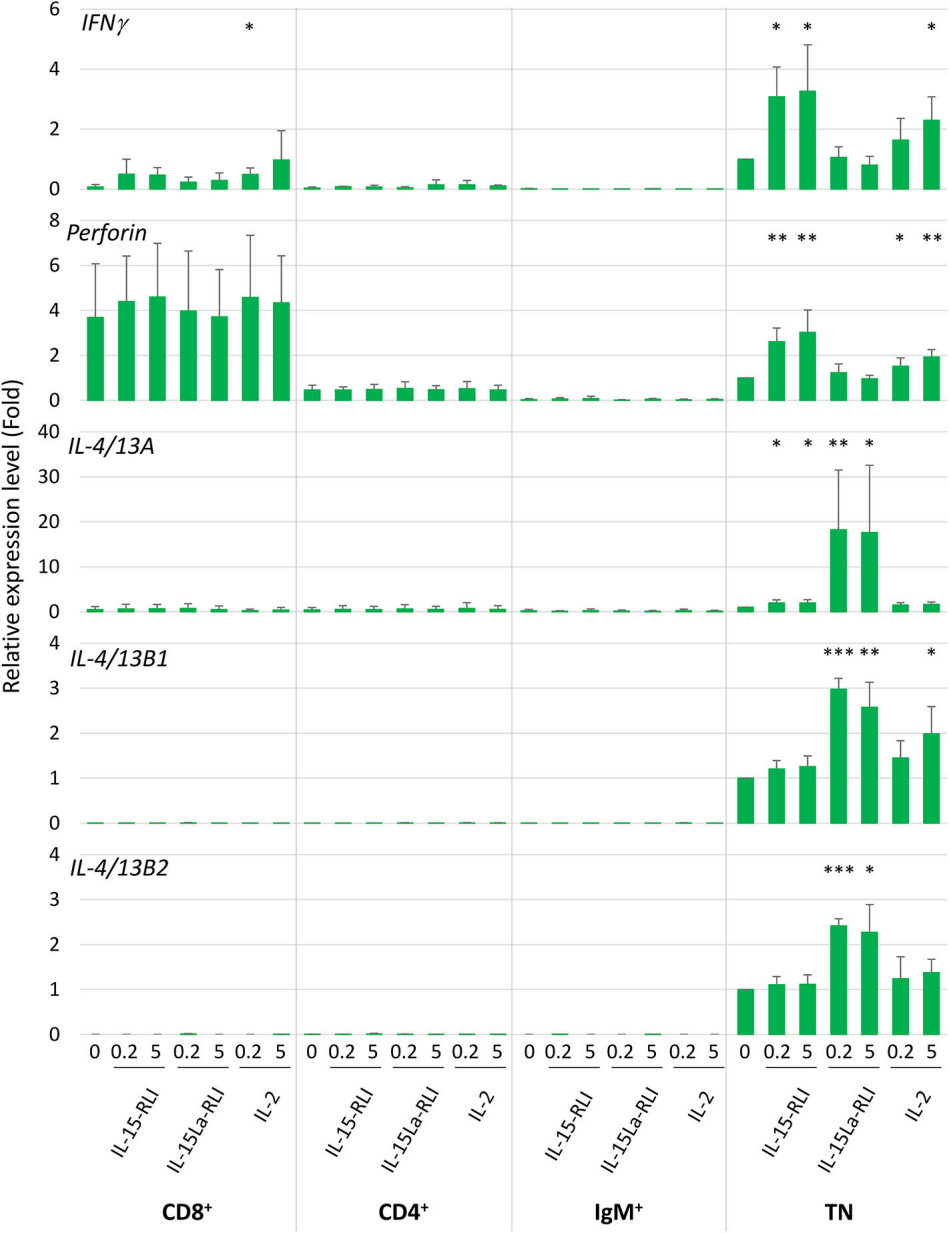
In trout CD4^−^CD8^−^IgM^−^ splenocytes, trout IL-15La-RLI selectively enhanced expression of signature genes for type 2 immunity; trout IL-15 predominantly enhanced expression of signature genes for type 1 immunity, and trout IL-2 enhanced genes of either signature. Relative expression levels of *IFN*γ, *perforin, IL-4/13A, IL-4/13B1*, and *IL-4/13B2* in CD4^+^, CD8^+^, IgM^+^, and CD4^−^CD8^−^IgM^−^ (TN) trout spleen morphological lymphocytes were measured by RT-qPCR after incubation for 12 h with purified recombinant trout cytokines IL-2, IL-15-RLI, and IL-15La-RLI (produced in insect cells) at 0.2 and 5 nM. Expression levels were normalized to *EF1A* expression and the values for the mock-treated TN control were set to 1 in each experimental panel. The average values of four biological experiments are shown together with error bars representing SD. Asterisks indicate cases with estimated significance as described for Figure 10. A table with the underlying Ct values is shown in Supplementary File 6C(a). For depiction at a larger scale and an alternative analysis of the results for the CD4^+^, CD8^+^, and IgM^+^ cells, see Supplementary Files 6C(b) and 6C(c).

An additional stimulation experiment was performed using 0.2 and 5 nM concentrations of purified trout IL-2, IL-15-RLI, and IL-15La-RLI for stimulation of sorted CD4^+^, CD8^+^, IgM^+^, and CD4^−^CD8^−^IgM^−^ (triple negative or TN) fractions of spleen morphological lymphocytes. On average, the relative abundancies of each of the four fractions were: 24% CD4^+^ cells, 6% CD8^+^ cells, 39% IgM^+^ cells, and 31% TN cells (Supplementary File 3E). From previous studies it follows that, as in mammals, and although probably none of the populations was fully homogeneous, the trout CD4^+^ cells included helper and regulatory TCRαβ^+^ T cells (35, 52, 55), the CD8^+^ cells, which besides CD8αβ cells might also comprise CD8αα cells, included cytotoxic TCRαβ^+^ T cells (51, 56), the IgM^+^ cells probably predominantly represented IgM^+^ B cells [e.g., (57)], and the TN cells probably were a mixture of several cell populations such as NK cells, innate lymphoid cells (ILCs), IgT B cells, and thrombocytes [e.g., (57–60)]. RT-qPCR analysis revealed that among the four populations, the TN cells expressed the highest constitutive and cytokine-induced expression levels of *IFN*γ, *IL-4/13A, IL-4/13B1*, and *IL-4/13B2* (Figure 11). The highest constitutive levels of *perforin* were found in CD8^+^ cells (Figure 11), but, for interpretation at the single cell level, it should be realized that this may be a more homogenous population than the TN cells. Only in the TN cells the *perforin* levels were found significantly enhanced after cytokine stimulation (Figure 11 and Supplementary File 6C). Expression patterns induced by the individual cytokines were similar as observed for total splenocytes (Figure 10), with IL-15-RLI efficiently enhancing the type 1 immunity marker genes *IFN*γ and *perforin*, with IL-15La-RLI efficiently enhancing the type 2 immunity marker genes *IL-4/13A, IL-4/13B1*, and *IL-4/13B2*, and with IL-2 efficiently enhancing the type 1 immunity marker genes *IFN*γ and *perforin* but also the type 2 immunity marker gene *IL-4/13B1* (Figure 11). Different from the observations for trout total splenocytes (Figure 10), however, was that IL-15-RLI was found to have (*p* < 0.05) a stimulatory effect on *IL-4/13A* expression by TN cells, although the levels of *IL-4/13A* induced by IL-15-RLI were much lower than induced by IL-15La-RLI (Figure 11). Such IL-15 activity would be in agreement with some reports for mammals, since although the overall dominant effect of mammalian IL-15 is the stimulation of type 1 immunity (7, 31, 32), in isolated experiments mammalian IL-15 was found able, for example, to induce the expression of the type 2 immunity cytokine IL-4 in mast cells (61).

IL-2 and IL-15 are known as important growth and survival factors for distinct populations of lymphocytes (3, 25, 30, 31, 62), and the observation in the present study that there is no stringent correlation between cytokine-mediated induction of pSTAT5 and marker gene expression (compare Figure 9 and Figure 11) may relate to the fact that cell growth/survival and cell functional activity are not identical processes. It should also be realized that mammalian IL-2 and IL-15 can activate more transcription factors than only their dominantly activated transcription factor STAT5 (15, 17, 61), and future studies should establish antibodies for allowing a more extensive analysis of activated transcription factors in fish. Future research in fish should also try to establish antibodies against potential receptors of the IL-2/15/15L family and other cell surface markers so that sensitive cell populations can be further characterized.

### *In vivo* Confirmation That Trout IL-15La-RLI Efficiently Induces Type 2 Immunity Marker Gene Expression

**Figure 12:**
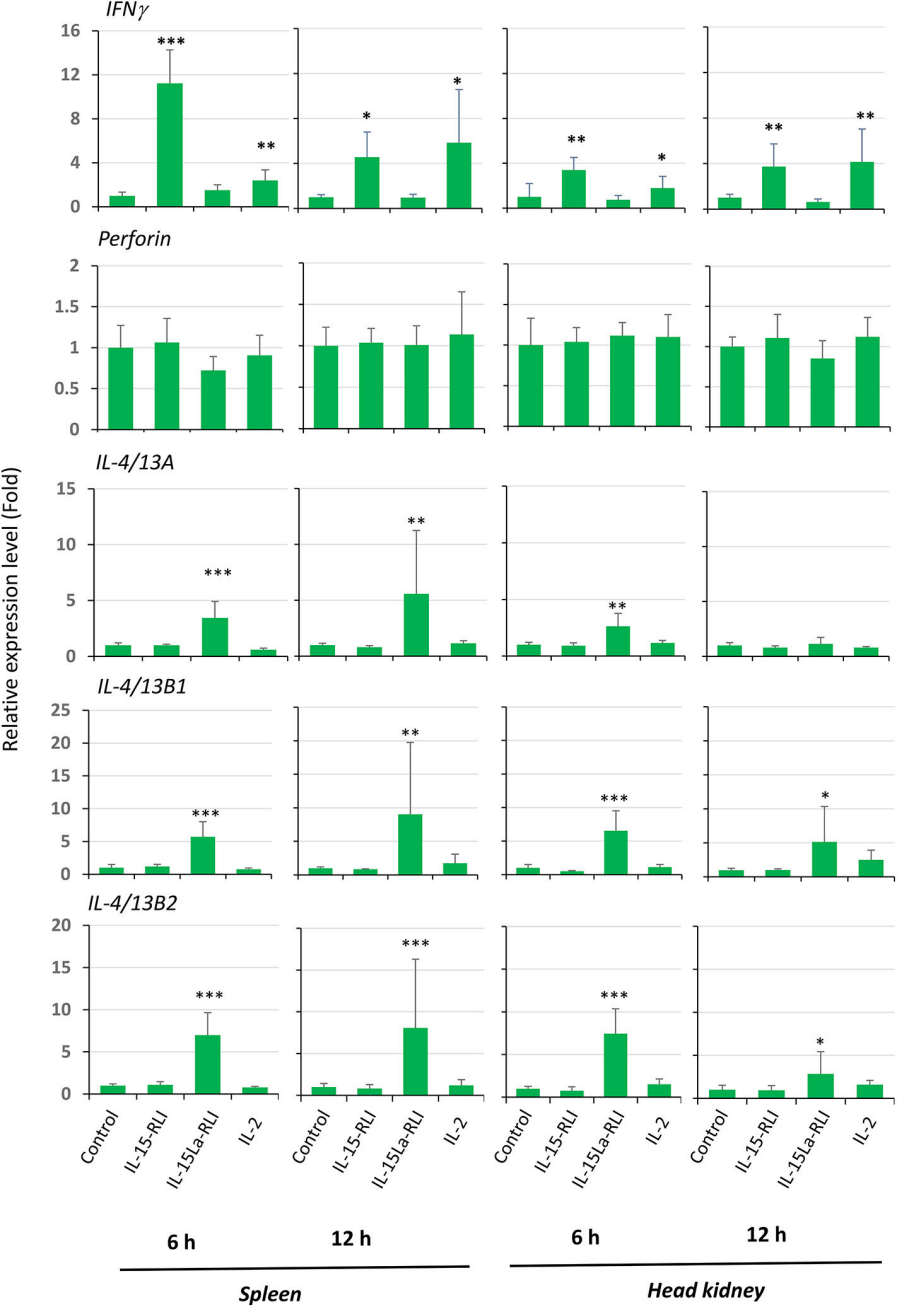
*In vivo*, in trout spleen and head kidney, trout IL-15-RLI selectively enhanced expression of *IFN*γ, whereas trout IL-15La-RLI selectively enhanced expression of *IL-4/13* genes; trout IL-2 enhanced both types of genes, although statistical significance was only observed for the *IFN*γ enhancement. Relative expression levels of *interferon* γ (*IFN*γ), *perforin, IL-4/13A, IL-4/13B1*, and *IL-4/13B2* were measured by RT-qPCR in the spleen and head kidney of rainbow trout isolated at 6 h and 12 h post intraperitoneal injection with 50 μl (1 μM) purified recombinant trout cytokines IL-2, IL-15-RLI, or IL-15La-RLI (produced in insect cells). *N* = 5 per group. Expression levels were equilibrated against *EF1A* expression and the values for the buffer control were set to 1 in each experimental panel. The average values of each group are shown together with error bars representing SD. Asterisks indicate cases with estimated significance as described for Figure 10. A table with the underlying Ct values is shown in Supplementary File 6D.

Ten juvenile rainbow trout each were injected intraperitoneally with 50 μl (1 μM) recombinant IL-2, IL-15-RLI, or IL-15La-RLI purified from insect cells, or with buffer control. At 6 h and 12 h the spleen and head kidney were harvested from five fish per treatment, and the RNA was isolated and subjected to RT-qPCR analysis for expression of *IFN*γ, *perforin, IL-4/13A, IL-4/13B1*, and *IL-4/13B2*. The results confirmed that IL-15-RLI is efficient in inducing a type 1 immune response, namely by enhancing *IFN*γ expression, and that IL-15La-RLI is efficient in inducing a type 2 immune response, namely by enhancing *IL-4/13A, IL-4/13B1*, and *IL-4/13B2* expression (Figure 12). IL-2, reminiscent of the above described *in vitro* results (Figures 10, 11), enhanced the expression of *IFN*γ as well as of *IL-4/13B1* and *IL-4/13B2*, although the latter could not be concluded with statistical reliability (Figure 12). In contrast to the *in vitro* results (Figures 10, 11), *in vivo* the expression of *perforin* was not sensitive to the cytokine treatment (Figure 12).

As extra confirmation of the reliability of the results, the “spleen 6 h” RNA samples were pooled per five identically-treated trout and analyzed by next generation sequencing (NGS). The number of reads specific for the cytokine genes were too few for performing statistical analysis, but the observations agreed with IL-15-RLI efficiently inducing *IFN*γ expression vs. IL-15La-RLI efficiently inducing *IL-4/13A, IL-4/13B1*, and *IL-4/13B2* expression (Supplementary File 8).

## Discussion

The current study shows within species, and cross-species, interactions between the cytokines trout IL-2, IL-15, IL-15La, and IL-15Lb, and bovine IL-15 and IL-15L, and the receptor chain IL-15Rα of both cattle and trout (Figure 5). We are not aware of any other reports directly showing fish-mammalian cross-species interactions between cytokines and receptor chains, or between cytokines and their heterodimer complex partners. Trout and cattle shared their last common ancestor around 416 million years ago (39), emphasizing how ancient the IL-2/15/15L-to-IL-15Rα interaction system is. The result was not unexpected, because residues in IL-15 and IL-15Rα for ligand-receptor binding are very well-conserved from cartilaginous fish to mammals, and the respective IL-15 residues are also well-conserved in IL-15L and in fish IL-2 (Figure 3A) (13). Whilst mammalian IL-15 binds IL-15Rα with an unusually high affinity (5, 20), mammalian IL-2 binds IL-2Rα with much lower affinity (20), agreeing with the relatively poor conservation of the relevant binding residues among tetrapod IL-2 and IL-2Rα (Figure 3A) (13), and the differences in stability of free mammalian IL-2 and IL-15 (63–65). It was estimated that *IL-2R*α originated from an *IL-15R*α duplication early in tetrapod evolution (13, 35), but when in tetrapod evolution IL-2 and IL-2Rα acquired their mutual specificity (Figure 5) (19, 20, 66) is unclear. For example, chicken IL-2 (67) is still very similar to IL-15 (Figure 3A) and in the past was even mistaken for it (68), and it would be interesting to investigate its alpha receptor chain binding specificity.

The stable secretion of human IL-15 is significantly enhanced by co-expression with soluble IL-15Rα (65, 69). Likewise, stable secretion of bovine and trout IL-15 and IL-15L was largely enhanced by co-expression with soluble IL-15Rα (Figure 6). Furthermore, as found for human IL-2 (41), bovine and trout IL-2 were stably secreted in the absence of co-expression with the respective soluble receptor alpha chain, IL-2Rα or IL-15Rα (Figure 6). Therefore, it can be concluded that during evolution the propensities of IL-2 to act as a free cytokine and of IL-15 and IL-15L to behave as a “heterodimer” with IL-15Rα were already established at the level of fish. Compared to IL-15, IL-15L appears to be even more dependent on in *trans* presentation with IL-15Rα than found for IL-15, both in regard to apparent stability (Figure 6) and function (Figures 7, 8, Supplementary File 6B). In the literature, the established abilities of mammalian IL-2 to be presented in *trans* (70), and of mammalian IL-15 to function as a free cytokine [e.g., (21, 34)], are sometimes forgotten. However, that trout IL-2 was also readily found at the surface of IL-15Rα co-expressing cells (Figure 5) and trout IL-15 was also able to function as a free cytokine (Figure 7, Supplementary Files 5C, 6B), suggest that both the in *cis* and in *trans* pathways are functionally relevant ancient traits of both cytokines. Future studies should focus on the identification of the signaling receptors for the trout cytokines, and further investigate potential functional differences between the free and IL-15Rα-bound cytokine forms.

One important reason for the selection of IL-2 over IL-15 for acquiring a dominant role in T_reg_ stimulation during evolution was probably that its free diffusion can aid in the recruitment of T_regs_ to sites of inflammation (25). In a pufferfish, CD4^+^IL-15Rα^+^ naïve lymphocytes were found to express *FOXP3* and to have immunosuppressive functions, while CD4^+^IL-15Rα^−^ lymphocytes from this fish did not express *FOXP3* (35). Furthermore, the ability of zebrafish FOXP3 to induce T_reg_-like functions has been shown or suggested (71, 72). Therefore, despite the fact that fish do not have a separate IL-2Rα chain (13, 35), a preferred usage by fish IL-2 of IL-15Rα in *cis* may allow the cytokine to have a similarly important role in T_reg_ stimulation as in mammals. Different uses of the receptor alpha chain may also have caused, during evolution, IL-15 to be selected over IL-2 for important roles in the stimulation of lymphocytes of mucosal tissues (Figures 7, 9) (31, 73–77), because IL-15 presentation at the cell membrane allows the power of cytokine signaling to be retained within confined niches. In short, our data reveal that important characteristics relating to the mechanistic and functional “dichotomy” (16) observed for mammalian IL-2 and IL-15 were already established in a common ancestor of mammals and teleost fish.

Size exclusion chromatography (and also Western blot data) suggest that trout IL-2 molecules produced in insect cells form homodimers (Supplementary File 4F), and homodimer structures have also been described in some studies for recombinant mammalian IL-2 (53). Homodimer structures may also explain a large band observed upon Western blot analysis of trout IL-15La expressed in transfected mammalian cells (Figure 6, Supplementary Files 5A,B) or purified from insect cells (Supplementary File 4B), and there is evidence that, at least under some conditions, human IL-15 can form noncovalent homodimers (78, 79). A related short-chain four α-helix bundle cytokine for which homodimer formation is known is IL-5 (80), but IL-2 and IL-15 are generally considered to be monomers. However, given the indications for dimer formation in both fish and mammals, the possibility that IL-2/15/15L family cytokines may potentially form homodimers as a functionally relevant ancient trait should be critically evaluated in future studies.

*IL-15L* intact gene appears to have been lost in amphibians, birds, and many mammals (13). We have not found a function for bovine IL-15L as yet, and the present study is the first to report on IL-15L functions, including the ability of rainbow trout IL-15L to stimulate DN thymocytes and CD4^−^CD8^−^IgM^−^ splenocytes. We are not aware of any other ancient cytokine shared between fish and mammals for which the function hitherto was not known.

The developmental path of mammalian T lymphocytes within the thymus is from an early DN stage toward an intermediate DP stage, after which the cells mature to become CD4SP or CD8SP T cells that ultimately can leave the thymus (81). Fish thymocyte progressive development has not been studied in detail, but available knowledge of fish thymus organization, gene expression, and functions of mature T cells [e.g., (51, 52, 82, 83), reviewed in (55)] suggest a similar development to mammals. Probably, as in mammals (81), DN thymocytes in trout importantly consist of several stages of early T cells. In addition, as in mammals, the trout DN thymocytes likely include some B cells, although they are scarce in trout thymus [e.g., (51)], and, based on findings in mammals, may include several developmental stages of NK cells and ILCs, including multipotent precursors that may also develop into T cells (84, 85). Future research should try to identify more precisely the (sub-) population of fish DN thymocytes which is sensitive to IL-15L.

While most of the results obtained in the present study for trout IL-2 and IL-15 agree well with reports for mammals, an exception is the detected sensitivity of trout DP thymocytes to IL-2 (Figures 8, 9). In addition to being refractory to IL-2 and IL-15, mammalian DP thymocytes have low sensitivity to the STAT5 activating cytokine IL-7 (86, 87). Of relevance to these findings is the observation that in mice in which IL-7 sensitivity was induced at the DP stage (by genetic engineering), IL-7 stimulation could induce thymocyte development into mature CD8^+^ T cells in the absence of the normal requirement for positive selection mediated by TCR-pMHC interaction, thus bypassing a critical step in T cell education (88). Hence, it is puzzling that trout DP thymocytes are so sensitive to IL-2. Future work should try to determine whether this *ex vivo* finding has relevance within the fish thymus, try to discover where in the fish thymus IL-2 is expressed, and investigate whether the fish DP population can be divided into IL-2 responding and non-responding populations. Possibly, the IL-2-sensitive DP thymocytes are T_reg_ cells expressing relatively high levels of IL-15Rα [see mammalian study (89)], but antibodies against trout IL-15Rα which could help investigate this matter are not yet available.

Trout splenocytes were found sensitive to IL-15La-RLI as indicated by STAT5 phosphorylation (Figure 9), and these cells were chosen for a detailed analysis by RT-qPCR analysis. In total splenocytes, trout IL-2 enhanced expression of the type 1 immunity marker genes *IFN*γ and *perforin*, and also of the type 2 immunity marker genes *IL-4/13B1* and *IL-4/13B2* (Figure 10 and Supplementary File 6B), which is reminiscent of previous findings for IL-2 in trout (49, 90) and mammals (62, 91–93). In contrast, if using these target cells, trout IL-15, free or complexed with IL-15Rα, only induced the type 1 immunity marker genes *IFN*γ and *perforin* (Figure 10 and Supplementary File 6B), activities agreeing with previous findings for mammalian IL-15 (32, 94) and free trout IL-15 (43). Our most exciting novel finding is that trout IL-15Rα-complexed IL-15L only enhanced expression of the type 2 immunity marker genes *IL-4/13A, IL-4/13B1*, and *IL-4/13B2* (Figures 10, 11, Supplementary File 6B), and so can have an opposite immune function relative to IL-15. This contrasting effect of IL-15 and IL-15L was also confirmed *in vivo* (Figure 12 and Supplementary File 8). When separating trout spleen lymphocyte subpopulations using antibodies against CD4, CD8, and IgM, the highest levels of *IL-4/13A, IL-4/13B1*, and *IL-4/13B2* expression were found for CD4^−^CD8^−^IgM^−^ cells, especially after stimulation with IL-15La-RLI (Figure 11), suggesting that this cell population contains a subpopulation which is very important for type 2 immunity. Based on comparison with mammalian studies, and recent indications for the existence of such cells in fish (60), we suspect that these cells are similar to mammalian type 2 innate lymphoid cells (ILC2) which are specifically dedicated to type 2 immunity [reviewed in (95)]. Meanwhile, after stimulation with trout IL-15-RLI, the trout CD4^−^CD8α^−^IgM^−^ splenocytes upregulated *IFN*γ and *perforin* (Figure 11), perhaps involving a cell subpopulation similar to mammalian NK cells because these cells are particularly sensitive to IL-15 (7, 31, 32, 94). Neither ILC2 nor NK cells have been properly identified in fish, and the present study provides additional support for their existence. The *IL-4/13* genes are homologs of mammalian *IL-4* and *IL-13* (96, 97), and IL-15L is the first cytokine found to specifically induce their expression in fish. In mammals, the cytokines TSLP, IL-25, and IL-33 are important for stimulating ILC2 cells, and we speculate that absence of one or more of these molecules in fish, as their genes have not been detected so far (55), may explain the stricter evolutionary conservation of IL-15L in fishes compared to tetrapod species.

**Figure 13:**
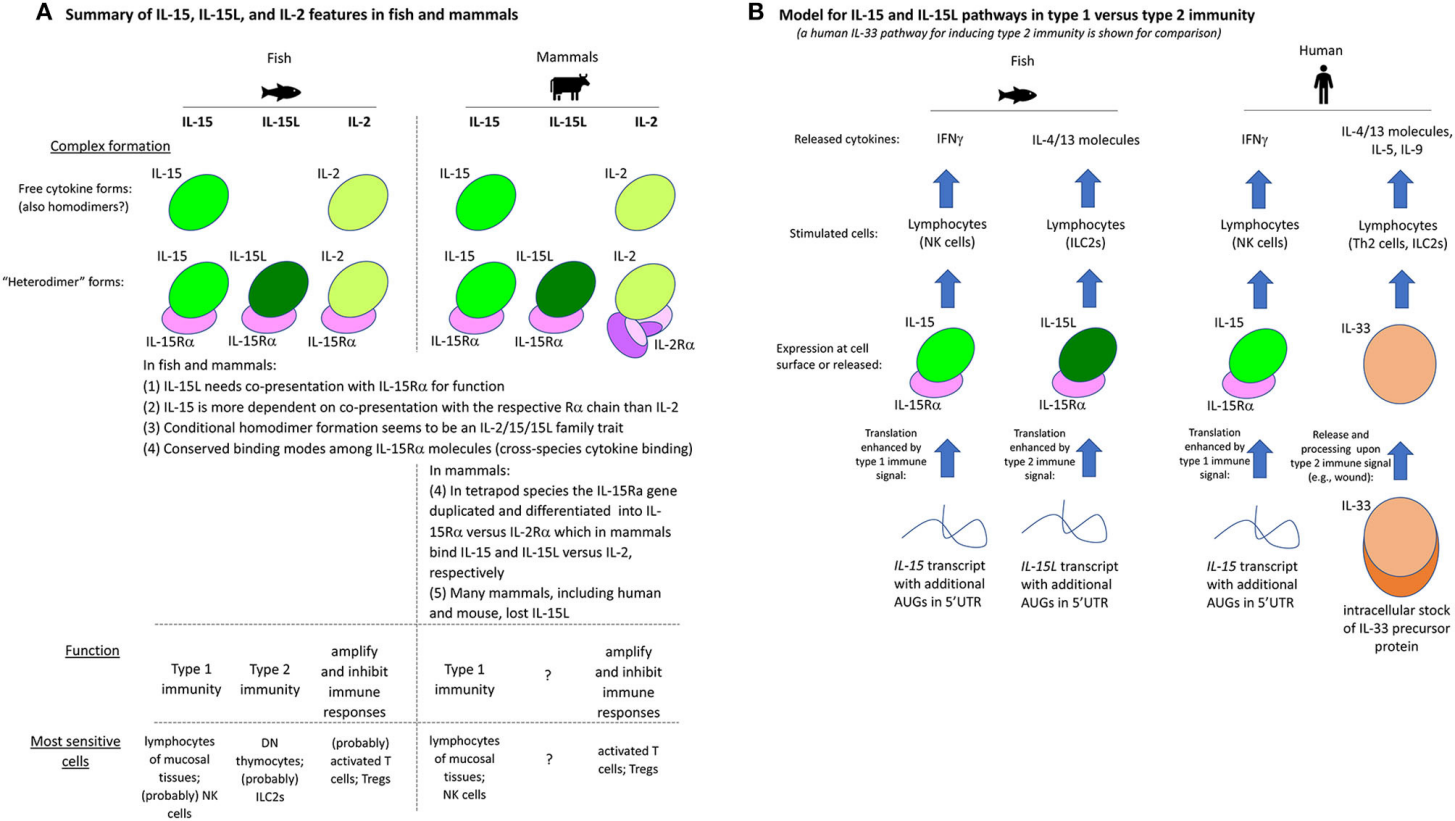
**(A)** Summary of IL-15, IL-15L, and IL-2 features in fish and mammals. **(B)** Model for IL-15 and IL-15L pathways in type 1 vs. type 2 immunity (a human IL-33 pathway for inducing type 2 immunity is shown for comparison).

For convenience of the reader, we have summarized the experimental results of the present study for the trout IL-2/15/15L cytokines in Table 2. Furthermore, Figure 13A provides a schematic comparison between the features of these cytokines in fish and mammals, and Figure 13B provides a model of how we envision that IL-15 vs. IL-15L may predominantly function in stimulating type 1 vs. type 2 immunity; important in that model is a yet unknown regulation at the transcript translation level, to which future research should be dedicated.

**Table 2:** Data obtained in the present study for rainbow trout IL-15La, IL-15Lb, IL-15, and IL-2.

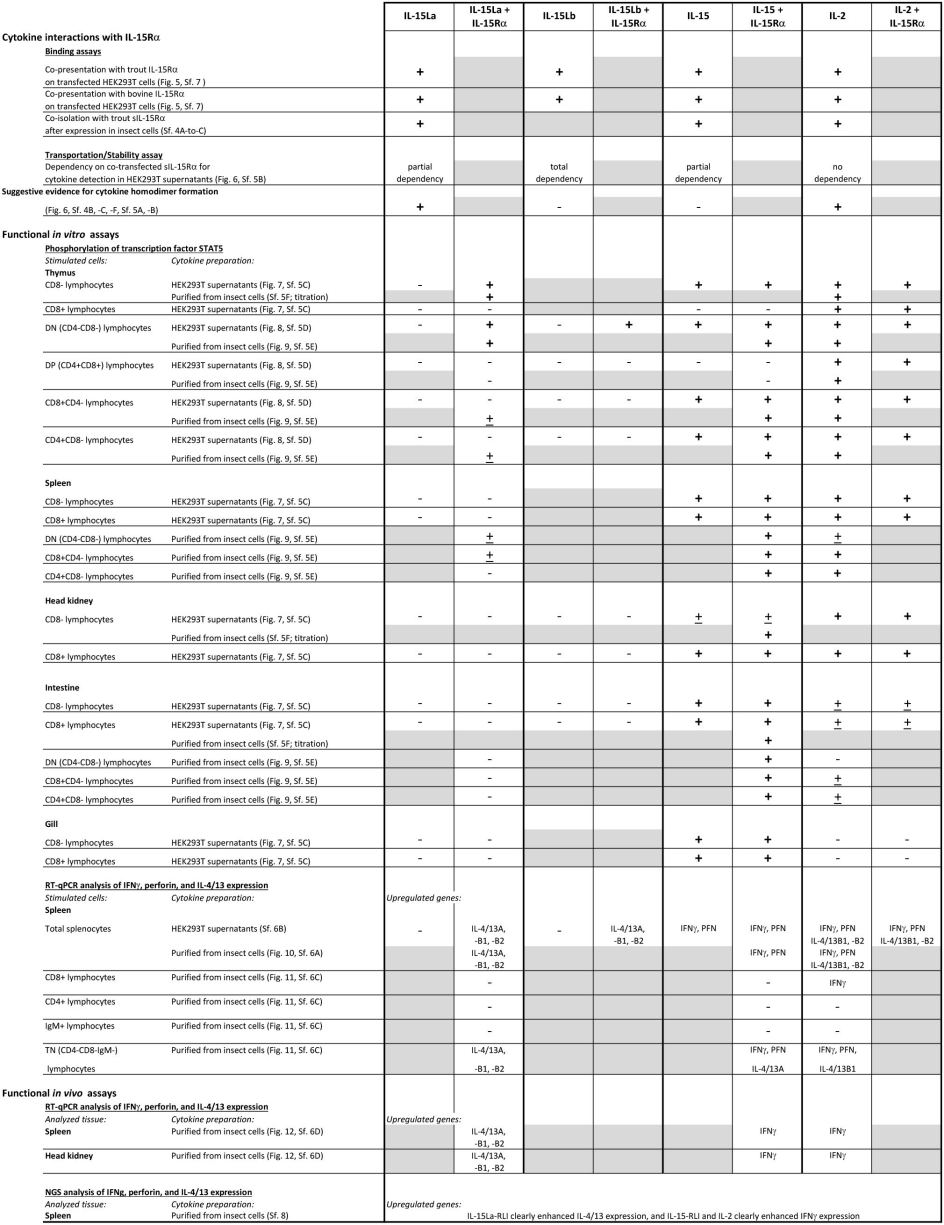

*The proteins were tested in combination with or without soluble rainbow trout IL-15Rα*.

*+, clearly positive reaction; -, no reaction; ±, weak reaction*.

*When expressed in insect cells, IL-15La + IL15Rα, or IL-15 + IL-15Rα, were expressed as genetic fusion (receptor-linker-interleukin; RLI) forms*.

*Figures and Supplementary files (Sf.) with the respective information are indicated between brackets*.

*Gray fields represent absence of experiments or not applicable*.

In conclusion, the present study reveals that the mechanistic and functional dichotomies between IL-2 and IL-15 are an ancient phenomenon, as evidenced by their conservation in both fish and mammals. Furthermore, we identified an unexpected cytokine playing a role in the type 2 immunity cytokine cascade in fish, namely IL-15L, which is closely related to the type 1 immunity cytokine IL-15. These findings are an important step in characterizing the IL-2/15/15L cytokine family and for understanding the original blueprint of the cytokine network in jawed vertebrates.

## Materials and Methods

### Rainbow Trout

Rainbow trout (*Oncorhynchus mykiss*) weighing between 80 and 300 gram were used in this study at three different facilities:

1. Inland Station, National Research Institute of Aquaculture (NRIA; Mie, Japan). Fish were fed commercial dry pellets and kept in 15°C flow-through water. For semi-quantitative RT-PCR analysis, a trout individual (Trout-1) of strain Tokyo, Tokyo Metropolitan Fisheries Experimental Station (Tokyo, Japan), was investigated. For determining IL-15La and IL-15Lb sequences, 5'-RACE analysis and semi-quantitative RT-PCR, a trout individual (Trout-2) of the homozygous clonal rainbow trout strain C25 was used. These homozygous isogeneic trout had been produced from outbred strain Nagano at the Nagano Prefectural Fisheries Experimental Station (Nagano, Japan), by gynogenesis over two generations by suppression of mitosis and meiosis in the first and second generations, respectively (98). Clonality had been confirmed by DNA fingerprinting. For convenient propagation of the strains, some of the gynogenetic animals had been subjected to a treatment with methyltestosterone and developed as homozygous neomales.

2. Scottish Fish Immunology Research Center (SFIRC), the University of Aberdeen, UK. Fish were fed commercial dry pellets and kept in 15±1°C recirculating water. The six trout individuals used for RT-qPCR analysis had been purchased from the Mill of Elrich Trout Fishery (Aberdeenshire, Scotland, UK).

3. Friedrich-Loeffler-Institut (FLI), Federal Research Institute for Animal Health (Insel Riems-Greifswald, Germany). Fish were fed commercial dry pellets and kept at 15°C in a partially recirculating water system. The investigated trout individuals belonged to the homozygous clonal strain C25 (see above). Most of the experiments described in the present study were done at the FLI.

Fish handling and experimental protocols complied with the guidelines for animal welfare in the respective countries and institutes.

### Rainbow Trout Permanent Cell Lines and Primary Head Kidney (HK) Macrophage Cultures

Four rainbow trout cell lines were used for gene expression analysis: a monocyte/macrophage-like cell line RTS-11 from spleen (99), an epithelial cell line RTL from liver (100), a fibroblastic cell line RTG-2 from gonad (101), and an epithelial cell line RTGill from gills (102). Cells were maintained in Leibovitz (L-15) medium (Invitrogen) containing 30% fetal bovine serum (FBS; Labtech International, for RTS-11 cells) or 10% FBS (for the other three cell lines and for primary HK macrophages) and antibiotics (100 U penicillin/ml and 100 μg streptomycin/ml; Invitrogen) at 20°C. Primary HK macrophage cultures from four individual trout at the SFIRC were prepared as outlined by Costa et al. (103).

### Permanent Human and Insect Cell Lines

#### HEK (Human Embryo Kidney) 293T Cells

HEK293T cells were used for transient expression of recombinant proteins. Cells were maintained in minimal essential medium (MEM) supplied with 10% FBS at 37°C in a 2.5% CO_2_ atmosphere.

#### High Five and Sf9 Cells

Two insect cell lines, High Five and Sf9, were used for producing recombinant proteins. These cells were maintained in Grace's Insect medium supplied with lactalbumin hydrolysate, yeast extract and 5% FBS, at 26°C.

These cell lines and media were obtained from the Collection of Cell Lines in Veterinary Medicine (CCLV) at FLI.

### Database Searches and Analysis of Nucleotide and Deduced Amino Acid Sequences

BLAST similarity searches were performed on sequence datasets of the National Center for Biotechnology Information (NCBI; http://blast.ncbi.nlm.nih.gov/Blast.cgi) (104) and the Ensembl database of the European Bioinformatics Institute (EBI; https://www.ensembl.org/) (105). Retrieved sequences were analyzed using genetic analysis software GENETYX (Version 12.0.3) and FGENESH gene prediction software (www.softberry.com) (106). For deduced amino acid sequences, the leader peptides were predicted using SignalP (http://www.cbs.dtu.dk/services/SignalP/) software (107). Alignments of deduced amino acid sequences were performed manually, based on comparisons of more sequences, and considerations of gene and protein structures and of phylogeny (13), and also considering the clarity of the figure. For construction of a phylogenetic tree, see Supplementary File 1D.

Read numbers per 10^8^ reads of *IL-15La* and *IL-15Lb* were determined by similarity searches against tissue-specific single read archive (SRA) datasets using the BLAST search function at NCBI. For rainbow trout, the SRA datasets of Bioproject PRJEB4450 (NCBI datasets ERX297509-to-297524) (40), Bioproject PRJNA389609 (NCBI datasets SRX2894150-to-2894164) (108) and Bioproject PRJNA380337 (NCBI datasets SRX2668643-to-2668653 and SRX2668655-to-2668657; Norwegian University of Life Sciences) were investigated. For Atlantic salmon, the SRA datasets of Bioproject PRJNA260929 (NCBI datasets SRX1046658, SRX1052181, SRX1052182, SRX1052184, SRX1052187-to-1052192; Norwegian University of Life Sciences) and Bioproject PRJNA72713 (NCBI datasets SRX608567, SRX608569, SRX608571, SRX608574, SRX608575, SRX608579, SRX608583, SRX608588, SRX608594, SRX608599, SRX608607, SRX608616, SRX608620, SRX608621; University of Victoria) were investigated. The species-specific *IL-15La* or *IL-15Lb* ORF sequences were subjected to “Megablast” analysis (blastn) using default settings except that the “max target sequences” number was changed to 20,000 and the “word size” was changed to 64. To ensure specificity of the Megablast analysis, only matches with score values ≥187 for PRJEB4450, ≥185 for PRJNA389609 and PRJNA72713, ≥233 for PRJNA380337, ≥192 for Bioproject PRJNA260929 were counted.

### Isolation of RNA, Synthesis of cDNA, PCR Amplification, Sequencing and Cloning Into Expression Vectors

Total RNA samples of trout were isolated from tissues by two-fold purification with TRIzol (Gibco) and stored at the NRIA. Equal amounts of RNA were transcribed into cDNA using Superscript transcriptase (Invitrogen). A cDNA sample from spleen of Trout-2 was used for the amplification of the full-length *IL-15La* open reading frame (ORF) using primer set Trout_IL-15La_CDS and ExTaq polymerase kit (Takara) while a cDNA sample from gill of Trout-2 was used for the amplification of the full-length *IL-15Lb* ORF using primer set Trout_IL-15Lb_CDS. These primer sequences are shown in Supplementary File 9A. For 5'-RACE analysis cDNA samples were synthesized from total RNA of spleen and gill of Trout-2 using the SMARTER RACE cDNA amplification system (Clontech). The first PCR was performed using spleen cDNA (for IL-15La) or gill cDNA (for IL-15Lb) with NUP primer (provided with the kit) and a specific primer for corresponding gene. Subsequently, nested PCR was performed using each first PCR product with UPM primer (provided with the kit) and a specific inner primer for corresponding gene. The sequences of primers used for 5′-RACE analysis are shown in Supplementary File 9A. The first PCR schedule was 94°C for 5 min, 5 × (94°C for 30 s, 72°C for 1:30 min), 10 × (94°C for 30 s, 70°C for 30 s, 72°C for 1 min), 25 × (94°C for 30 s, 68°C for 30 s, 72°C for 1 min), 72°C for 7 min. After diluting the product of the first reaction (1/200), the amplification schedule for the nested PCR was 94°C for 5 min, 32 × (94°C for 30 s, 60°C for 1 min, 72°C for 30 s), 72°C for 7 min. The amplified *IL-15La* and *IL-15Lb* full-length ORF and 5'-RACE fragments were prepared for sequencing by standard TA-cloning with the pGEM T-Vector System (Promega). The sequences of multiple clones were determined by dideoxy chain termination method and using an automated sequencer to exclude PCR errors. Assembled sequences of the overlapping full-length ORF and 5′-RACE amplifications of rainbow trout *IL-15La* and *IL-15Lb* were deposited to GenBank and are available as accessions MK619679 and MK619680, respectively.

For semi-quantitative analysis of tissue distribution of transcripts, PCR was performed with the ExTaq polymerase kit, using equal amounts of cDNA solution as templates, and the primer sets Trout_IL-15La, Trout_IL-15Lb, and Trout_EF1A (Supplementary File 9B), for amplification of fragments of *IL-15La, IL-15Lb*, and *elongation factor 1 alpha* (*EF1A*), respectively. For the semi-quantitative PCR analysis of *IL-15La* and *IL-15Lb* expression, the amplification schedule was: 94°C for 5 min, 32 × (94°C for 30 s, 60°C for 30 s, 72°C for 40 s), 72°C for 7 min; and for *EF1A* amplification, the schedule was: 94°C for 5 min, 25 × (94°C for 30 s, 60°C for 30 s, 72°C for 30 s), 72°C for 7 min.

For construction of DNA expression vectors, gene sequences were amplified from cDNA or commercially ordered, and, often after PCR-mediated gene modifications, cloned into commercial DNA plasmid vectors by using appropriate restriction enzymes behind the CMV-IE promoter, or into the baculovirus transfer vector pFBD-P10Uhis-ieGFP behind the p10-promoter (for cloning details see Supplementary File 2). The vector pFBD-P10Uhis-ieGFP is based on the vector pFBDΔXhoI_Histag (109) which is a derivate of pFastBac-Dual (Invitrogen) in which the PolH promoter region was replaced by a CMV-IE promoter driven GFP expression cassette (Dr. Günther M. Keil, personal communication). The expression vectors were multiplied in *E. coli* and isolated by standard techniques. To check whether the sequences were correctly inserted, all DNA expression vectors were sequenced by dideoxy chain termination method and using an automated sequencer.

### Reverse Transcription Quantitative Real-Time PCR (RT-qPCR) Analysis of Trout IL-15La and IL-15Lb Tissue Distribution

Six healthy rainbow trout were used at the SFIRC for RT-qPCR analysis of *IL-15La* and *IL-15Lb* tissue distribution. The RNA preparations from trout tissues, cell lines, and primary HK macrophage cultures, and the following RT-qPCR analysis, were performed as described previously (54, 110). The relative expression levels of each IL-15L gene were normalized against the expression level of *EF1A*, a highly expressed gene widely used as house-keeping gene in gene expression analysis in salmonids. A common reference containing equal molar amounts of purified PCR products of trout *IL-15La, IL-15Lb*, and *EF1A* was used for the quantification. The primer sets used for amplification were Trout_IL-15La_qPCR, Trout_IL-15Lb_qPCR and Trout_EF1A_qPCR (Supplementary File 9B).

### Expression of Recombinant Proteins in Human HEK293T Cells

#### Transfection

HEK293T cells were transfected using X-tremeGENE HP DNA Transfection Reagent (Roche) as described in our previous study (111), with slight changes. For co-expression of cytokines with IL-15Rα or IL-2Rα, HEK293T cells at a 80–90% confluency were co-transfected with 2 μg of the cytokine-encoding plasmid together with 2 μg of IL-15Rα-encoding plasmid or IL-2Rα-encoding plasmid (in total 4 μg) per well. To express only cytokines or receptor α chains, HEK293T cells were co-transfected with 2 μg of the respective plasmid together with 2 μg of “empty” pcDNA3.1 or pRc/CMV2 commercial vector (Invitrogen). Negative control cells were transfected with 4 μg of empty vector. The recombinant molecules were expressed by using the following expression vectors (for sequences see Supplementary File 2): bovine IL-2, pRcCMV2-*Bos-IL-2-FLAG*; bovine IL-15; pRcCMV2-*Bos-IL-15-FLAG*; bovine IL-15L, pRcCMV2-*Bos-IL-15L-FLAG*; bov.IL-15Lhyb-h-RLI, pcDNA3.1-*IL-2-Lead-RLI-bov.IL-15Lhyb*; bovine (full-length) IL-15Rα, pcDNA3.1-*Bos-IL-15R*α*-Myc-His*; bovine soluble IL-15Rα (aka sIL-15Rα), pcDNA3.1-*Bos-solIL-15R*α*-Myc-His*; bovine (full-length) IL-2Rα, pcDNA3.1-*Bos-IL-2R*α*-Myc-His*; bovine soluble IL-2Rα (aka sIL-2Rα), pcDNA3.1-*Bos-solIL-2R*α*-Myc-His*; trout IL-2, pcDNA3.1-*trout-IL-2-FLAG*; trout IL-2(N), pcDNA3.1-*trout-IL-2-(non-tagged)*; trout IL-15, pcDNA3.1-*trout-IL-15-FLAG*; trout IL-15(N), pcDNA3.1-*trout-IL-15-(non-tagged)*; trout IL-15La, pcDNA3.1-*trout-IL-15La-FLAG*; trout IL-15La(N), pcDNA3.1-*trout-IL-15La-(non-tagged)*; trout IL-15Lb, pcDNA3.1-*trout-IL-15Lb-FLAG*; trout IL-15Lb(N), pcDNA3.1-*trout-IL-15Lb-(non-tagged)*; trout IL-15La-h-RLI, pcDNA3.1-*IL-2-Lead-RLI-trout-IL-15La*; trout (full-length) IL-15Rα, pcDNA3.1-*trout-IL-15R*α*-Myc*; trout soluble IL-15Rα (aka sIL-15Rα), pcDNA3.1-*trout-solIL-15R*α*-Myc*.

#### Analysis of Transfected HEK293T Cells by Flow Cytometry

In order to check the binding ability of receptor α-chains for each cytokine, HEK293T cells were co-transfected with plasmids encoding full-length (transmembrane) forms of IL-15Rα or IL-2Rα and plasmids encoding the cytokines, or, as negative controls, transfected with only one of these plasmids or with empty vector alone (see above). Two days after transfection, HEK293T cells were collected, washed and stained with mouse ANTI-FLAG M2 Monoclonal Antibody (Sigma) and anti-mouse IgG, IgM (H+L) secondary antibody conjugated with Alexa Fluor 488 (Thermo Fisher Scientific) diluted according to the manufacturer's instructions. The stained HEK293T cells were analyzed with a FACSCalibur flow cytometer (BD Biosciences). Conditions were adjusted by setting the thresholds for conjugate controls. Dead cells were excluded from analysis by propidium iodide (PI) staining. The data were analyzed using BD CellQuest Pro Software (BD Biosciences).

#### Expression of Soluble Cytokine (-Complexes) in HEK293T Cells

HEK293T cells were co-transfected with plasmids encoding soluble forms of IL-15Rα or IL-2Rα and plasmids encoding the cytokines, or transfected with only one of these plasmids, or (as negative control) with empty vector alone. Medium of HEK293T cells was replaced to EX-CELL Serum-Free Medium (Sigma) before transfection. Two days after transfection, 2 ml of supernatant was collected from each well, and filtered through a 0.22 μm pore PVDF membrane (Syringe Driven Filter Unit, Millex-GV). For analysis by Western blotting as shown in Figure 6 and Supplementary File 5B, the 2 ml supernatants were concentrated to 40–50 μl by ultrafiltration with a 3 kDa nominal molecular weight cutoff membrane (Amicon Ultracel - 3K, Millipore), yielding the “concentrated supernatant” samples. For leukocyte stimulation experiments, supernatants were used without concentration (“unconcentrated supernatant” samples). Remaining HEK293T cells in each well were collected, pelleted and lysed in 100 μl of NP40 Cell Lysis Buffer (Thermo Fisher Scientific) supplied with Protease Inhibitor Cocktail (Sigma), and used for further analysis as “cell lysate” samples.

### SDS-PAGE and Western Blotting

Fifteen μl of the samples were mixed with 5 μl of 4 × reducing Laemmli Sample Buffer (Bio-Rad), heated for 3 min at 95°C and electrophoresed using (unless mentioned otherwise) 12% poly-acrylamide gels and standard procedures (112) and with PageRuler Prestained Protein Ladder (Thermo Fisher Scientific) as molecular weight marker. After electrophoretic separation, proteins were either visualized by treatment with Coomassie Brilliant Blue (CBB) R-250 staining solution (BioRad) or prepared for Western blotting by transfer to Amersham Hybond P 0.45 PVDF membranes (GE Healthcare) using a Trans-Blot Turbo Transfer System (Bio-Rad). Membranes were blocked by incubation in StartingBlock (TBS) Blocking Buffer (Thermo Fisher Scientific) and subsequently incubated overnight at 4°C with mouse ANTI-FLAG M2 Monoclonal Antibody, Myc-Tag (9B11) Mouse mAb or Phospho-Stat5 XP Rabbit mAb (Cell Signaling Technology) specific for phosphorylated Tyr694 (Tyr694 and surrounding residues are conserved between trout and mouse STAT5), made up in blocking buffer. The membranes were washed with TBS/0.1% Tween-20, followed by incubations with HRP-Conjugated Goat Anti-mouse IgG (Pierce) or HRP-linked Anti-rabbit IgG (Cell Signaling Technology) in blocking buffer for 1–2 h. All antibodies were used at the concentrations recommended by the manufacturer. Bands were visualized by chemiluminescence reaction (SuperSignal West Pico Chemiluminescent Substrate, Thermo Fisher Scientific) and documented on a VersaDoc 4000 MP workstation (BioRad) using Quantity One software (BioRad). As a loading control, membranes which had been subjected to pSTAT5-detection were stripped by incubation in 0.1 M glycine-HCI buffer (pH 2.8) for 2 h with gentle shaking at room temperature and subsequently reprobed for actin using mAb C4 (Millipore).

### Expression of Recombinant Proteins in Insect Cells

#### Construction of Recombinant Bacmid DNA

Recombinant plasmids were isolated and transformed to DH10Bac competent cells (Invitrogen) with standard procedure, after which bacmid DNA was isolated. The recombinant plasmids are explained in Supplementary File 2, with the names of the encoded recombinant proteins and plasmids as follows: trout IL-2, pFBD-P10Uhis-ieGFP-*trout-IL-2-FLAG*; trout IL-15, pFBD-P10Uhis-ieGFP-*trout-IL-15-FLAG*; trout IL-15La, pFBD-P10Uhis-ieGFP-*trout-IL-15La-FLAG*; trout soluble IL-15Rα (aka sIL-15Rα), pFBD-P10Uhis-ieGFP-*trout-solIL-15R*α*-Myc*; trout IL-15-RLI, pFBD-P10Uhis-ieGFP-*trout-IL-15-RLI*; trout IL-15La-RLI, pFBD-P10Uhis-ieGFP-*trout-IL-15La-RLI*.

#### Transfection of Recombinant Bacmid DNA Into High Five Insect Cells

High Five cells were seeded into a 6-well plate and incubated at 26°C for 1 h. A transfection mix with 5 μg bacmid DNA and 6 μl X-tremeGENE reagent in 100 μl α-MEM (Sigma) was prepared for each cytokine and incubated at room temperature for 40 min. These transfection mixes were diluted with 900 μl Insect-XPRESS medium, and then dropped onto the High Five cells. After 5 h incubation, the supernatant was replaced by 2 ml of fresh Insect-XPRESS medium per well and continued to be cultured. After 3 days cultivation, cells and supernatants were collected and stored at −80°C.

#### Isolation of Recombinant Baculoviruses by Plaque Assay

Sf9 cells were seeded into 6-well plates and incubated for 30 min at room temperature. Aliquots of the transfected High Five cells and their supernatants were thawed and diluted from 10^0^ to 10^−2^ in Grace's insect medium, and 100 μl of each dilution was added to the well. After 1 h cultivation at 26°C, supernatants were removed and cultures were overlaid with 1% low-melting agarose containing Grace's insect medium. After 3 days cultivation, GFP-positive plaques were picked and resuspended individually in 1 ml of Grace's insect medium. Each resuspended plaque was transferred into flasks with 10^5^ Sf9 cells to be infected. Infection progress was monitored by GFP fluorescence. After 5–7 days cultivation, Sf9 cells and their supernatants were collected, aliquoted and kept at −80°C as recombinant baculovirus stocks.

#### Titration of Recombinant Baculoviruses by Endpoint Dilution Assay

Aliquots of recombinant baculovirus stocks were thawed and diluted from 10^−1^ to 10^−8^ in Grace's insect medium, and 100 μl of each virus dilution was pipetted into 96-well plates in quadruplicate. Subsequently, 6 × 10^4^ freshly harvested Sf9 cells/well were added. After 5–7 days incubation at 26 °C, the numbers of GFP-positive wells were counted and virus titers were calculated as endpoint dilution assay TCID50 [TCID_50_ = D ^(n/p+0.5)^ × 1/sample volume (ml); *D* = dilution factor; *n* = number of positive wells; *p* = number of parallel values].

#### Infection of Sf9 Cells With Recombinant Baculoviruses

To obtain recombinant cytokines, Sf9 cells were infected in suspension (1 × 10^6^ /ml) or in T175 flasks with Insect-XPRESS medium. For suspension culture, 0.1% Pluronic-F68 (Gibco) was added. For infection with recombinant baculoviruses encoding IL-15-RLI or IL-15La-RLI an MOI of 2–3 was used, while infection with recombinant baculovirus encoding IL-2 was carried out with an MOI of 0.5 in order to reduce aggregation events. After 4–6 days incubation at 26°C, supernatants were collected, filtered through a 0.22 μm membrane and kept at 4°C until further analysis or purification.

### Purification of Recombinant Cytokines From Insect Cell Supernatants

Filtered supernatants from infected Sf9 cells were mixed with ANTI-FLAG M2 Affinity Gel (Sigma) at a ratio of 600:1 (e.g., 300 ml of supernatant was mixed with 0.5 ml of affinity gel). After overnight incubation at 4 °C, the mixtures were poured into 10 ml columns with 35 μm filter pore size (MoBiTec), washed with ~150 ml of TBS and eluted six times with 1 ml aliquots of 0.1 M glycine HCl, pH 3.5 into vials containing 20 μl of 1 M Tris, pH 8.0. Subsequently the buffer was exchanged and concentrated to 300 μl PBS (-) using Amicon Ultracel - 3K centrifugal filters. Protein concentrations were determined with Pierce BCA Protein Assay Kit (Thermo Fischer Scientific) according to the manufacturer's instructions. Amount of substance (mol) for each recombinant protein was calculated based on the protein amount in the preparations and molecular weight. The molecular weight of recombinant proteins was estimated based on their amino acid sequences using Compute pI/Mw tool (https://web.expasy.org/compute_pi/). Purified recombinant proteins were analyzed immediately by Western blotting or gel filtration chromatography, or they were supplemented with 0.1% Bovine serum albumin (BSA) and 50% glycerol (recombinant protein storage buffer) for storage at −20°C.

### Gel Filtration Chromatography

To analyze purified recombinant proteins, gel filtration chromatography was carried out on a Superose 12 column (30 cm length, 1 cm diameter, Pharmacia) using an HPLC system (BT 9200 Titan pump, BT 9520 IN UV monitor, Eppendorf Biotronik) for solvent delivery and UV monitoring at 280 nm. For the equilibration with PBS (-) and for the separation of proteins, a constant flow rate of 0.5 ml/min was maintained throughout the experiment. Samples of recombinant proteins containing between 10 and 15 μg were diluted in 0.5 ml PBS (-), injected using a 0.5 ml sample loop (Rheodyne), and fractionated (1 fraction/0.5 ml). The fractionated samples corresponding to peaks in the chromatogram were concentrated to 40–50 μl by ultrafiltration with an Amicon Ultracel - 3K filter and were analyzed by SDS-PAGE and Western blotting. The calibration of the column was carried out using standard procedures and the same chromatographic conditions as described for the recombinant proteins. The void volume was estimated with Blue dextrane (Sigma). For the calibration, elution volumes of calibrant proteins (Sigma) were plotted against the decadic logarithms of their molecular weights and linear regression models were calculated which were then used for the determination of molecular weights of the recombinant proteins.

### Deglycosylation Assay

PNGase-F digestion of the lysates of transfected HEK293T cells and purified recombinant cytokine preparations was performed using the PNGase-F kit (New England Biolabs) with similar procedures as described in our previous study (111). Prior to assay, the concentration of purified recombinant protein preparations in PBS (-) was adjusted to ~500 ng/30 μl. Nine μl of the cell lysates or the recombinant cytokines were subjected to assay as suggested by the manufacturer, and incubated in the presence or absence (mock control) of PNGase-F. Digested samples were subjected to buffer exchange to PBS (-) and concentrated to 40–50 μl by ultrafiltration with an Amicon Ultracel - 3K filter. SDS-PAGE and Western blotting analysis were performed as described above except for using 16% poly-acrylamide gels for analysis of the purified cytokines [Supplementary Files 4D(e),4E(e),4F(e)].

### Establishment of a Monoclonal Antibody (mAb) Against Rainbow Trout CD8α

Although an anti-trout CD8α mAb had already been established by our group (51), a new mAb clone named 7α8c with a different immunoglobulin isotype was established enabling multi-color immunostaining with mAbs against other molecules. MAb 7α8c was established as previously described (52, 113) with slight modifications as follows: Rats were immunized twice, with a 3-4 week interval, into the tail base with Normal Rat Kidney cells expressing trout CD8α (51) emulsified in Complete Freund's Adjuvant (Sigma). Hybridomas were cloned twice by limiting dilution, and one of the resulting clones, designated as 7α8c, was selected for further experiments. Supernatants were stored as 50% glycerol stocks at −20°C. For validation of mAb specificity, HEK293T cells expressing trout CD8α-HA (established by Takizawa et al., unpublished) were tested for the reactivity with mAb 7α8c by flow cytometry using FACSCanto II (BD Biosciences). As a positive expression control, an anti-HA mAb was applied. Anti-rat IgG Alexa Fluor 488 (Thermo Fisher scientific) and anti-Mouse IgG, IgM (H+L) Alexa Fluor 488 Secondary Antibody were used as secondary conjugates, respectively. To further prove the specificity of mAb 7α8c, mAb^+^ and mAb^−^ lymphocyte subpopulations were flow sorted from trout intestine as described in the next paragraph. Total RNA was extracted from the subpopulations using NucleoSpin RNA kit (Macherey-Nagel). As was done in all the experiments for which we used the NucleoSpin RNA kit, total RNA was treated with rDNase I on the NucleoSpin column according to the manufacturer's instruction in order to degrade genomic DNA prior to downstream steps. Total RNA was then subjected to semi-quantitative one step RT-PCR analysis with primers for β*-actin* and *CD8*α (Supplementary File 9B) as described previously (51). The cycle numbers used for β*-actin* and *CD8*α were 25 cycles and 37 cycles, respectively.

### Isolation of Rainbow Trout Lymphocyte Subpopulations

Leukocytes from trout thymus, gill, head kidney (HK), spleen, and intestine were isolated as described previously (51). Briefly, the cell suspensions were layered onto an isotonic Percoll (GE Healthcare) gradient (ρ = 1.075 g/ml) and centrifuged at 650 × g for 40 min. After centrifugation, cells lying at the interface were collected and washed twice with cold mixed medium (MM): Iscove's DMEM/Ham's F12 (Gibco) at a ratio of 1:1, supplemented with 10% fetal bovine serum (FBS) and 100 U penicillin/ml and 100 μg streptomycin/ml. If leukocyte isolation was followed by flow sorting, leukocytes from 4 to 8 clonal individuals were pooled to get sufficient amounts of lymphocytes (Supplementary File 3). The cell suspensions were kept on ice until further preparation.

To isolate CD8α^+^ and CD8α^−^ lymphocytes, leukocytes from thymus, gill, HK, spleen, and intestine were stained with anti-CD8α (clone 13.2D; rat IgG2a isotype) (51) and anti-rat IgG Alexa Fluor 488. To isolate CD8 single positive [SP], CD4SP, double positive [DP] and double negative [DN] lymphocytes, leukocytes from thymus, spleen, and intestine were stained with anti-CD4-1 (rat IgG2a isotype) (52), anti-CD4-2 (rat IgG2b isotype) (52) and anti-CD8α [clone 7α8c; rat IgG1 isotype (Supplementary File 3A)] mAbs. Stained cells were detected with anti-rat IgG2a-PE (eBioscience), anti-rat IgG2b-PE (eBioscience), and anti-rat IgG1-FITC conjugates (BD Bioscience), respectively. To isolate CD8SP, CD4SP, IgMSP, and triple negative [TN] lymphocytes, splenocytes were stained with anti-CD4-1, anti-CD4-2, and anti-CD8α mAbs as described above in addition to anti-IgM (mouse IgG1 isotype) mAb (114). Stained cells were detected with anti-rat IgG2a-eFluor 660 (eBioscience), anti-rat IgG2b-eFluor 660 (eBioscience), anti-rat IgG1-FITC, and anti-mouse IgG1-Brilliant Violet 421™ conjugates (Biolegend), respectively.

Cell suspensions were incubated on ice with mAbs and corresponding secondary conjugates for 30 min and then washed twice with MM after each respective staining steps. For negative controls, conjugate controls were prepared as described above. Doublets were excluded by FSC-A/FSC-H gating, and dead cells were excluded by DAPI (4', 6-diamidino-2-phenylindole)- or PI- staining. Secondary antibodies were tested before the experiments to exclude possible cross-reactions between isotype/species specific conjugates. Stained cells were sorted using a BD FACSAria™ Fusion flow cytometer (BD Biosciences). For flow sorting, only “lymphocyte gate” cells (FSC^low^/SSC^low^; aka “morphological lymphocytes”) where considered; it should be noted that the so isolated cell population, besides lymphocytes, is expected to also contain thrombocytes and small monocytes. For sorting of IgMSP and TN lymphocytes, only the CD4^−^CD8^−^ DN population was considered. After all conditions were adjusted by setting the thresholds for conjugate controls and the compensation parameters, test sortings were performed for each of the populations to confirm their purity. The purity of each sorted lymphocyte subpopulation was at least 99.2%. Data on flow cytometry were analyzed using FlowJo V10 software (Tree Star). Sorted-lymphocyte subpopulations were cultivated overnight with MM (containing 20% FBS) at 15°C under a 2.5% CO_2_ atmosphere, prior to the next experimental steps.

### Stimulation of Trout Leukocytes With Recombinant Cytokines

#### Stimulation of Trout Leukocyte Subpopulations and Western Blot Analysis

CD8^+^ and CD8^−^ lymphocytes were isolated from trout thymus, gill, HK, spleen, and intestine while CD8SP, CD4SP, DP, and DN lymphocytes were isolated from trout thymus, spleen and intestine, as described above. In each experiment, equal numbers of lymphocyte subpopulations (1–3 × 10^5^/incubation, depending on the yields achieved after flow sorting) were resuspended in 400 μl of supernatants containing recombinant cytokines (1:1 dilution in MM) from transfected HEK293T or in 400 μl of purified recombinant proteins diluted in MM (5, 25, and 125 nM) and incubated for 15 min at 15°C. As negative controls, lymphocyte subpopulations were incubated with supernatants from mock-transfected HEK293T cells (1:1 dilution in MM) or protein storage buffer (see paragraph *Purification of recombinant cytokines with anti-FLAG agarose*) diluted with MM. After incubation, lymphocytes were pelleted at 1,500 × g for 5 min at 4°C, and lysed with 17 μl of NP40 Cell Lysis Buffer supplied with Protease Inhibitor Cocktail and Halt Phosphatase Inhibitor Cocktail (Thermo Fisher Scientific). Then the samples were subjected to Western blot analysis for detection of phosphorylated STAT5 similar to as described above except for using 8% poly-acrylamide gels.

#### Stimulation of Trout Leukocytes and RT-qPCR Analysis

Trout total splenocytes were freshly isolated as described above. Samples were used per individual fish in “total splenocytes” stimulation experiments, but pooled from multiple clonal fish prior to flow sorting into CD8SP, CD4SP, IgMSP, and TN lymphocytes. Total splenocytes were incubated for 4 or 12 h with 400 μl of supernatants containing recombinant cytokines (1:1 dilution in MM) from transfected HEK293T cells or with 400 μl of purified recombinant proteins diluted in MM (5, 25, and 125 nM). The subpopulations were incubated for 12 h with 400 μl of purified recombinant proteins diluted in MM (0.2 nM and 5 nM). For negative controls, total splenocytes and their subpopulations were mock-treated as described above. In each experiment, equal numbers of total splenocytes (2 × 10^5^) or lymphocyte subpopulations (1–3 × 10^5^) were incubated. After incubation at 15°C, cells were pelleted as described above, followed by treatment with 350 ul of Lysis Buffer RA1 (supplied by NucleoSpin RNA kit) containing 1/100 2-mercaptoethanol.

Total RNA was extracted from the incubated cells using NucleoSpin RNA kit and aliquots corresponding to 0.5–1.6 × 10^5^ cells were reverse transcribed into cDNA using SensiFAST™ cDNA Synthesis Kit (Bioline) according to the manufacturer's instruction. The resulting cDNA was diluted 1:6 with distilled water. Five μl of the diluted cDNA was used for qPCR detection of expression of *IFN*γ, *Perforin, IL-4/13A, IL-4/13B1, IL-4/13B2*, and *EF1A* using primer sets Trout_IFNγ (1/2)_qPCR, Trout_PFN1_qPCR, Trout_IL-4/13A_qPCR, Trout_IL-4/13B1_qPCR, Trout_IL-4/13B2_qPCR, and Trout_EF1A_qPCR (Supplementary File 9B). qPCRs were performed using SensiFAST™ SYBR Lo-ROX Kit (Bioline) with Stratagene Mx3000P and MxPro software version 4.10 (Agilent Technologies). The comparative quantitation mode was chosen with default settings except for the amplification conditions (120 s at 95°C, followed by 40 cycles of 5 s at 95°C, 11 s at 60°C and 15 s at 72°C). As in the other RT-qPCR experiments performed in this study, each primer set was designed with at least one primer across an intron or corresponding to different exons to avoid amplification from genomic DNA, and after each RT-qPCR amplification, melting curve analysis of PCR products was performed in order to exclude the presence of amplified genomic DNA, unspecific products, or primer dimer synthesis. All PCR reactions were done in duplicate (independent mixing of same cDNA and same amplification mix into two wells of the same PCR plate) for technical replication, and non-template controls were included. Based on the two replicates, the Ct values were calculated automatically by MxPro software, and used for analysis. The Ct-variabilities between technical duplicates were lower than 0.5 in most cases. However, especially at higher Ct values when the number of target gene transcripts was low, these variabilities were sometimes above 0.5 (indicated in Italic in Supplementary File 6). The relative expression levels in the lymphocytes were normalized to the expression levels of *EF1A* using the equation 2^−ΔΔ*Ct*^ (115). For the graphs in Figure 10 and Supplementary File 6A, the *EF1A*-normalized gene expression levels were normalized to those of the relevant control samples (4 or 12 h) in the same experiment which were set as 1, while such normalization was applied for the graphs in Figure 11 and Supplementary File 6B by setting each TN control sample as the standard. This method was chosen because it focuses on the fold-differences induced within cell samples, and in the case of Figure 11 also allows an instantaneous impression of the differences in expression levels between cell populations.

### Stimulation of Rainbow Trout With Recombinant Cytokines

#### Stimulation of Rainbow Trout and RT-qPCR Analysis

Forty rainbow trout juveniles of ~10 grams, ten per cytokine, were injected intraperitoneally (i.p.) with 50 μl recombinant cytokine (1 μM) in protein storage buffer or with buffer control; the cytokines were purified IL-2, IL-15-RLI, or IL-15La-RLI that had been produced in insect cells. At 6 h and 12 h after protein injection the spleen and head kidney were harvested from five fish per treatment and stored in RNAlater (Ambion) for RNA extraction. Total RNA was extracted using NucleoSpin RNA kit (Macherey-Nagel) and 1 μg RNA was subjected to cDNA synthesis using QuantiTect Reverse Transcription Kit (Qiagen). Transcripts of *IFN*γ, *Perforin, IL-4/13A, IL-4/13B1, IL-4/13B2*, and *EF1A* were analyzed by qPCR using primer sets as listed in the manuscript table (Supplementary File 9B) and PowerUp SYBR Green Master mix (Applied Biosystems) with CFX96 PCR system (Bio-Rad) and CFX Manager™ Software (Bio-Rad). The comparative quantitation mode was chosen with default settings except for the amplification conditions (120 s at 50°C, 120 s at 95°C followed by 40 cycles of 3 s at 95°C and 30 s at 60°C). All reactions were done in duplicate and the mean Ct values were used for analysis. The relative expression levels in spleen and head kidney were normalized to the expression levels of *EF1A*. Quality controls were similar to as described in the paragraph “*Stimulation of trout leukocytes and RT-qPCR analysis*.” For the statistical evaluation of the qPCR data, one-way ANOVA was applied to the log-adjusted *EF1A*-normalized gene expression levels using IBM SPSS Statistics 25 Software.

#### Next Generation Sequencing (NGS) and Quantification of Immune Gene Reads

Total RNA samples derived from spleen from the four groups of five fish of the above described 6 h treatment panel were used for NGS analysis. RNA quality from each fish was confirmed using the RNA 6000 Pico chips by Agilent 2100 Bioanalyzer. Total RNA samples were pooled per IL-2, IL-15-RLI, IL-15La-RLI, or buffer control treatment (*n* = 5 per pool) and polyadenylated mRNA was purified from 10 μg pooled total RNA using the Dynabeads mRNA DIRECT Micro kit (Thermo Fisher Scientific) and the addition ERCC ExFold RNA Spike-In mix 1 was used as the external RNA control. Whole cDNA barcoded libraries were then generated using the Ion Total RNA-Seq Kit v2 (Thermo Fisher Scientific) and quantified by the KAPA Library Quantification Kit (Roche) on Ion Torrent platform (Thermo Fisher Scientific). The final equimolar pools were used for sequencing by Ion S5XL sequencing system with the Ion 540 OT2 kit (Thermo Fischer Scientific) for the generation of the necessary datasets, resulting in 20 million 80 bps single end reads, on average, per sample. The percentages of high quality reads in the samples generated after stimulation with IL-15-RLI (85%), IL15-La-RLI (83%), IL-2 (84%) and buffer control (83%) were determined by Geneious RNA assembler (https://www.geneious.com) based on comparison with rainbow trout genome sequence (GenBank assembly accession: GCA_002163495.1). The number of reads specific for the open reading frames of immune marker genes of interest were determined by the method of minimum 99.9% mapping quality without gaps using Geneious 11.1.5 (https://www.geneious.com), and the results are in Supplementary File 8.

#### Statistics

For the statistical evaluation of the qPCR data, the paired samples *t*-test was applied to the log-adjusted *EF1A*-normalized gene expression levels calculated for biological quadruplicates using IBM SPSS Statistics 25 Software (54). Calculated *p*-values < 0.05 between cytokine-treated and mock-treated samples of the same cell populations were considered to be significant.

#### Sample-Size Estimation and Replicates

For important observations concerning a quantitative issue, we required confirmation by at least three independent biological tests, often involving multiple approaches (not necessarily each approach was performed in triplicate). No outlier data were excluded. For RT-qPCR and flow cytometry experiments all data obtained are presented. Most of the Western blot results, including repeats, are shown, and after the experimental conditions had been established no Western blots were excluded for analysis/interpretation although some are not presented for editorial reasons such as the quality of the image. The replicate data are shown in the Supplementary Files and are mentioned at appropriate locations in the main text.

## Data Availability Statement

The datasets generated for this study can be found in online repositories. The names of the repository/repositories and accession number(s) can be found in the article/Supplementary Material.

## Ethics Statement

The animal study was reviewed and approved by relevant laws and committees in the different countries. The animals used were fish. In Germany, the experiments complied with the Guidelines of the European Union Council (2010/63/EU) for the use of laboratory animals, and were in agreement with the German Animal Protection Act (Deutsches Tierschutzgesetz). In Scotland the fish handing and experimental protocols complied with the Guidelines of the European Union Council (2010/63/EU) for the use of laboratory animals, and were carried out under UK Home Office project license PPL 60/4013, approved by the ethics committee at the University of Aberdeen. In Japan the experiments complied with the Law for the Humane Treatment and Management of Animals.

## Author Contributions

TY: conceptualization, formal analysis, funding acquisition, validation, investigation, visualization, methodology, and writing—review and editing. CC: conceptualization, formal analysis, validation, investigation, and writing—review and editing. AKa: conceptualization, validation, investigation, visualization, methodology, and writing—review and editing. MK: investigation and methodology. FP: conceptualization, formal analysis, validation, and investigation. EW: investigation. TW: formal analysis, validation, investigation, visualization, and writing—review and editing. CS: supervision, validation, project administration, and writing—review and editing. AKi: investigation. MF: resources and investigation. KH: supervision, project administration, and writing—review and editing. UF: conceptualization, supervision, funding acquisition, validation, investigation, visualization, methodology, project administration, and writing—review and editing. JD: conceptualization, supervision, funding acquisition, validation, investigation, visualization, methodology, writing—original draft, and writing—review and editing. All authors contributed to the article and approved the submitted version.

## Conflict of Interest

The authors declare that the research was conducted in the absence of any commercial or financial relationships that could be construed as a potential conflict of interest.
