# Supplementary material for: Ancient Cytokine Interleukin 15-Like (IL-15L) Induces a Type 2 Immune Response
Source: Front Immunol. 2020 Oct 29;11:549319. doi: 10.3389/fimmu.2020.549319 (PMC7658486; doi:10.3389/fimmu.2020.549319)
Supplement: Supplementary file 1 [file Data_Sheet_1.pdf]

Supplementary file 1

Additional information on *IL-15L* nucleotide and deduced amino acid sequences

Table of Contents **Page**

1A: Trout *IL-15La* cDNA sequence 2

1B: Trout *IL-15Lb* cDNA sequence 4

1C: Expression levels of rainbow trout and Atlantic salmon *IL-15La* and *IL-15Lb* transcripts in various tissues and cell cultures 6

1D: Rainbow trout *IL-15La* and *IL-15Lb* probably derived from a gene duplication in the salmonid lineage 10

References used in this file 11

Supplementary file 1A. Trout *IL-15La* cDNA sequence.

Figure (b) shows an assembly of two sequences obtained from spleen cDNA. One sequence contained the entire coding sequence and was amplified by primers Trout_IL-15La_CDS_F and Trout_IL-15La_CDS_R. The other sequence was obtained by nested 5'-RACE, using SMARTer RACE cDNA amplification kit (Clontech) and two PCR reactions; for the first PCR reaction the gene-specific primer Trout_IL-15La_5'RACE_R was used in conjunction with the Clontech primer UPM, which was followed by diluting the mixture and a second PCR reaction using the gene-specific primer Trout-IL-15La_5'RACE.nes_R in conjunction with the Clontech primer NUP. The 5'-RACE result is shown in (a), with the arrow pointing at the analyzed band. Other than database comparisons (see main text Table 1), no efforts were dedicated to potentially finding alternative transcripts from the same gene. Primer sequences, or their complementary sequences, are underlined in (b), with the primer direction indicated besides the primer name. The intron positions were determined by comparison with the genomic sequence of GenBank accession MSJN01002393, and are indicated by downward triangles. ATG motifs in the 5'UTR region are shaded yellow. Amino acids are indicated below the first nucleotides of codons.

(a) *5'RACE PCR*

Left lane is size marker in bp units. Right lane is nested 5'-RACE result for trout IL-15La.

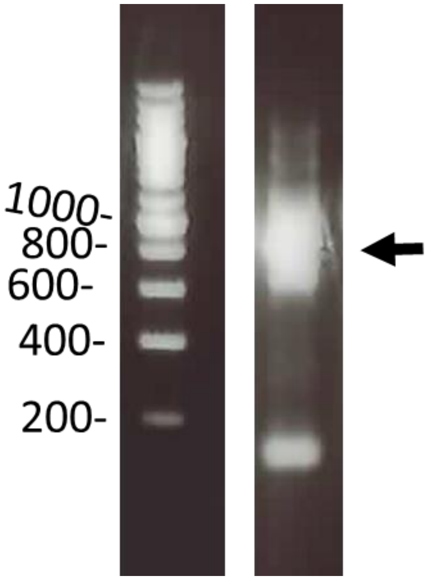

(Supplementary file 1A)

(b) *Assembled sequence of trout IL-15La ORF and 5'-RACE product.*

```
        10        20        30        40        50        60        70        80        90
GTGCACTAATAAAGAACTGCGGTTGTGTTCGGGTTGGACTCTCTAGGCTACACTCTTGAGTGTAAATGAGCATTTGAAATGTCAGGATTT

       100       110       120       130       140       150       160       170       180
TGTTGCAGTACAAATATTATAGATTTTAGCTACACAATTTGAACTTCAACTGTGGATTTTAAAGTATCCTATGTCAAGTCATCTGCTTCT

       190       200       210       220       230       240       250       260       270
TGACGGAATTACTTTTGTTTTCACTTTGTACCTACATACGGTGCAAACTTCAGTAGTCTTTCAAGCTGATTTACCATGTCCGGTGAGAAG

       280       290       300       310       320       330       340       350       360
CCTAGACAAGAAAAAGGAGGCAGATGCTGACGGCTTGCCTCATCTCTTCTGCTGCGCTGCTTTCCAGGACACACAAGAGAGATGCTGCAT

       370       380       390       400       410       420       430       440       450
AACAAGTCACCAGGAAGATAGAAGACAGAGTGTTTTACCCCTCATCCAAAAGTGGGGAGCCTACTGGCTAAGAAGGAGGCACATAGCTGG

       460       470       480       490       500       510       520       530       540
AGTCTTGCCTCATCTCTACTGCTTCTTCCCAGGACACACAGGAGATGTAGTGTCACTAAGAAGGCTGCAATTGACATGGACAATATGCTG

       550       560       570       580       590       600       610       620       630
AATCCCAAAACAATGGAGGATTTTAAAATAGTTATCTTAATTTTAAGCTACTTCTCTACTGATCTAAAGCTCATGTGAAAGAGTGGGCCC
                                                   Trout_IL-15La_CDS_F >
       640       650       660       670       680       690       700       710       720
ATGCCATGCTGAGGAGACAGAGGACTGACACTCTTCTAGCCCTTTTGCTGTGGTTTCTCTTCTTCATCGCCATGACAATGAAACAGGCAT
     M  L  R  R  Q  R  T  D  T  L  L  A  L  L  L  W  F  L  F  F  I  A  M  T  M  K  Q  A  Y

       730       740       750       760       770       780       790       800       810
ATGGAAAATCCATGTGCAGTAAAGAACTTCCCGGAATTGTGCGAAAATGCATTGAGGAAGTTCACAAGATGGAATCATTTGATTGCAGAC
  G  K  S  M  C  S  K  E  L  P  G  I  V  R  K  C  I  E  E  V  H  K  M  E  S  F  D  C  R  L

       820       830       840       850       860       870       880       890       900
TGTACACCCCAACTTTGGCTGATTATCAGAAGTGCCCCACGTCCACACTCATATGCTTTGAAAAAGAAGTGAATGTCCTGGTGTTAGAAT
  Y  T  P  T  L  A  D  Y  Q  K  C  P  T  S  T  L  I  C  F  E  K  E  V  N  V  L  V  L  E  S
< Trout_IL-15La_5'RACE.nes_R
       910       920       930       940       950       960       970       980       990
CTGGGAATAAGTCCTCACCCATATACAAGCCAAAACTATCCATCCGGCTGAAGTCCTTGATCAAACAGAAAGAAGGTGCCAACTGTCCAG
  G  N  K  S  S  P  I  Y  K  P  K  L  S  I  R  L  K  S  L  I  K  Q  K  E  G  A  N  C  P  D
                                              < Trout_IL-15La_5'RACE_R
      1000      1010      1020      1030      1040      1050      1060      1070      1080
ACTGTGAGGCCCACAGAGAAAGGGCAGCAAAGGATTTCTTAACAACATTGCAAACAATTCTGGAGTGGATGAACGATCAGGGGTGTCGGA
  C  E  A  H  R  E  R  A  A  K  D  F  L  T  T  L  Q  T  I  L  E  W  M  N  D  Q  G  C  R  K

      1090      1100      1110      1120
AGCCATCCAGCCATTGAGATGGACACATCCATAGACAGGGACA
  P  S  S  H  *
                    < Trout_IL-15La_CDS_R
```

Supplementary file 1B. Trout *IL-15Lb* cDNA sequence.

Figure (b) shows an assembly of two sequences obtained from spleen cDNA. One sequence contained the entire coding sequence and was amplified by primers Trout_IL-15Lb_CDS_F and Trout_IL-15Lb_CDS_R. The other sequence was obtained by nested 5'-RACE, using SMARTer RACE cDNA amplification kit (Clontech) and two PCR reactions; for the first PCR reaction the gene-specific primer Trout_IL-15Lb_5'RACE_R was used in conjunction with the Clontech primer UPM, which was followed by diluting the mixture and a second PCR reaction using the gene-specific primer Trout-IL-15Lb_5'RACE.nes_R in conjunction with the Clontech primer NUP. The 5'-RACE result is shown in (a), with the arrow pointing at the analyzed band. Other than comparisons with database reports of *IL-15Lb* transcripts in related species (see main text Table 1), no efforts were dedicated to potentially finding alternative transcripts from the same gene. Primer sequences, or their complementary sequences, are underlined in (b), with the primer direction indicated besides the primer name. The intron positions were determined by comparison with the genomic sequence of GenBank accession MSJN01001275, and are indicated by downward triangles. ATG motifs in the 5'UTR region are shaded yellow. Amino acids are indicated below the first nucleotides of codons.

(a) *5'RACE PCR.*
Left lane is size marker in bp units. Right lane is the nested 5'-RACE result for trout IL-15Lb.

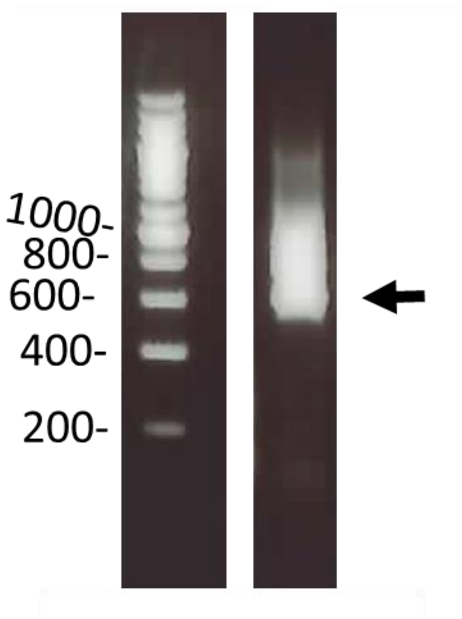

(Supplementary file 1A)

(b) *Assembled sequence of trout IL-15Lb ORF and 5’-RACE product.*

```
        10        20        30        40        50        60        70        80        90
GTTTTCACTTTGTACCTACCTACGATGCAAACTTCAAAAGTCGCTCAAGCTGATTTAACCATGCCAGGTGAGGAGTCTAGCCAAGAAAAA

       100       110       120       130       140       150       160       170       180
GGAGGCAGATGCTGAAGACGTGCCTAATTTCTTCTGCTGCTTTCCAGGACACACAAGAAAGAGGCTGCATAGACACACAGGGGATATACT

       190       200       210       220       230       240       250       260       270
GTGTCACAAAGAAGGCTGCAGATGACATGGACAAGAGGCTGAACCCCAAAACAATGGAGGATTTTAAACTAATTCTCCTAATTTAAGCTA
                Trout_IL-15Lb_CDS_F >
       280       290       300       310       320       330       340       350       360
TTTTCCCACTGATCTGAAGCTCCTGCGACAAAGTGGGCCCATGCCATGTTGAGGAGACAGAGAACTGGCTCTCTTCTGAACGCTTTGCTG
                                             M  L  R  R  Q  R  T  G  S  L  L  N  A  L  L

       370       380       390       400       410       420       430       440       450
TGGTTTCTCTTCTTCATTGCCATGACAATGAAACAGGCTTATGGACAATCCATTAGCAGTTCAGAAATTCACCAAATTGTGAAAACATTT
W  F  L  F  F  I  A  M  T  M  K  Q  A  Y  G  Q  S  I  S  S  S  E  I  H  Q  I  V  K  T  F

       460       470       480       490       500       510       520       530       540
ATTAAGGAGGAAGTTCACAGGATGGAATCATTTGATTGCAGACTGTACACCCCAACTTTAGCTGATTATAAGAAATGTCCCAGGTCCACA
I  K  E  E  V  H  R  M  E  S  F  D  C  R  L  Y  T  P  T  L  A  D  Y  K  K  C  P  R  S  T

       550       560       570       580       590       600       610       620       630
CTCACATGCTTTACGACAGAAGTAAAAGTCCTGATGTTAGAATATGGGAAACGTTCTTCATCCTTACACCAGAAAAGACTCACCAAACGA
L  T  C  F  T  T  E  V  K  V  L  M  L  E  Y  G  K  R  S  S  S  L  H  Q  K  R  L  T  K  R
< Trout_IL-15Lb_5’RACE.nes_R
       640       650       660       670       680       690       700       710       720
CTGACTAAATTGATGTCCTTGATAAAACAGAAGGATGGTGCCAACTGTCCACACTGTGAGGTCCACAGAGAACAGGCAGCAAATGATTTC
L  T  K  L  M  S  L  I  K  Q  K  D  G  A  N  C  P  H  C  E  V  H  R  E  Q  A  A  N  D  F

       730       740       750       760       770       780       790       800       810
TTAACAACATTACTAGGGATTCTGGAGTGGATGAACAATCAGGGGTCTCAGTTGCCAGACAGCCACTGAGATGTACACACCCATAGCCAG
L  T  T  L  L  G  I  L  E  W  M  N  N  Q  G  S  Q  L  P  D  S  H  *
 < Trout_IL-15Lb_5’RACE_R
       820       830       840
GGACATCCAAGGACTCACATAAGAGCAGA
        < Trout_IL15-Lb_CDS_R
```

Supplementary file 1C. Expression levels of rainbow trout and Atlantic salmon *IL-15La* and *IL-15Lb* transcripts in various tissues and cell cultures.

This figure contains a table with the relative *IL-15La* and *IL-15Lb* expression levels per investigated trout tissue and cell culture sample as determined by RT-qPCR, which are the data used for the graphs shown in main text Fig. 2 (a). Furthermore, the results are shown for analyses of *IL-15La* and *IL-15Lb* expression levels in various tissues of rainbow trout and Atlantic salmon as determined by semi-quantitative RT-PCR (b) and by counting matches in single read archive (SRA) datasets (c). Despite variation between fish individuals, rather consistent findings were that: (i) *IL-15La* expression was more ubiquitously distributed than *IL-15Lb* expression; (ii) *IL-15Lb* expression was relatively high in gill; (iii) both *IL-15La* and *IL-15Lb* expression tended to be relatively low in head kidney. These findings agree with the RT-qPCR results shown in main text Fig. 2.

Detailed legends for figures (a), (b) and (c) are:

(a) *Expression of IL-15La and IL-15Lb in rainbow trout tissues, cell lines and primary head kidney (HK) macrophages analyzed by RT-qPCR.* The expression levels of *IL-15La* and *IL-15Lb* were determined by RT-qPCR in seventeen tissues from six rainbow trout individuals, and four flasks of cells from each cell line and primary HK macrophages. The transcript levels wer calculated using a serial dilution of references that contained equal molar amounts of probes for each gene and was normalized against the expression level of *EF1A*. The expression level relative to *EF1A* (x1,000,000) for each sample is shown. MS, missing samples. 0, non-detectable.

(b) *IL-15La and IL-15Lb expression analyzed for tissues of two trout individuals by semi-quantitative gene-specific RT-PCR.* The expected sizes of the amplified bands for *IL-15La*, *IL-15Lb* and *EF1A* were 204, 281, and 377 bp, respectively. In the photographs of the agarose gels after electrophoresis, the left lanes contain the size marker with sizes indicated in bp. The results for head kidney and gill are highlighted.

(c) *IL-15La and IL-15Lb expression analyzed for tissues of three rainbow trout and two Atlantic salmon individuals by counting of gene-specific matches per 10^8 database reads.* Read numbers per 10^8 reads of *IL-15La* and *IL-15Lb* were determined by similarity searches against tissue-specific single read archive (SRA) datasets available at NCBI. For rainbow trout, the SRA datasets of Bioprojects PRJEB4450, PRJNA389609 and PRJNA380337 were investigated. For Atlantic salmon, the SRA datasets of Bioprojects PRJNA260929 and PRJNA72713 were investigated. Bioproject PRJEB4450 concerns tissues of a homozygous clonal 1-year-old female rainbow trout sampled 3 weeks after spawning (1). Bioproject PRJNA389609 concerns thirteen different tissues that were collected from a single immature (2-year old, 250 g) male homozygous rainbow trout of the Swanson clonal line, while the oocyte and pineal gland samples were pooled from multiple trout individuals (2). Bioproject PRJNA380337 concerns rainbow trout tissues for which detailed information has not been provided (authored by the Norwegian University of Life Sciences). Bioproject PRJNA260929 concerns tissues of a 1-year-old single homozygous female Atlantic salmon from the AquaGen aquaculture strain named "Sally" (3) (Dr. Unni Grimholt, personal communication). Bioproject PRJNA72713 concerns Atlantic salmon tissues for which detailed information has not been provided

(authored by the University of Victoria). The results for the tissues head kidney and gill are highlighted.

(Supplementary file 1C)

(a) Expression of IL-15La and IL-15Lb in rainbow trout tissues, cell lines and primary head kidney (HK) macrophages analyzed by RT-qPCR.

	IL-15La expression in tissues						*IL-15Lb* expression in tissues					
Tissue	Fish 1	Fish 2	Fish 3	Fish 4	Fish 5	Fish 6	Fish 1	Fish 2	Fish 3	Fish 4	Fish 5	Fish 6
Liver	15.58	22.71	3.23	14.64	17.92	98.43	0.02	0.00	0.00	0.00	0.00	0.00
HK	18.43	22.51	11.95	13.73	61.20	176.17	0.08	0.02	0.01	0.01	0.06	0.10
Tail fins	13.77	0.00	20.09	16.67	17.95	404.76	0.03	0.07	0.06	0.05	0.19	1.01
Scales	70.71	77.13	30.60	22.08	54.63	243.20	1.97	0.26	0.08	0.00	0.19	0.97
Adipose tissue	80.13	211.43	62.91	45.21	156.69	88.15	0.20	0.00	0.00	0.04	0.10	0.10
Caudal kidney	44.11	101.27	28.82	57.27	301.52	157.47	0.11	0.05	0.04	0.14	0.96	0.73
Thymus	209.99	89.01	190.16	51.48	46.24	272.29	10.08	1.66	2.91	3.61	1.94	65.97
Spleen	171.00	180.80	117.32	93.29	42.99	531.03	6.42	6.11	3.47	3.97	1.34	21.67
Gills	164.79	266.21	125.06	51.64	40.24	897.17	3.04	10.20	1.89	1.77	1.23	20.22
Blood	137.91	404.88	312.29	MS	232.37	334.68	0.00	0.00	0.00	MS	0.00	0.00
Heart	167.75	461.90	126.40	61.89	499.30	649.81	0.00	0.00	0.00	0.00	0.03	0.00
Skin	101.27	197.64	190.40	90.82	199.47	1238.09	0.30	0.00	0.06	0.04	0.04	0.20
Adipose fin	225.00	588.31	440.72	341.51	316.84	451.03	3.13	6.57	1.55	MS	2.04	8.17
Intestine	89.79	156.01	140.85	81.69	809.54	1099.55	1.72	0.65	1.22	0.73	8.96	2.23
Brain	268.10	951.69	318.92	352.61	184.20	1063.26	0.54	0.48	0.00	0.00	0.00	0.33
Muscle	592.69	432.56	753.16	221.94	365.48	2090.16	0.00	0.00	1.11	0.00	0.00	0.00
Gonad	649.09	1032.90	255.66	389.44	1334.84	1647.01	2.54	0.75	0.63	0.52	2.18	2.47

	IL-15La expression in cell lines and HK macrophages						*IL-15Lb* expression in cell lines and HK macrophages					
Cells	Flask 1	Flask 2	Flask 3	Flask 4			Flask 1	Flask 2	Flask 3	Flask 4		
RTL	1291.99	1600.00	1678.29	2468.27			0.00	0.00	0.00	0.00		
RTG-2	128.65	53.12	55.98	41.36			0.00	0.00	0.00	0.00		
RTGill	5.96	5.08	4.80	6.01			0.00	0.00	0.00	0.00		
RTS-11	5.24	0.00	7.85	0.00			0.00	0.00	0.00	0.00		
HK Macrophages	16.11	5.05	6.87	9.56			0.00	0.00	0.00	0.00		

(b) IL-15La and IL-15Lb expression analyzed for tissues of two trout individuals by semi-quantitative gene-specific RT-PCR.

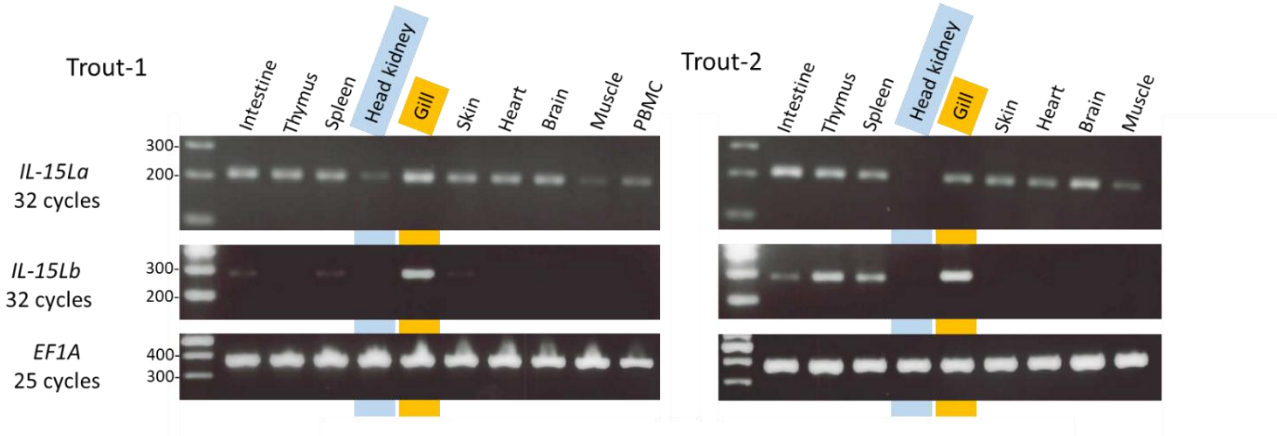

(Supplementary file 1C)

(c) IL-15La and IL-15Lb expression analyzed for tissues of three rainbow trout and two Atlantic salmon individuals by counting of gene-specific matches per 10^8 database reads.

Matching reads / 10^8 (100,000,000) total reads

Trout (Bioproject PRJEB4450)

	Intestine	Spleen	Kidney	Head Kidney	Gill (branchiae)	Skin	Heart	Brain	White muscle	Red muscle	Pineal gland	Hypophysis	Liver	Stomach	Bone	Testis	Ovary	Eye	Pyloric caeca	Nose	Pancreas	Fat	Oocyte
IL-15La	35	14	3	2	15	7	2	3	3	7	N.A.	0	23	8	5	N.A.	49	N.A.	N.A.	N.A.	N.A.	N.A.	N.A.
IL-15Lb	114	41	0	0	115	27	0	0	0	0	N.A.	0	3	9	25	N.A.	13	N.A.	N.A.	N.A.	N.A.	N.A.	N.A.

Trout (Bioproject PRJNA389609)

	Intestine	Spleen	Kidney	Head Kidney	Gill	Skin	Heart	Brain	White muscle	Red muscle	Pineal gland	Hypophysis	Liver	Stomach	Bone	Testis	Ovary	Eye	Pyloric caeca	Nose	Pancreas	Fat	Oocyte
IL-15La	38	17	22	17	55	9	N.A.	6	12	14	5	N.A.	13	7	N.A.	14	N.A.	N.A.	N.A.	N.A.	N.A.	13	3
IL-15Lb	74	14	2	1	35	3	N.A.	0	2	0	0	N.A.	0	3	N.A.	2	N.A.	N.A.	N.A.	N.A.	N.A.	0	0

Trout (Bioproject PRJNA380337)

	Intestine	Spleen	Kidney	Head Kidney	Gill	Skin	Heart	Brain	Muscle	Pineal gland	Hypophysis	Liver (average of 1,2,3)	Stomach	Bone	Testis	Ovary	Eye	Pyloric caeca	Nose	Pancreas	Fat	Oocyte
IL-15La	14	0	3	3	46	39	6	15	6	N.A.	N.A.	6	N.A.	N.A.	N.A.	N.A.	34	24	N.A.	N.A.	N.A.	N.A.
IL-15Lb	6	22	0	0	93	0	0	0	0	N.A.	N.A.	0	N.A.	N.A.	N.A.	N.A.	6	9	N.A.	N.A.	N.A.	N.A.

Salmon (Bioproject PRJNA260929)

	Intestine	Spleen	Kidney	Head Kidney	Gill	Skin	Heart	Brain	Muscle	Pineal gland	Hypophysis	Liver	Stomach	Bone	Testis	Ovary	Eye	Pyloric caeca	Nose	Pancreas	Fat	Oocyte
IL-15La	216	47	N.A.	N.A.	14	113	73	0	0	N.A.	N.A.	147	N.A.	N.A.	N.A.	N.A.	N.A.	95	N.A.	213	N.A.	N.A.
IL-15Lb	0	0	N.A.	N.A.	100	301	0	0	0	N.A.	N.A.	0	N.A.	N.A.	N.A.	N.A.	N.A.	354	N.A.	0	N.A.	N.A.

Salmon (Bioproject PRJNA72713)

	Intestine	Spleen	Kidney	Head Kidney	Gill	Skin	Heart	Brain	Muscle	Pineal gland	Hypophysis	Liver	Stomach	Bone	Testis	Ovary	Eye	Pyloric caeca	Nose	Pancreas	Fat	Oocyte
IL-15La	50	56	52	17	N.A.	12	65	10	49	N.A.	N.A.	167	N.A.	N.A.	23	7	0	192	67	N.A.	N.A.	N.A.
IL-15Lb	0	0	0	0	N.A.	0	3	0	0	N.A.	N.A.	0	N.A.	N.A.	8	0	0	0	30	N.A.	N.A.	N.A.

Supplementary file 1D. Rainbow trout *IL-15La* and *IL-15Lb* probably derived from a gene duplication in the salmonid lineage.

The sequence divergence in the IL-2/15/15L family of cytokines is enormous and attempts to use phylogenetic tree software analysis to reliably determine the precise relationships between IL-15L and related cytokines were inconclusive [not shown and see (4)]. However, phylogenetic tree software does give reliable results when addressing more recent events in evolution, like for example the *IL-15L a-b* duplication observed in trout and salmon. Therefore, we only analyzed a selected group of molecules.

A tree (a) was created using all the IL-15L amino acid sequences shown main text Fig. 3 with as outgroup human IL-15, all aligned as in main text Fig. 3, and only using the parts aligned with mature human IL-15. The analysis was conducted in MEGA6 (5) by UPGMA method (6). The percentage of replicate trees in which the associated taxa clustered together in the bootstrap test (10000 replicates) are shown next to the branches. The tree is drawn to scale, with branch lengths in the same units as those of the evolutionary distances used to infer the phylogenetic tree. The evolutionary distances were computed using the Poisson correction method and are in the units of the number of amino acid substitutions per site. All ambiguous positions were removed for each sequence pair. The important results are highlighted in blue and show that the salmonid *IL-15L a-b* duplication occurred after the ancestors of salmonids and zebrafish separated, and before the separation of the species lineages leading to rainbow trout and Atlantic salmon. This *IL-15L* duplication also included the neighboring genes (main text Fig. 1) and presumably was part of the whole genome duplication early in the salmonid lineage (1, 3, 7).

(a) *Phylogenetic tree.*

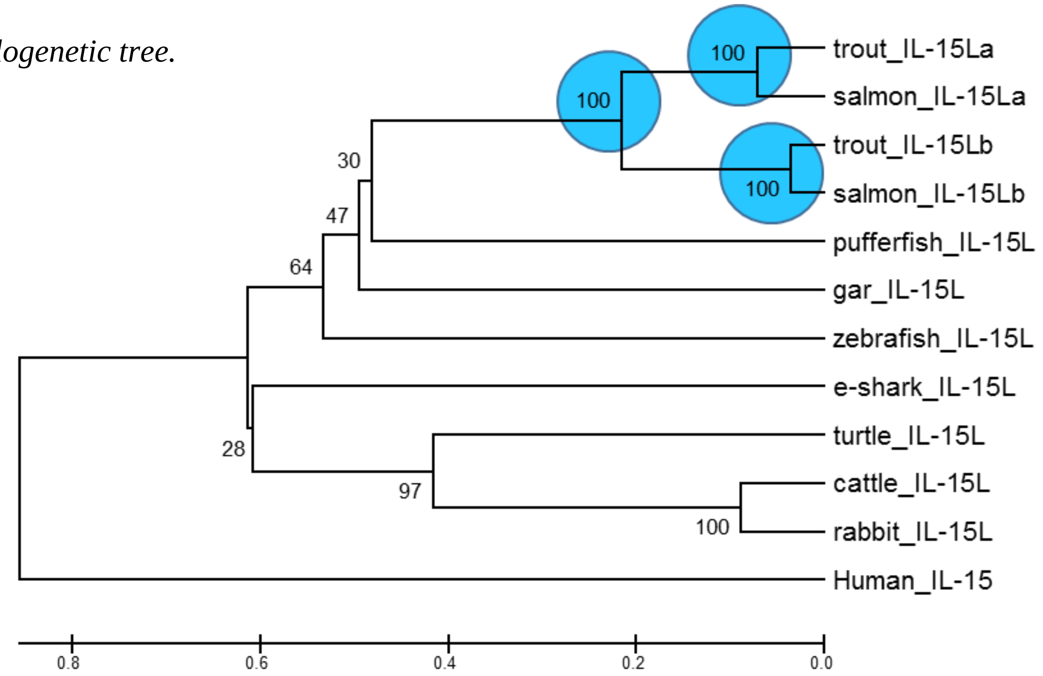

References used in this file

1. Berthelot C, Brunet F, Chalopin D, Juanchich A, Bernard M, Noël B, et al. The rainbow trout genome provides novel insights into evolution after whole-genome duplication in vertebrates. Nat Commun. (2014) 5:3657. doi: 10.1038/ncomms4657

2. Salem M, Paneru B, Al-Tobasei R, Abdouni F, Thorgaard GH, Rexroad CE, et al. Transcriptome assembly, gene annotation and tissue gene expression atlas of the rainbow trout. PLoS ONE. (2015) 10:e0121778. doi: 10.1371/journal.pone.0121778

3. Lien S, Koop BF, Sandve SR, Miller JR, Kent MP, Nome T, et al. The Atlantic salmon genome provides insights into rediploidization. Nature. (2016) 533:200–205. doi: 10.1038/nature17164

4. Dijkstra JM, Takizawa F, Fischer U, Friedrich M, Soto-Lampe V, Lefèvre C, et al. Identification of a gene for an ancient cytokine, interleukin 15-like, in mammals; interleukins 2 and 15 co-evolved with this third family member, all sharing binding motifs for IL-15Ra. Immunogenetics. (2014) 66:93–103. doi: 10.1007/s00251-013-0747-0

5. Tamura K, Stecher G, Peterson D, Filipski A, Kumar S. MEGA6: molecular Q21 evolutionary genetics analysis version 6.0. Mol Biol Evol. (2013) 30:2725–9. doi: 10.1093/molbev/mst197

6. Sneath PHA, Sokal RR. Numerical Taxonomy. San Francisco, CA: Freeman (1973).

7. Allendorf FW, Thorgaard GH. Tetraploidy the evolution of salmonid fishes. In: Turner BJ. Editor. The Evolutionary Genetics of Fishes. Boston, MA: Springer (1984) p. 1–53
