# Supplementary material for: Ancient Cytokine Interleukin 15-Like (IL-15L) Induces a Type 2 Immune Response
Source: Front Immunol. 2020 Oct 29;11:549319. doi: 10.3389/fimmu.2020.549319 (PMC7658486; doi:10.3389/fimmu.2020.549319)
Supplement: Supplementary file 2 [file Data_Sheet_2.pdf]

Supplementary file 2

Expression vectors used in this study

Table of Contents **Page**

Legend to Supplementary files 2A, 2B, 2C and 2D	3
2A: Expression vectors for (modified) bovine cytokines and receptor chains	4
- pRcCMV2-*Bos-IL-2-FLAG*	4
- pRcCMV2-*Bos-IL-15-FLAG*	4
- pRcCMV2-*Bos-IL-15L-FLAG*	5
- pRcCMV2-*Bos-IL-15L-(non-tagged)*	5
- pRcCMV2-*Bos-IL-15Lhyb-FLAG*	6
- pcDNA3.1-*IL-2-Lead-RLI-bov.IL-15Lhyb*	6
- pcDNA3.1-*Bos-IL-15Rα-Myc-His*	7
- pcDNA3.1-*Bos-solIL-15Rα-Myc-His*	8
- pcDNA3.1-*Bos-IL-2Rα-Myc-His*	8
- pcDNA3.1-*Bos-solIL-2Rα-Myc-His*	9
2B: Expression vector for a genetic fusion of human IL-15Rα and IL-15	10
- pcDNA3.1-*IL-2-Lead-RLI*	10
2C: Expression vectors for (modified) trout cytokines and IL-15Rα	11
- pcDNA3.1-*trout-IL-2-FLAG*	11
- pcDNA3.1-*trout-IL-2-(non-tagged)*	11
- pcDNA3.1-*trout-IL-15-FLAG*	12
- pcDNA3.1-*trout-IL-15-(non-tagged)*	12
- pcDNA3.1-*trout-IL-15La-FLAG*	13
- pcDNA3.1-*trout-IL-15La-(non-tagged)*	13
- pcDNA3.1-*trout-IL-15Lb-FLAG*	14
- pcDNA3.1-*trout-IL-15Lb-(non-tagged)*	14
- pcDNA3.1-*IL-2-Lead-RLI-trout-IL-15La*	15
- pcDNA3.1-*trout-IL-15Rα-Myc*	15
- pcDNA3.1-*trout-solIL-15Rα-Myc*	16

2D: Vectors for expressing (modified) trout cytokines and trout IL-15Rα in insect cells using a baculovirus system. 17

- pFBD-P10Uhis-ieGFP-*trout-IL-2-FLAG* 17
- pFBD-P10Uhis-ieGFP-*trout-IL-15-FLAG* 17
- pFBD-P10Uhis-ieGFP-*trout-IL-15La-FLAG* 18
- pFBD-P10Uhis-ieGFP-*trout-solIL-15Rα-Myc* 18
- pFBD-P10Uhis-ieGFP-*trout-IL-15-RLI* 19
- pFBD-P10Uhis-ieGFP-*trout-IL-15La-RLI* 19

References used in this file 21

Legend to Supplementary files 2A, 2B, 2C and 2D.

This Supplementary file shows the sequence fragments containing bovine (2A), human (2B), and trout (2C) *IL-2*, *IL-15*, *IL-15L*, *IL-2Rα* and/or *IL-15Rα* sequences, or modifications thereof, which were cloned into DNA plasmid expression vectors behind a CMV immediate early promoter or into a baculovirus transfer vector behind a p10 promoter (2D). The texts for the individual constructs explain wat the vectors encode, and how the encoded protein is named in the paper. The pink font sequence fragments indicate the restriction sites used for cloning. The DNA plasmid expression vector used for cloning was either pRc/CMV2 (Invitrogen) or pcDNA^{TM}3.1/myc-His B (Invitrogen). The baculovirus transfer vector used for cloning was pFBD-P10Uhis-ieGFP (a derivate of pFastBac-Dual [Invitrogen], see main text Materials and methods section). The cloning strategies varied and were somewhat arbitrarily because experiments were done at different time points and constructs were made in different laboratories.

Several of the expression constructs with bovine *IL-2*, *IL-15L*, *IL-15Rα* and *IL-2Rα* were already described in detail in references (1) and (2), but for convenience are shown here again.

Because the information on the construction method is not so relevant, the below only briefly mentions whether the construction involved an amplification from cDNA or a purchase of a synthetic gene sequence (Invitrogen) and describes the GenBank accession number of a matching sequence. When relevant, additional information is given.

Green font nucleotide sequence: By choice of primer sequences, or synthetic gene ordering, an ACC motif was added directly upstream of the start codon to assure efficient translation.

Red font amino acid sequence: By choice of primer sequences, or synthetic gene ordering, FLAG tag sequences were added.

Orange font amino acid sequence: By choice of primer sequences, or as part of the commercial vector, Myc tag sequences were added.

Purple font amino acid sequence: As part of the commercial vector, poly-His tag sequences were added.

Other colors are explained in the individual texts.

Supplementary file 2A.
Expression vectors for (modified) bovine cytokines and receptor chains.

pRcCMV2-*Bos-IL-2-FLAG*

This expression vector encodes bovine FLAG-tagged IL-2 (named IL-2).
Cloned from cDNA. The *IL-2* part of the sequence is identical with GenBank accession EU276068.
[for cloning details see (1)]

AAGCTTACCATGTACAAGATACAACTCTTGTCTTGCATTGCACTAACTCTTGCACTCGT
TGCAAACGGTGCACCTACTTCAAGCTCTACGGGGAACACAATGAAAGAAGTGAAGTCAT
TGCTGCTGGATTTACAGTTGCTTTTGGAGAAAGTTAAAAATCCTGAGAACCTCAAGCTC
TCCAGGATGCATACATTTGACTTTTACGTGCCCAAGGTTAACGCTACAGAATTGAAACA
TCTTAAGTGTTTACTAGAAGAACTCAAACTTCTAGAGGAAGTGCTAAATTTAGCTCCAA
GCAAAAACCTGAACCCCAGAGAGATCAAGGATTCAATGGACAATATCAAGAGAATCGTT
TTGGAACTACAGGGATCTGAAACAAGATTCACATGTGAATATGATGATGCAACAGTAAA
CGCTGTAGAATTTCTGAACAAATGGATTACCTTTTGTCAAAGCATCTACTCAACAATGA
CTGATTATAAGGATGACGACGATAAGTAAGGGCCC

MYKIQLLSCIALTLALVANGAPTSSSTGNTMKEVKSLLLDLQLLLEKVKNPENLKLSRM
HTFDFYVPKVNATELKHLKCLLEELKLLEEVLNLAPSKNLNPREIKDSMDNIKRIVLEL
QGSETRFTCEYDDATVNAVEFLNKWITFCQSIYSTMTDYKDDDDK

pRcCMV2-*Bos-IL-15-FLAG*

This expression vector encodes bovine FLAG-tagged IL-15 (named IL-15).
The sequence was commercially ordered (Invitrogen). The leader sequence is replaced with that of bovine IL-2 (shaded blue), and the codons were optimized for expression in human cells. The encoded IL-15 part is identical to GenBank accession ELR44697.

AAGCTTACCATGTACAAGATCCAGCTGCTGAGCTGTATCGCCCTGACCCTGGCCCTGGT
GGCCAACGGCAATTGGCAGTACGTGATCAACGACCTGAAAACCATCGAGCACCTGATCC
AGAGCATCCACATGGACGCCACCCTGTACACCGAGAGCGACGCCCACCCCAACTGCAAA
GTGACCGCCATGCAGTGCTTTCTGCTGGAACTGAGAGTGATCCTGCACGAGAGCAAGAA
CGCCACCATCTACGAGATCATCGAGAATCTGACCATGCTGGCCAACAGCAACCTGAGCA
GCATCGAGAACAAGACCGAGCTGGGCTGCAAAGAGTGCGAGGAACTGGAAGAGAAGTCC
ATCAAAGAGTTTCTGAAGTCCTTCGTGCACATCGTGCAGATGTTCATCAACACCAGCGA
CTACAAGGACGACGACGACAAGTGATCTAGAGGGCCC

MYKIQLLSCIALTLALVANGNWQYVINDLKTIEHLIQSIHMDATLYTESDAHPNCKVTA
MQCFLLELRVILHESKNATIYEIIENLTMLANSNLSSIENKTELGCKECEELEEKSIKE
FLKSFVHIVQMFINTSDYKDDDDK

pRcCMV2-*Bos-IL-15L-FLAG*

This expression vector encodes bovine FLAG-tagged IL-15L (named IL-15L).
Cloned from cDNA. The *IL-15L* part of the sequence is identical with GenBank accession NM_001301213.
[for cloning details see (1)]

```
AAGCTTACCATGTGGCTTCTCTGGACCACCCTCCTGCTGGTGCTGCCCTTGGGAGGCCT
AGGACCACTCCTCTGCCCAAGGGAGCCTTTCTACTTCCTCATTGCCATCACGAAGATGC
TGGAAAACAAAAATGATGGCAGTCTGTACACCCCAGATAATCTATTGGTGTGTCCTGCT
GAGACTCTCCGATGCTTCCGGCTGGAGTTGTCTGTGATCGGGTTTGAGGAGGGCCCATC
CGTGGGGATCGTTGTGTTCCGCCTACAGCGCCTACTGGATGCCCTGGGGTCCCAGCTGT
GGGTGATTGATCAGGGCCCTTGTCCACCCTGCGAAGGACACCCTCAGAGACCAGTCCCT
CTTTTTCTGGCCAAACTCTTGGAGTTATTACAGGGGACTTGTGCTCGGGACCTGCCCTC
AGCAGATTACAAGGATGACGACGATAAGTAATCTAGA
```

```
MWLLWTTLLLVLPLGGLGPLLCPREPFYFLIAITKMLENKNDGSLYTPDNLLVCPAETL
RCFRLELSVIGFEEGPSVGIVVFRLQRLLDALGSQLWVIDQGPCPPCEGHPQRPVPLFL
AKLLELLQGTCARDLPSADYKDDDDK
```

pRcCMV2-*Bos-IL-15L-(non-tagged)*

This expression vector encodes bovine non-tagged IL-15L [named IL-15L(N)].
Cloned from cDNA. The *IL-15L* sequence is identical with GenBank accession NM_001301213.
[for cloning details see (1)]

```
AAGCTTACCATGTGGCTTCTCTGGACCACCCTCCTGCTGGTGCTGCCCTTGGGAGGCCT
AGGACCACTCCTCTGCCCAAGGGAGCCTTTCTACTTCCTCATTGCCATCACGAAGATGC
TGGAAAACAAAAATGATGGCAGTCTGTACACCCCAGATAATCTATTGGTGTGTCCTGCT
GAGACTCTCCGATGCTTCCGGCTGGAGTTGTCTGTGATCGGGTTTGAGGAGGGCCCATC
CGTGGGGATCGTTGTGTTCCGCCTACAGCGCCTACTGGATGCCCTGGGGTCCCAGCTGT
GGGTGATTGATCAGGGCCCTTGTCCACCCTGCGAAGGACACCCTCAGAGACCAGTCCCT
CTTTTTCTGGCCAAACTCTTGGAGTTATTACAGGGGACTTGTGCTCGGGACCTGCCCTC
AGCATAATCTAGA
```

```
MWLLWTTLLLVLPLGGLGPLLCPREPFYFLIAITKMLENKNDGSLYTPDNLLVCPAETL
RCFRLELSVIGFEEGPSVGIVVFRLQRLLDALGSQLWVIDQGPCPPCEGHPQRPVPLFL
AKLLELLQGTCARDLPSA
```

pRcCMV2-*Bos-IL-15Lhyb-FLAG*

This expression vector encodes bovine FLAG-tagged IL-15L in which a few motifs have been replaced for that of other IL-2/15/15L family members (named IL-15Lhyb).
The part encoding the mature protein was commercially ordered (Invitrogen) with the codons optimized for expression in insect cells, and fused to sequences encoding authentic bovine IL-15L leader sequence and a C-terminal FLAG-tag by using appropriate primers and overlap PCR. The rationale behind the substitutions (shaded yellow) was that *in vitro* handling of bovine IL-15L protein revealed instability (not shown), while two of the modified motifs were different in mammals from IL-2/15/15L family consensus, and we thought that adding of the trout IL-15La glycosylation motif might aid stability. Unfortunately, an improved stability, or a function, was not found for this IL-15Lhyb molecule.

```
AAGCTTACCATGTGGCTTCTCTGGACCACCCTCCTGCTGGTGCTGCCCTTGGGAGGCCT
AGGACCTTTGTTGTGCCCCCGTGAACCCTTCTACTTCCTGCGCAAGATCACCAAGATGC
TCGAGAACAAGAACGACGGTTCCCTGTACACCCCCGACAACCTGCTCGTGTGCCCCGCT
GAAACCCTGCGCTGCTTCCGTCTGGAACTGTCCGTGATCGGTTTCGAGGAAGGCAACAA
GTCCTCCGTGGGTATCGTGGTGTTCAGGCTGCAGCGTCTGCTGGACGCTCTGGGTTCTC
AGCTGTGGGTCACCGACCAGGGACCTTGCCCTCCTTGCGAGGGTCACCCTCAACGTCCC
GTGCCTCTGTTCCTGGCTAAGCTGCTCGAGCTGCTGCAGGGAACCTGCGCTCGTGACCT
GCCCTCCGCTGATTACAAGGATGACGACGATAAGTAATCTAGA
```

```
MWLLWTTLLLVLPLGGLGPLLCPREPFYFLRKITKMLENKNDGSLYTPDNLLVCPAETL
RCFRLELSVIGFEEGNKSSVGIVVFRLQRLLDALGSQLWVTDQGPCPPCEGHPQRPVPL
FLAKLLELLQGTCARDLPSADYKDDDDK
```

pcDNA3.1-*IL-2-Lead-RLI-bov.IL-15Lhyb*

This expression vector is based on the RLI construct described by Mortier *et al.* 2006 (3), and encodes a genetic fusion of a human IL-2 leader, a FLAG-tag, a large part of the human IL-15Rα ectodomain, a glycine-serine linker (shaded blue), and the above described bovine IL-15Lhyb sequence (with the modified motifs shaded yellow) (named bov.IL-15Lhyb-h-RLI).
IL-15Rα does not interact with the signaling receptor IL-2Rβ·IL-2Rγ, and is thought to fixate IL-15 into the proper conformation for binding IL-2Rβ·IL-2Rγ (4); given the observed cross-species activity (main text Fig. 5), we speculated that RLI-versions of bovine and trout IL-15L would have similar stability and signaling advantages as observed for all-human RLI (3, 5, 6).
The linker plus IL-15Lhyb part were commercially ordered (Invitrogen) with the codons optimized for expression in insect cells, and fused to the N-terminal coding part by using plasmid pcDNA3.1-*IL-2-Lead-RLI*, appropriate primers and overlap PCR.

GGTACCACCATGTACAGGATGCAACTCCTGTCTTGCATTGCACTAAGTCTTGCACTTGT
CACAAACAGTGACTACAAGGATGACGATGACAAGATAGAAGGTAGGATCACATGCCCTC
CCCCCATGTCCGTGGAACACGCAGACATCTGGGTCAAGAGCTACAGCTTGTACTCCAGG
GAGCGGTACATTTGTAACTCTGGTTTCAAGCGTAAAGCCGGCACGTCCAGCCTGACGGA
GTGCGTGTTGAACAAGGCCACGAATGTCGCCCACTGGACAACCCCCAGTCTCAAATGCA
TTAGAGACCCTGCCCTGGTTCACCAAAGGCCAGCGCCACCCTCTGGAGGCTCCGGTGGA
GGTGGGAGTGGCGGTGGATCCGGTGGCGGAGGCAGCCCTTTGTTGTGCCCCCGTGAACC
CTTCTACTTCCTGCGCAAGATCACCAAGATGCTCGAGAACAAGAACGACGGTTCCCTGT
ACACCCCCGACAACCTGCTCGTGTGCCCCGCTGAAACCCTGCGCTGCTTCCGTCTGGAA
CTGTCCGTGATCGGTTTCGAGGAAGGCAACAAGTCCTCCGTGGGTATCGTGGTGTTCAG
GCTGCAGCGTCTGCTGGACGCTCTGGGTTCTCAGCTGTGGGTCACCGACCAGGGACCTT
GCCCTCCTTGCGAGGGTCACCCTCAACGTCCCGTGCCTCTGTTCCTGGCTAAGCTGCTC
GAGCTGCTGCAGGGAACCTGCGCTCGTGACCTGCCCTCCGCTTAATCTAGA

MYRMQLLSCIALSLALVTNSDYKDDDDKIEGRITCPPPMSVEHADIWVKSYSLYSRERY
ICNSGFKRKAGTSSLTECVLNKATNVAHWTTPSLKCIRDPALVHQRPAPPSGGSGGGGS
GGGSGGGGSPLLCPREPFYFLRKITKMLENKNDGSLYTPDNLLVCPAETLRCFRLELSV
IGFEEGNKSSVGIVVFRLQRLLDALGSQLWVTDQGPCPPCEGHPQRPVPLFLAKLLELL
QGTCARDLPSA

pcDNA3.1-*Bos-IL-15Rα-Myc-His*

This expression vector encodes full-length bovine Myc-tagged IL-15Rα (named IL-15Rα).
Cloned from cDNA. The *IL-15Rα* part of the sequence is identical with GenBank accession XM_015465884.
[for cloning details see (1)]

AAGCTTACCATGTCCGGGCGGCTCCGGGGCCGCGGGGCCGGCGCCCTCCCCGCGCTGGG
GCTGCTGCTGCTACTACTGCTGCTCGGATCTTCGGCCACGCCGGGCATCACCTGCCCGA
CTCCCACATCCGTGGAGCATGCAGACATCCAGGTCAAGAGTTACAGCATCAACTCCAGG
GAGCGGTATGTTTGTAATTCTGGCTTCAAGCGTAAAGCTGGGACTTCCAGCTTGACCCA
GTGTGTGTTTAACGAGACCGCGAAAGTCGCCCACTGGACCACTCCCAACCTCAAGTGCA
TCAGAGACCCCTCCCTGAGTCACCAAAGGCCACCCTCCACAGCAGCGCCTACAGGGTTG
ACCCCAGAGCCAGAGAGCCCCACCCCCTCCGGAAAAGAGCCAGATCTTACTTCCAAGTC
AGACACCAAAGTGGCCACAAGGCCAGCTACTGGACCAGGCTCCAGGCTGCCATCCACAG
CTCCTCCTGTGGGAACCACAGGGGTAGTCAGTAAGGAGACCACCTACGTCCCAGCTCAG
ACAGCAGCCAAGGCTCCGGAACACACATACCCGGCCTTGCAGGACACGCCCGGTGCATA
TCAGAACAATCCCAGAGTTGTGACCGCCGTCTCAACTGTCACTGTGCTCTTTGTAGTAT
GCCTGGTGTTTCTTCTGGGACGTTGCCTGTGGTCAAGGCGAGCCCACCAGACACCCGGT
GTTGAGATGGAGAGCATGGAGAGTGTGCCAATGACCACGGGGGCCGATGCCAGAGGGGA
GGACACAGAAATCCACCCGCATGGCCTAGGAGGCTCCGGGGACGCTGAGGCCAGCAGCG
GCCGCAGTGAAGGCCCAGCTCTTCCCCAGTCAGAGAGGACCTCGAGTCTAGAGGGCCCG
CGGTTCGAACAAAAACTCATCTCAGAAGAGGATCTGAATATGCATACCGGTCATCATCA
CCATCACCATTGA

MSGRLRGRGAGALPALGLLLLLLLLGSSATPGITCPTPTSVEHADIQVKSYSINSRERY
VCNSGFKRKAGTSSLTQCVFNETAKVAHWTTPNLKCIRDPSLSHQRPPSTAAPTGLTPE
PESPTPSGKEPDLTSKSDTKVATRPATGPGSRLPSTAPPVGTTGVVSKETTYVPAQTAA
KAPEHTYPALQDTPGAYQNNPRVVTAVSTVTVLFVVCLVFLLGRCLWSRRAHQTPGVEM
ESMESVPMTTGADARGEDTEIHPHGLGGSGDAEASSGRSEGPALPQSERTSSLEGPRFE
QKLISEEDLNMHTGHHHHHH

pcDNA3.1-*Bos-solIL-15Rα-Myc-His*

This expression vector encodes bovine soluble Myc-tagged IL-15Rα (named soluble IL-15Rα or sIL-15Rα).
Cloned from cDNA. The *IL-15Rα* part of the sequence is identical with GenBank accession XM_015465884.
[for cloning details see (1)]

AAGCTTACCATGTCCGGGCGGCTCCGGGGCCGCGGGGCCGGCGCCCTCCCCGCGCTGGG
GCTGCTGCTGCTACTACTGCTGCTCGGATCTTCGGCCACGCCGGGCATCACCTGCCCGA
CTCCCACATCCGTGGAGCATGCAGACATCCAGGTCAAGAGTTACAGCATCAACTCCAGG
GAGCGGTATGTTTGTAATTCTGGCTTCAAGCGTAAAGCTGGGACTTCCAGCTTGACCCA
GTGTGTGTTTAACGAGACCGCGAAAGTCGCCCACTGGACCACTCCCAACCTCAAGTGCA
TCAGAGACCCCTCCCTGAGTCACCAAAGGCCACCCTCCACAGCAGCGCCTACAGGGTTG
ACCCCAGAGCCAGAGAGCCCCACCCCCTCCGGAAAAGAGCCAGATCTTACTTCCAAGTC
AGACACCAAAGTGGCCACAAGGCCAGCTACTGGACCAGGCTCCAGGCTGCCATCCACAG
CTCCTCCTGTGGGAACCACAGGGGTAGTCAGTAAGGAGACCACCTACGTCCCAGCTCAG
ACAGCAGCCAAGGCTCCGGAACACACATACCCGGCCTTGCAGGACACGCCCGGTGCATA
TCAGAACAATCCCAGCTCGAGTCTAGAGGGCCCGCGGTTCGAACAAAAACTCATCTCAG
AAGAGGATCTGAATATGCATACCGGTCATCATCACCATCACCATTGA

MSGRLRGRGAGALPALGLLLLLLLLGSSATPGITCPTPTSVEHADIQVKSYSINSRERY
VCNSGFKRKAGTSSLTQCVFNETAKVAHWTTPNLKCIRDPSLSHQRPPSTAAPTGLTPE
PESPTPSGKEPDLTSKSDTKVATRPATGPGSRLPSTAPPVGTTGVVSKETTYVPAQTAA
KAPEHTYPALQDTPGAYQNNPSSSLEGPRFEQKLISEEDLNMHTGHHHHHH

pcDNA3.1-*Bos-IL-2Rα-Myc-His*

This expression vector encodes bovine full-length Myc-tagged IL-2Rα (named IL-2Rα).
Cloned from cDNA. Except for a single nucleotide replacement (shaded yellow), the *IL-2Rα* part of the sequence is identical with GenBank accession BC133546.
[for cloning details see (1); through primer choice a silent mutation was introduced at the single yellow-shaded C position in order to delete a gene-internal restriction site]

AAGCTTACCATGGAGCCCAGCTTGCTGATGTGGAGGTTCTTCGTATTCATCGTGGTACC
TGGCTGCGTGACAGAGGCTTGTCATGATGACCCTCCGAGTCTCAGAAACGCCATGTTCA
AGGTCTTCAGGTACGAGGTGGGCACCATGATAAACTGCGACTGCAAGACAGGCTTCCGC
AGAGTGTCGGCCGTCATGCGCTGCGTGGGGGACTCCAGCCACTCTGCCTGGGAAAACAG
ATGCTTCTGCAACAGCACCTCCCCTGCTAAGAACCCAGTAAAACAAGTCACTCCTGCAC
CCGAAGAACAGAGGGAGAAAAAACCCACAGATGCGCAGAACCAAACGCAGCCTCCGGAG
GAAGCTGACCTTCCAGGTCACTGTGAGGAACCGCCACCATGGGAACACGAACGTGAACC
TTTAAAGAGAGTCTACCATTTCACGCTGGGGCAGACGGTTCATTACCAGTGCGCCCAGG
GATTCAGGGCCCTACAGACCAGTCCTGCTGAAAGCACCTGCATGATGATCAACGGGGAG
CTGAGGTGGACCAGGCCCAGGCTCAAGTGCATACGTGAAGGGGAGCACGGTCAGGCTTC
AGATGACGCAGAGCCTCAGGAGAGCACGGAAGCTCCCCCTGGGAGTGGAACTTTCTTAC
CAACCAGGATGGCAGGGACCACAGATTTCCAGAAGCCCACAGATGAGATTGCAACGCTG
GATACGTTCATATTTACCACTGAGTACCAGATTGCAGTGGCCGGCTGCACCCTCCTGCT
CGCCAGCATCCTCCTCCTGAGCTGCCTCACCTGGCAGCGGAAATGGAAGAAGAACAGAA
GGACAATCTCGAGTCTAGAGGGCCCGCGGTTCGAACAAAAACTCATCTCAGAAGAGGAT
CTGAATATGCATACCGGTCATCATCACCATCACCATTGA

MEPSLLMWRFFVFIVVPGCVTEACHDDPPSLRNAMFKVFRYEVGTMINCDCKTGFRRVS
AVMRCVGDSSHSAWENRCFCNSTSPAKNPVKQVTPAPEEQREKKPTDAQNQTQPPEEAD
LPGHCEEPPPWEHEREPLKRVYHFTLGQTVHYQCAQGFRALQTSPAESTCMMINGELRW
TRPRLKCIREGEHGQASDDAEPQESTEAPPGSGTFLPTRMAGTTDFQKPTDEIATLDTF
IFTTEYQIAVAGCTLLLASILLLSCLTWQRKWKKNRRTISSLEGPRFEQKLISEEDLNM
HTGHHHHHH

pcDNA3.1-*Bos-solIL-2Rα-Myc-His*

This expression vector encodes bovine soluble Myc-tagged IL-2Rα (named soluble IL-2Rα or sIL-2Rα).
Cloned from cDNA. Except for a single nucleotide replacement (shaded yellow), the *IL-2Rα* part of the sequence is identical with GenBank accession BC133546.
[for cloning details see (1); through primer choice a silent mutation was introduced at the single yellow-shaded C position in order to delete a gene-internal restriction site]

AAGCTTACCATGGAGCCCAGCTTGCTGATGTGGAGGTTCTTCGTATTCATCGTGGTACC
TGGCTGCGTGACAGAGGCTTGTCATGATGACCCTCCGAGTCTCAGAAACGCCATGTTCA
AGGTCTTCAGGTACGAGGTGGGCACCATGATAAACTGCGACTGCAAGACAGGCTTCCGC
AGAGTGTCGGCCGTCATGCGCTGCGTGGGGGACTCCAGCCACTCTGCCTGGGAAAACAG
ATGCTTCTGCAACAGCACCTCCCCTGCTAAGAACCCAGTAAAACAAGTCACTCCTGCAC
CCGAAGAACAGAGGGAGAAAAAACCCACAGATGCGCAGAACCAAACGCAGCCTCCGGAG
GAAGCTGACCTTCCAGGTCACTGTGAGGAACCGCCACCATGGGAACACGAACGTGAACC
TTTAAAGAGAGTCTACCATTTCACGCTGGGGCAGACGGTTCATTACCAGTGCGCCCAGG
GATTCAGGGCCCTACAGACCAGTCCTGCTGAAAGCACCTGCATGATGATCAACGGGGAG
CTGAGGTGGACCAGGCCCAGGCTCAAGTGCATACGTGAAGGGGAGCACGGTCAGGCTTC
AGATGACGCAGAGCCTCAGGAGAGCACGGAAGCTCCCCCTGGGAGTGGAACTTTCTTAC
CAACCAGGATGGCAGGGACCACAGATTTCCAGAAGCCCACAGATGACTCGAGTCTAGAG

GGCCCGCGGTTCGAACAAAAACTCATCTCAGAAGAGGATCTGAATATGCATACCGGTCA
TCATCACCATCACCATTGA

MEPSLLMWRFFVFIVVPGCVTEACHDDPPSLRNAMFKVFRYEVGTMINCDCKTGFRRVS
AVMRCVGDSSHSAWENRCFCNSTSPAKNPVKQVTPAPEEQREKKPTDAQNQTQPPEEAD
LPGHCEEPPPWEHEREPLKRVYHFTLGQTVHYQCAQGFRALQTSPAESTCMMINGELRW
TRPRLKCIREGEHGQASDDAEPQESTEAPPGSGTFLPTRMAGTTDFQKPTDEIATLDTF
IFTTEYQIAVAGCTLLLASILLLSCLTWQRKWKKNRRTISSLEGPRFEQKLISEEDLNM
HTGHHHHHH

Supplementary file 2B.
Expression vector for a genetic fusion of human IL-15Rα and IL-15.

pcDNA3.1-*IL-2-Lead-RLI*

Except for the leader sequence which is of human IL-2, the encoded sequence is identical to the RLI molecule described by Mortier *et al.* 2006 (5) which is a genetic fusion subsequently encoding a FLAG-tag, a large part of the human IL-15Rα ectodomain, a glycine-serine linker (shaded blue), and human IL-15 (named RLI).
Amplified from commercial cDNA and using appropriate primers and overlap PCR.

GGTACCACCATGTACAGGATGCAACTCCTGTCTTGCATTGCACTAAGTCTTGCACTTGT
CACAAACAGTGACTACAAGGATGACGATGACAAGATAGAAGGTAGGATCACATGCCCTC
CCCCCATGTCCGTGGAACACGCAGACATCTGGGTCAAGAGCTACAGCTTGTACTCCAGG
GAGCGGTACATTTGTAACTCTGGTTTCAAGCGTAAAGCCGGCACGTCCAGCCTGACGGA
GTGCGTGTTGAACAAGGCCACGAATGTCGCCCACTGGACAACCCCCAGTCTCAAATGCA
TTAGAGACCCTGCCCTGGTTCACCAAAGGCCAGCGCCACCCTCTGGAGGCTCCGGTGGA
GGTGGGAGTGGCGGTGGATCCGGTGGCGGAGGCAGCCTGCAGAACTGGGTGAATGTAAT
AAGTGATTTGAAAAAAATTGAAGATCTTATTCAATCTATGCATATTGATGCTACTTTAT
ATACGGAAAGTGATGTTCACCCCAGTTGCAAAGTAACAGCAATGAAGTGCTTTCTCTTG
GAGTTACAAGTTATTTCACTTGAGTCCGGAGATGCAAGTATTCATGATACAGTAGAAAA
TCTGATCATCCTAGCAAACAACAGTTTGTCTTCTAATGGGAATGTAACAGAATCTGGAT
GCAAAGAATGTGAGGAACTGGAGGAAAAAAATATTAAAGAATTTTTGCAGAGTTTTGTA
CATATTGTCCAAATGTTCATCAACACTTCTTAATCTAGA

MYRMQLLSCIALSLALVTNSDYKDDDDKIEGRITCPPPMSVEHADIWVKSYSLYSRERY
ICNSGFKRKAGTSSLTECVLNKATNVAHWTTPSLKCIRDPALVHQRPAPPSGGSGGGGS
GGGSGGGGSLQNWVNVISDLKKIEDLIQSMHIDATLYTESDVHPSCKVTAMKCFLLELQ
VISLESGDASIHDTVENLIILANNSLSSNGNVTESGCKECEELEEKNIKEFLQSFVHIV
QMFINTS

Supplementary file 2C.
Expression vectors for (modified) trout cytokines and IL-15Rα.

pcDNA3.1-*trout-IL-2-FLAG*

This expression vector encodes rainbow trout FLAG-tagged IL-2 (named IL-2).
Cloned from cDNA. The *IL-2* part of the sequence is identical with GenBank accession FJ571512.

AAGCTTACCATGGACCGTCGTTACAGGATTTCCTTTTTGACGCTTTTTCTCACCGGTTG
TCTACAAGGAAACCCAATTCCCAGACTCCTAGCTGGAATCGATTATCTAGAAGAAAATA
TTACTTGTCCAGATTCAGTCTTCTATACACCAACTGATGTAGAGGATAGTTGCATTGTT
GCAGCATTGGCCTGTTCCATTAAGGAACTGGACACTGTGAAAGTAGAATGCCTCGATAA
CGCGGTCCATCTGGAAAGTATGCAACACCACATCAGCATGACTGCCACGGACCTACAAA
AGACGATTGATAAGGAGAACAGCACAACGGACACTTCAGAATGCATCTGTGAAGACAAG
CGGTTGGAAAAGTCTTTCAAGGACTTCATTCAGAACATAAGACATTTAACTCAAGCTCA
TGCTGCAAAGCGTCTAAGTTCAGATTACAAGGATGACGACGATAAGTAACTCGAG

MDRRYRISFLTLFLTGCLQGNPIPRLLAGIDYLEENITCPDSVFYTPTDVEDSCIVAAL
ACSIKELDTVKVECLDNAVHLESMQHHISMTATDLQKTIDKENSTTDTSECICEDKRLE
KSFKDFIQNIRHLTQAHAAKRLSSDYKDDDDK

pcDNA3.1-*trout-IL-2-(non-tagged)*

This expression vector encodes rainbow trout non-tagged IL-2 [named IL-2(N)].
Cloned from cDNA. The *IL-2* part of the sequence is identical with GenBank accession FJ571512.

AAGCTTACCATGGACCGTCGTTACAGGATTTCCTTTTTGACGCTTTTTCTCACCGGTTG
TCTACAAGGAAACCCAATTCCCAGACTCCTAGCTGGAATCGATTATCTAGAAGAAAATA
TTACTTGTCCAGATTCAGTCTTCTATACACCAACTGATGTAGAGGATAGTTGCATTGTT
GCAGCATTGGCCTGTTCCATTAAGGAACTGGACACTGTGAAAGTAGAATGCCTCGATAA
CGCGGTCCATCTGGAAAGTATGCAACACCACATCAGCATGACTGCCACGGACCTACAAA
AGACGATTGATAAGGAGAACAGCACAACGGACACTTCAGAATGCATCTGTGAAGACAAG
CGGTTGGAAAAGTCTTTCAAGGACTTCATTCAGAACATAAGACATTTAACTCAAGCTCA
TGCTGCAAAGCGTCTAAGTTCATAACTCGAG

MDRRYRISFLTLFLTGCLQGNPIPRLLAGIDYLEENITCPDSVFYTPTDVEDSCIVAAL
ACSIKELDTVKVECLDNAVHLESMQHHISMTATDLQKTIDKENSTTDTSECICEDKRLE
KSFKDFIQNIRHLTQAHAAKRLSS

pcDNA3.1-*trout-IL-15-FLAG*

This expression vector encodes rainbow trout FLAG-tagged IL-15 (named IL-15). Cloned from cDNA. The *IL-15* part of the sequence is identical with GenBank accession NM_001124395.

AAGCTTACCATGACAGGTTTTTTGACAGTGCTCCTTTTTTGCATTCGTTTATTGGAGCG
CAGGACAAAGAAAAGTGTGCGATGGATCTGTCTCTTCTGGGGTTTCCATTACTATCCAC
ACCAGCGTCTGAACATTGAGCTCTGGAATTGCTTCATAATATTGAGCTGCCTGAGTGCC
ACCGCACATTTGCCCATTGCTGGTGCTGCTGAAACACACGGGATGACAATAGATGACGT
TAAAGAGCTTCAGTCGGAGTTGAAAAACTTGAAAAGTACCATAGAAAAATCAGATGCCT
GTTTGTATGCTCCTACCAACGATGACATCTACAATGACCACTGCATCTTTAAGTTCATG
CACTGTTATTTATTGGAGTTGGAGGTTGTCCTGTTTGAGGACATGTCGGTCACAGATAA
CTACCATGACGAAATAAAAACATCCATCTACCATCGGAAAAAACATTTGGAAGAACATG
AACGCCAATATAACAGTTCTAGATGCTCACCGTGTGAGGCACAGAGAGTTGCCAACTCC
ACAATATTCCTCTACAACCTGGAGCGCCTTTTGGAGAGAATAGGGCAAACTGTCAGTGA
TTACAAGGATGACGACGATAAGTAACTCGAG

MTGFLTVLLFCIRLLERRTKKSVRWICLFWGFHYYPHQRLNIELWNCFIILSCLSATAH
LPIAGAAETHGMTIDDVKELQSELKNLKSTIEKSDACLYAPTNDDIYNDHCIFKFMHCY
LLELEVVLFEDMSVTDNYHDEIKTSIYHRKKHLEEHERQYNSSRCSPCEAQRVANSTIF
LYNLERLLERIGQTVSDYKDDDDK

pcDNA3.1-*trout-IL-15-(non-tagged)*

This expression vector encodes rainbow trout non-tagged IL-15 [named IL-15(N)]. Cloned from cDNA. The *IL-15* part of the sequence is identical with GenBank accession NM_001124395.

AAGCTTACCATGACAGGTTTTTTGACAGTGCTCCTTTTTTGCATTCGTTTATTGGAGCG
CAGGACAAAGAAAAGTGTGCGATGGATCTGTCTCTTCTGGGGTTTCCATTACTATCCAC
ACCAGCGTCTGAACATTGAGCTCTGGAATTGCTTCATAATATTGAGCTGCCTGAGTGCC
ACCGCACATTTGCCCATTGCTGGTGCTGCTGAAACACACGGGATGACAATAGATGACGT
TAAAGAGCTTCAGTCGGAGTTGAAAAACTTGAAAAGTACCATAGAAAAATCAGATGCCT
GTTTGTATGCTCCTACCAACGATGACATCTACAATGACCACTGCATCTTTAAGTTCATG
CACTGTTATTTATTGGAGTTGGAGGTTGTCCTGTTTGAGGACATGTCGGTCACAGATAA
CTACCATGACGAAATAAAAACATCCATCTACCATCGGAAAAAACATTTGGAAGAACATG
AACGCCAATATAACAGTTCTAGATGCTCACCGTGTGAGGCACAGAGAGTTGCCAACTCC
ACAATATTCCTCTACAACCTGGAGCGCCTTTTGGAGAGAATAGGGCAAACTGTCAGTTG
ACTCGAG

MTGFLTVLLFCIRLLERRTKKSVRWICLFWGFHYYPHQRLNIELWNCFIILSCLSATAH
LPIAGAAETHGMTIDDVKELQSELKNLKSTIEKSDACLYAPTNDDIYNDHCIFKFMHCY
LLELEVVLFEDMSVTDNYHDEIKTSIYHRKKHLEEHERQYNSSRCSPCEAQRVANSTIF
LYNLERLLERIGQTVS

pcDNA3.1-*trout-IL-15La-FLAG*

This expression vector encodes rainbow trout FLAG-tagged IL-15La (named IL-15La). Cloned from cDNA. The *IL-15La* part of the sequence is identical with GenBank accession MK619679.

AAGCTTACCATGCTGAGGAGACAGAGGACTGACACTCTTCTAGCCCTTTTGCTGTGGTT
TCTCTTCTTCATCGCCATGACAATGAAACAGGCATATGGAAAATCCATGTGCAGTAAAG
AACTTCCCGGAATTGTGCGAAAATGCATTGAGGAAGTTCACAAGATGGAATCATTTGAT
TGCAGACTGTACACCCCAACTTTGGCTGATTATCAGAAGTGCCCCACGTCCACACTCAT
ATGCTTTGAAAAAGAAGTGAATGTCCTGGTGTTAGAATCTGGGAATAAGTCCTCACCCA
TATACAAGCCAAAACTATCCATCCGGCTGAAGTCCTTGATCAAACAGAAAGAAGGTGCC
AACTGTCCAGACTGTGAGGCCCACAGAGAAAGGGCAGCAAAGGATTTCTTAACAACATT
GCAAACAATTCTGGAGTGGATGAACGATCAGGGGTGTCGGAAGCCATCCAGCCATGATT
ACAAGGATGACGACGATAAGTGACTCGAG

MLRRQRTDTLLALLLWFLFFIAMTMKQAYGKSMCSKELPGIVRKCIEEVHKMESFDCRL
YTPTLADYQKCPTSTLICFEKEVNVLVLESGNKSSPIYKPKLSIRLKSLIKQKEGANCP
DCEAHRERAAKDFLTTLQTILEWMNDQGCRKPSSHDYKDDDDK

pcDNA3.1-*trout-IL-15La-(non-tagged)*

This expression vector encodes rainbow trout un-tagged IL-15La [named IL-15La(N)]. Cloned from cDNA. The *IL-15La* part of the sequence is identical with GenBank accession MK619679.

AAGCTTACCATGCTGAGGAGACAGAGGACTGACACTCTTCTAGCCCTTTTGCTGTGGTT
TCTCTTCTTCATCGCCATGACAATGAAACAGGCATATGGAAAATCCATGTGCAGTAAAG
AACTTCCCGGAATTGTGCGAAAATGCATTGAGGAAGTTCACAAGATGGAATCATTTGAT
TGCAGACTGTACACCCCAACTTTGGCTGATTATCAGAAGTGCCCCACGTCCACACTCAT
ATGCTTTGAAAAAGAAGTGAATGTCCTGGTGTTAGAATCTGGGAATAAGTCCTCACCCA
TATACAAGCCAAAACTATCCATCCGGCTGAAGTCCTTGATCAAACAGAAAGAAGGTGCC
AACTGTCCAGACTGTGAGGCCCACAGAGAAAGGGCAGCAAAGGATTTCTTAACAACATT
GCAAACAATTCTGGAGTGGATGAACGATCAGGGGTGTCGGAAGCCATCCAGCCATTGAC
TCGAG

MLRRQRTDTLLALLLWFLFFIAMTMKQAYGKSMCSKELPGIVRKCIEEVHKMESFDCRL
YTPTLADYQKCPTSTLICFEKEVNVLVLESGNKSSPIYKPKLSIRLKSLIKQKEGANCP
DCEAHRERAAKDFLTTLQTILEWMNDQGCRKPSSH

pcDNA3.1-*trout-IL-15Lb-FLAG*

This expression vector encodes rainbow trout FLAG-tagged IL-15Lb (named IL-15Lb). Cloned from cDNA. The *IL-15Lb* part of the sequence is identical with GenBank accession MK619680.

AAGCTTACCATGTTGAGGAGACAGAGAACTGGCTCTCTTCTGAACGCTTTGCTGTGGTT
TCTCTTCTTCATTGCCATGACAATGAAACAGGCTTATGGACAATCCATTAGCAGTTCAG
AAATTCACCAAATTGTGAAAACATTTATTAAGGAGGAAGTTCACAGGATGGAATCATTT
GATTGCAGACTGTACACCCCAACTTTAGCTGATTATAAGAAATGTCCCAGGTCCACACT
CACATGCTTTACGACAGAAGTAAAAGTCCTGATGTTAGAATATGGGAAACGTTCTTCAT
CCTTACACCAGAAAAGACTCACCAAACGACTGACTAAATTGATGTCCTTGATAAAACAG
AAGGATGGTGCCAACTGTCCACACTGTGAGGTCCACAGAGAACAGGCAGCAAATGATTT
CTTAACAACATTACTAGGGATTCTGGAGTGGATGAACAATCAGGGGTCTCAGTTGCCAG
ACAGCCACGATTACAAGGATGACGACGATAAGTGACTCGAG

MLRRQRTGSLLNALLWFLFFIAMTMKQAYGQSISSSEIHQIVKTFIKEEVHRMESFDCR
LYTPTLADYKKCPRSTLTCFTTEVKVLMLEYGKRSSSLHQKRLTKRLTKLMSLIKQKDG
ANCPHCEVHREQAANDFLTTLLGILEWMNNQGSQLPDSHDYKDDDDK

pcDNA3.1-*trout-IL-15Lb-(non-tagged)*

This expression vector encodes rainbow trout un-tagged IL-15Lb [named IL-15Lb(N)]. Cloned from cDNA. The *IL-15Lb* part of the sequence is identical with GenBank accession MK619680.

AAGCTTACCATGTTGAGGAGACAGAGAACTGGCTCTCTTCTGAACGCTTTGCTGTGGTT
TCTCTTCTTCATTGCCATGACAATGAAACAGGCTTATGGACAATCCATTAGCAGTTCAG
AAATTCACCAAATTGTGAAAACATTTATTAAGGAGGAAGTTCACAGGATGGAATCATTT
GATTGCAGACTGTACACCCCAACTTTAGCTGATTATAAGAAATGtCCCAGGTCCACACT
CACATGCTTTACGACAGAAGTAAAAGTCCTGATGTTAGAATATGGGAAACGTTCTTCAT
CCTTACACCAGAAAAGACTCACCAAACGACTGACTAAATTGATGTCCTTGATAAAACAG
AAGGATGGTGCCAACTGTCCACACTGTGAGGTCCACAGAGAACAGGCAGCAAATGATTT
CTTAACAACATTACTAGGGATTCTGGAGTGGATGAACAATCAGGGGTCTCAGTTGCCAG
ACAGCCACTGACTCGAG

MLRRQRTGSLLNALLWFLFFIAMTMKQAYGQSISSSEIHQIVKTFIKEEVHRMESFDCR
LYTPTLADYKKCPRSTLTCFTTEVKVLMLEYGKRSSSLHQKRLTKRLTKLMSLIKQKDG
ANCPHCEVHREQAANDFLTTLLGILEWMNNQGSQLPDSH

<u>pcDNA3.1-*IL-2-Lead-RLI-trout-IL-15La*</u>

This expression vector is based on the RLI construct described by Mortier *et al.* 2006 (3), and encodes a genetic fusion of a human IL-2 leader, a FLAG-tag, a large part of the human IL-15Rα ectodomain, a glycine-serine linker (shaded blue), and the rainbow trout IL-15La sequence (named IL-15La-h-RLI; indicated as “RLI” in main text Fig. 8).
The linker plus *IL-15La* part were commercially ordered (Invitrogen) with the codons optimized for expression in insect cells, and fused to the N-terminal coding part by using the above described plasmid pcDNA3.1-*IL-2-Lead-RLI*, appropriate primers and overlap PCR.

```
GGTACCACCATGTACAGGATGCAACTCCTGTCTTGCATTGCACTAAGTCTTGCACTTGT
CACAAACAGTGACTACAAGGATGACGATGACAAGATAGAAGGTAGGATCACATGCCCTC
CCCCCATGTCCGTGGAACACGCAGACATCTGGGTCAAGAGCTACAGCTTGTACTCCAGG
GAGCGGTACATTTGTAACTCTGGTTTCAAGCGTAAAGCCGGCACGTCCAGCCTGACGGA
GTGCGTGTTGAACAAGGCCACGAATGTCGCCCACTGGACAACCCCCAGTCTCAAATGCA
TTAGAGACCCTGCCCTGGTTCACCAAAGGCCAGCGCCACCCTCTGGAGGCTCCGGTGGA
GGTGGGAGTGGCGGTGGATCCGGTGGCGGAGGCAGCAAGCAAGCTTACGGCAAGTCCAT
GTGCTCCAAGGAACTGCCCGGTATCGTGCGCAAGTGCATCGAAGAGGTGCACAAGATGG
AATCCTTCGACTGCCGTCTGTACACCCCCACCCTGGCTGACTACCAGAAGTGCCCTACC
TCCACCCTGATCTGCTTCGAGAAGGAAGTGAACGTCCTGGTCCTCGAGTCCGGCAACAA
GTCCTCCCCAATCTACAAGCCCAAGCTGTCCATCCGTCTGAAGTCCCTGATCAAGCAGA
AGGAAGGCGCTAACTGCCCCGACTGCGAGGCTCACAGAGAGCGTGCTGCTAAGGACTTC
CTGACCACCCTGCAGACCATCCTCGAGTGGATGAACGACCAGGGTTGCCGCAAGCCCTC
CTCCCACTAATCTAGA
```

```
MYRMQLLSCIALSLALVTNSDYKDDDDKIEGRITCPPPMSVEHADIWVKSYSLYSRERY
ICNSGFKRKAGTSSLTECVLNKATNVAHWTTPSLKCIRDPALVHQRPAPPSGGSGGGGS
GGGSGGGGSKQAYGKSMCSKELPGIVRKCIEEVHKMESFDCRLYTPTLADYQKCPTSTL
ICFEKEVNVLVLESGNKSSPIYKPKLSIRLKSLIKQKEGANCPDCEAHRERAAKDFLTT
LQTILEWMNDQGCRKPSSH
```

<u>pcDNA3.1-*trout-IL-15Rα-Myc*</u>

This expression vector encodes rainbow trout full-length Myc-tagged IL-15Rα (named IL-15Rα).
Cloned and polished from cDNA. The *IL-15Rα* part of the sequence is, except for 1 silent nucleotide exchange, identical with GenBank accession GFIN01030410.

```
AAGCTTACCATGCGGCTGCGTCTCCTTTCCCTCCTTCTTATCGTCAAAGTGTGTCGACT
CAATACTGTCAGTCCAGACGTGTGCCCACCCCTGTCTCATTGGAAGCTCACCAAGCCTT
TGAACACGAGGGAAATTTATCAAGAAAACGAATCCCTGCGCTACCAGTGTGTGGATGGC
TATGTGAGGAAGGCGGGAACCTCCAATCTCATCAAGTGCAAACGGATTGGTCAGGTTCT
TCAGTGGACACTCCCAACACTGATATGCATACCTGACCCATCCATTCCAACTACGATAG
AACCTTCAACAACCCACAAGACAACTCAATTTCCTACAACGCACCTCTCAGTGCCCACA
```

GAGAGAGTTAGCCCAAGACCAACCACTCTCAGTTCACTTTTATCTCAAACACTGCATTC
TGTCACAGAGACCCAAGAGGTCATTACTACGGTTACAGTGACAACCACCATCATCAGTG
ACATCACATCAGTGCCAGTGAATAAGTCCAGTGCGGTGACCTCTGTCTCCACGAAGACT
GTTTCTCCATCAGAGAGTTACTGTGTCCCCAACAGAGCCCCAACCTCCAGTGACTATCC
AGTTGGCGATGGAAGCACTGGGAAGAGTTCTGCTACAGCCATATATGCAGGTGTAGGTG
GGGTGGTAGTAACCATCTTGCTGGTTGCAGCGTTTGGAGTACTAATGTTTTGGAGAAGG
AAACGGATGCAGCGGCTTGATCTGTCGCCAACACCAGACGAAATTATGCAAATGAATGT
TGTGACAGAACAAAAACTCATCTCAGAAGAGGATCTGTGACTCGAG

MRLRLLSLLLIVKVCRLNTVSPDVCPPLSHWKLTKPLNTREIYQENESLRYQCVDGYVR
KAGTSNLIKCKRIGQVLQWTLPTLICIPDPSIPTTIEPSTTHKTTQFPTTHLSVPTERV
SPRPTTLSSLLSQTLHSVTETQEVITTVTVTTTIISDITSVPVNKSSAVTSVSTKTVSP
SESYCVPNRAPTSSDYPVGDGSTGKSSATAIYAGVGGVVVTILLVAAFGVLMFWRRKRM
QRLDLSPTPDEIMQMNVVTEQKLISEEDL

pcDNA3.1-*trout-solIL-15Rα-Myc*

This expression vector encodes rainbow trout soluble Myc-tagged IL-15Rα (named soluble IL-15Rα or sIL-15Rα).
Cloned and polished from cDNA. The *IL-15Rα* part of the sequence is, except for 1 silent nucleotide exchange, identical with GenBank accession GFIN01030410.

AAGCTTACCATGCGGCTGCGTCTCCTTTCCCTCCTTCTTATCGTCAAAGTGTGTCGACT
CAATACTGTCAGTCCAGACGTGTGCCCACCCCTGTCTCATTGGAAGCTCACCAAGCCTT
TGAACACGAGGGAAATTTATCAAGAAAACGAATCCCTGCGCTACCAGTGTGTGGATGGC
TATGTGAGGAAGGCGGGAACCTCCAATCTCATCAAGTGCAAACGGATTGGTCAGGTTCT
TCAGTGGACACTCCCAACACTGATATGCATACCTGACCCATCCATTCCAACTACGATAG
AACCTTCAACAACCCACAAGACAACTCAAGAACAAAAACTCATCTCAGAAGAGGATCTG
TGACTCGAG

MRLRLLSLLLIVKVCRLNTVSPDVCPPLSHWKLTKPLNTREIYQENESLRYQCVDGYVR
KAGTSNLIKCKRIGQVLQWTLPTLICIPDPSIPTTIEPSTTHKTTQEQKLISEEDL

Supplementary file 2D.
Vectors for expressing (modified) trout cytokines and trout IL-15Rα in insect cells using a baculovirus system.

<u>pFBD-P10Uhis-ieGFP-*trout-IL-2-FLAG*</u>

This expression vector encodes rainbow trout FLAG-tagged IL-2 (named IL-2). Cloned from cDNA. The *IL-2* part of the sequence is identical with GenBank accession FJ571512.

CCCGGGACCATGGACCGTCGTTACAGGATTTCCTTTTTGACGCTTTTTCTCACCGGTTG
TCTACAAGGAAACCCAATTCCCAGACTCCTAGCTGGAATCGATTATCTAGAAGAAAATA
TTACTTGTCCAGATTCAGTCTTCTATACACCAACTGATGTAGAGGATAGTTGCATTGTT
GCAGCATTGGCCTGTTCCATTAAGGAACTGGACACTGTGAAAGTAGAATGCCTCGATAA
CGCGGTCCATCTGGAAAGTATGCAACACCACATCAGCATGACTGCCACGGACCTACAAA
AGACGATTGATAAGGAGAACAGCACAACGGACACTTCAGAATGCATCTGTGAAGACAAG
CGGTTGGAAAAGTCTTTCAAGGACTTCATTCAGAACATAAGACATTTAACTCAAGCTCA
TGCTGCAAAGCGTCTAAGTTCAGATTACAAGGATGACGACGATAAGTAACCCGGG

MDRRYRISFLTLFLTGCLQGNPIPRLLAGIDYLEENITCPDSVFYTPTDVEDSCIVAAL
ACSIKELDTVKVECLDNAVHLESMQHHISMTATDLQKTIDKENSTTDTSECICEDKRLE
KSFKDFIQNIRHLTQAHAAKRLSSDYKDDDDK

<u>pFBD-P10Uhis-ieGFP-*trout-IL-15-FLAG*</u>

This expression vector encodes rainbow trout FLAG-tagged IL-15 (named IL-15). The sequence was commercially ordered (Invitrogen). The signal peptide is replaced with that of rainbow trout IL-2. The *IL-2* part of the sequence is identical with GenBank accession FJ571512. The *IL-15* part of the sequence is identical with GenBank accession NM_001124395. The encoded IL-2 leader sequence is shaded blue.

CCCGGGACCATGGACCGTCGTTACAGGATTTCCTTTTTGACGCTTTTTCTCACCGGTTG
TCTACAAGGAGCTGAAACACACGGGATGACAATAGATGACGTTAAAGAGCTTCAGTCGG
AGTTGAAAAACTTGAAAAGTACCATAGAAAAATCAGATGCCTGTTTGTATGCTCCTACC
AACGATGACATCTACAATGACCACTGCATCTTTAAGTTCATGCACTGTTATTTATTGGA
GTTGGAGGTTGTCCTGTTTGAGGACATGTCGGTCACAGATAACTACCATGACGAAATAA
AAACATCCATCTACCATCGGAAAAAACATTTGGAAGAACATGAACGCCAATATAACAGT
TCTAGATGCTCACCGTGTGAGGCACAGAGAGTTGCCAACTCCACAATATTCCTCTACAA
CCTGGAGCGCCTTTTGGAGAGAATAGGGCAAACTGTCAGTGATTACAAGGATGACGACG
ATAAGTGACCCGGG

MDRRYRISFLTLFLTGCLQGAETHGMTIDDVKELQSELKNLKSTIEKSDACLYAPTNDD
IYNDHCIFKFMHCYLLELEVVLFEDMSVTDNYHDEIKTSIYHRKKHLEEHERQYNSSRC
SPCEAQRVANSTIFLYNLERLLERIGQTVSDYKDDDDK

pFBD-P10Uhis-ieGFP-*trout-IL-15La-FLAG*

This expression vector encodes rainbow trout FLAG-tagged IL-15La (named IL-15La). Cloned from cDNA. The *IL-15La* part of the sequence is identical with GenBank accession MK619679.

CCCGGGACCATGCTGAGGAGACAGAGGACTGACACTCTTCTAGCCCTTTTGCTGTGGTT
TCTCTTCTTCATCGCCATGACAATGAAACAGGCATATGGAAAATCCATGTGCAGTAAAG
AACTTCCCGGAATTGTGCGAAAATGCATTGAGGAAGTTCACAAGATGGAATCATTTGAT
TGCAGACTGTACACCCCAACTTTGGCTGATTATCAGAAGTGCCCCACGTCCACACTCAT
ATGCTTTGAAAAAGAAGTGAATGTCCTGGTGTTAGAATCTGGGAATAAGTCCTCACCCA
TATACAAGCCAAAACTATCCATCCGGCTGAAGTCCTTGATCAAACAGAAAGAAGGTGCC
AACTGTCCAGACTGTGAGGCCCACAGAGAAAGGGCAGCAAAGGATTTCTTAACAACATT
GCAAACAATTCTGGAGTGGATGAACGATCAGGGGTGTCGGAAGCCATCCAGCCATGATT
ACAAGGATGACGACGATAAGTAACCCGGG

MLRRQRTDTLLALLLWFLFFIAMTMKQAYGKSMCSKELPGIVRKCIEEVHKMESFDCRL
YTPTLADYQKCPTSTLICFEKEVNVLVLESGNKSSPIYKPKLSIRLKSLIKQKEGANCP
DCEAHRERAAKDFLTTLQTILEWMNDQGCRKPSSHDYKDDDDK

pFBD-P10Uhis-ieGFP-*trout-solIL-15Rα-Myc*

This expression vector encodes rainbow trout soluble Myc-tagged IL-15Rα (named soluble IL-15Rα or sIL-15Rα).
Cloned from cDNA. The *IL-15Rα* part of the sequence is, except for 1 silent nucleotide exchange, identical with GenBank accession GFIN01030410.

CCCGGGACCATGCGGCTGCGTCTCCTTTCCCTCCTTCTTATCGTCAAAGTGTGTCGACT
CAATACTGTCAGTCCAGACGTGTGCCCACCCCTGTCTCATTGGAAGCTCACCAAGCCTT
TGAACACGAGGGAAATTTATCAAGAAAACGAATCCCTGCGCTACCAGTGTGTGGATGGC
TATGTGAGGAAGGCGGGAACCTCCAATCTCATCAAGTGCAAACGGATTGGTCAGGTTCT
TCAGTGGACACTCCCAACACTGATATGCATACCTGACCCATCCATTCCAACTACGATAG
AACCTTCAACAACCCACAAGACAACTCAAGAACAAAAACTCATCTCAGAAGAGGATCTG
TGACCCGGG

MRLRLLSLLLIVKVCRLNTVSPDVCPPLSHWKLTKPLNTREIYQENESLRYQCVDGYVR
KAGTSNLIKCKRIGQVLQWTLPTLICIPDPSIPTTIEPSTTHKTTQEQKLISEEDL

<u>pFBD-P10Uhis-ieGFP-*trout-IL-15-RLI*</u>

This expression vector is based on the RLI construct described by Mortier *et al.* 2006 (3), and encodes a genetic fusion of a human IL-2 leader, a FLAG-tag, a large part of the trout IL-15Rα ectodomain, a glycine-serine linker (shaded blue), and the rainbow trout IL-15 sequence (named IL-15-RLI).
The sequence was commercially ordered (Invitrogen). The *IL-15Rα* part of the sequence is, except for 1 silent nucleotide exchange, identical with GenBank accession GFIN01030410. The *IL-15* part of the sequence is identical with GenBank accession NM_001124395.

CCCGGGACCATGTACAGGATGCAACTCCTGTCTTGCATTGCACTAAGTCTTGCACTTGT
CACAAACAGTGACTACAAGGATGACGATGACAAGAATACTGTCAGTCCAGACGTGTGCC
CACCCCTGTCTCATTGGAAGCTCACCAAGCCTTTGAACACGAGGGAAATTTATCAAGAA
AACGAATCCCTGCGCTACCAGTGTGTGGATGGCTATGTGAGGAAGGCGGGAACCTCCAA
TCTCATCAAGTGCAAACGGATTGGTCAGGTTCTTCAGTGGACACTCCCAACACTGATAT
GCATACCTGACCCATCCATTCCAACTACGATAGAACCTTCAACATCTGGAGGCTCCGGT
GGAGGTGGGAGTGGCGGTGGATCCGGTGGCGGAGGCAGCGAAACACACGGGATGACAAT
AGATGACGTTAAAGAGCTTCAGTCGGAGTTGAAAAACTTGAAAAGTACCATAGAAAAAT
CAGATGCCTGTTTGTATGCTCCTACCAACGATGACATCTACAATGACCACTGCATCTTT
AAGTTCATGCACTGTTATTTATTGGAGTTGGAGGTTGTCCTGTTTGAGGACATGTCGGT
CACAGATAACTACCATGACGAAATAAAAACATCCATCTACCATCGGAAAAAACATTTGG
AAGAACATGAACGCCAATATAACAGTTCTAGATGCTCACCGTGTGAGGCACAGAGAGTT
GCCAACTCCACAATATTCCTCTACAACCTGGAGCGCCTTTTGGAGAGAATAGGGCAAAC
TGTCAGTTGACCCGGG

MYRMQLLSCIALSLALVTNSDYKDDDDKNTVSPDVCPPLSHWKLTKPLNTREIYQENES
LRYQCVDGYVRKAGTSNLIKCKRIGQVLQWTLPTLICIPDPSIPTTIEPSTSGGSGGGG
SGGGSGGGGSETHGMTIDDVKELQSELKNLKSTIEKSDACLYAPTNDDIYNDHCIFKFM
HCYLLELEVVLFEDMSVTDNYHDEIKTSIYHRKKHLEEHERQYNSSRCSPCEAQRVANS
TIFLYNLERLLERIGQTVS

<u>pFBD-P10Uhis-ieGFP-*trout-IL-15La-RLI*</u>

This expression vector is based on the RLI construct described by Mortier *et al.* 2006 (3), and encodes a genetic fusion of a human IL-2 leader, a FLAG-tag, a large part of the rainbow trout IL-15Rα ectodomain, a glycine-serine linker (shaded blue), and the rainbow trout IL-15La sequence (named IL-15La-RLI).
The sequence was commercially ordered (Invitrogen). The *IL-15Rα* part of the sequence is, except for 1 silent nucleotide exchange, identical with GenBank accession GFIN01030410. The *IL-15La* codons were optimized for expression in insect cells.

CCCGGGACCATGTACAGGATGCAACTCCTGTCTTGCATTGCACTAAGTCTTGCACTTGT
CACAAACAGTGACTACAAGGATGACGATGACAAGAATACTGTCAGTCCAGACGTGTGCC
CACCCCTGTCTCATTGGAAGCTCACCAAGCCTTTGAACACGAGGGAAATTTATCAAGAA
AACGAATCCCTGCGCTACCAGTGTGTGGATGGCTATGTGAGGAAGGCGGGAACCTCCAA

TCTCATCAAGTGCAAACGGATTGGTCAGGTTCTTCAGTGGACACTCCCAACACTGATAT
GCATACCTGACCCATCCATTCCAACTACGATAGAACCTTCAACATCTGGAGGCTCCGGT
GGAGGTGGGAGTGGCGGTGGATCCGGTGGCGGAGGCAGCAAGCAAGCTTACGGCAAGTC
CATGTGCTCCAAGGAACTGCCCGGTATCGTGCGCAAGTGCATCGAAGAGGTGCACAAGA
TGGAATCCTTCGACTGCCGTCTGTACACCCCCACCCTGGCTGACTACCAGAAGTGCCCT
ACCTCCACCCTGATCTGCTTCGAGAAGGAAGTGAACGTCCTGGTCCTCGAGTCCGGCAA
CAAGTCCTCCCCAATCTACAAGCCCAAGCTGTCCATCCGTCTGAAGTCCCTGATCAAGC
AGAAGGAAGGCGCTAACTGCCCCGACTGCGAGGCTCACAGAGAGCGTGCTGCTAAGGAC
TTCCTGACCACCCTGCAGACCATCCTCGAGTGGATGAACGACCAGGGTTGCCGCAAGCC
CTCCTCCCACTAACCCGGG

MYRMQLLSCIALSLALVTNSDYKDDDDKNTVSPDVCPPLSHWKLTKPLNTREIYQENES
LRYQCVDGYVRKAGTSNLIKCKRIGQVLQWTLPTLICIPDPSIPTTIEPSTSGGSGGGG
SGGGSGGGGSKQAYGKSMCSKELPGIVRKCIEEVHKMESFDCRLYTPTLADYQKCPTST
LICFEKEVNVLVLESGNKSSPIYKPKLSIRLKSLIKQKEGANCPDCEAHRERAAKDFLT
TLQTILEWMNDQGCRKPSSH

References used in this file

1. Dijkstra JM, Takizawa F, Fischer U, Friedrich M, Soto-Lampe V, Lefèvre C, et al. Identification of a gene for an ancient cytokine, interleukin 15-like, in mammals; interleukins 2 and 15 co-evolved with this third family member, all sharing binding motifs for IL-15Ra. Immunogenetics. (2014) 66:93–103. doi: 10.1007/s00251-013-0747-0

2. Yamaguchi T, Schares S, Fischer U, Dijkstra JM. Identification of a fourth ancient member of the IL-3/IL-5/GM-CSF cytokine family, KK34, in many mammals. Dev Comp Immunol. (2016) 65:268–79. doi: 10.1016/j.dci.2016.07.018

3. Mortier E, Quéméner A, Vusio P, Lorenzen I, Boublik Y, Grötzinger J, et al. Soluble interleukin-15 receptor alpha (IL-15R alpha)-sushi as a selective and potent agonist of IL-15 action through IL-15R beta/gamma. Hyperagonist IL-15 × IL-15R alpha fusion proteins. J Biol Chem. (2006) 281:1612–9. doi: 10.1074/jbc.M508624200

4. Ring AM, Lin JX, Feng D,Mitra S, RickertM, Bowman GR, et al. Mechanistic and structural insight into the functional dichotomy between IL-2 and IL-15. Nat Immunol. (2012) 13:1187–95. doi: 10.1038/ni.2449

5. Bessard A, Solé V, Bouchaud G, Quéméner A, Jacques Y. High antitumor activity of RLI, an interleukin-15 (IL-15)-IL-15 receptor alpha fusion protein, in metastatic melanoma and colorectal cancer. Mol Cancer Ther. (2009) 8:2736–45. doi: 10.1158/1535-7163.MCT-09-0275

6. VincentM, Bessard A, Cochonneau D, Teppaz G, Solé V,MaillassonM, et al. Tumor targeting of the IL-15 superagonist RLI by an anti-GD2 antibody strongly enhances its antitumor potency. Int J Cancer. (2013) 133:757–65. doi: 10.1002/ijc.28059
