# Supplementary material for: Ancient Cytokine Interleukin 15-Like (IL-15L) Induces a Type 2 Immune Response
Source: Front Immunol. 2020 Oct 29;11:549319. doi: 10.3389/fimmu.2020.549319 (PMC7658486; doi:10.3389/fimmu.2020.549319)
Supplement: Supplementary file 3 [file Data_Sheet_3.pdf]

Supplementary file 3

Isolation of rainbow trout lymphocyte subpopulations by flow cytometry

Table of Contents **Page**

Introduction and general explanation of the file contents 2

3A: Establishment of a new monoclonal antibody against rainbow trout CD8α 4

3B: Representative sortings of $CD8\alpha^+$ versus $CD8\alpha^-$ rainbow trout lymphocytes 6

3C: Representative sorting of $CD4^-CD8^-$ (double negative, DN), $CD4^+CD8^+$ (double positive, DP), CD4 single positive (SP) and CD8SP rainbow trout lymphocytes from thymus 8

3D: Representative sorting of CD4SP, CD8SP, IgMSP and $CD4^-CD8^-IgM^-$ triple negative (TN) rainbow trout splenocytes 10

3E: Percentages of CD4SP, CD8SP, IgMSP and TN cells among rainbow trout spleen lymphocytes 11

References used in this file 12

Introduction and general explanation of the file contents.

Teleost fish lack lymph nodes [e.g. (1)], but they possess a bona fide spleen and thymus [e.g. (1-6)], while the head kidney (HK; pronephros) simultaneously is a hematopoietic organ and a secondary lymphoid organ [e.g. (4, 7, 8)]. It has also been shown that gills as the respiratory organ of teleosts [e.g. (4, 6)] and the intestine [e.g. (4, 9)] are mucosal organs that contain numerous lymphocytes. Therefore, in this study, rainbow trout leukocytes were isolated from the lymphoid tissues spleen, thymus and HK, and from the mucosal tissues gill and intestine.

There are several lines of evidence that indicate that teleost fish cytotoxic TCRαβ T cells express CD8α as known in mammals [e.g. (10-13)]. Whereas mammals have only one CD4 molecule, teleost fish have two quite diverged CD4-1 and CD4-2 molecules which in lymphocytes are mostly co-expressed [e.g. (14-17)]; both CD4-1 and CD4-2 show expression patterns and molecular features consistent with a CD4 marker function of helper and regulatory T cells as known in mammals [e.g. (14-21)]. Fish B cells do not undergo an immunoglobulin class switch thus lacking IgG, IgA and IgE, and the main B cell population expresses IgM [e.g. (22)].

In the present study, monoclonal antibodies (mAbs) against trout CD8α, CD4-1, CD4-2 and IgM were used to label leukocytes that had been isolated by Percoll density gradient centrifugation, after which cells with lymphocyte features (FSC^{low} and SSC^{low} gated cells; described below as “lymphocytes”) were flow sorted into subpopulations depending on their labeling. To allow distinguishing usage of anti-CD8α together with other mAbs, a new mAb was established against trout CD8α possessing a different IgG isotype than the already available mAb 13.2D (10); this is explained in Supplementary file 3A. For CD4 labeling, a mixture containing both mAb 4.1.2 which recognizes trout CD4-1 and mAb 4.2.12 which recognizes trout CD4-2 (23) was used. For IgM labeling the mAb 1.14 was used (24). Doublets and dead cells were excluded by FSC-A/FSC-H gating and by propidium iodide (PI) or 4’, 6-diamidino-2-phenylin-dole (DAPI) staining, respectively.

Details on the antibodies used for flow cytometry are:

Primary antibodies:

mAb 13.2D: anti-trout CD8α, rat IgG2a isotype (10)
mAb 7α8c: anti-trout CD8α, rat IgG1 isotype (Supplementary file 3A)
mAb 4.1.2: anti-trout CD4-1, rat IgG2a isotype (14)
mAb 4.2.12: anti-trout CD4-2, rat IgG2b isotype (14)
mAb 1.14: anti-trout IgM, mouse IgG1 isotype (24)

Fluorochrome-conjugated secondary antibodies:

anti-rat IgG Alexa Fluor 488 (Thermo Fischer Scientific)
anti-rat IgG1-FITC (BD Bioscience)
anti-rat IgG2a-PE (eBioscience)
anti-rat IgG2a-eFluor 660 (eBioscience)
anti-rat IgG2b-PE (eBioscience)
anti-rat IgG2b-eFluor 660 (eBioscience)

anti-mouse IgG1-Brilliant Violet 421™ (Biolegend)

In the various experiments, different sets of the above listed antibodies were used for the flow sorting of lymphocyte subpopulations. The Supplementary files 3B-to-3E are representative results for the flow sorting used in the present study, concerning lymphocytes from different tissues and different antibody combinations.
Supplementary file 3B shows representative sorting results for lymphocytes of various tissues when using primary antibody anti-trout CD8α (mAb 13.2D) plus secondary antibody anti-rat IgG Alexa Fluor 488. Two subpopulations were obtained, being $CD8^+$ and $CD8^-$ lymphocytes. This type of sorting was used for experiments of which results are shown in Supplementary file 5C.

Supplementary file 3C shows representative sorting results for lymphocytes of various tissues when using primary antibodies anti-trout CD8α (mAb 7α8c), anti-trout CD4-1 (mAb 4.1.2), and anti-trout CD4-2 (mAb 4.2.12), plus secondary antibodies anti-rat IgG1-FITC, anti-rat IgG2a-PE and anti-rat IgG2b-PE. Four subpopulations were obtained from thymus, being CD4SP, CD8SP, $CD4^-CD8^-$ (DN) and $CD4^+CD8^+$ (DP) lymphocytes while three subpopulations were obtained from spleen and intestine, being CD4SP, CD8SP and DN. This type of sorting was used for experiments of which results are shown in main text Fig. 9, and in Supplementary files 5C(f), 5C(k), 5D and 5E.

Supplementary file 3D shows representative sorting results for lymphocytes of various tissues when using primary antibodies anti-trout CD8α (mAb 7α8c), anti-trout CD4-1 (mAb 4.1.2), anti-trout CD4-2 (mAb 4.2.12), and anti-trout IgM (mAb 1.14), plus secondary antibodies anti-rat IgG1-FITC, anti-rat IgG2a-eFluor 660, anti-rat IgG2b-eFluor 660 and anti-mouse IgG1-Brilliant Violet 421™. Four subpopulations were obtained, being CD4SP (aka $CD4^+$), CD8SP (aka $CD8^+$), IgMSP (aka IgM^+), and $CD4^-CD8^-IgM^-$ (TN) lymphocytes. This type of sorting was used for experiments of which results are shown in main text Fig. 11 and in Supplementary file 6C.

Supplementary file 3E summarizes the relative numbers of the CD8SP (aka $CD8^+$), CD4SP (aka $CD4^+$) IgMSP (aka IgM^+), and $CD4^-CD8^-IgM^-$ (TN) cells among the spleen lymphocytes used for the experiments resulting in main text Fig. 11 (and Supplementary file 6C). This summary intends to help with the interpretation at the whole tissue level of the gene expression patterns observed within cell subpopulations.

At a note: Like in mammals, teleost lymphocytes can be distinguished from monocytes/macrophages and granulocytes by flow cytometry according to their scatter characteristics (FSC^{low} and SSC^{low}) as small non-granulated cells [e.g. (8, 10, 14)]. Cells within this population are referred to as “lymphocyte gate cells” or “morphological lymphocytes”, and often are simply called “lymphocytes” in the present paper. However, it should be mentioned that teleost thrombocytes are nucleated cells with similar scatter characteristics as lymphocytes [e.g. (25)] and thus be contained in the corresponding negative populations.

Supplementary file 3A Establishment of a new mAb against rainbow trout CD8α.

A new anti-trout CD8α mAb 7α8c with another Ig isotype than the previously published anti-trout CD8α mAb 13.2D (50) was established because its Ig isotype was more suitable for multicolor staining in combination with mAbs against other molecules.

(a) Flow cytometry analysis of HEK293T cells transfected for expression of HA-tagged trout CD8α (HEK293T-CD8α-HA) and parental HEK293T cells stained with anti-HA (upper plots) and anti-trout CD8α 7α8c mAb (lower plots). (b) Rainbow trout leukocytes from intestine were stained with anti-CD8α 7α8c mAb and FITC-conjugated goat anti-rat IgG, and sorted into 7α8c-negative (-) and 7α8c-positive (+) lymphocytes. RNA was extracted from both populations and analyzed by RT-PCR using specific primers for *CD8α* (upper panel) and *β-actin* (lower panel). Distilled water served as a negative control. Numbers of cycles are provided on the right side of the figure.

(a) *Confirmation of 7α8c antibody specificity by flow cytometry analysis of HEK293T cells transfected for rainbow trout CD8α*

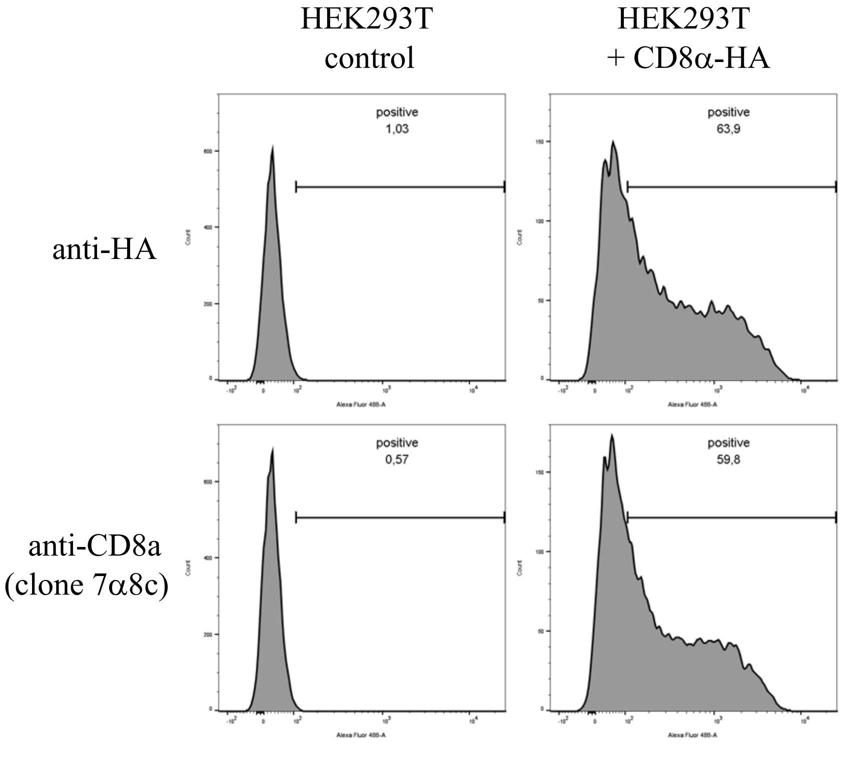

(Supplementary file 3A)

(b) *Confirmation of 7α8c antibody specificity by semi-quantitative RT-PCR analysis of flow-sorted rainbow trout intestinal lymphocytes*

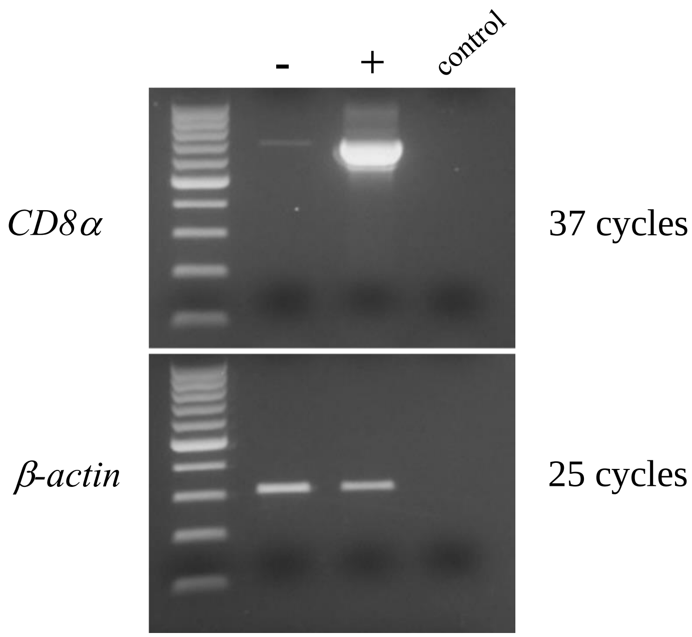

Supplementary file 3B Representative sortings of CD8α+ versus CD8α- rainbow trout lymphocytes.

Leukocytes from thymus (a), intestine (b), head kidney (c), spleen (d) and gill (e) were stained with anti-CD8α mAb. The X-axis depicts relative cell size (FSC; forward scatter) while the Y-axis represents fluorescent intensity with the anti-CD8α mAb. The Y-axis is set to a biexponential scale. FSC^{low} and SSC^{low} lymphocytes were gated and fluorescence intensities from unsorted leukocytes (Pre-sort; left panel), sorted CD8α- lymphocytes (Negative; middle panel) and sorted CD8α+ lymphocytes (Positive; right panel) were plotted. CD8α+ lymphocytes are depicted in the upper right quadrants while the CD8α- cells appear in the lower right quadrants, respectively. Data are from a single representative of at least two independent experiments. In each experiment, leukocytes from 4 - 6 clonal individual trout were pooled.

(a) Rainbow trout thymocytes

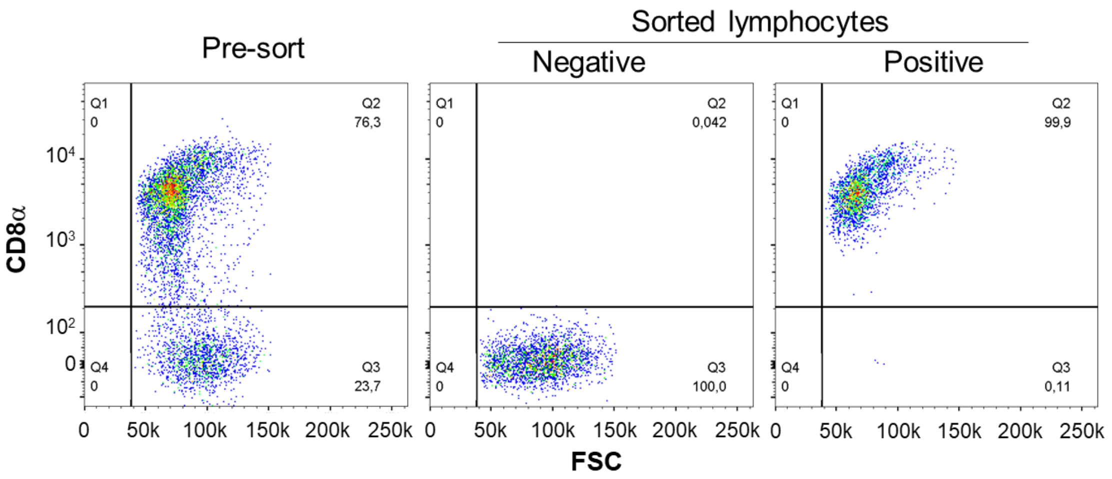

(b) Rainbow trout intestine lymphocytes

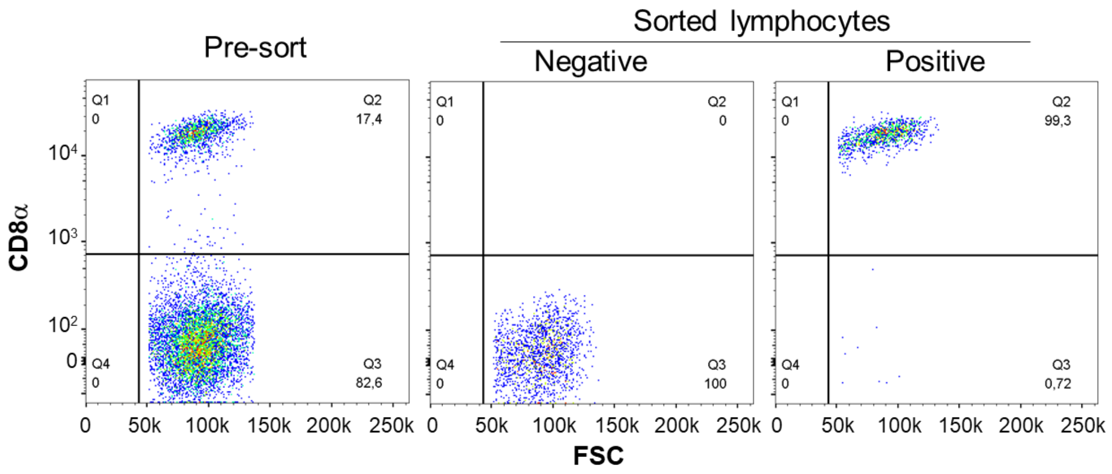

(Supplementary file 3B)

(c) Rainbow trout head kidney lymphocytes

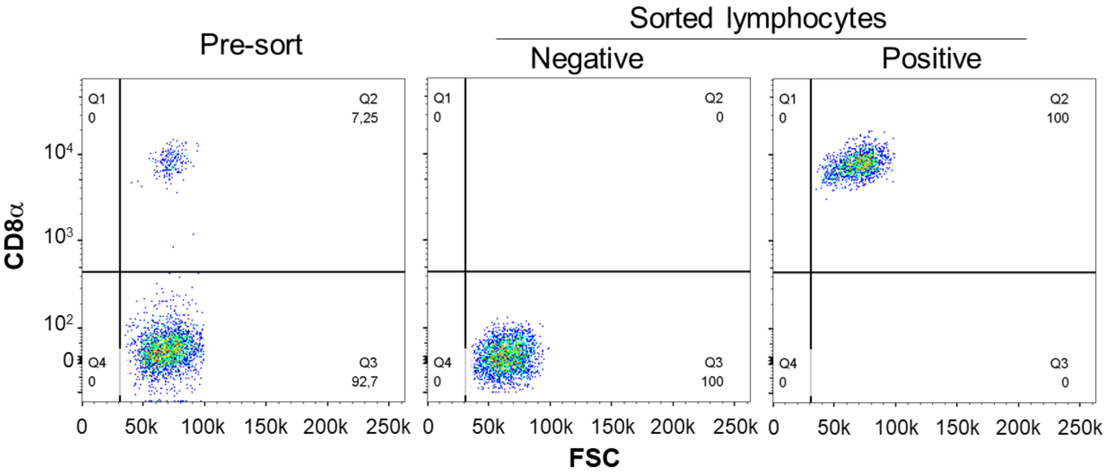

(d) Rainbow trout spleen lymphocytes

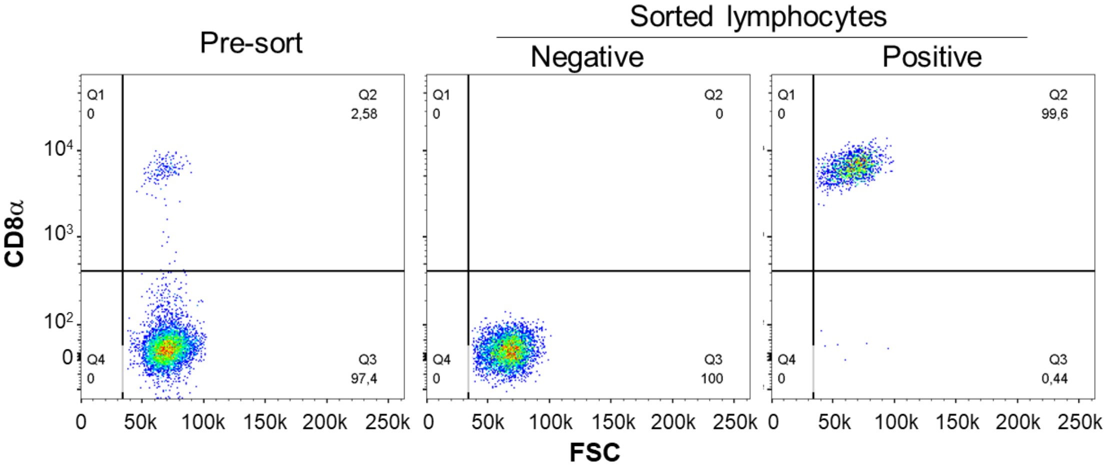

(e) Rainbow trout gill lymphocytes

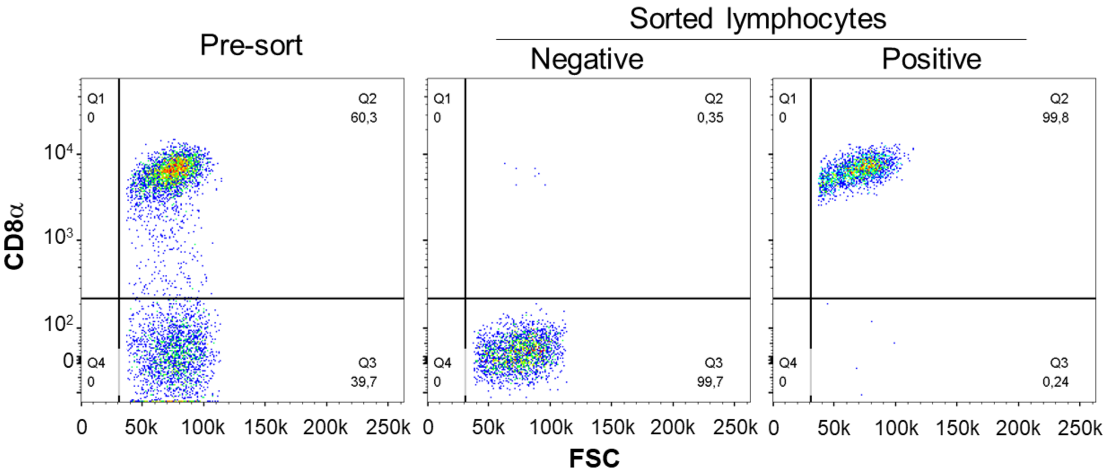

Supplementary file 3C Representative sortings of DN, DP, CD4SP and CD8SP rainbow trout lymphocytes from thymus, intestine and spleen.

Leukocytes from thymus (a), intestine (b), and spleen (c) were stained with anti-CD8α, anti-CD4-1 and anti-CD4-2 mAbs and sorted into the respective subpopulations. The X-axis depicts fluorescent intensity of staining with anti-CD8α mAb while the Y-axis represents fluorescent intensity with the anti-CD4 mAbs. Both axes are set to a biexponential scale. FSC^{low} and SSC^{low} lymphocytes were gated and their fluorescence intensities were plotted as follows: (a) stainings of thymocytes prior to sorting (Pre-sort; left panel), sorted DN thymocytes (upper middle panel), sorted DP thymocytes (upper right panel), sorted CD8SP thymocytes (lower middle panel) and sorted CD4SP thymocytes (lower right panel). Figures (b) and (c) show stainings of lymphocytes prior to sorting (Pre-sort; far left panel), sorted DN lymphocytes (left panel), sorted CD8SP lymphocytes (CD8SP; right panel) and sorted CD4SP lymphocytes (far right panel). Data are from a single representative of 5 independent experiments. In each experiment, leukocytes from 6 - 8 clonal individual trout were pooled.

(a) *Rainbow trout thymocytes*

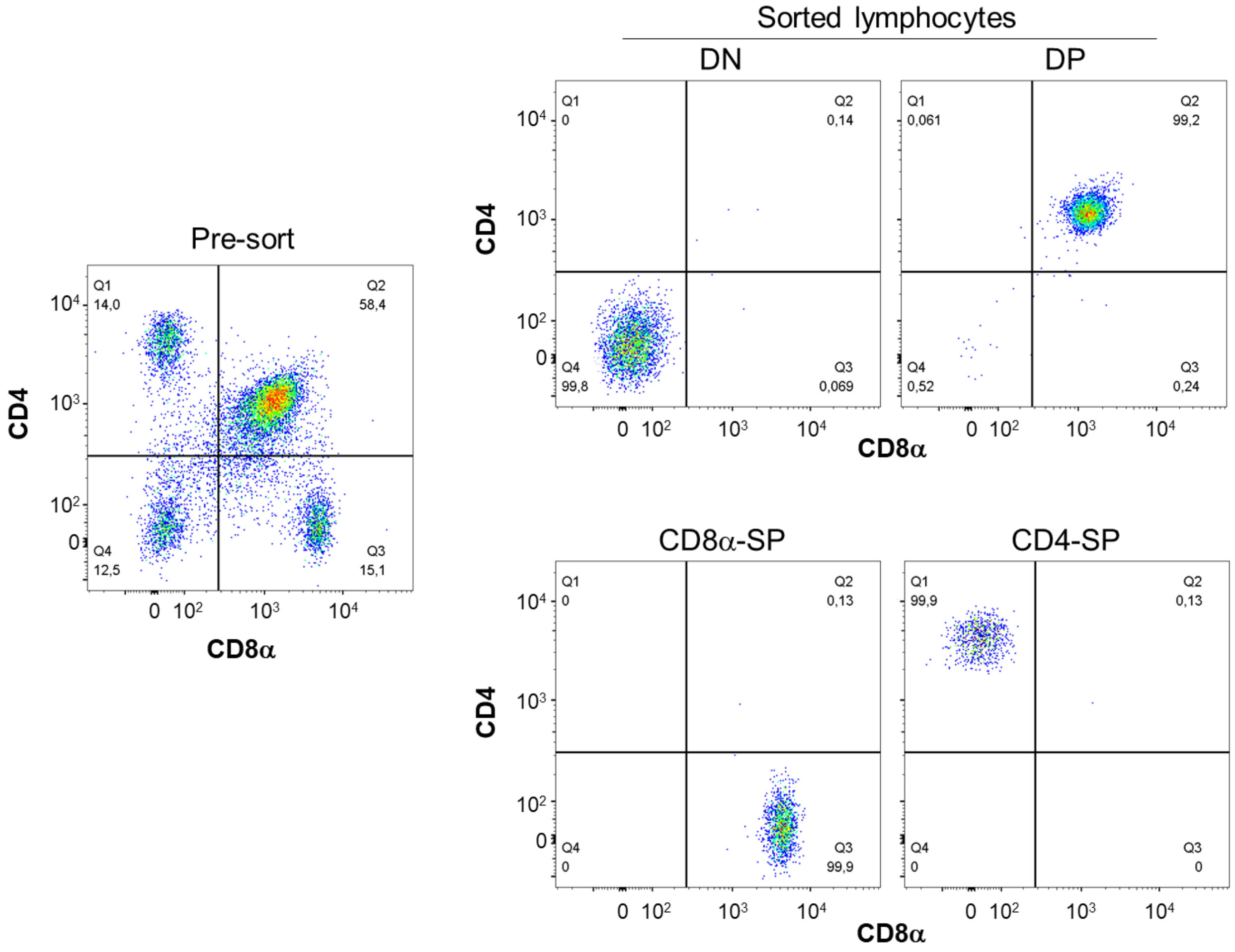

(Supplementary file 3C)

(b) *Rainbow trout intestine lymphocytes*

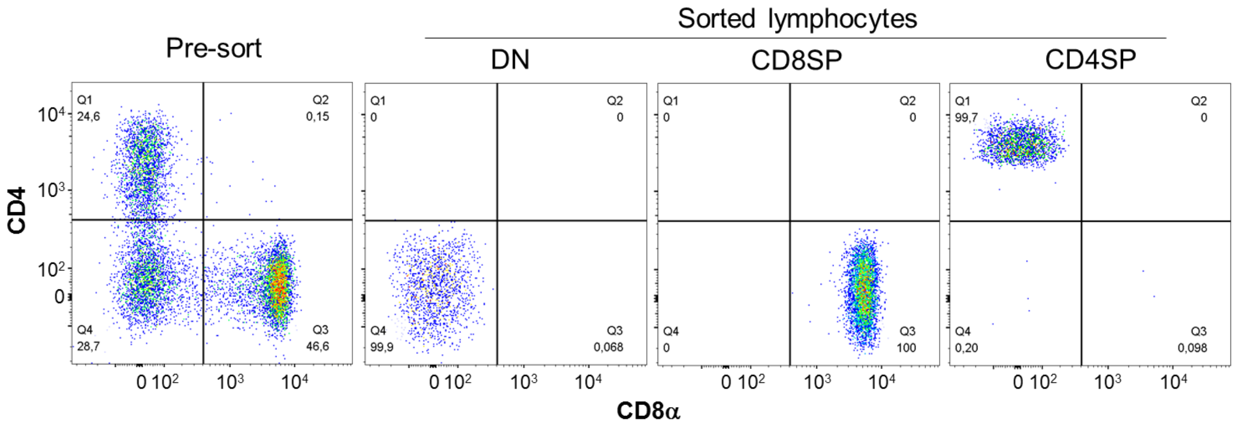

(c) *Rainbow trout spleen lymphocytes*

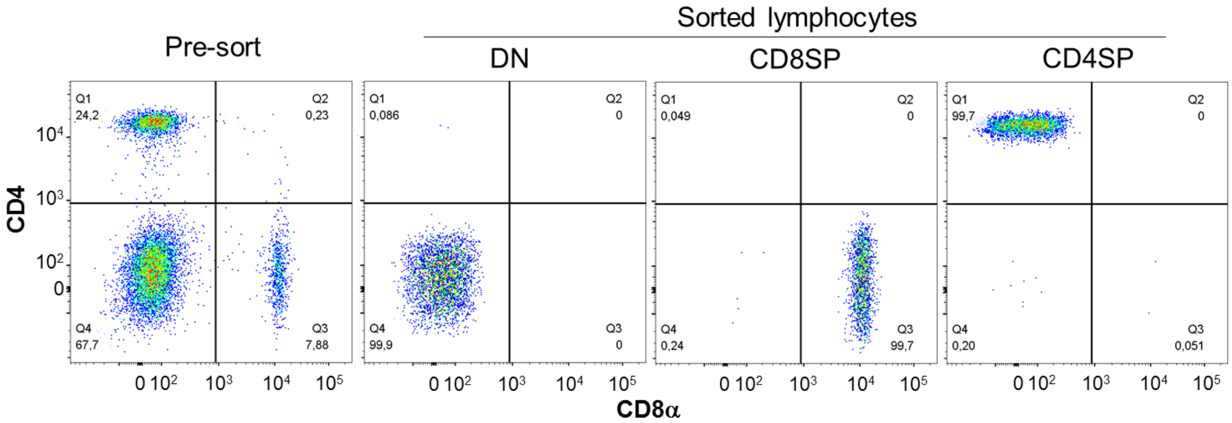

Supplementary file 3D Representative sorting of CD4SP, CD8SP, IgMSP and TN (triple negative; CD4$^-$CD8$^-$IgM$^-$) rainbow trout splenocytes.

Splenocytes were stained with anti-CD8α, anti-CD4-1, anti-CD4-2 and anti-IgM mAbs and flow sorted. FSC^{low} and SSC^{low} lymphocytes were gated, and considered for further sorting steps. Representative dot plots show CD4 versus CD8α (a), IgM versus CD8α (b), and IgM versus CD4 (c). The fluorescence intensities of stainings prior to sorting (left Pre-sort panels) was plotted. CD4$^-$ CD8$^-$ double negative (DN) populations were further gated and used for sorting into IgM$^+$ and IgM$^-$ splenocytes. Fluorescence intensities of sorted cells are depicted in the right panels: TN splenocytes (far left panels), sorted CD4SP (left panels), sorted CD8SP splenocytes (right panels) and sorted IgMSP splenocytes (far right panels). Both axes are set to a biexponential scale. Data are from a single representative of 5 independent experiments. In each experiment, leukocytes from 8 individuals were pooled.

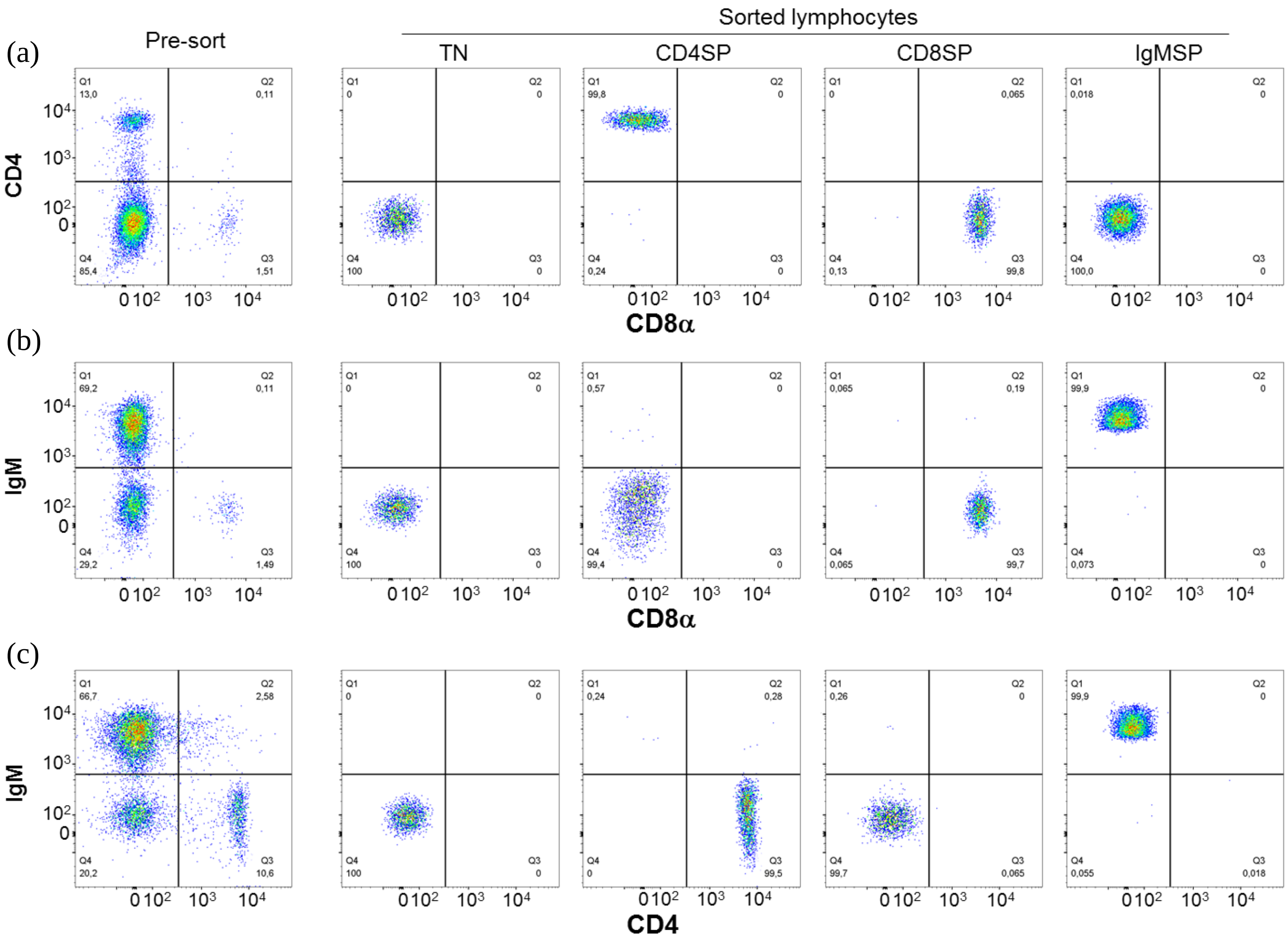

Supplementary file 3E Percentages of CD4SP, CD8SP, IgMSP and TN cells among rainbow trout spleen lymphocytes.

For the experiments of which the results are shown in main text Fig. 11, spleens of 8 individuals were pooled in four independent experiments. For one of those experiments, the sorting results into CD4SP, CD8SP, IgMSP and TN cell subpopulations are shown in Supplementary file 3D. The tables below summarize the percentages of subpopulation cells relative to spleen lymphocytes for each of the four experiments. Table (a) shows the raw data provided by the cell sorter, and Table (b) shows calculated percentages of IgM^+ and TN splenocytes, based on dividing the percentage of DN splenocytes according to the ratio of IgM^+ and IgM^- splenocytes.

(a)

% in lymphocyte gate	CD8SP	CD4SP	DN		within DN	
					IgM+	IgM- (TN)
#1	7.6	29.3	62.9		53.3	46.7
#2	6.1	23.4	70.3		52.8	47.2
#3	6.5	22.0	71.4		59.8	40.2
#4	5.7	20.2	74.0		56.0	44.0

(b)

% in lymphocyte gate	CD8SP	CD4SP	IgMSP	TN
#1	7.6	29.3	33.5	29.4
#2	6.1	23.4	37.1	33.2
#3	6.5	22.0	42.7	28.7
#4	5.7	20.2	41.4	32.6
Av.	**6.5**	**23.7**	**38.7**	**31.0**

References used in this file

1. Lieschke GJ, Trede NS. Fish immunology. Curr Biol. (2009) 19.16:R678–82. doi: 10.1016/j.cub.2009.06.068

2. Zapata A. Lymphoid organs of teleost fish. I. Ultrastructure of the thymus of Rutilus rutilus. Dev Comp Immunol. (1981) 5:427–36. doi: 10.1016/S0145-305X(81)80055-9

3. Zapata A. Lymphoid organs of teleost fish. III. Splenic lymphoid tissue of Rutilus rutilus and Gobio gobio. Dev Comp Immunol. (1982) 6:87–94. doi: 10.1016/0145-305X(82)90010-6

4. Press CM, Evensen Ø. The morphology of the immune system in teleost fishes. Fish Shellfish Immunol. (1999) 9:309–18. doi: 10.1006/fsim.1998.0181

5. Bowden TJ, Cook P, Rombout JHWM. Development and function of the thymus in teleosts. Fish Shellfish Immunol. (2005) 19:413–27. doi: 10.1016/j.fsi.2005.02.003

6. Koppang EO, Fischer U, Moore L, Tranulis MA, Dijkstra JM, Köllner B, et al. Salmonid T cells assemble in the thymus, spleen and in novel interbranchial lymphoid tissue. J Anat. (2010) 217:728–39. doi: 10.1111/j.1469-7580.2010.01305.x

7. Kobayashi I, Sekiya M, Moritomo T, Ototake M, Nakanishi T. Demonstration of hematopoietic stem cells in ginbuna carp (Carassius auratus langsdorfii) kidney. Dev Comp Immunol. (2006) 30:1034–46. doi: 10.1016/j.dci.2006.01.005

8. Kobayashi I, Saito K, Moritomo T, Araki K, Takizawa F, Nakanishi T. Characterization and localization of side population (SP) cells in zebrafish kidney hematopoietic tissue. Blood. (2008) 111:1131–7. doi: 10.1182/blood-2007-08-104299

9. Rombout JH, Abelli L, Picchietti S, Scapigliati G, Kiron V. Teleost intestinal immunology. Fish Shellfish Immunol. (2011) 31:616–26. doi: 10.1016/j.fsi.2010.09.001

10. Takizawa F, Dijkstra JM, Kotterba P, Korytár T, Kock H, Köllner B, et al. The expression of CD8a discriminates distinct T cell subsets in teleost fish. Dev Comp Immunol. (2011) 35:752–63. doi: 10.1016/j.dci.2011.02.008

11. Somamoto T, Koppang EO, Fischer U. Antiviral functions of CD8(+) cytotoxic T cells in teleost fish. Dev Comp Immunol. (2014) 43:197–204. doi: 10.1016/j.dci.2013.07.014

12. Hayashi N, Takeuchi M, Nakanishi T, Hashimoto K, Dijkstra JM. Zinc-dependent binding between peptides derived from rainbow trout CD8alpha LCK. Fish Shellfish Immunol. (2010) 28:72–6. doi: 10.1016/j.fsi.2009.09.020

13. Chang YT, Kai YH, Chi SC, Song YL. Cytotoxic CD8a+ leucocytes have heterogeneous features in antigen recognition and class I MHC restriction in grouper. Fish Shellfish Immunol. (2011) 30:1283–93. doi: 10.1016/j.fsi.2011.03.018

14. Takizawa F, Magadan S, Parra D, Xu Z, Korytár T, Boudinot P, et al. Novel teleost CD4-Bearing cell populations provide insights into the evolutionary origins and primordial roles of CD4+lymphocytes and CD4+macrophages. J Immunol. (2016) 196:4522–35. doi: 10.4049/jimmunol.1600222

15. Suetake H, Araki K, Suzuki Y. Cloning, expression, and characterization of fugu CD4, the first ectothermic animal CD4. Immunogenetics. (2004) 56:368–74. doi: 10.1007/s00251-004-0694-x

16. Dijkstra JM, Somamoto T, Moore L, Hordvik I, Ototake M, Fischer U. Identification and characterization of a second CD4-like gene in teleost fish. Mol Immunol. (2006) 43:410–9. doi: 10.1016/j.molimm.2005.03.005

17. Dee CT, Nagaraju RT, Athanasiadis EI, Gray C, del Ama LF, Johnston SA, et al. CD4-transgenic zebrafish reveal tissue-resident Th2- and regulatory T cell-like populations and diverse mononuclear phagocytes. J Immunol. (2016) 197:3520–30. doi: 10.4049/jimmunol.1600959

18. Wen Y, Fang W, Xiang LX, Pan RL, Shao JZ. Identification of Treg-like cells in Tetraodon: insight into the origin of regulatory T subsets during early vertebrate evolution. Cell Mol Life Sci. (2011) 68:2615–26. doi: 10.1007/s00018-010-0574-5

19. Yamaguchi T, Takizawa F, Fischer U, Dijkstra JM. Along the axis between type 1 and type 2 immunity; principles conserved in evolution from fish to mammals. Biology (Basel). (2015) 4:814–59. doi: 10.3390/biology4040814

20. Somamoto T, Kondo M, Nakanishi T, Nakao M. Helper function of CD4+ lymphocytes in antiviral immunity in ginbuna crucian carp, Carassius auratus langsdorfii. Dev Comp Immunol. (2014) 44:111–5. doi: 10.1016/j.dci.2013.12.008

21. Taylor EB, Wilson M, Bengten E. The Src tyrosine kinase Lck binds to CD2, CD4-1, and CD4-2 T cell co-receptors in channel catfish, Ictalurus punctatus. Mol Immunol. (2015) 66:126–38. doi: 10.1016/j.molimm.2015.02.023

22. Flajnik MF. The last flag unfurled? a new immunoglobulin isotype in fish expressed in early development. Nat Immunol. (2005) 6:229–30. doi: 10.1038/ni0305-229

23. Clausen J, Vergeiner B, Enk M, Petzer AL, Gastl G, Gunsilius E. Functional significance of the activation-associated receptors CD25 and CD69 on human NK-cells and NK-like T-cells. Immunobiology. (2003) 207:85–93. doi: 10.1078/0171-2985-00219

24. DeLuca D, Wilson M, Warr GW. Lymphocyte heterogeneity in the trout, salmo gairdneri, defined with monoclonal antibodies to IgM. Eur J Immunol. (1983) 13:546–51. doi: 10.1002/eji.1830130706

25. Köllner B, Fischer U, Rombout JH, Taverne-Thiele JJ, Hansen JD. Potential involvement of rainbow trout thrombocytes in immune functions: a study using a panel of monoclonal antibodies RT-PCR. Dev Comp Immunol. (2004) 28:1049–62. doi: 10.1016/j.dci.2004.03.005
