# Supplementary material for: Ancient Cytokine Interleukin 15-Like (IL-15L) Induces a Type 2 Immune Response
Source: Front Immunol. 2020 Oct 29;11:549319. doi: 10.3389/fimmu.2020.549319 (PMC7658486; doi:10.3389/fimmu.2020.549319)

Supplementary file 4

Preparations of recombinant trout cytokines, soluble IL-15Rα (sIL-15Rα) and RLI fusion proteins from insect cells

Table of Contents **Page**

4A: Co-purification by anti-FLAG agarose reveals binding of trout IL-15 to trout sIL-15Rα 2

4B: Co-purification by anti-FLAG agarose reveals binding of trout IL-15La to trout sIL-15Rα 3

4C: Co-purification by anti-FLAG agarose reveals binding of trout IL-2 to trout sIL-15Rα 4

4D: Analysis of purified recombinant trout IL-15-RLI 5

4E: Analysis of purified recombinant trout IL-15La-RLI 8

4F: Analysis of purified recombinant trout IL-2 10

Supplementary file 4A Co-purification by anti-FLAG agarose reveals binding of trout IL-15 to trout sIL-15Rα.

Approximately 100 ml of supernatant each of insect cells infected with recombinant baculovirus(es) for inducing expression of FLAG-tagged trout IL-15 and/or Myc-tagged trout sIL-15Rα (IL-15, sIL-15Rα, and IL-15+sIL-15Rα) were purified using agarose-bound anti-FLAG and chromatography columns. The flow-through sample from the column was collected ("Flow-through"). After washing, elution and a final buffer exchange for 300 µl PBS, the purified product was obtained ("Eluted"). Of both the Eluted and Flow-through samples 15 µl per lane was analyzed by SDS-PAGE followed by anti-FLAG (a) and anti-Myc (b) Western blot (WB) analyses. M, size marker. The results for (a) and (b) were obtained using separate gels.

The results show that, in case alone, sIL-15Rα could not be retained and subsequently eluted using the anti-FLAG agarose [see lane sIL-15Rα-Eluted in (b)], whereas IL-15 was readily detectable in the Eluted fraction [see lane IL-15-Eluted in (a)]. In contrast, from the supernatants in which IL-15 and sIL-15Rα were present together (IL-15+sIL-15Rα), also sIL-15Rα was readily detectable in the Eluted fraction [see lane IL-15+sIL-15Rα-Eluted in (b)]. The results provide evidence for binding between trout IL-15 and IL-15Rα.

(a) *anti-FLAG WB for detection of IL-15*

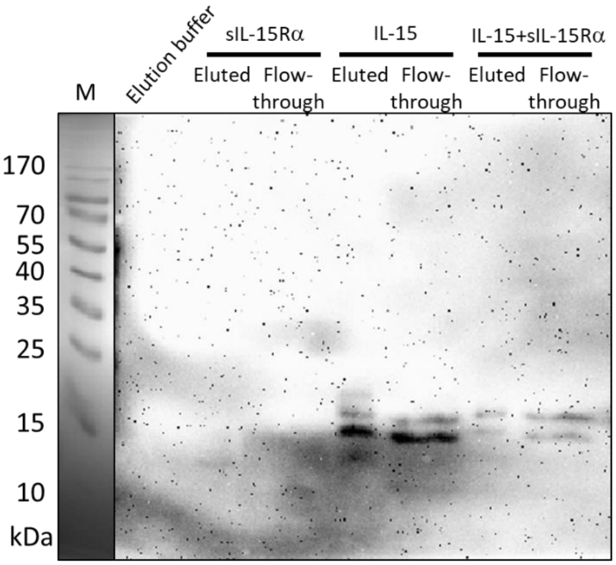

(b) *anti-Myc WB for detection of sIL-15Rα*

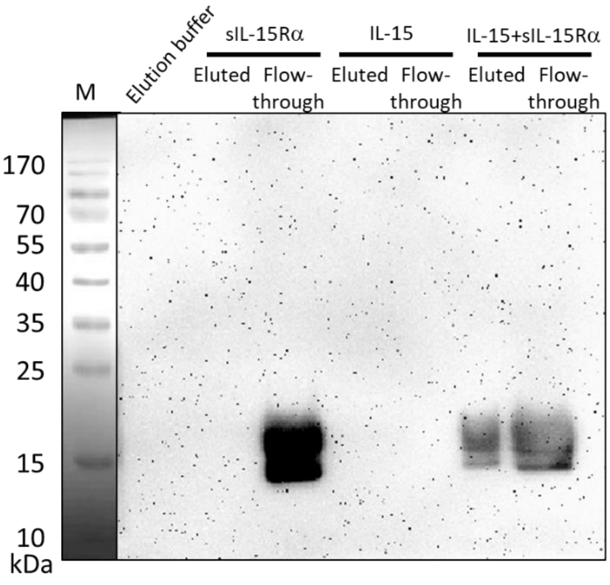

Supplementary file 4B Co-purification by anti-FLAG agarose reveals binding of trout IL-15La to trout sIL-15Rα.

Approximately 100 ml of supernatant of insect cells infected with recombinant baculoviruses for inducing expression of FLAG-tagged trout IL-15La plus Myc-tagged trout sIL-15Rα was purified using agarose-bound anti-FLAG and chromatography columns. The flow-through sample from the column was collected (“Flow-through”). After washing, elution and a final buffer exchange for 300 µl PBS, the purified product was obtained (“Eluted”). Of both the Eluted and Flow-through samples 15 µl per lane was analyzed by SDS-PAGE followed by non-specific protein staining using Coomassie blue (a), as well as by anti-FLAG (b) and anti-Myc (c) Western blot (WB) analyses. M, size marker. The results for (a), (b) and (c) were obtained using separate gels.

The results indicate that both IL-15La and sIL-15Rα were efficiently eluted. Because, under similar conditions, from supernatants in which sIL-15Rα was present alone the sIL-15Rα protein was not detectably eluted [see Supplementary file 4A(b)], the results provide evidence for binding between trout IL-15La and IL-15Rα. The higher weight band (or bands) detected with anti-FLAG WB might be a homodimer form (or forms) of IL-15La.

(a) *Coomassie blue staining*

(b) *anti-FLAG WB for detection of IL-15La*

(c) *anti-Myc WB for detection of sIL-15Rα*

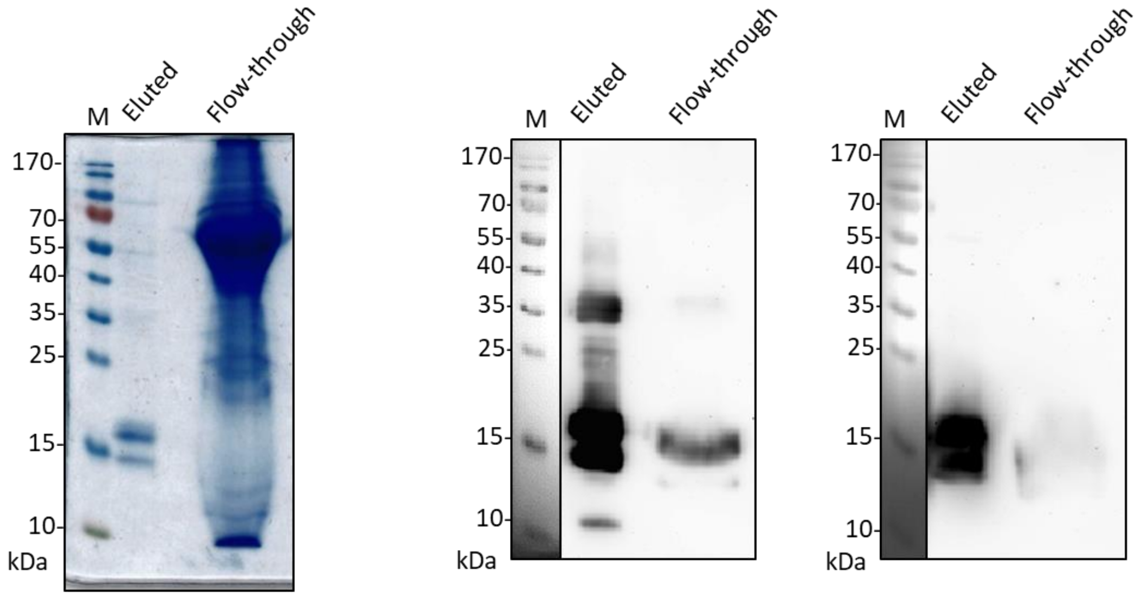

Supplementary file 4C Co-purification by anti-FLAG agarose reveals binding of trout IL-2 to trout sIL-15Rα.

Approximately 100 ml of supernatant of insect cells infected with recombinant baculoviruses for inducing expression of FLAG-tagged trout IL-2 plus Myc-tagged trout sIL-15Rα was purified using agarose-bound anti-FLAG and chromatography columns. The flow-through sample from the column was collected ("Flow-through"). After washing, elution and a final buffer exchange for 300 µl PBS, the purified product was obtained ("Eluted"). Of both the Eluted and Flow-through samples 15 µl per lane was analyzed by SDS-PAGE followed by non-specific protein staining using Coomassie blue (a), and anti-FLAG (b) and anti-Myc (c) Western blot (WB) analyses. M, size marker. The results for (a), (b) and (c) were obtained using separate gels.

The results indicate that both IL-2 and sIL-15Rα were efficiently eluted. Because, under similar conditions, from supernatants in which sIL-15Rα was present alone the sIL-15Rα protein was not detectably eluted [see Supplementary file 4A(b)], the results provide evidence for binding between trout IL-2 and IL-15Rα. The higher weight band (or bands) detected with anti-FLAG WB might be a homodimer form (or forms) of IL-2, and/or maybe a complex of IL-2 and sIL-15Rα.

(a) *Coomassie blue staining* (b) *anti-FLAG WB for detection of IL-2* (c) *anti-Myc WB for detection of sIL-15Rα*

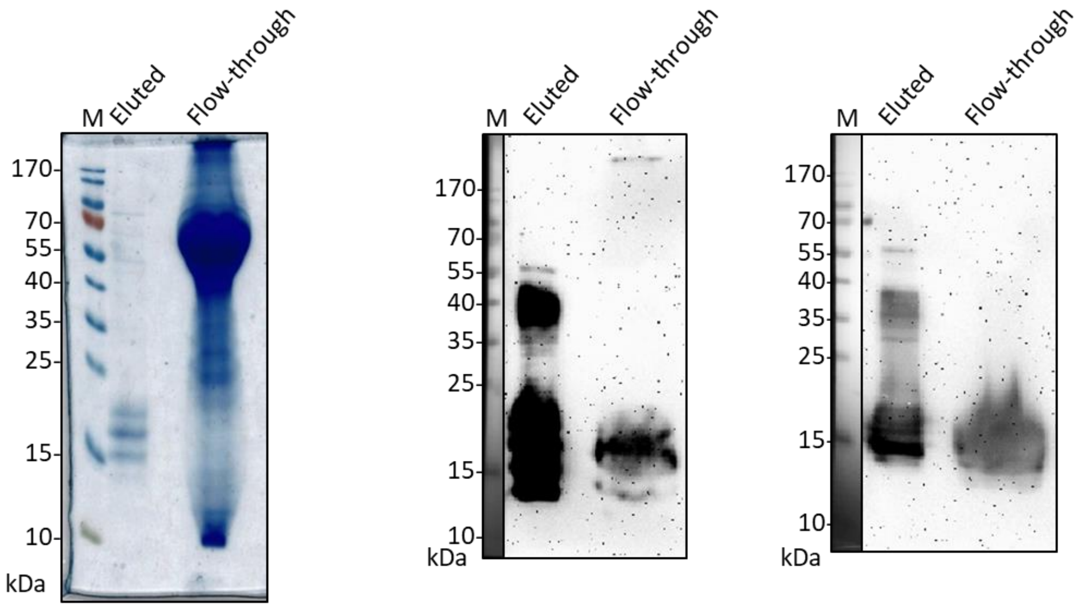

Supplementary file 4D Analysis of purified recombinant trout IL-15-RLI.

Approximately 300 ml of supernatant of insect cells infected with recombinant baculovirus for inducing expression of FLAG-tagged trout IL-15-RLI (a fusion protein of trout IL-15 and trout sIL-15Rα) was purified using agarose-bound anti-FLAG and chromatography columns. After washing, elution and a final buffer exchange for 300 μl PBS, the purified product was obtained ("Purified IL-15-RLI"). Part of this product was mixed with glycerol and BSA and stored at -20 °C for later use in functional assays, while another part was investigated for preparation quality by size exclusion chromatography (aka "gel filtration") [(a), with (b) for estimating the protein size corresponding with the major peak F2 in (a)], and by SDS-PAGE followed by non-specific protein staining using Coomassie blue (c) and anti-FLAG Western blot analysis (d). Not only the "Purified IL-15-RLI" sample was analyzed by Coomassie blue staining and Western blot analysis, but also the fractions F1, F2 and F3 which were eluted using gel filtration (a) and concentrated before loading onto gels for SDS-PAGE [(c) and (d)]. (b) Molecular weights of the purified recombinant proteins were estimated by gel filtration. The red dot represents the F2 peak shown in (a) and blue dots represent the following standard proteins: bovine serum albumin, 66 kDa; carbonic anhydrase, 29 kDa; cytochrome c, 12.4 kDa; aprotinin, 6.5 kDa. (e) N-glycosylation of proteins in the purified IL-15-RLI preparation was shown by anti-FLAG Western blot analysis of samples of this preparation that were not treated [Purified IL-15-RLI], subjected to treatment with PNGaseF [Purified IL-15-RLI (+)], or subjected to the corresponding mock treatment [Purified IL-15-RLI (-)] (e). For the SDS-PAGE analyses in (c) and (d), PBS was loaded as negative control [(c) and (d)]. M, size marker. The results for (c) and (d) were obtained using separate gels and loading 15 μl sample per lane, and the results for (e) were obtained by loading amounts corresponding to 1.2 μl (~160 ng) of the purified IL-15-RLI sample per lane.

The results showed that the purified IL-15-RLI preparation was rather pure [see the "Purified IL-15-RLI" lane in (c)]. The bands with an apparent molecular weight of ~35 kDa observed upon SDS-PAGE analysis [(c), (d) and (e)] can be concluded to include N-glycosylated (e) monomeric proteins, considering that the predicted molecular weight of the protein backbone (without leader sequence) is 26.4 kDa. Upon gel filtration the bulk of the protein behaved as having an approximate molecular weight of 42.6 kDa [(a) and (b)] which agrees best with the assumption that the bulk of the protein is present as a soluble monomer. Upon sensitive Western blot analysis, a small band of ~13 kDa was detected which may represent a breakdown product.

(Supplementary file 4D)

(a) *Gel filtration analysis of purified IL-15-RLI*

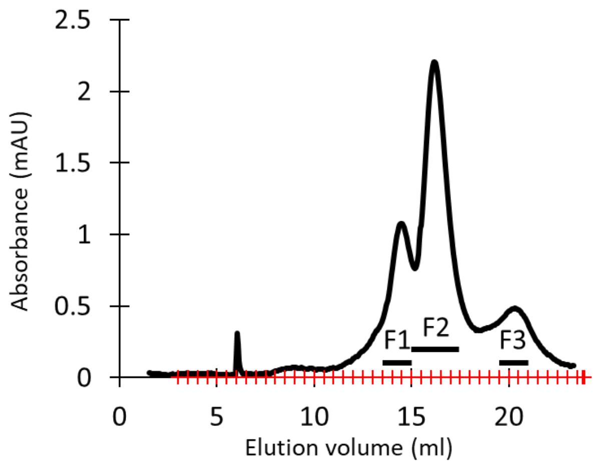

(b) *Gel filtration analysis for estimating the size of proteins in the F2 peak in (a)*

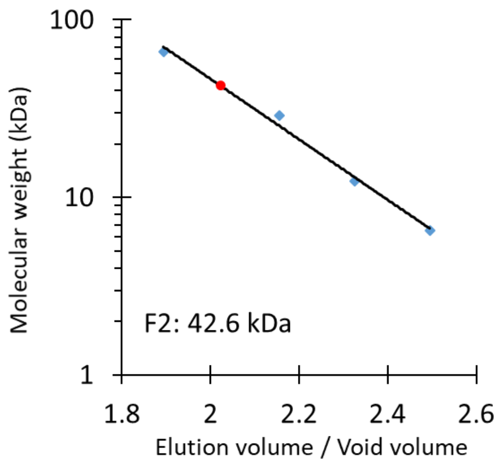

(c) *Coomassie blue staining*

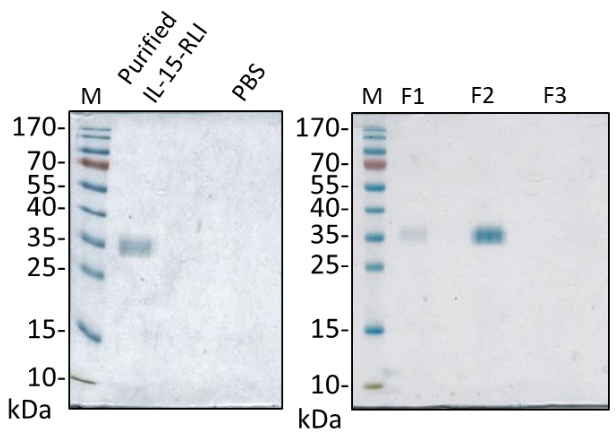

(d) *Anti-FLAG Western blot analysis*

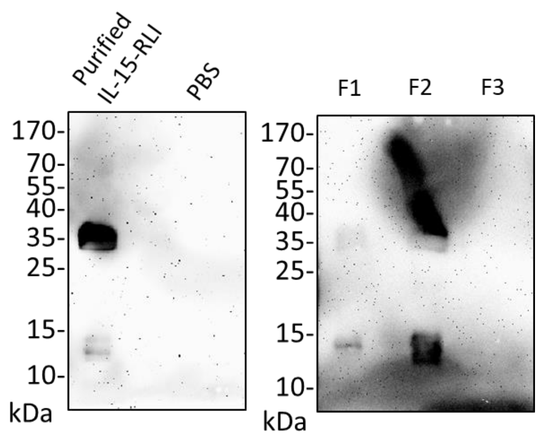

(Supplementary file 4D)

(e) *Analysis of N-glycosylation by PNGaseF treatment*

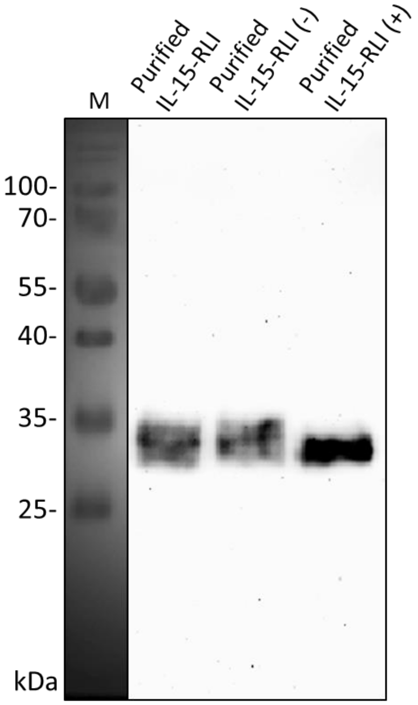

Supplementary file 4E Analysis of purified recombinant trout IL-15La-RLI.

Isolation from insect cells and subsequent analysis of recombinant FLAG-tagged IL-15La-RLI (a fusion product of trout IL-15La and trout sIL-15Ra) was performed as done for IL-15-RLI (see the legend of Supplementary file 4D). The results are shown in figures (a)-to-(e).

The results showed that the purified IL-15La-RLI preparation was rather pure [see the "Purified IL-15La-RLI" lane in (c)]. The bands with an apparent molecular weight of ~35 kDa observed upon SDS-PAGE analysis [(c), (d) and (e)] can be concluded to include N-glycosylated (e) monomeric proteins, considering that the predicted molecular weight of the protein backbone (without leader sequence) is 26.0 kDa. Upon gel filtration the bulk of the protein behaved as having an approximate molecular weight of 31.4 kDa [(a) and (b)] which agrees best with the assumption that the bulk of the protein is present as a soluble monomer. Upon sensitive Western blot analysis, a band of ~70 kDa was detected in the purified IL-15La-RLI preparation which may represent a homodimer (d). Western blot analysis of the F1 fraction isolated by gel filtration detected a small band of ~17 kDa which may represent a breakdown product resulting from treatment (d).

(a) *Gel filtration analysis of purified IL-15La-RLI*

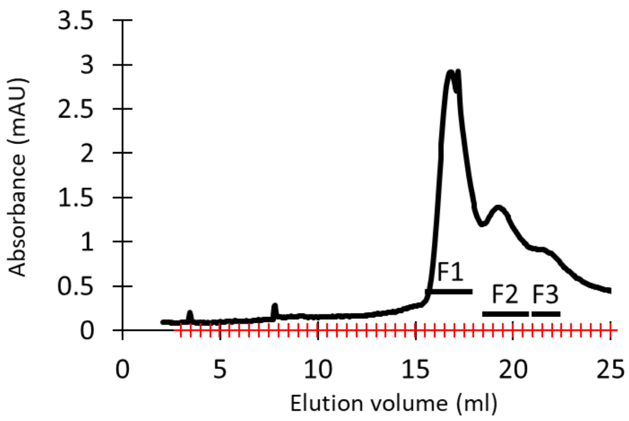

(b) *Gel filtration analysis for estimating the size of proteins in the F1 peak in (a)*

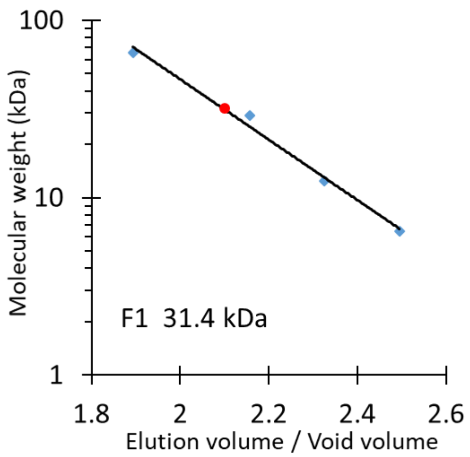

(Supplementary file 4E)

(c) *Coomassie blue staining*

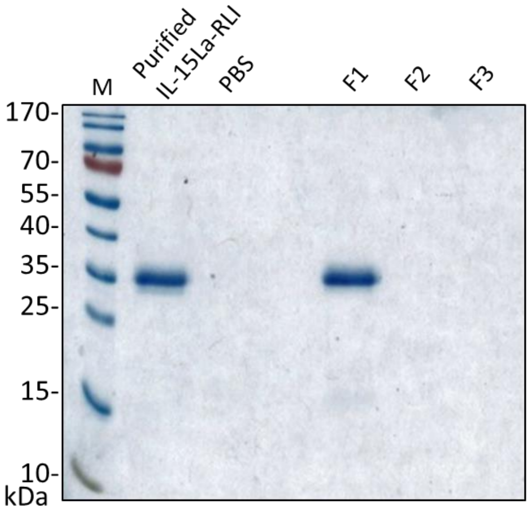

(d) *Anti-FLAG Western blot analysis*

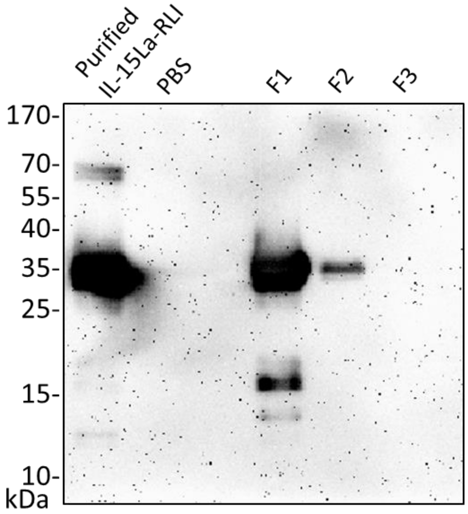

(e) *Analysis of N-glycosylation by PNGaseF treatment*

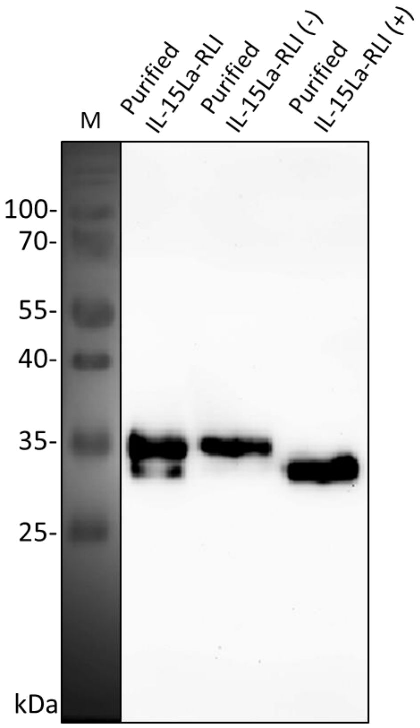

Supplementary file 4F Analysis of purified recombinant trout IL-2.

Isolation from insect cells and subsequent analysis of recombinant FLAG-tagged trout IL-2 was performed as done for IL-15-RLI (see the legend of Supplementary file 4D). The results of those analyses are shown in figures (a)-to-(e).

The results showed that the purified IL-2 preparation was rather pure [see the "Purified IL-2" lane in (c)]. The four major bands in the purified IL-2 preparation with an apparent molecular weight of approximately 13-19 kDa that were observed upon SDS-PAGE analysis [(c) and (d)] may include different levels of glycosylation of monomeric proteins, since treatment with PNGaseF affected the running behavior of at least the two biggest protein among these bands (e) and the predicted molecular weight of the protein backbone (without leader sequence) is 13.2 kDa. We do not understand all four bands in the 13-19 kDa range, and they may also represent other modifications besides N-glycosylation and/or breakdown products. Upon gel filtration the bulk of the protein behaved as having an approximate molecular weight of 31.4 kDa [(a) and (b); (c) shows that peak F4 contained most protein] which agrees best with the assumption that the bulk of the protein is present in soluble homodimer form. Upon sensitive Western blot analysis, a band of ~40 kDa was detected in the purified IL-2 preparation which may represent a homodimer form (d).

(a) *Gel filtration analysis of purified IL-2*

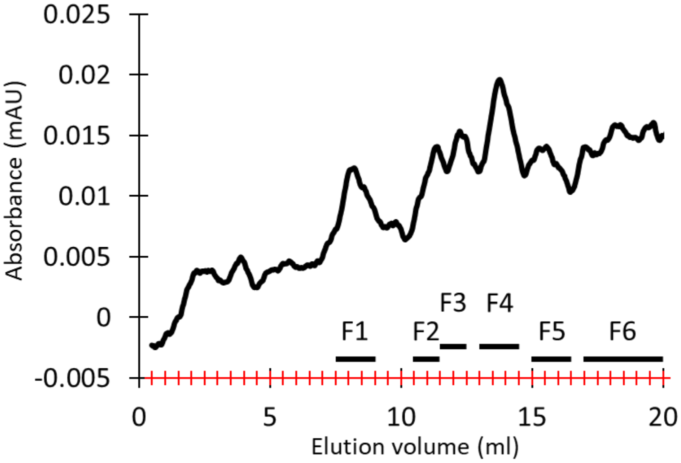

(b) *Gel filtration analysis for estimating the size of proteins in the F3 and F4 peaks (a)*

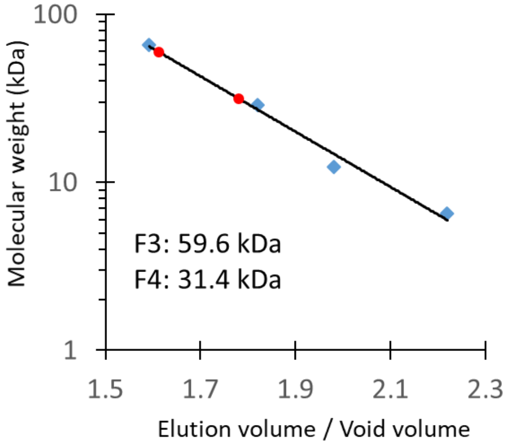

(Supplementary file 4F)

(c) *Coomassie blue staining*

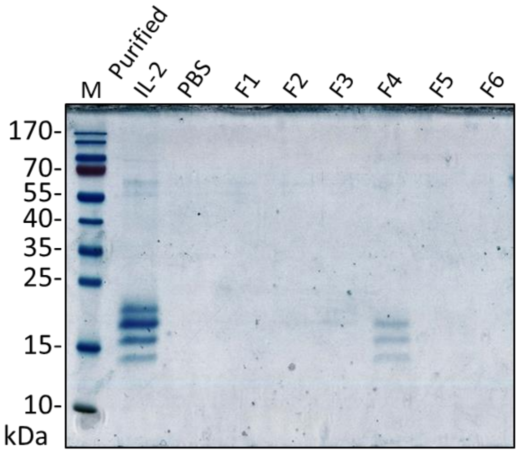

(d) *Anti-FLAG Western blot analysis*

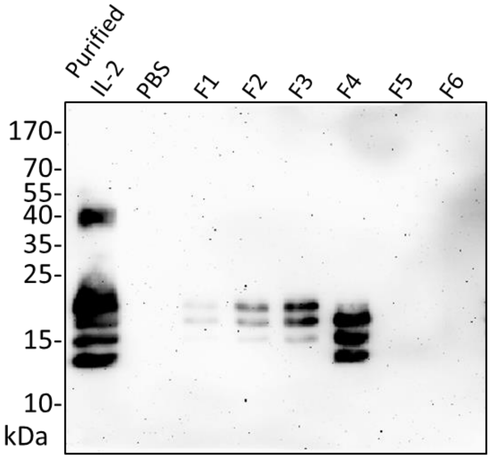

(e) *Analysis of N-glycosylation by PNGaseF treatment*

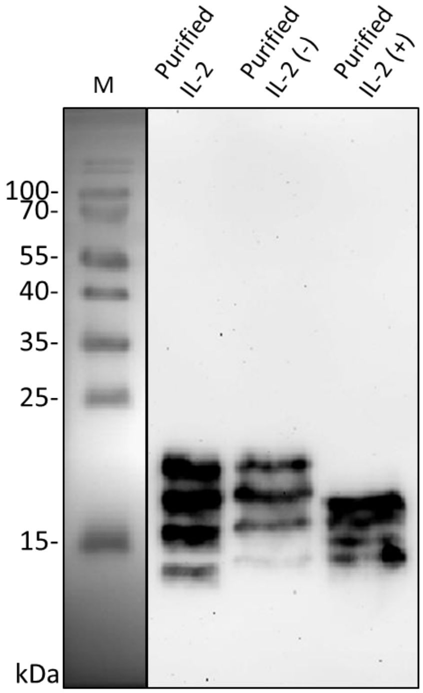

Supplement: Supplementary file 4 [file Data_Sheet_4.PDF]
