# Supplementary material for: Ancient Cytokine Interleukin 15-Like (IL-15L) Induces a Type 2 Immune Response
Source: Front Immunol. 2020 Oct 29;11:549319. doi: 10.3389/fimmu.2020.549319 (PMC7658486; doi:10.3389/fimmu.2020.549319)

Supplementary file 5

Additional Western blot results

Table of Contents **Page**

5A: Cytokine deglycosylation analyses *(extended version of main text Fig. 4)* 3

5B: Secretion of recombinant cytokines and alpha receptor chains from transfected HEK293T cells (*information supporting the main text Fig. 6 results*) 5

5C: Phosphorylation of STAT5 in various trout lymphocyte populations induced after incubation with recombinant trout cytokine containing HEK293T supernatants (*information supporting the main text Fig. 7 results*) 11

5D: Phosphorylation of STAT5 in DN, DP, CD4SP and CD8SP fractions of trout thymocytes induced after incubation with recombinant cytokine containing HEK293T supernatants (*information supporting the main text Fig. 8 results*) 23

5E: Phosphorylation of STAT5 in lymphocyte fractions of trout thymus, intestine and spleen, induced after incubation with purified recombinant trout cytokines (produced in insect cells) IL-2, IL-15-RLI and IL-15La-RLI at 5, 25 and 125 nM (*information supporting the main text Fig. 9 results*) 26

5F: Titration of the recombinant trout cytokine (produced in insect cells) concentration necessary for the induction of pSTAT5 29

Supplementary file 5A. Cytokine deglycosylation analyses *(extended version of main text Fig. 4)*.

Bovine IL-2 and IL-15, and trout IL-2, IL-15 and IL-15La, can be N-glycosylated.

Lysates of HEK293T cells that were transfected with DNA plasmids encoding FLAG-tagged cytokines were incubated overnight in digestion buffer (+) or not (-), and the incubated samples were digested with PNGase-F (+) or not (-). After that, the samples were analyzed by anti-FLAG Western blotting. Figures (a) and (b) show uncropped versions of the blot results of which parts of the lanes for the PNGase-F treated and mock treated samples are shown in main text Fig. 4; in addition to the lanes shown in Fig. 4, results for a PNGase-F treated untransfected HEK293T lysate control (the left lanes) and a transfected but untreated control (the second lanes from the left) are included. (a) Bovine IL-2 and IL-15, but not bovine IL-15L, show a shift in apparent molecular weight consistent with removal of N-linked sugar chains. (b) Trout IL-2, IL-15, and IL-15La, but not trout IL-15Lb, show a shift in apparent molecular weight consistent with removal of N-linked sugar chains. Some of the bands were also sensitive to mock treatment alone (e.g. the largest bovine IL-2 band, the smallest trout IL-15 band, and the trout IL-15Lb band), possibly because of aggregation. For the untreated sample of trout IL-15La expressing HEK293T cells, a band of ~37 kDa is observed which might represent an IL-15La homodimer.

(Supplementary file 5A)

(a) *Bovine IL-2 and IL-15 can be N-glycosylated.*

anti-FLAG Western blots

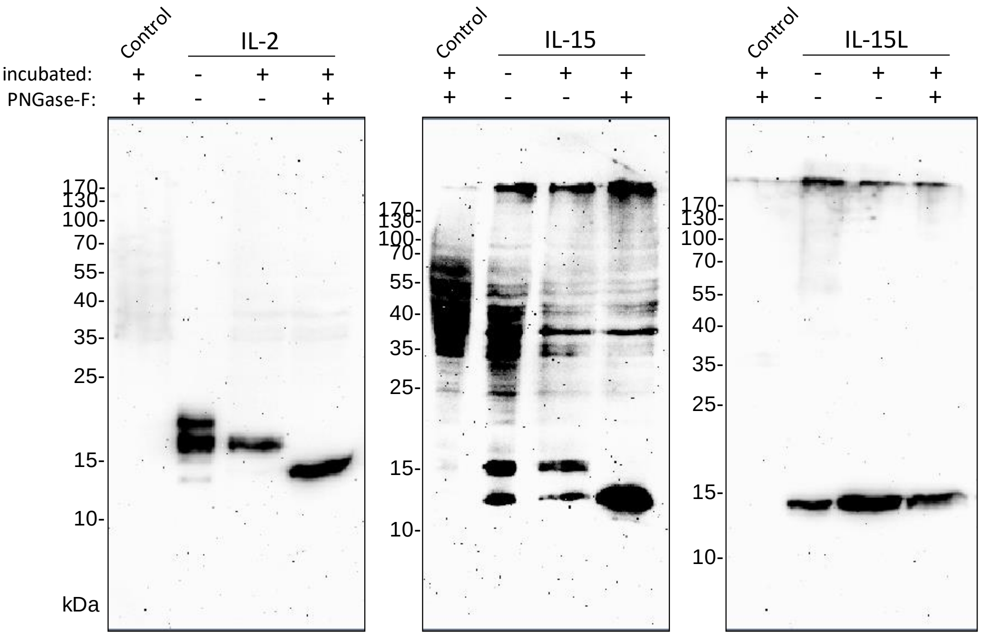

(b) *Trout IL-2, IL-15 and IL-15La can be N-glycosylated.*

anti-FLAG Western blots

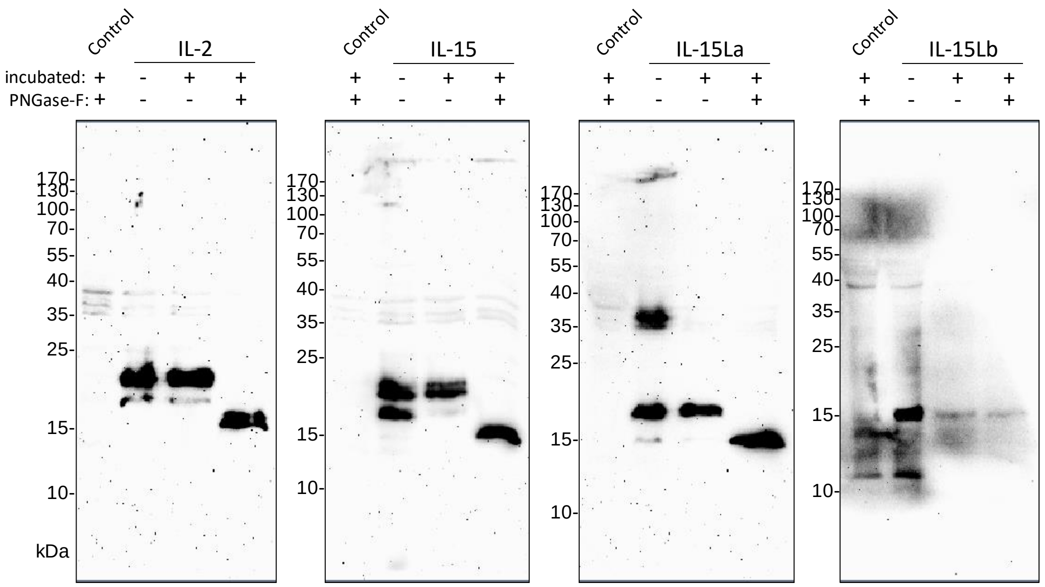

Supplementary file 5B. Secretion of recombinant cytokines and alpha receptor chains from transfected HEK293T cells *(information supporting the main text Fig. 6 results)*.

Co-expression with soluble IL-15Rα causes enhanced presence of bovine and trout IL-15 and IL-15L in the supernatant of transfected cells.

HEK293T cells were transfected with DNA plasmids for expression of FLAG-tagged cytokines and/or species-specific (bovine in the bovine panel, trout in the trout panel) Myc-tagged soluble IL-2Rα or IL-15Rα. Matching supernatants (upper blots; the supernatants were concentrated before loading) and cell lysates (lower blots) were compared by Western blot analyses using antibodies against FLAG (left blots) or Myc (right blots). The data shown are from two independent experiments for expression of the bovine molecules (Cattle experiments 1 and 2), and three independent experiments for expression of the trout molecules (Trout experiments 1, 2 and 3). Cropped versions of the anti-FLAG results of Cattle experiment 1 and Trout experiment 1 are shown in main text Fig. 6; a reason for the cropping was because the uncropped blot results include data for RLI protein versions in which modified bovine IL-15L or trout IL-15La were linked with human sIL-15Rα (bov.IL-15Lhyb-h-RLI and IL-15La-h-RLI). In our experiments we first used a combination of human sIL-15Rα and trout IL-15La in order to more precisely copy the previously published all-human RLI version (5), and only after we had found stability and function this fusion version (e.g. Figures (c) and (d), and main text Fig. 8) we progressed to making a fusion between trout IL-15La and trout sIL-15Rα which was produced in insect cells (IL-15La-RLI used for the experiments resulting in main text Figs. 9-11) We deleted the RLI-form results from the blot pictures shown in main text Fig. 6 because the explanation would have needed too much text space and might be confusing to readers. For more explanation of the bovine IL-15L modification in the bov.IL-15Lhyb-h-RLI sequence see Supplementary file 2. In the cell lysates with trout IL-15La a band of ~37 kDa is observed which might represent an IL-15La homodimer [(c), (d) and (e)].

(Supplementary file 5B)

(a) *Cattle, experiment 1*

Western blots

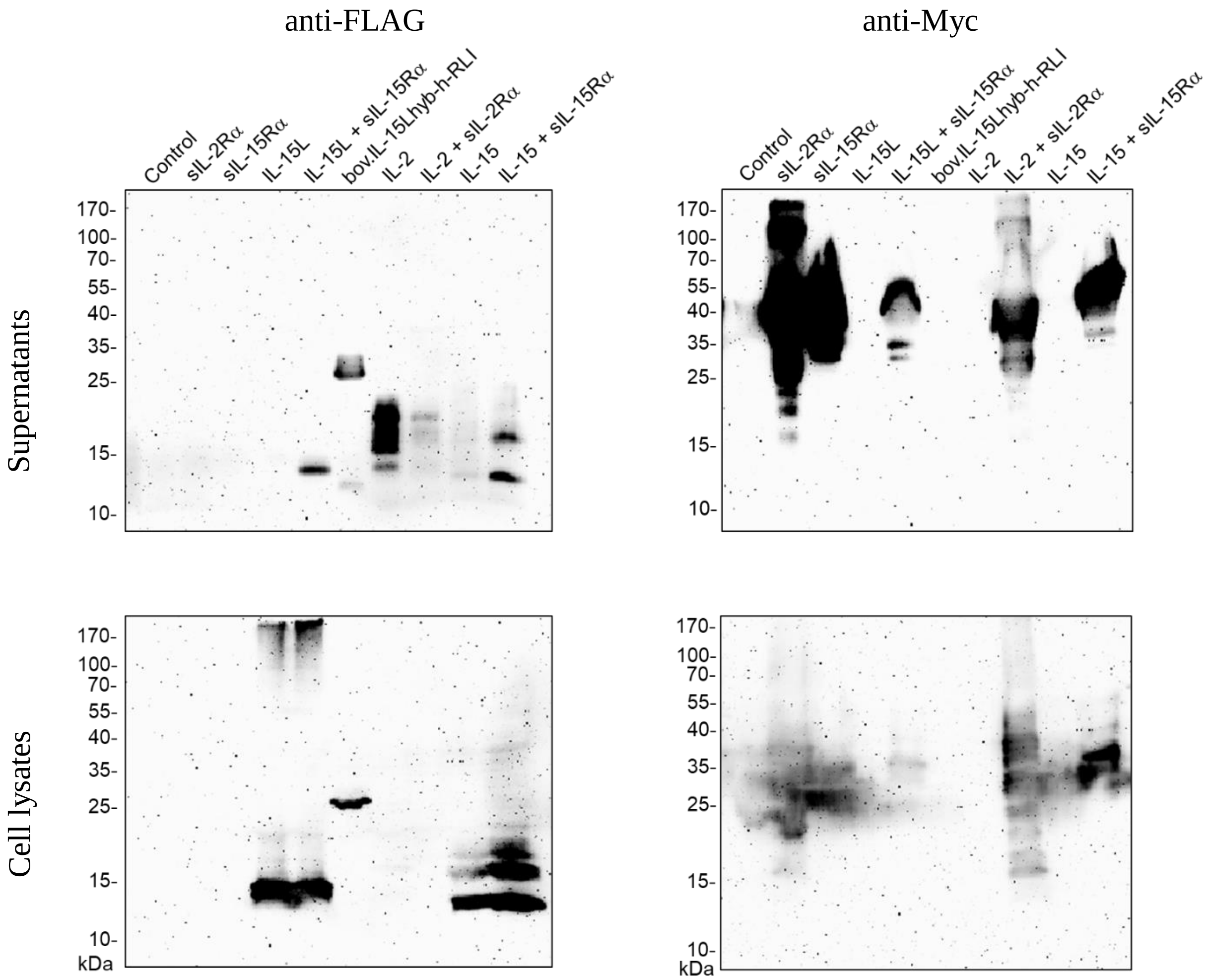

(Supplementary file 5B)

(b) *Cattle, experiment 2*

Western blots

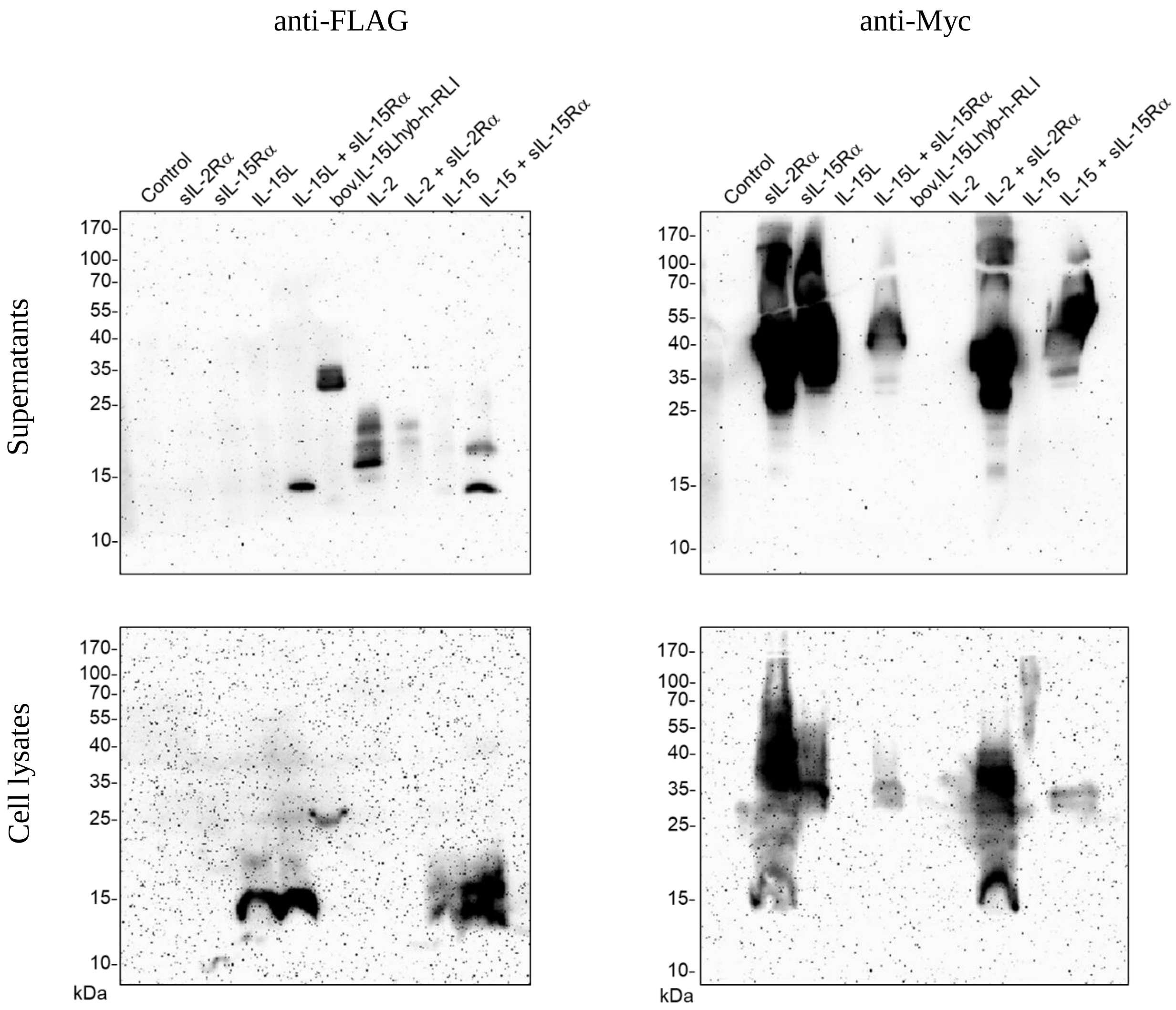

(Supplementary file 5B)

(c) *Trout, experiment 1*

Western blots

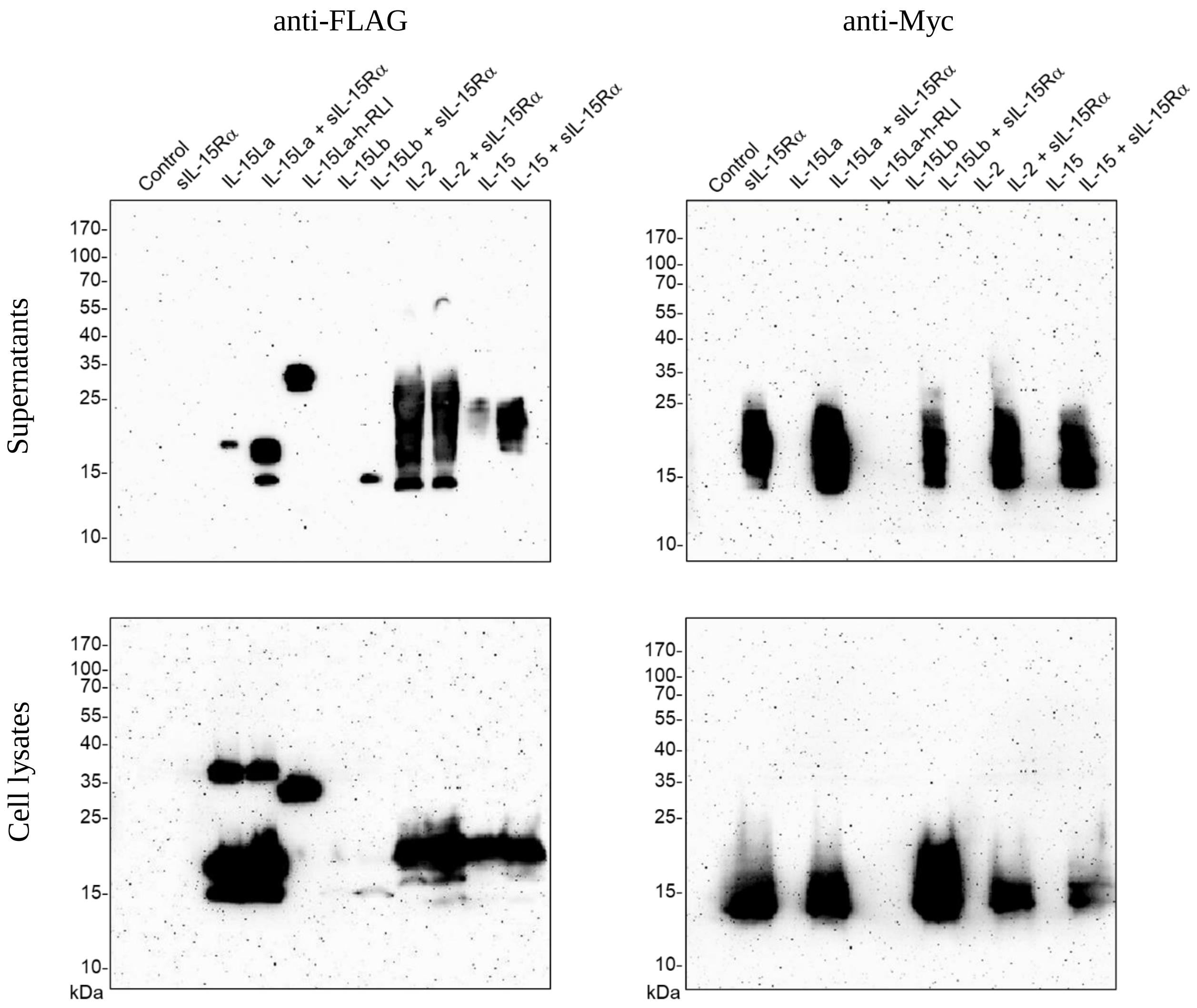

(Supplementary file 5B)

(d) *Trout, experiment 2*

Western blots

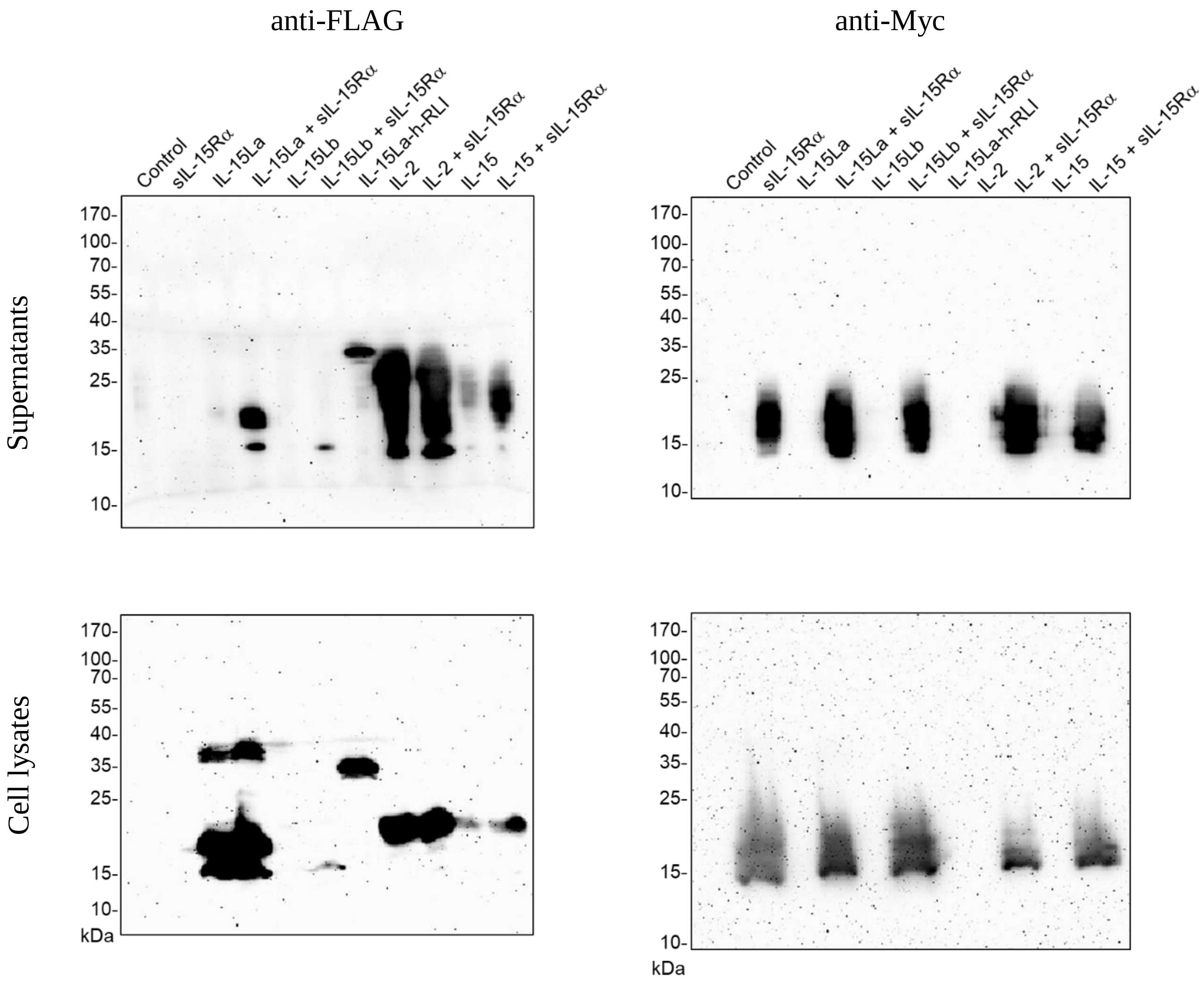

(Supplementary file 5B)

(e) *Trout, experiment 3*

Western blots

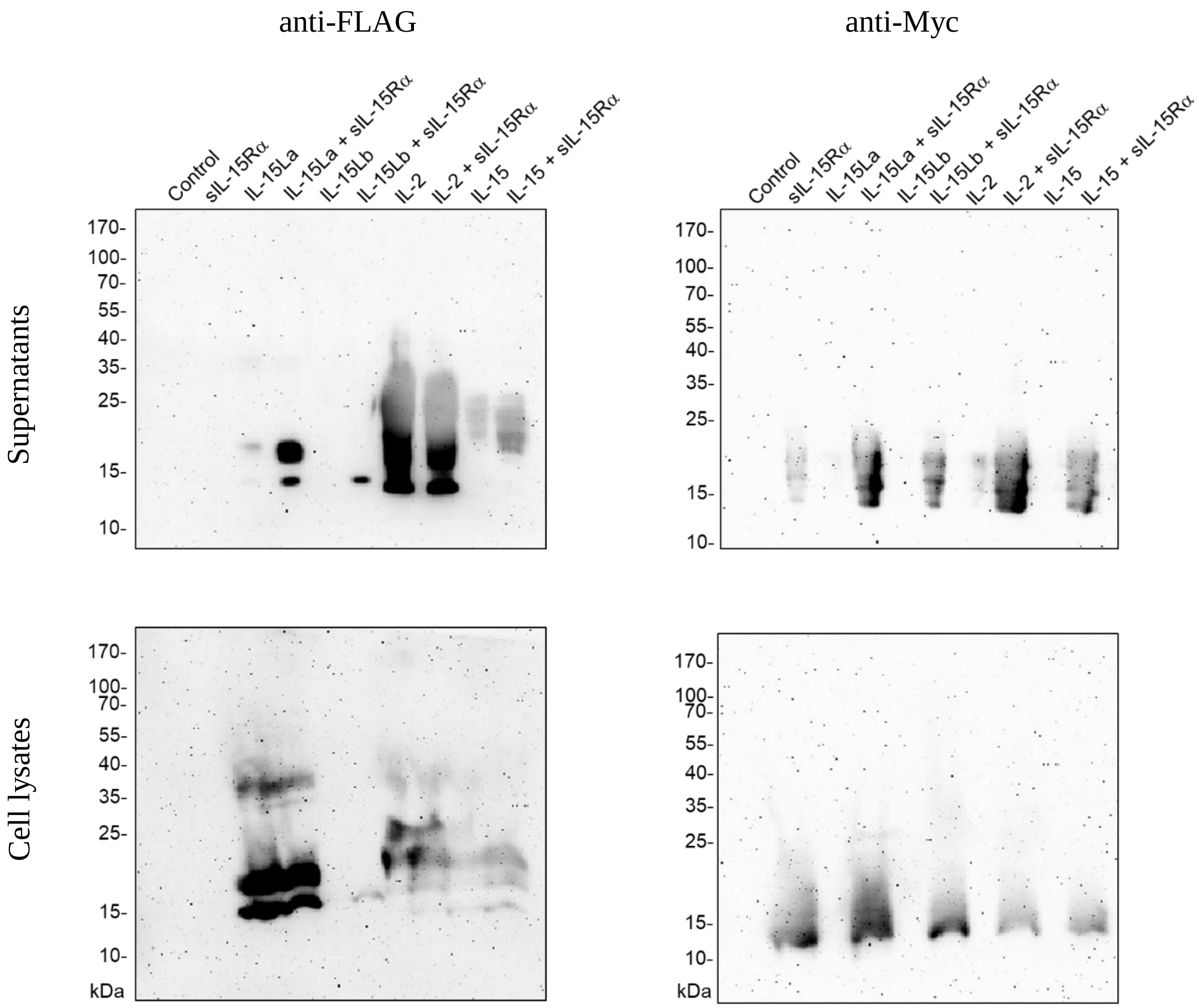

Supplementary file 5C. Phosphorylation of STAT5 in various trout lymphocyte populations induced after incubation with recombinant trout cytokine containing HEK293T supernatants *(information supporting the main text Fig. 7 results)*.

Western blot analyses of phosphorylated STAT5 (pSTAT5) in CD8α^+ and CD8α^- fractions of trout morphological lymphocytes that had been isolated from several tissues using flow sorting (for examples see Supplementary file 3) and had been incubated with recombinant trout cytokine containing supernatants from transfected HEK293T cells.

The results shown in this Supplementary file show uncropped versions of the Western blot results shown in main text Fig. 7, as well as confirmation of those results by independent experiments. In addition, in many of the experiments the HEK293T supernatants used for stimulation were investigated for the presence of recombinant cytokines by anti-FLAG Western blot analysis.

Lymphocytes were incubated for 15 min at 15 °C with the supernatants of HEK293T cells transfected for trout IL-2, IL-15 or IL-15La, with (+) or without (-) trout sIL-15Rα. In most cases FLAG-tagged cytokines were used, but in some instances also cytokines without an added tag were used [IL-2(N), IL-15 (N), IL-15La(N) and IL-15Lb(N)]; furthermore, in some experiments an RLI fusion product was used which consisted of a fusion between human sIL-15Rα and trout IL-15La (IL-15La-h-RLI; for explanation see the Supplementary file 5B legend). Treatment controls consisted of supernatants of HEK293T cells transfected with empty vector (Control), or with vector for trout sIL-15Rα alone (sIL-15Rα). Independent experiments for lymphocytes of the same tissue pooled from other trout individuals are numbered #1 etc. Results are shown for: Intestine#1 (a), Intestine#2 (b), Gill#1 (c), Gill#2 (d), Spleen#1 (e), Spleen#2 (f), Head kidney#1 (g), Head kidney#2 (h), Thymus#1 (i), Thymus#2 (j) and Thymus#3 (k). (figure parts 1, e.g. a-1) After stimulation, lysates were subjected to Western blot analysis using antibodies against phosphorylated STAT5. After stripping and washing, the same blots were analyzed by Western blot analysis using anti-actin as a loading control. (figure parts 2, e.g. a-2) In many but not all instances the (unconcentrated) HEK293T supernatants used for stimulation were subjected to anti-FLAG Western blot analysis; because the proteins were not concentrated first, the cytokine concentrations were often too low for allowing detection. However, a relevant finding was that in the investigated supernatants the IL-2 concentrations were higher than those of IL-15, concluding that in cases where IL-15 showed higher activity than IL-2 that could not be due to lower concentrations of IL-2. In some instances, more than one pooled tissue was analyzed for the same group of trout individuals using the same HEK293T supernatants (Spleen#1 and Gill#2; Intestine#1 and Head kidney#1; Gill#1 and Thymus#2); in such cases, for convenience of the reader, the Western blots of the HEK293T supernatants are repeatedly shown in the relevant figures. The experiments of which cropped blot results are shown in main text Fig. 7 are: Intestine#1, Gill#1, Spleen#1, Head kidney#1, and Thymus#1.

As exceptions, for the Spleen#2 and Thymus#3 experiments [(f) and (j)] cells were not only separated by using anti-CD8 but also by anti-CD4. For the Thymus#3 experiment only the stimulation of CD4$^-$ CD8$^-$ (DN) thymocytes was investigated.

(Supplementary file 5C)

(a) *Intestine#1*

Western blots

(a-1) *Results of the lymphocyte stimulation experiments*

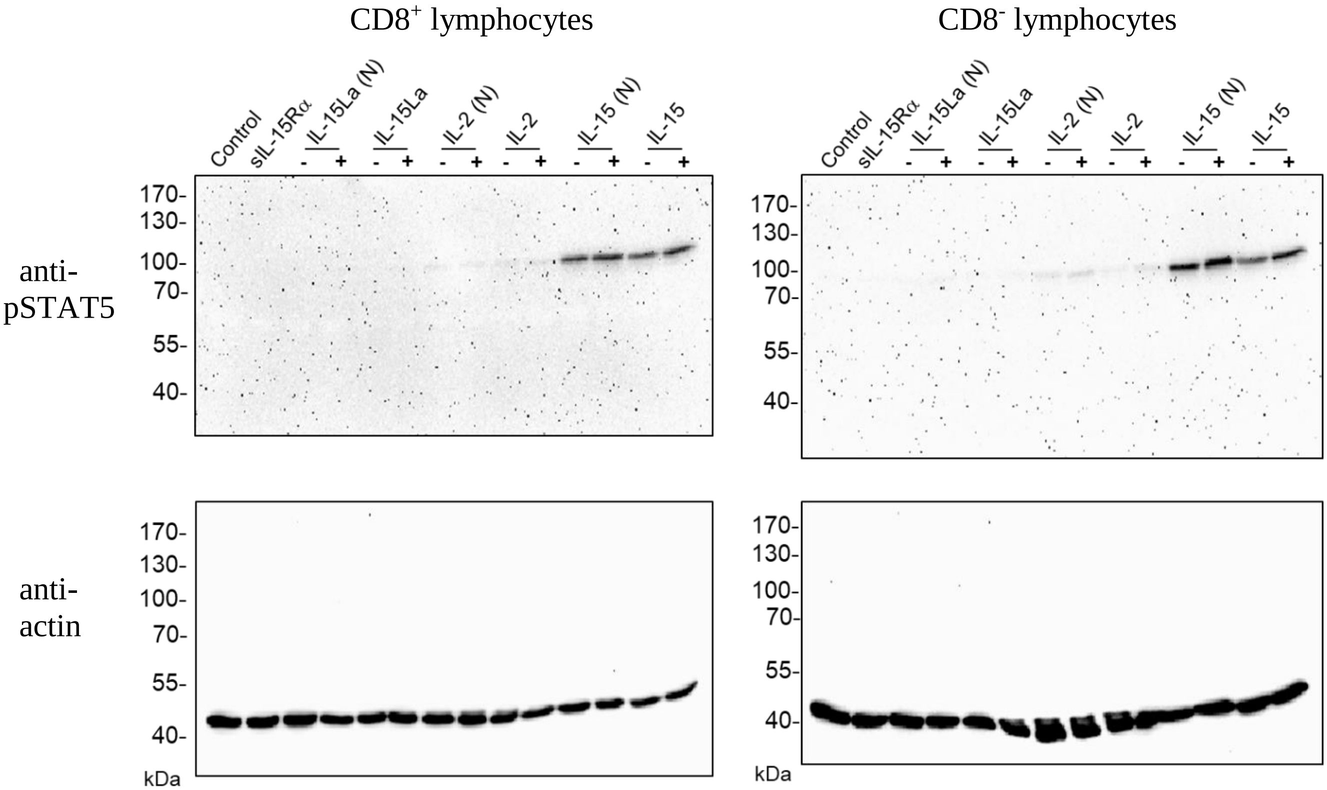

(a-2) *anti-FLAG Western blot of the HEK293T supernatants used for the stimulation*

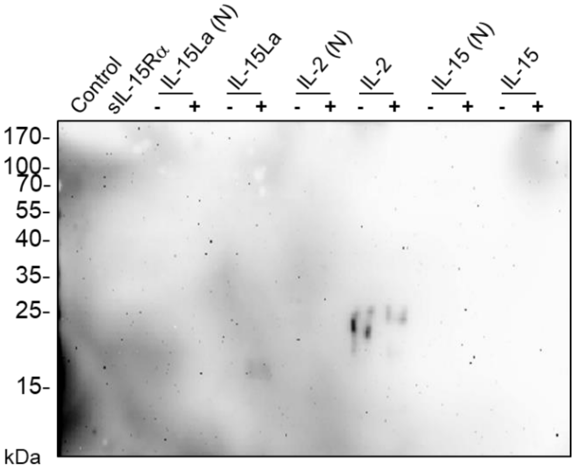

(Supplementary file 5C)

(b) *Intestine#2*

Western blots

(b-1) *Results of the lymphocyte stimulation experiments*

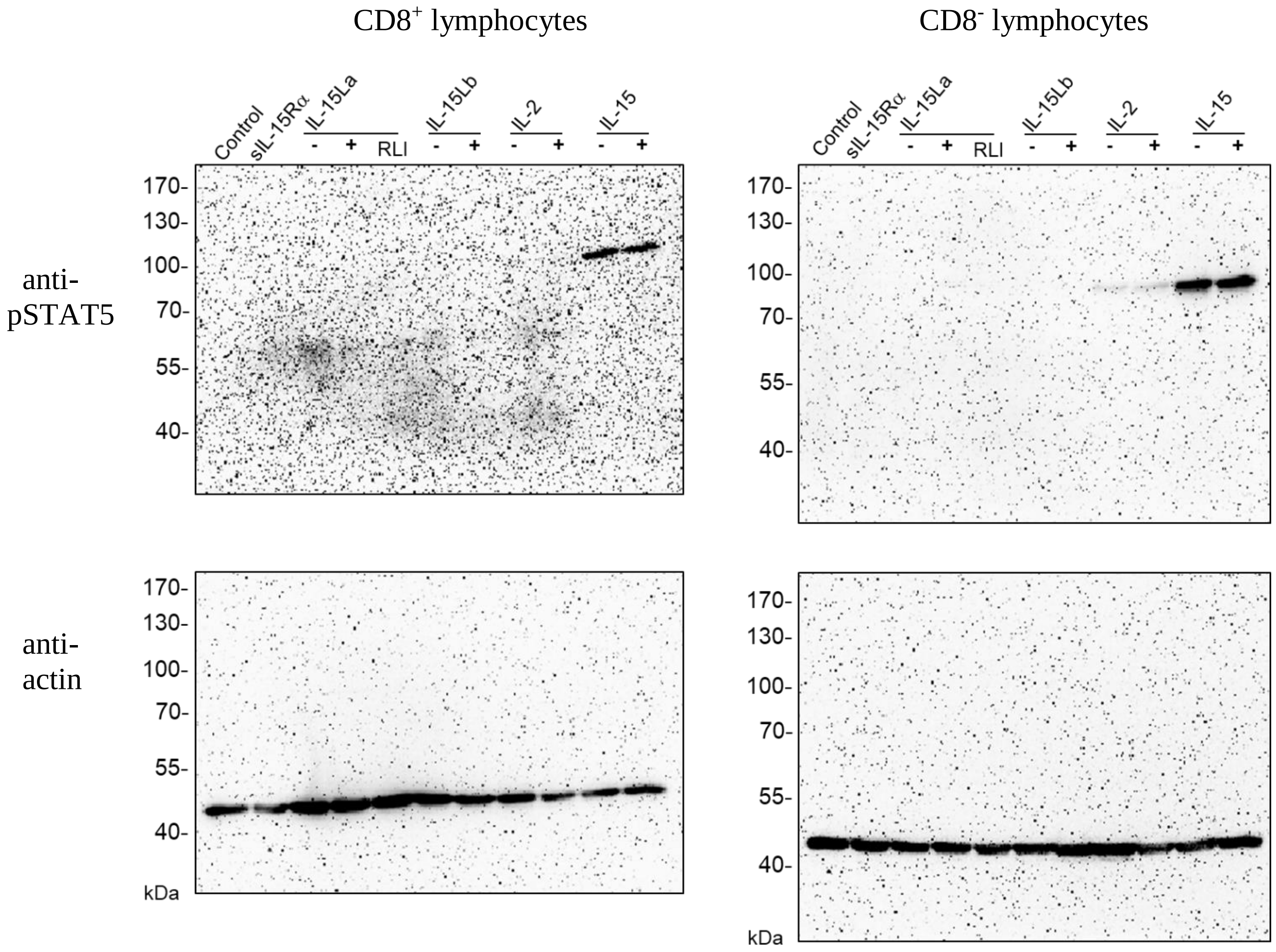

(Supplementary file 5C)

(c) *Gill#1*

Western blots

(c-1) *Results of the lymphocyte stimulation experiments*

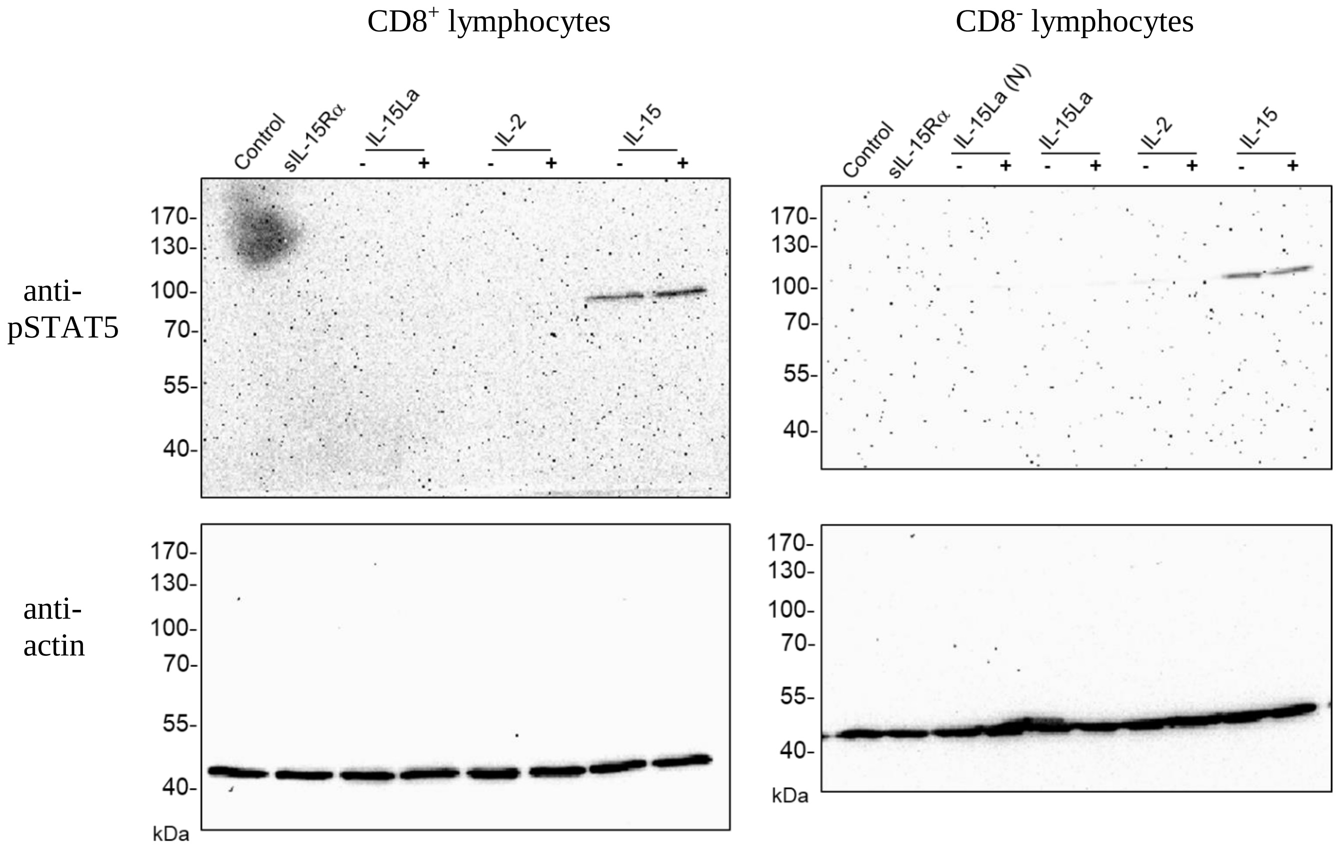

(c-2) *anti-FLAG Western blot of the HEK293T supernatants used for the stimulation*

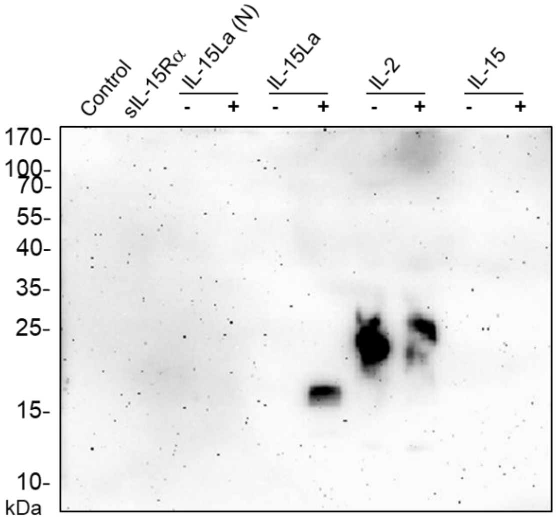

(Supplementary file 5C)

(d) *Gill#2*

Western blots

(d-1) Results of the lymphocyte stimulation experiments

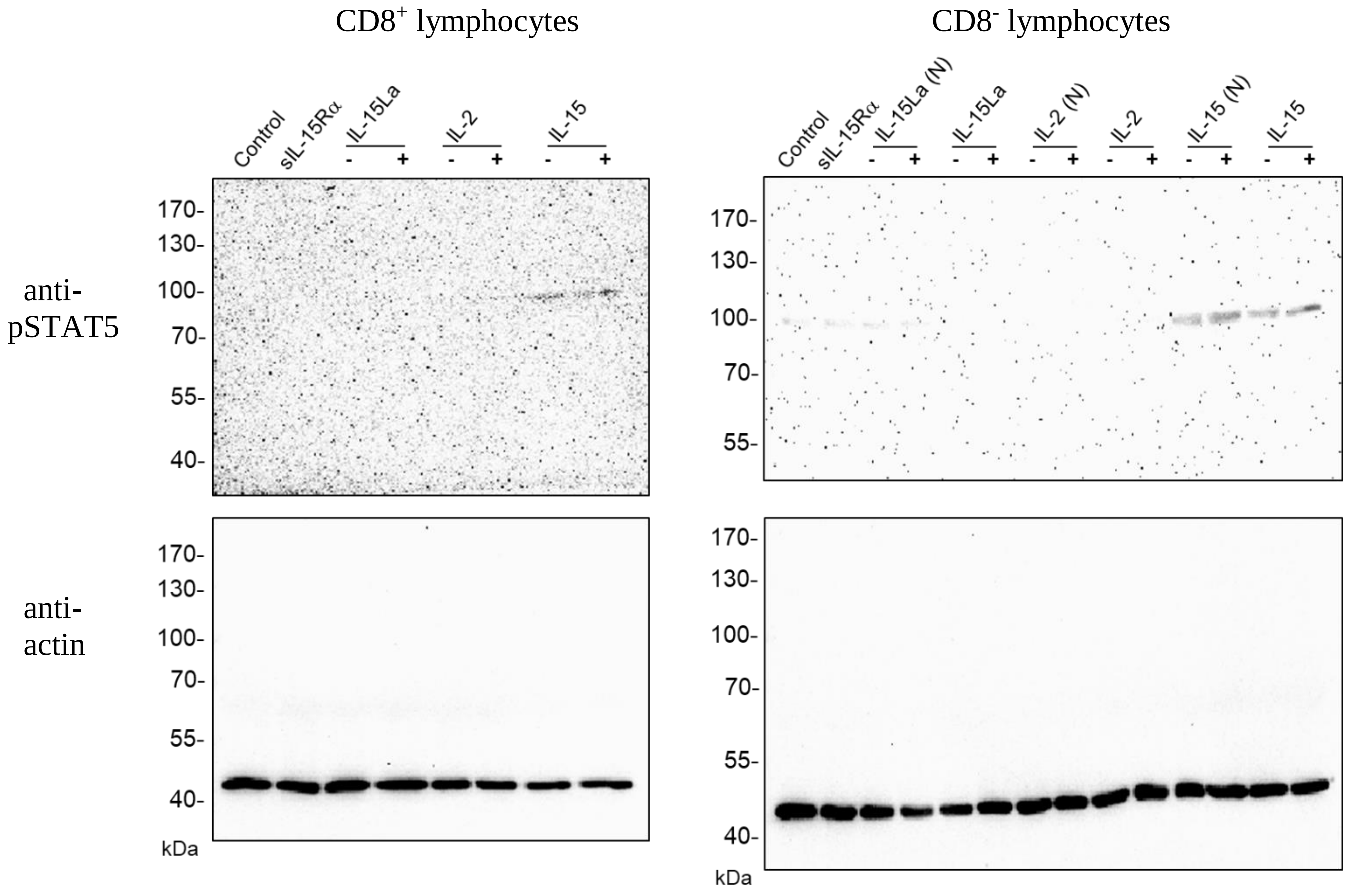

(d-2) *anti-FLAG Western blot of the HEK293T supernatants used for the stimulation*

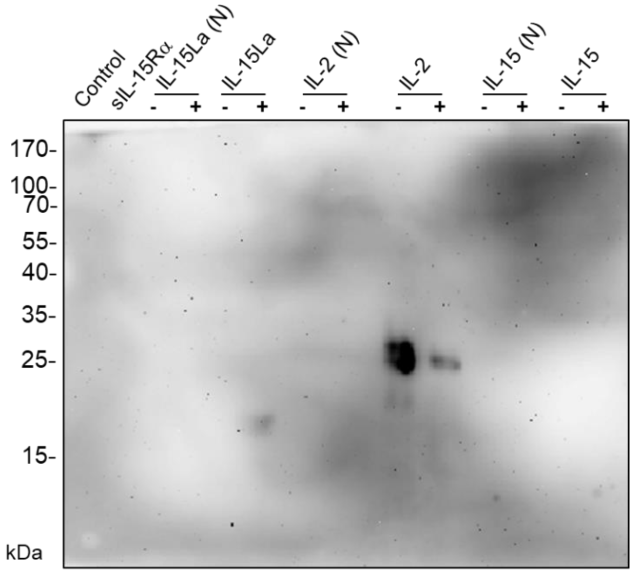

(Supplementary file 5C)

(e) *Spleen#1*

Western blots

(e-1) *Results of the lymphocyte stimulation experiments*

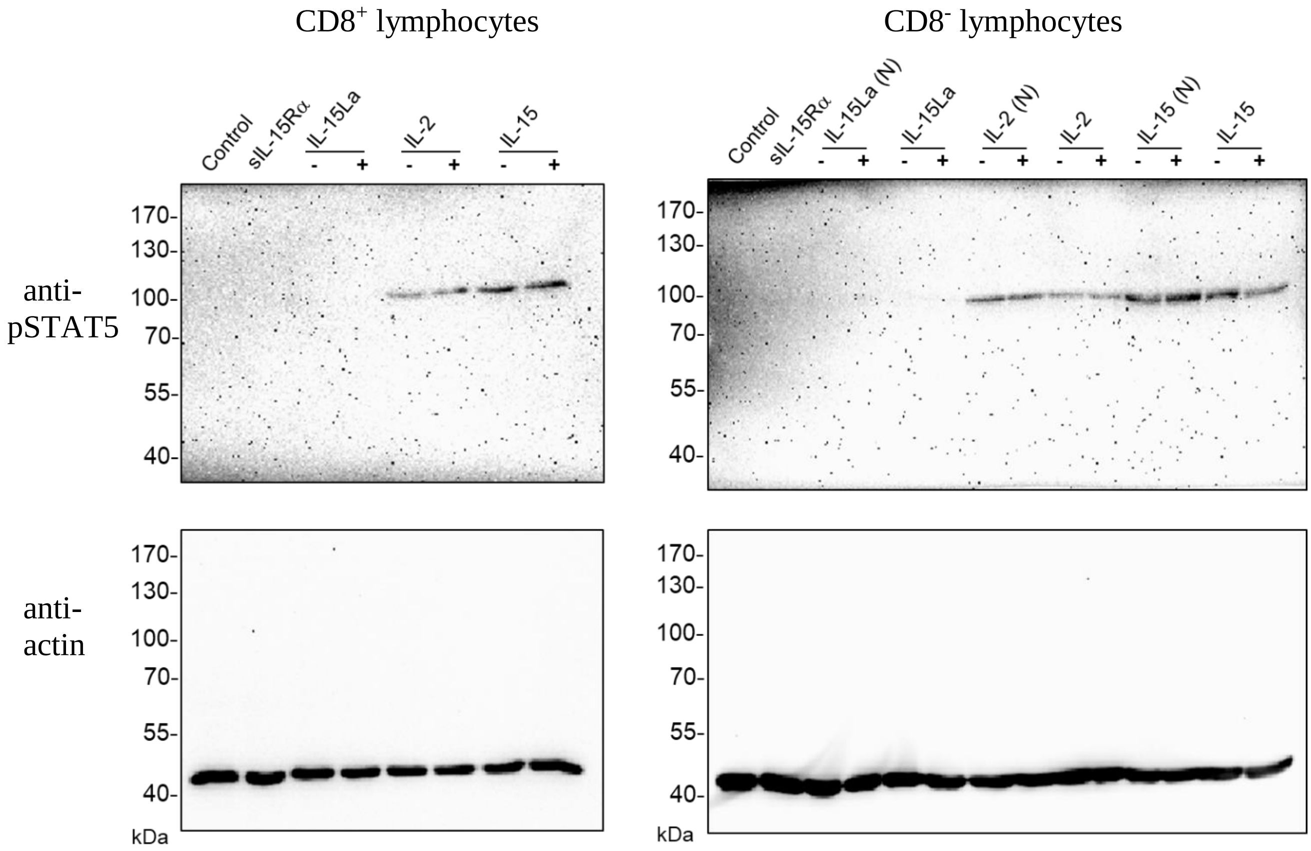

(e-2) *anti-FLAG Western blot of the HEK293T supernatants used for the stimulation*

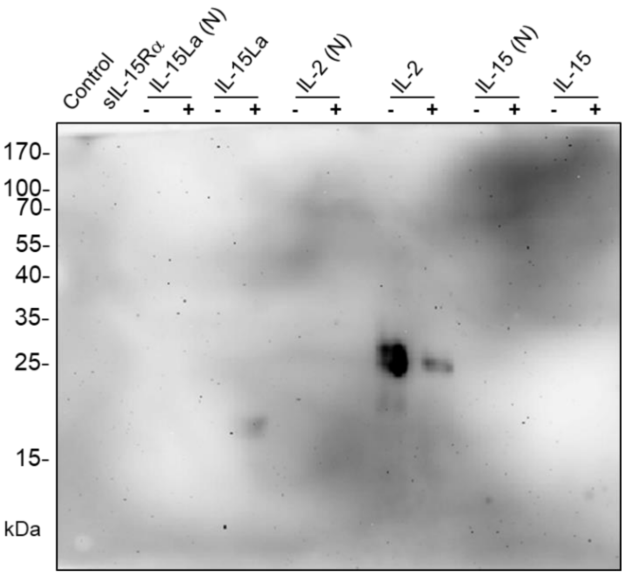

(Supplementary file 5C)

(f) *Spleen#2*

Western blots

(f-1) *Results of the lymphocyte stimulation experiments*

CD8$^+$ lymphocytes

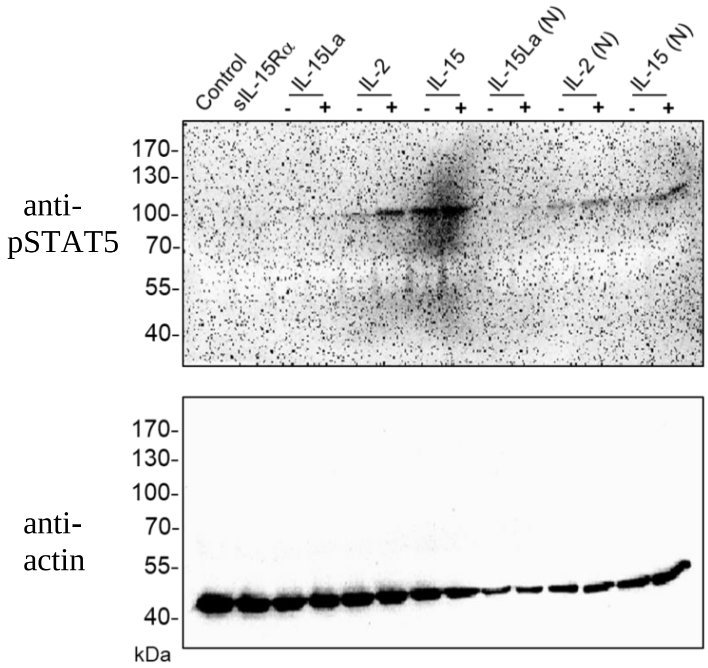

CD8$^-$ lymphocytes

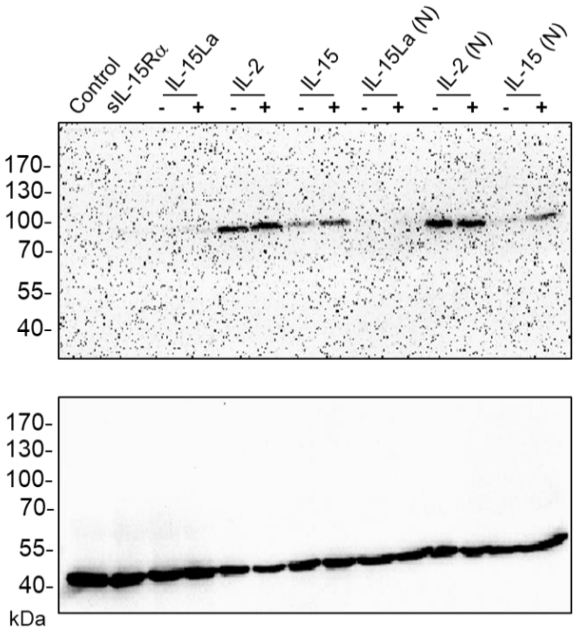

CD4$^-$CD8$^-$(DN) lymphocytes

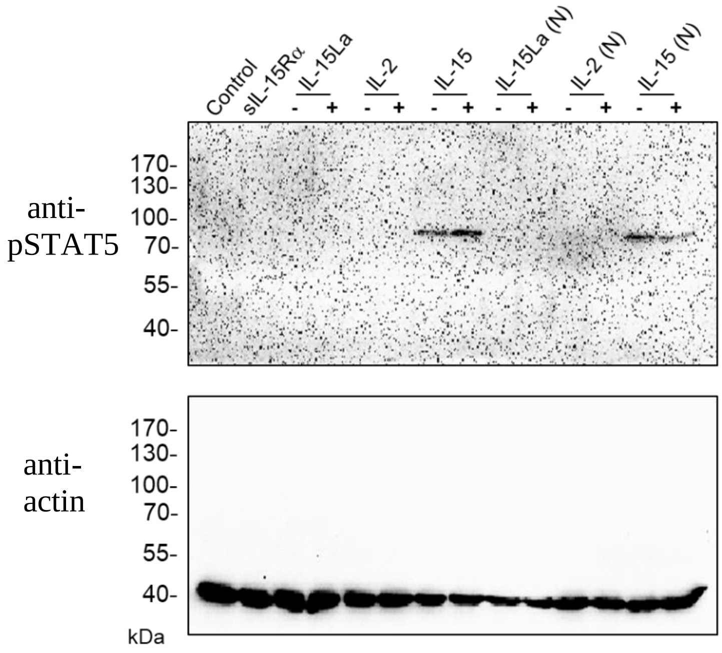

(f-2) *anti-FLAG Western blot of the HEK293T supernatants used for the stimulation*

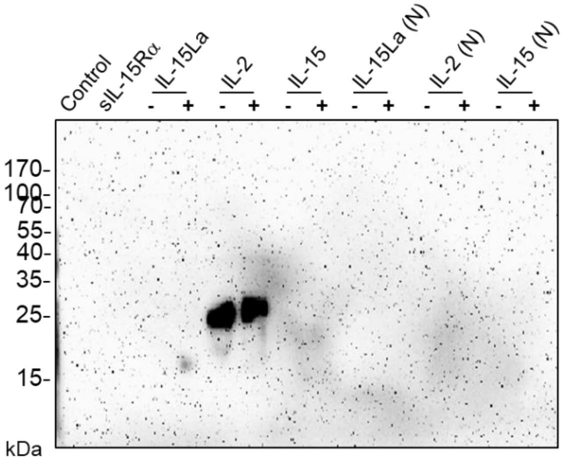

(Supplementary file 5C)

(g) *Head kidney#1*

Western blots

(g-1) *Results of the lymphocyte stimulation experiments*

CD8+ lymphocytes CD8- lymphocytes

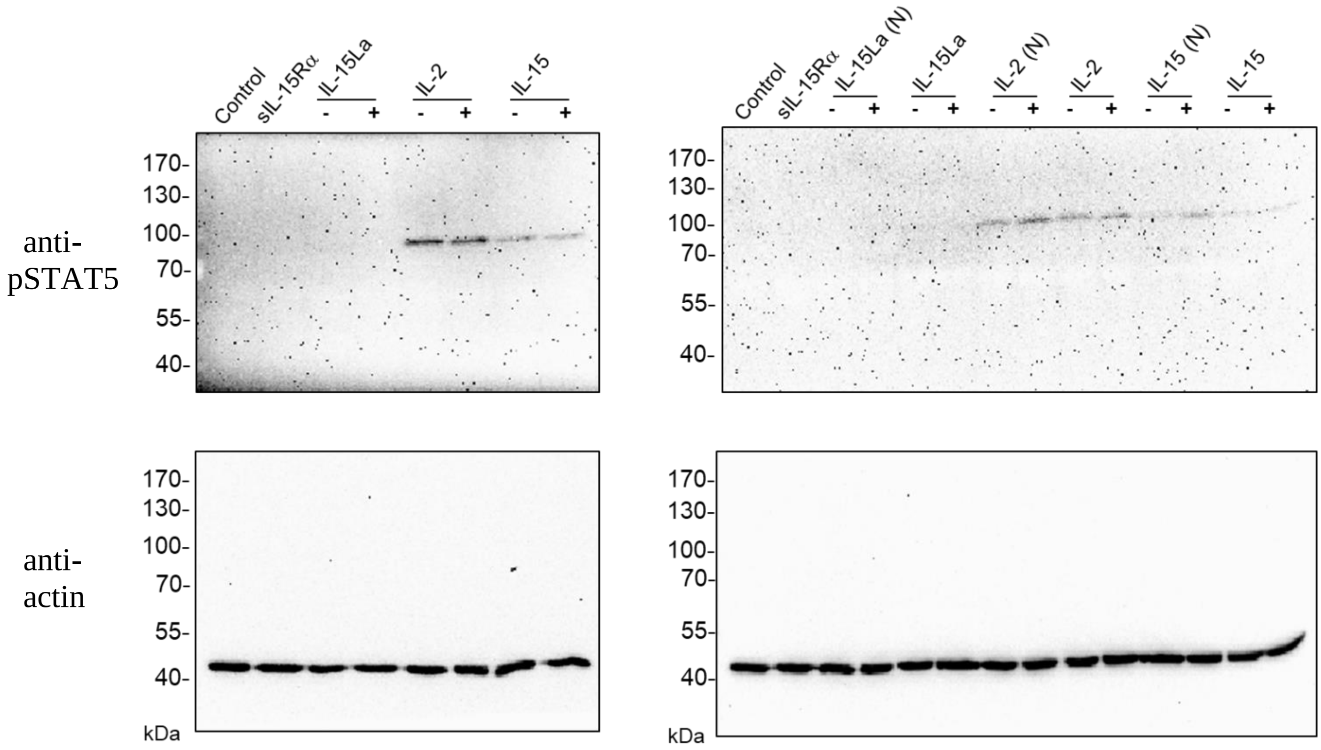

(g-2) *anti-FLAG Western blot of the HEK293T supernatants used for the stimulation*

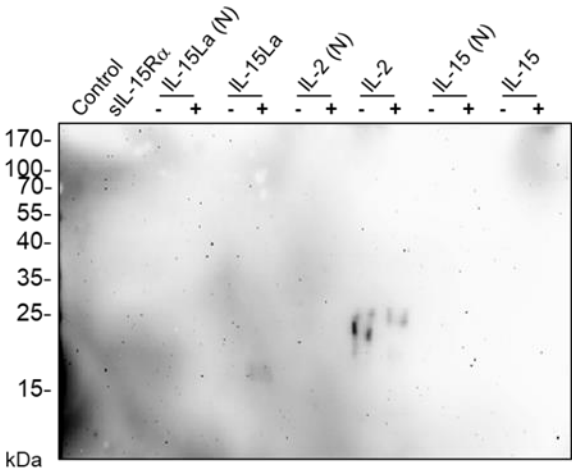

(Supplementary file 5C)

(h) *Head kidney#2*

Western blots

(h-1) *Results of the lymphocyte stimulation experiments*

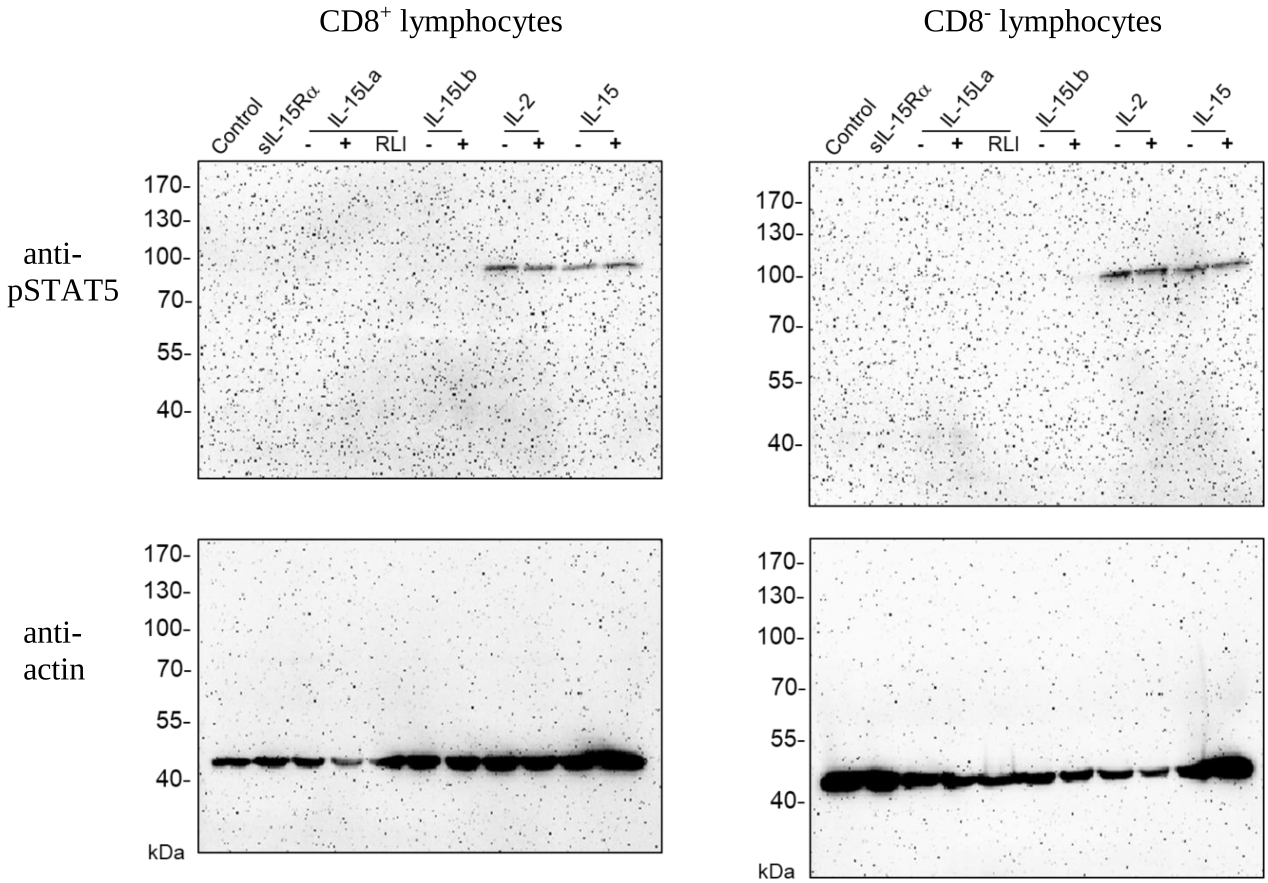

(h-2) *anti-FLAG Western blot of the HEK293T supernatants used for the stimulation*

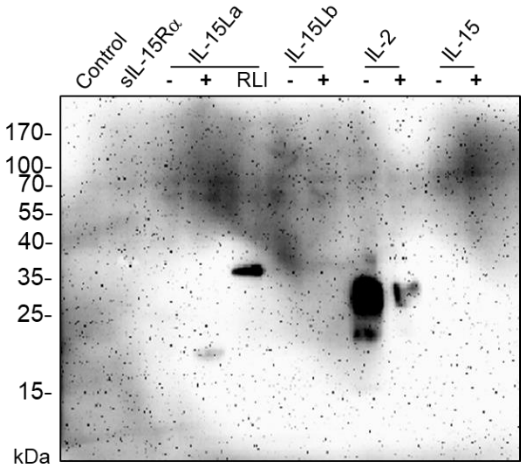

(Supplementary file 5C)

(i) *Thymus#1*

Western blots

(i-1) Results of the lymphocyte stimulation experiments

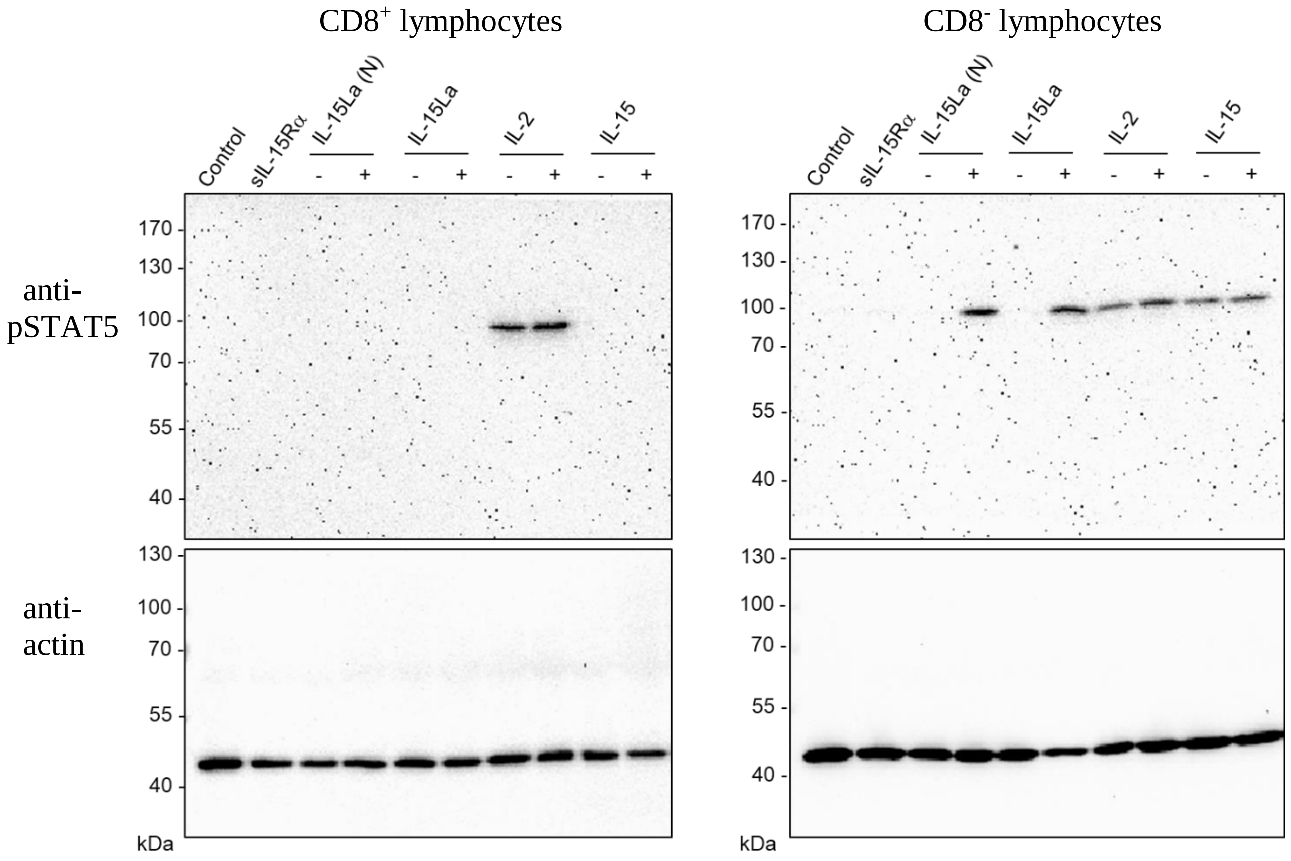

(i-2) *anti-FLAG Western blot of the HEK293T supernatants used for the stimulation*

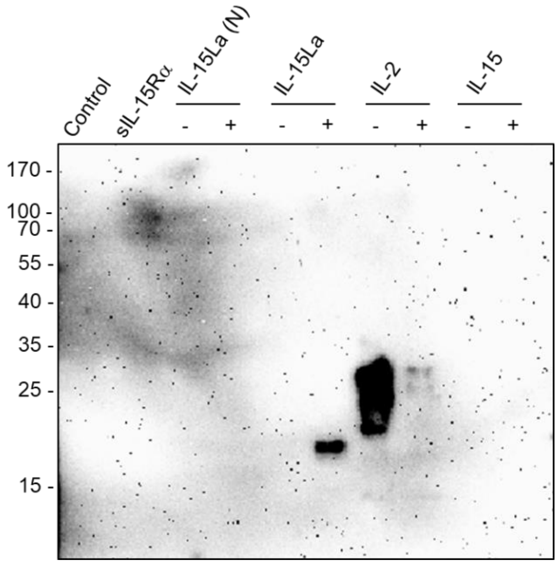

(Supplementary file 5C)

(j) *Thymus#2*

Western blots

(j-1) *Results of the lymphocyte stimulation experiments*

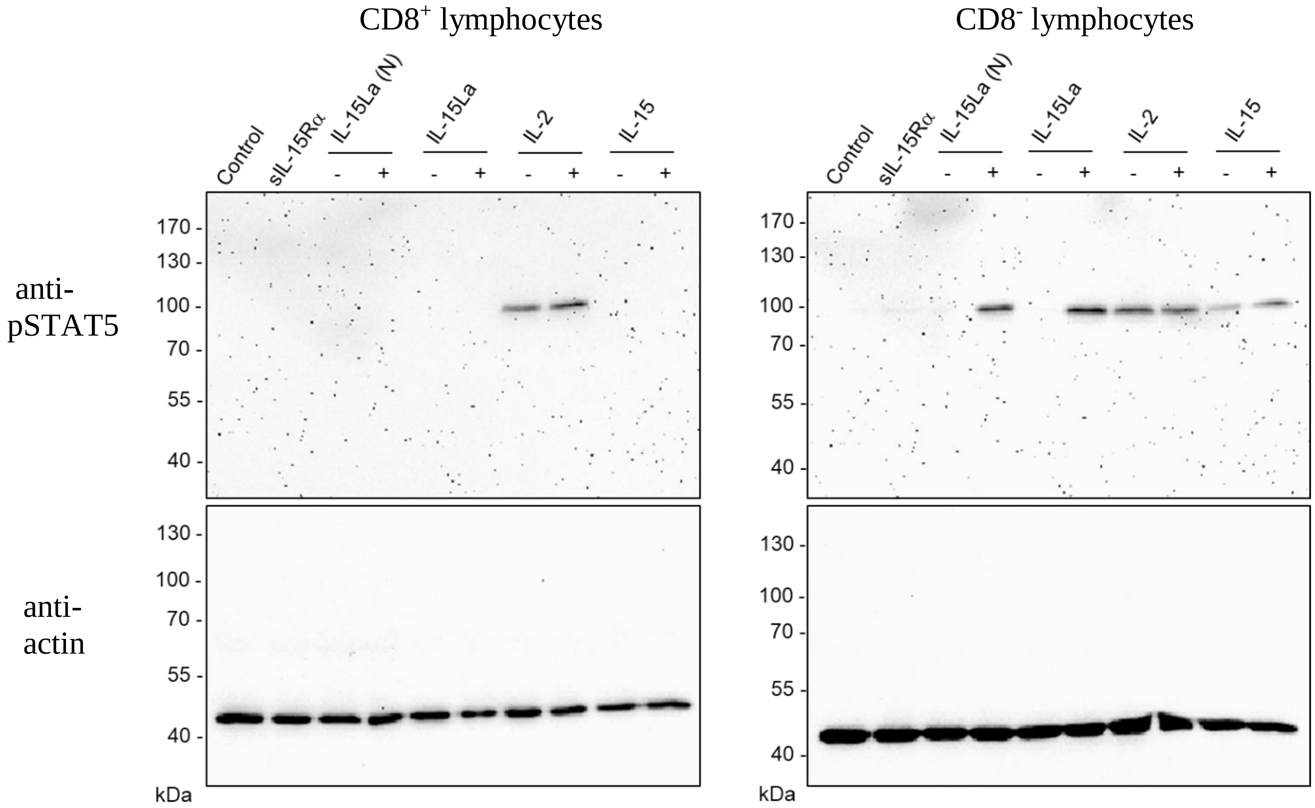

(j-2) *anti-FLAG Western blot of the HEK293T supernatants used for the stimulation*

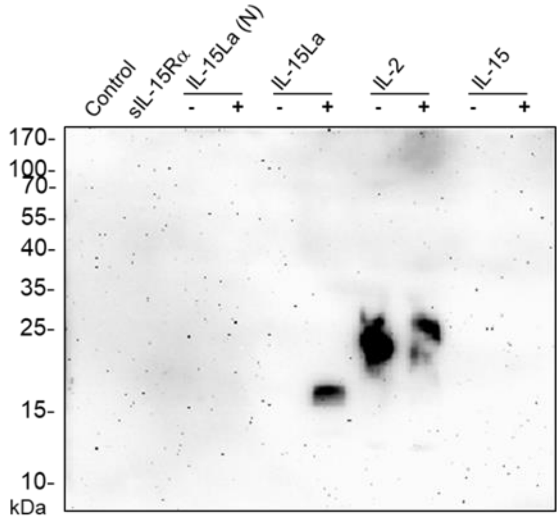

(Supplementary file 5C)

(k) *Thymus#3*

Western blots

(k-1) *Results of the lymphocyte stimulation experiments*

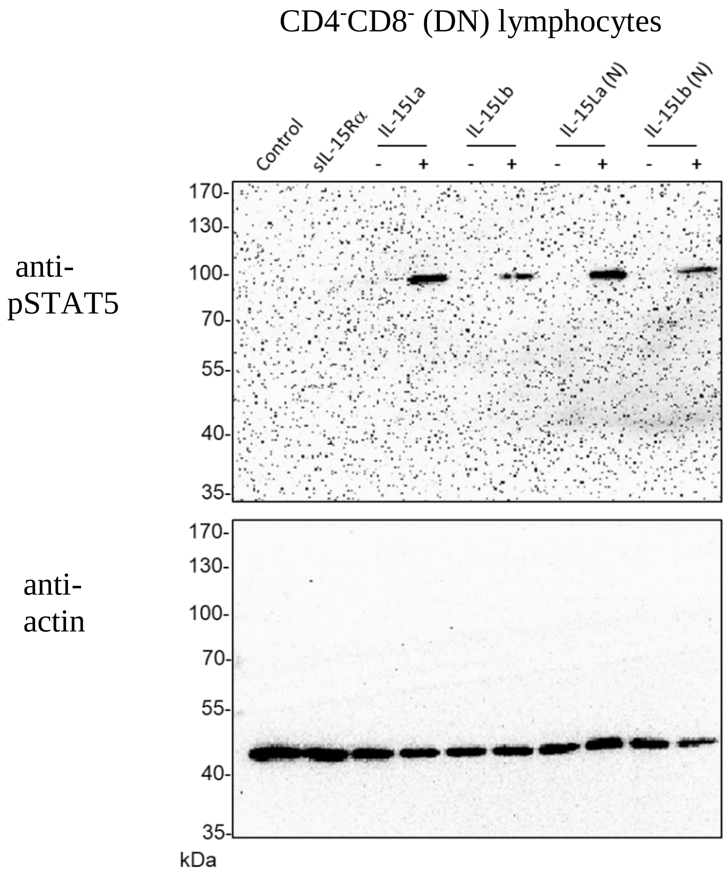

Supplementary file 5D. Phosphorylation of STAT5 in DN, DP, CD4SP and CD8SP fractions of trout thymocytes induced after incubation with recombinant cytokine containing HEK293T supernatants (*information supporting the main text Fig. 8 results*).

Western blot analyses of phosphorylated STAT5 (pSTAT5) in $CD8^-CD4^-$ (DN), $CD8^+CD4^+$ (DP), $CD8^+CD4^-$ (CD8SP), and $CD8^-CD4^+$ (CD4SP) fractions of morphological lymphocytes that had been isolated from trout thymus using flow sorting (for an example see Supplementary file 3) and had been incubated with recombinant trout cytokine containing supernatants from transfected HEK293T cells.

This Supplementary file supports the main text Fig. 8. Figure (a) is identical to main text Fig. 8, except that now also the loading control results are shown. Figure (b) is an independent confirmation of the main text Fig. 8 result.

Trout thymocytes were separated using appropriate antibodies and flow sorting into $CD8^-CD4^-$ (DN), $CD8^+CD4^+$ (DP), $CD8^+CD4^-$ (CD8SP), and $CD8^-CD4^+$ (CD4SP) populations. Isolated populations were incubated for 15 min at 15 °C with the supernatants of HEK293T cells transfected for trout IL-2, IL-15 or IL-15La, with or without trout sIL-15Rα. In a few cases, supernatants of HEK293T cells transfected for RLI fusion products including human sIL-15Rα were used (see explanation in the Supplementary file 5B legend). Experiments 1 and 2 were performed independently from each other, using thymocytes pooled from different trout individuals and HEK293T supernatants from different transfections.

(a) (Experiment 1). This figure is identical to main text Fig. 8, except that now also the anti-actin loading control blot results are shown. "-" and "+" refer to whether sIL-15Rα was co-expressed with the indicated cytokines. The RLI form used in this experiment was a fusion of human sIL-15Rα and trout IL-15La (IL-15La-h-RLI).

(b) (Experiment 2). (b-1) In addition to the cytokines used in Experiment 1, also a fusion between human sIL-15Rα and modified bovine IL-15L was tested (bov.IL-15Lhyb-h-RLI). This incubation of the modified bovine molecule with trout lymphocytes was just one of our many different trials to find a function for bovine IL-15L, none of which produced positive results so far (this negative lane is not relevant for the present study, but was left to show the intact blot result). (b-2) Different from Experiment 1, in Experiment 2 the unconcentrated HEK293T supernatants were subjected to anti-FLAG Western blot analysis. As discussed in the Supplementary file 5C legend, not for all cytokines the concentrations were sufficiently high for being detectable in this manner, but the blot result helps to see for example that the IL-2 concentrations used were higher than the IL-15 concentrations.

(Supplementary file 5D)

(a) *Experiment 1*

Main text Fig. 8 but now also showing the anti-actin loading control results.

Western blots

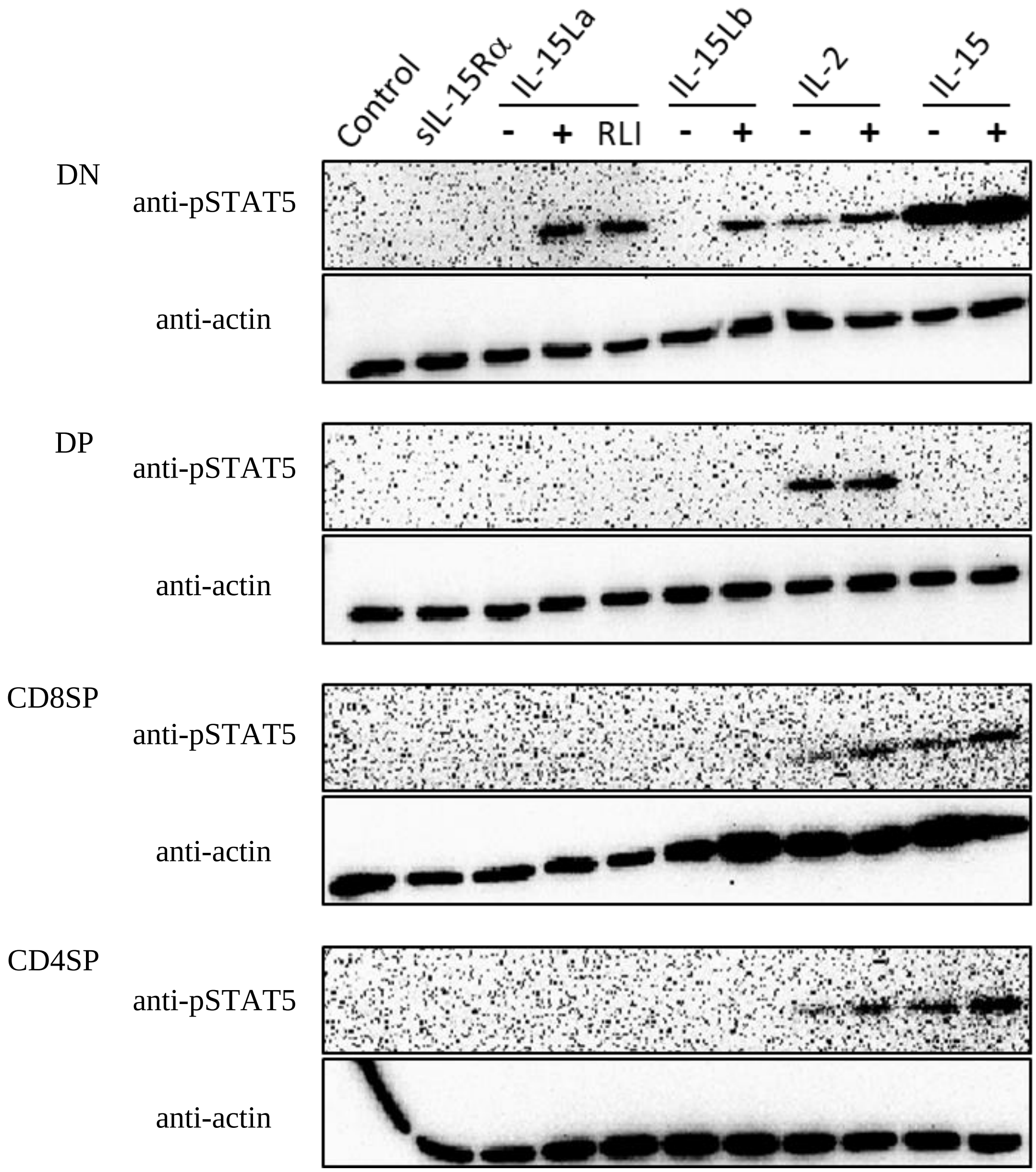

(Supplementary file 5D)

(b) *Experiment 2*

Independent confirmation of the main text Fig. 8 result. Western blots

(b-1) Results of the lymphocyte stimulation experiments. Upper blots show anti-pSTAT5 results, lower blots show the anti-actin loading control results

M, marker; 1, Vector control; 2, trout sIL-15Ra; 3, IL-15La-h-RLI (trout IL-15La linked with human sIL-15Rα); 4, bov.IL-15Lhyb-h-RLI (modified bovine IL-15L linked with human sIL-15Rα); 5, trout IL-15La; 6, trout IL-15La+sIL-15Rα; 7, trout IL-15Lb; 8, trout IL-15Lb+sIL-15Rα; 9, trout IL-2; 10, trout IL-2+sIL-15Rα; 11, trout IL-15; 12, trout IL-15+sIL-15Rα.

DN

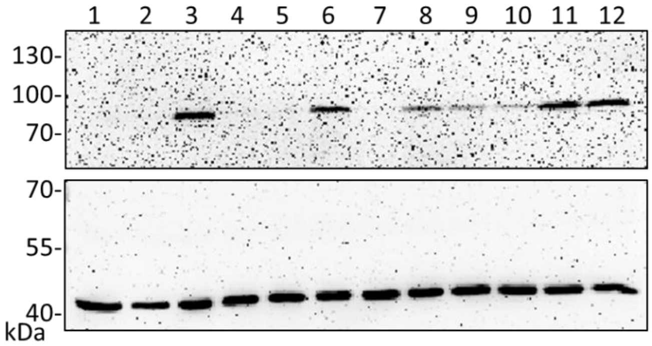

DP

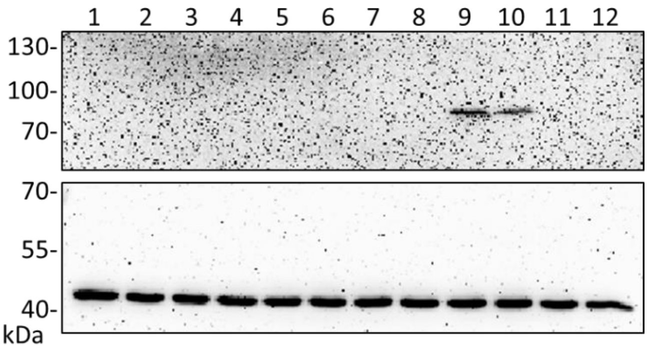

CD8SP

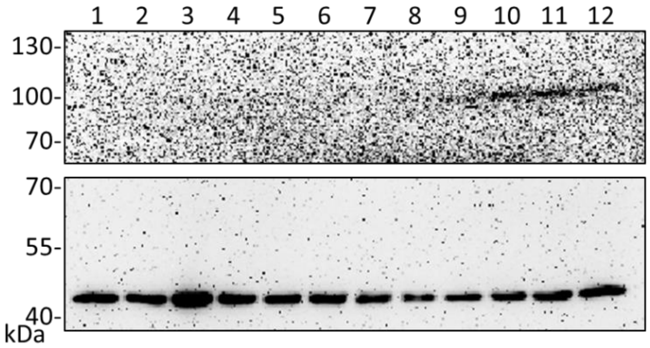

CD4SP

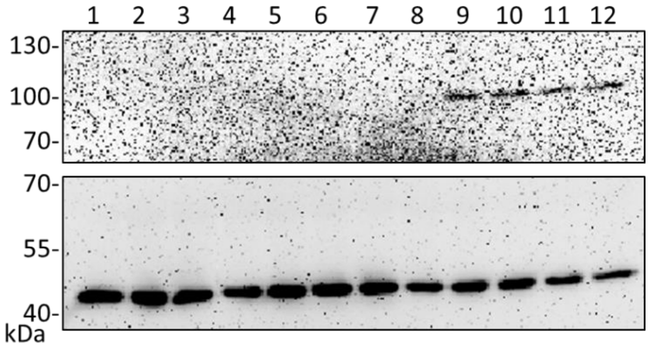

(b-2) anti-FLAG Western blot of the HEK293T supernatants used for the stimulation

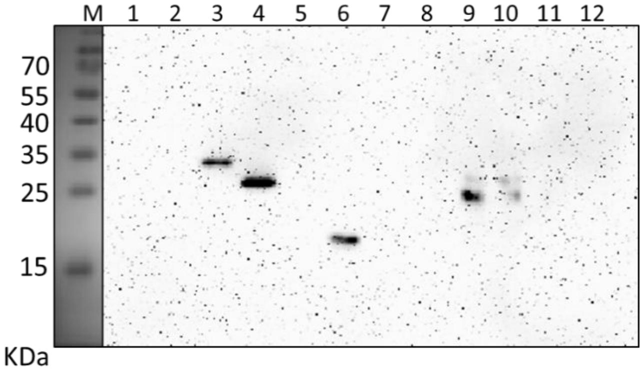

Supplementary file 5E. Phosphorylation of STAT5 in lymphocyte fractions of trout thymus, intestine and spleen, induced after incubation with purified recombinant trout cytokines (produced in insect cells) IL-2, IL-15-RLI and IL-15La-RLI at 5, 25 and 125 nM (*information supporting the main text Fig. 9 results*).

Western blot analyses of phosphorylated STAT5 (pSTAT5) in $CD8^-CD4^-$ (DN), $CD8^+CD4^+$ (DP; only isolated from thymocytes), $CD8^+CD4^-$ (CD8SP), and $CD8^-CD4^+$ (CD4SP) fractions of morphological lymphocytes that had been isolated from trout thymus, intestine and spleen using flow sorting (for examples see Supplementary file 3) and had been incubated for 15 min at 15 °C with recombinant trout cytokines purified from insect cells (for protein purification see Supplementary file 4). These cytokines were trout IL-2, IL-15-RLI (a fusion of trout IL-15 and trout sIL-15Rα) and IL-15La-RLI (a fusion of trout IL-15La and trout sIL-15Rα), and they were used in 5, 25 and 125 nM concentrations. As negative controls, cells were mock treated (Control). After stripping and washing, the same blots were analyzed by Western blot analysis using anti-actin as a loading control.

This Supplementary file supports the main text Fig. 9. Figure (a) is identical to main text Fig. 9, except that now also the anti-actin loading control results are shown. Figure (b) is based on an independent but similar experiment as done for figure (a), although only done for thymocytes. The (b) result confirms many of the observations in (a) for thymocytes, for example that DN thymocytes are sensitive to IL-15La-RLI and that DP thymocytes are only sensitive to IL-2. However, comparison of the (a) and (b) results for thymocytes also shows that preparations pooled from different trout individuals can have different relative sensitivities to the different cytokines, as for example the DN thymocyte preparation used for the (b) experiment was more sensitive to IL-15La-RLI than to IL-2 or IL-15-RLI. In the future, when hopefully the respective cytokine receptors will have been determined and antibodies will have been established against those receptors, the different sensitivities for cytokines between cells of different trout individuals may be addressed in more detail.

(Supplementary file 5E)

(a) *Main text Fig. 9 with anti-actin loading control.*

Western blots

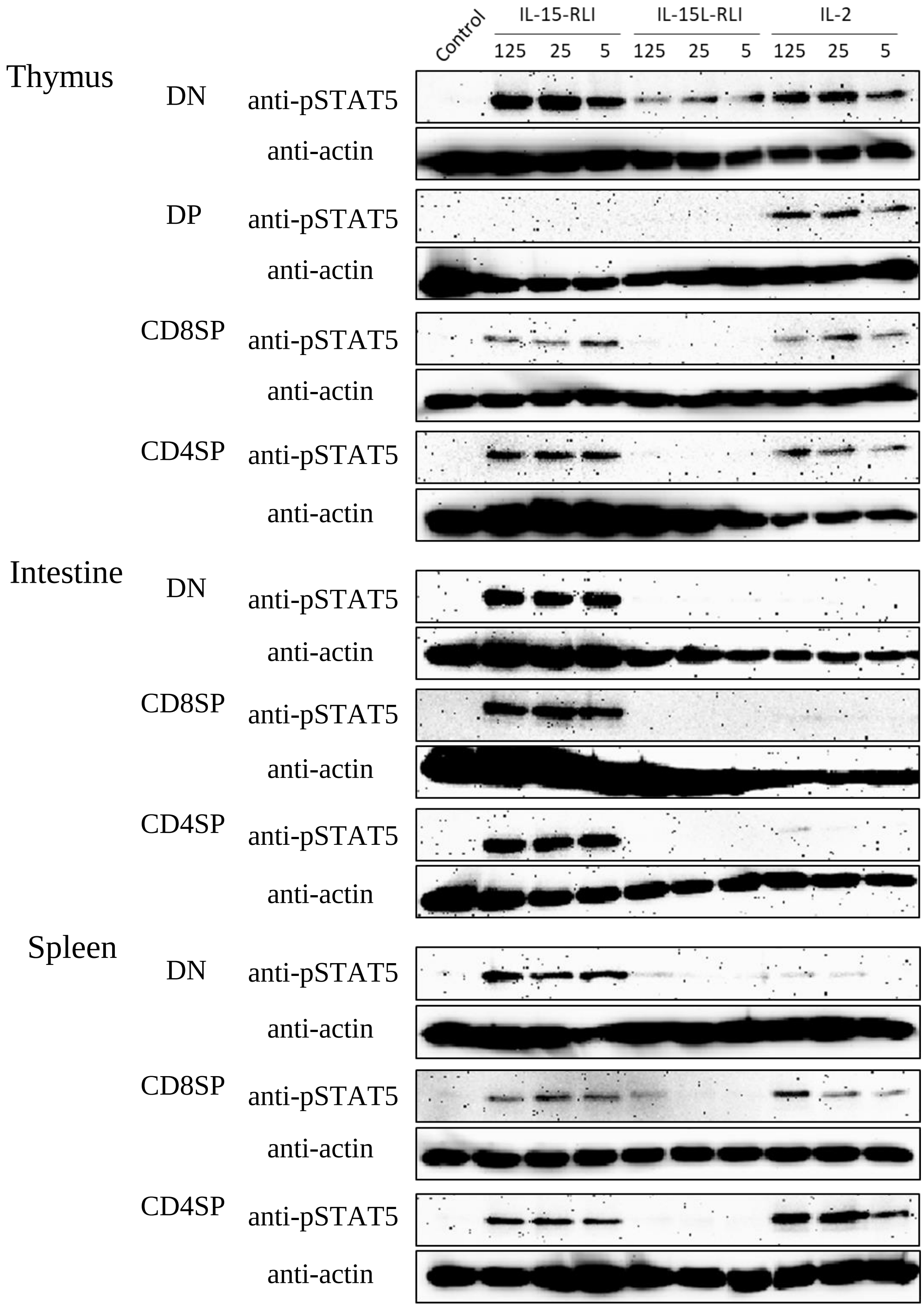

(Supplementary file 5E)

(b) *Stimulation of thymocytes pooled from different trout individuals than used for the experiment shown in (a).*

Western blots

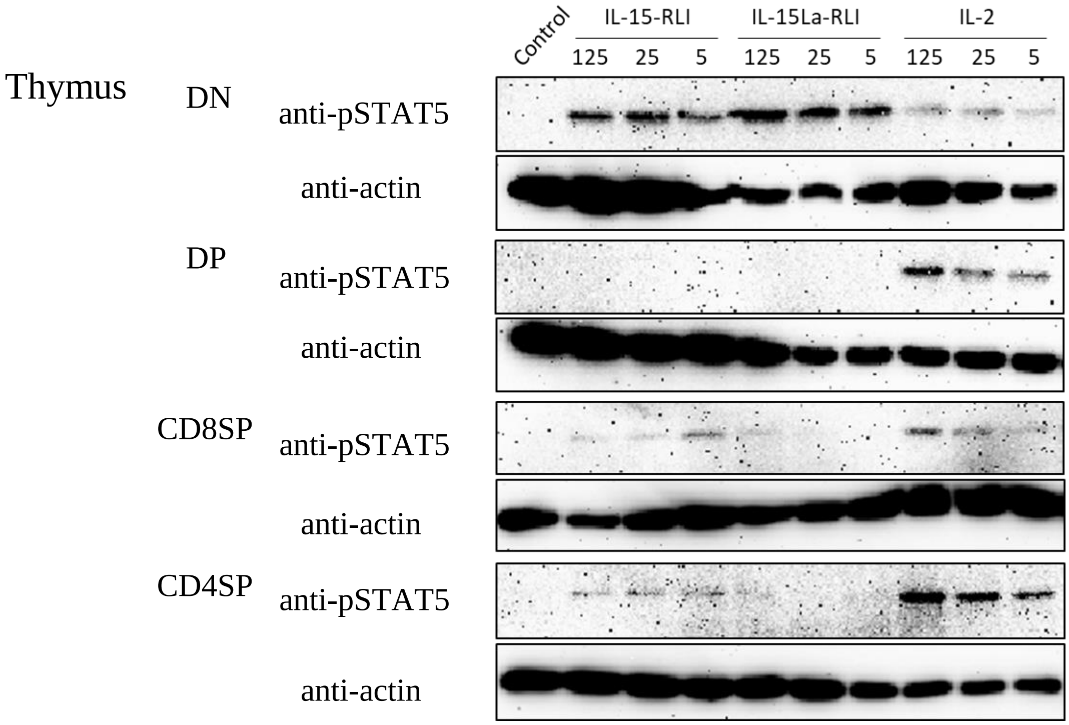

Supplementary file 5F. Titration of the recombinant trout cytokine (produced in insect cells) concentration necessary for the induction of pSTAT5.

Western blot analyses of phosphorylated STAT5 (pSTAT5) in sensitive trout lymphocyte populations that had been isolated using flow sorting (for examples see Supplementary file 3) and had been incubated for 15 min at 15 °C with recombinant trout cytokines purified from insect cells (for protein purification see Supplementary file 4). These cytokines were trout IL-2, IL-15-RLI (a fusion of trout IL-15 and trout sIL-15Rα), IL-15La-RLI (a fusion of trout IL-15La and trout sIL-15Rα), IL-15/sIL-15Rα (noncovalent complex of trout IL-15 and trout sIL-15Rα) and IL-15/sIL-15Rα (noncovalent complex of trout IL-15 and trout sIL-15Rα), and they were used in 0.008-to-25 nM concentrations. As negative controls, cells were mock treated (Control). After washing, the same blots were analyzed by Western blot analysis using anti-actin as a loading control.

If using sensitive cells, a weak induction of pSTAT5 could be observed even at 0.008 nM for IL-2, IL-15-RLI and IL-15La-RLI. When comparing IL-15-RLI with IL-15/sIL-15Rα, or IL-15La-RLI with IL-15La/sIL-15Rα, similar sensitivities were observed, which suggests that the fusion proteins were functionally similar to the corresponding noncovalently associated complexes.

(a) Comparison of the pSTAT5 inducing effects of IL-15-RLI and IL-15/sIL-15Rα on the $CD8^-$ fraction of intestinal lymphocytes that had been separated by into $CD8^+$ and $CD8^-$ populations by flow sorting. Both cytokine preparations were able to induce detectable pSTAT5 levels from a concentration of 0.2 nM, which suggests their functional similarity.

(b) Comparison of the pSTAT5 inducing effects of IL-15La-RLI and IL-15La/sIL-15Rα on the $CD8^-$ fraction of thymocytes that had been separated into $CD8^+$ and $CD8^-$ populations by flow sorting. Both cytokine preparations were able to induce detectable pSTAT5 levels already at a concentration of 0.008 nM which suggests that IL-15La-RLI is at least similarly potent as IL-15La/sIL-15Rα.

(c) Analysis of the pSTAT5 inducing effects of IL-15-RLI on the $CD8^-$ fraction of head kidney lymphocytes that had been separated into $CD8^+$ and $CD8^-$ populations by flow sorting. It was found that IL-15-RLI can induce detectable pSTAT5 levels already at a concentration of 0.008 nM.

(d) Analysis of the pSTAT5 inducing effects of IL-2 on the $CD8^-$ fraction of thymocytes that had been separated into $CD8^+$ and $CD8^-$ populations by flow sorting. It was found that IL-2 can induce detectable pSTAT5 levels already at a concentration of 0.008 nM.

(Supplementary file 5F)

(a) *Similar potencies of trout IL-15-RLI fusion protein and non-covalent association of trout IL-15 with trout sIL-15Rα.*

Western blots

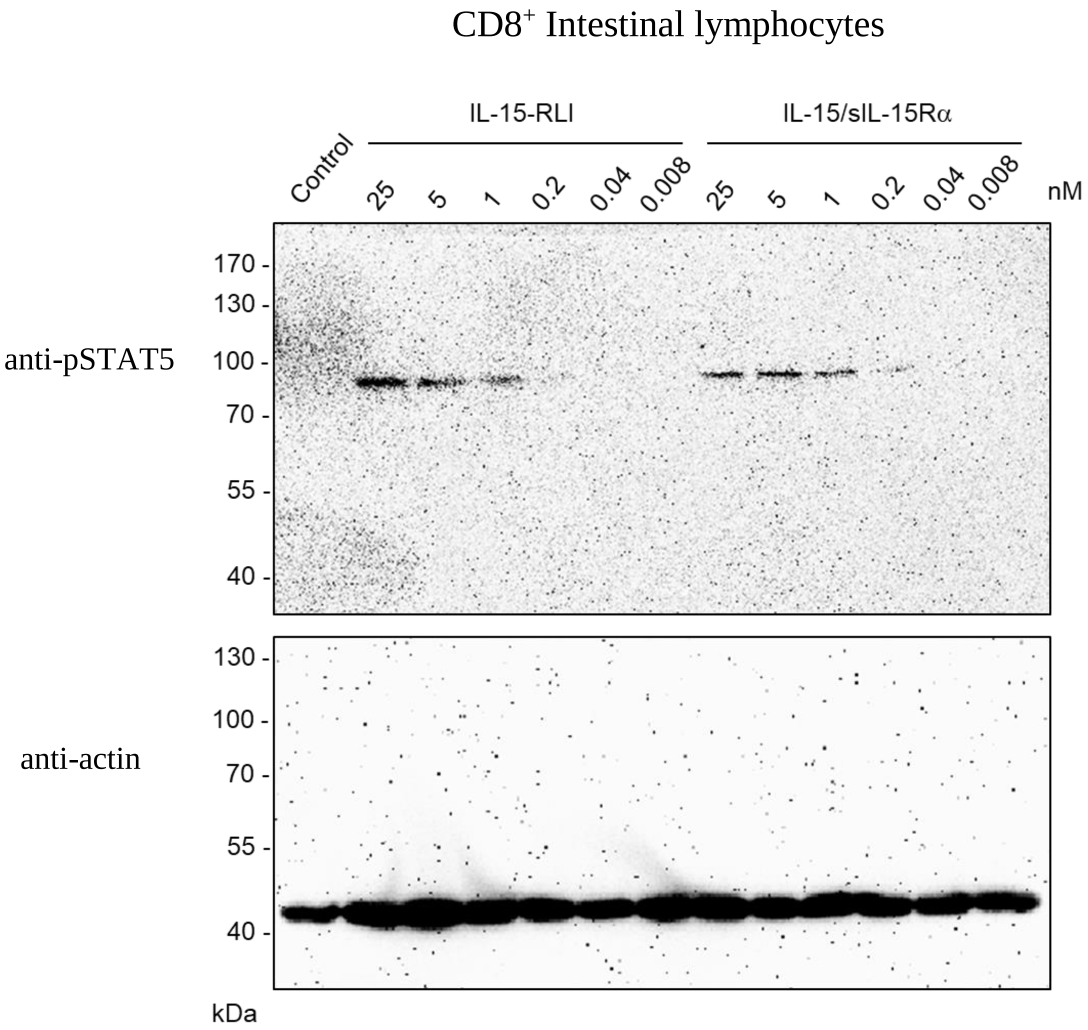

(Supplementary file 5F)

(b) *Similar potencies of trout IL-15La-RLI fusion protein and non-covalent associations of trout IL-15La with trout sIL-15Rα.*

Western blots

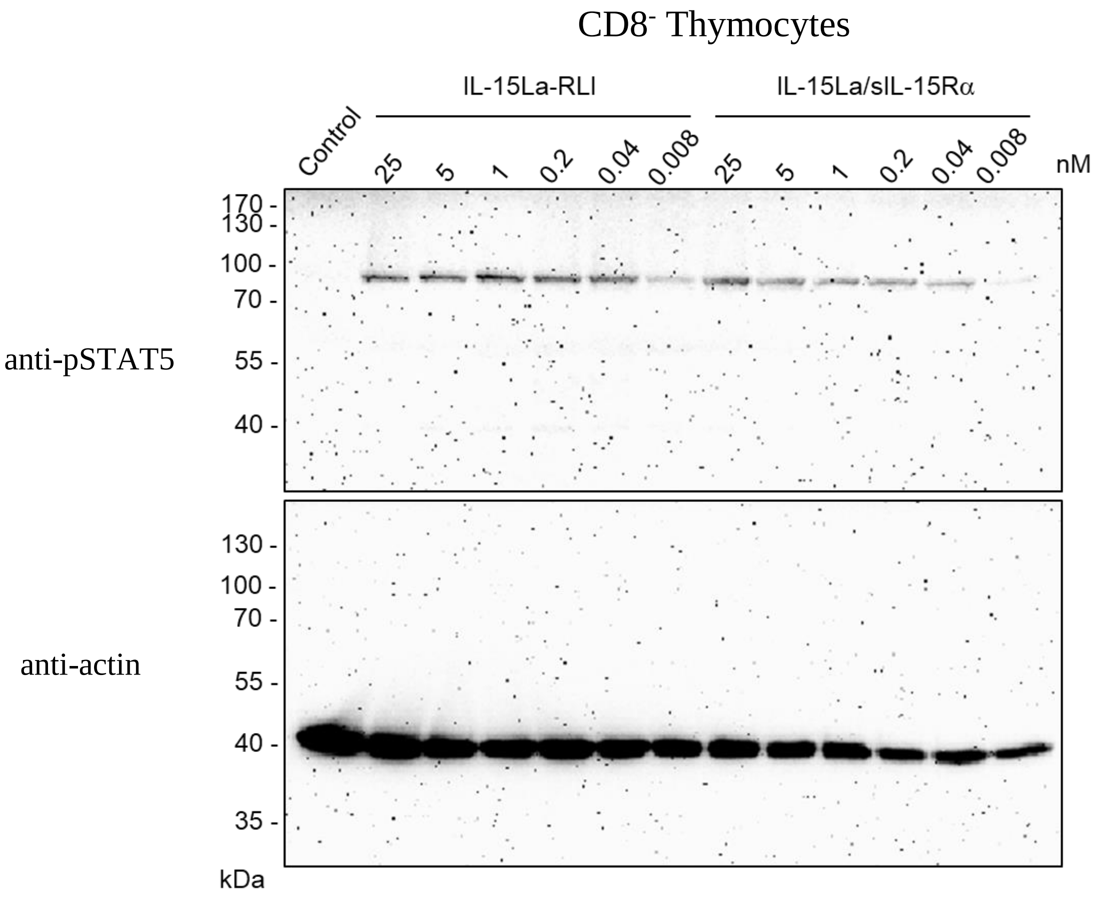

(Supplementary file 5F)

(c) *High sensitivity of trout CD8⁻ head kidney thymocytes to trout IL-15-RLI.*

Western blots

CD8⁻ Head kidney lymphocytes

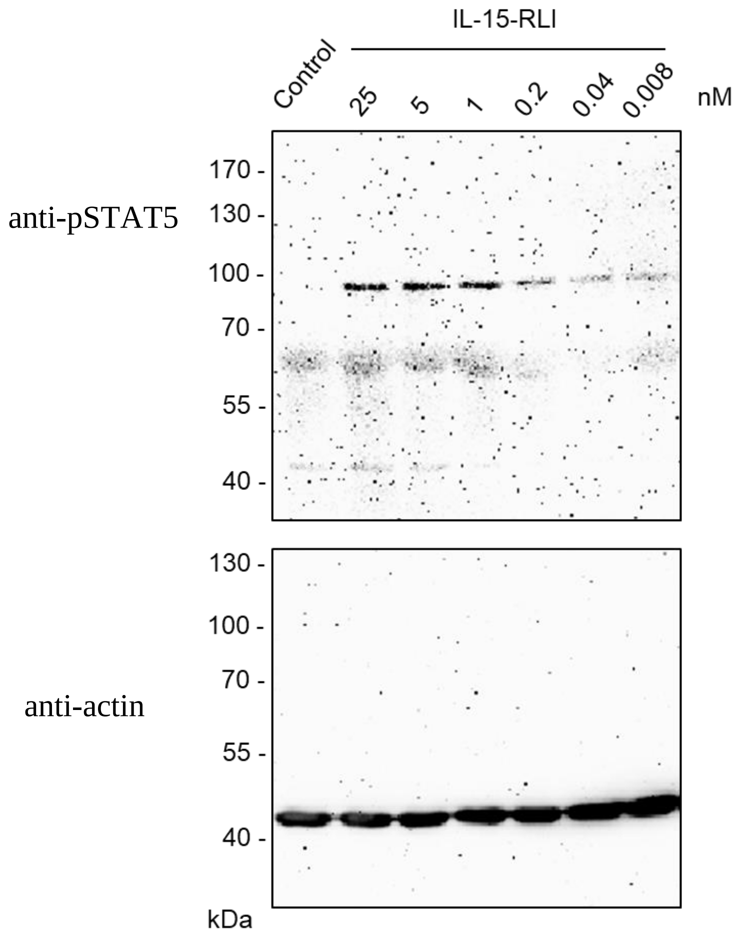

(Supplementary file 5F)

(d) *High sensitivity of trout CD8⁻ thymocytes to trout IL-2.*

Western blots

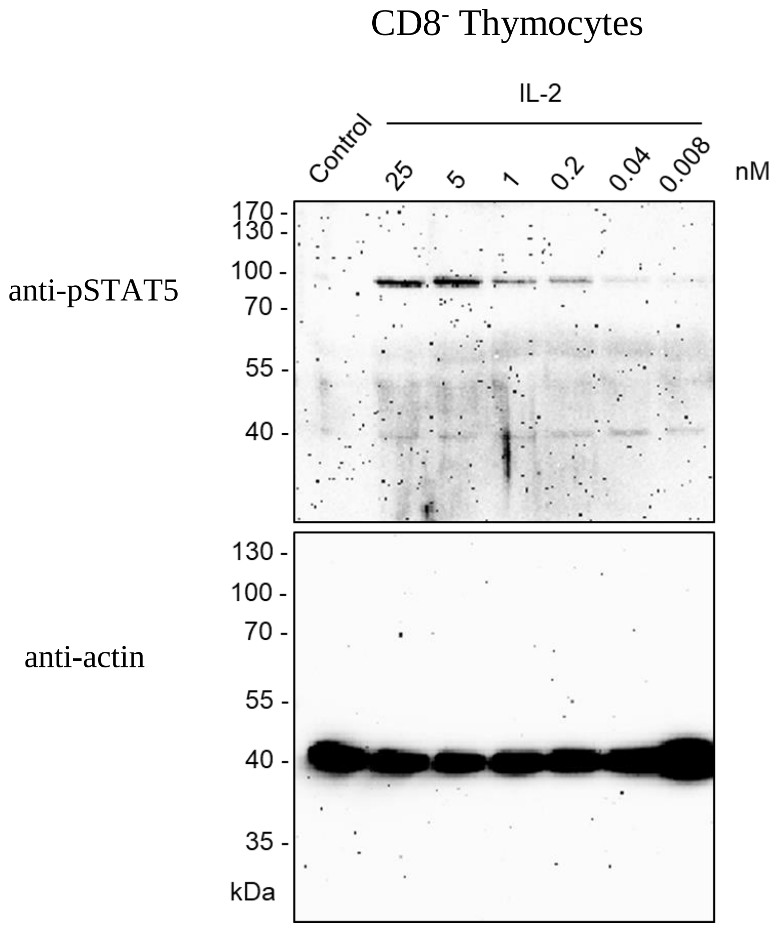

Supplement: Supplementary file 5 [file Data_Sheet_5.PDF]
