# Supplementary material for: Ancient Cytokine Interleukin 15-Like (IL-15L) Induces a Type 2 Immune Response
Source: Front Immunol. 2020 Oct 29;11:549319. doi: 10.3389/fimmu.2020.549319 (PMC7658486; doi:10.3389/fimmu.2020.549319)
Supplement: Supplementary file 6 [file Data_Sheet_6.PDF]

Supplementary file 7

Table with recombinant cytokine expression levels at the surface of transfected cells as determined by flow cytometry.

Percentages of $FLAG^{+}$ cells among live HEK293T cells after their transfection for FLAG-tagged trout or bovine IL-2/15/15L-family cytokines with or without co-transfection for trout or bovine IL-15Rα or bovine IL-2Rα. The values were determined by flow cytometry as shown in main Text Fig. 5, and include the data shown in Fig. 5 plus those of independent experiment repeats. Light orange color is used for highlighting values that were deemed indicative for cytokine to alpha chain receptor binding.

Trout cytokines

IL-2 only	IL-2 + Trout IL-15Rα	IL-2 only	IL-2 + Bovine IL-2Rα	IL-2 only	IL-2 + Bovine IL-15Rα
11.04	73.97	11.04	3.46	11.04	87.36
0.47	31.28	0.58	0.95	0.55	20.37
0.45	17.90				
6.70	64.92	0.45	0.92	0.19	22.79

IL-15 only	IL-15 + Trout IL-15Rα	IL-15 only	IL-15 + Bovine IL-2Rα	IL-15 only	IL-15 + Bovine IL-15Rα
1.44	76.24	1.44	1.61	1.44	95.14
0.74	35.07	0.73	1.29	1.06	31.90
0.80	20.63				
1.85	72.38	0.51	0.82	0.60	37.35

IL-15La only	IL-15La + Trout IL-15Rα	IL-15La only	IL-15La + Bovine IL-2Rα	IL-15La only	IL-15La + Bovine IL-15Rα
12.29	20.38	12.29	8.08	12.29	86.46
3.69	28.68	3.95	4.79	3.07	32.43
2.83	17.46				
11.06	61.56	2.29	3.08	4.30	37.00

IL-15Lb only	IL-15Lb + Trout IL-15Rα	IL-15Lb only	IL-15Lb + Bovine IL-2Rα	IL-15Lb only	IL-15Lb + Bovine IL-15Rα
0.03	10.73	0.03	0.24	0.03	29.28
0.52	3.18	0.52	0.21	0.52	10.45
0.72	4.27	0.72	0.76	0.72	15.61

Bovine cytokines

IL-2 only	IL-2 + Trout IL-15Rα	IL-2 only	IL-2 + Bovine IL-2Rα	IL-2 only	IL-2 + Bovine IL-15Rα
0.35	0.18	0.35	27.70	0.35	0.43
0.51	0.45	0.39	42.48	0.39	0.35
0.64	1.67	0.48	23.80	0.26	0.49

IL-15 only	IL-15 + Trout IL-15Rα	IL-15 only	IL-15 + Bovine IL-2Rα	IL-15 only	IL-15 + Bovine IL-15Rα
54.46	68.94	54.46	51.34	54.46	91.19
15.41	29.22	15.41	8.24	15.41	44.42
17.88	34.62	17.88	10.37	17.88	51.70

IL-15L only	IL-15L + Trout IL-15Rα	IL-15L only	IL-15L + Bovine IL-2Rα	IL-15L only	IL-15L + Bovine IL-15Rα
1.89	22.71	1.89	2.29	1.89	68.90
1.72	6.17	2.94	3.88	2.94	45.82
2.21	5.86	2.17	2.72	5.73	43.82
0.16	12.77	0.16	0.44	0.16	35.40
