# Supplementary material for: Ancient Cytokine Interleukin 15-Like (IL-15L) Induces a Type 2 Immune Response
Source: Front Immunol. 2020 Oct 29;11:549319. doi: 10.3389/fimmu.2020.549319 (PMC7658486; doi:10.3389/fimmu.2020.549319)
Supplement: Supplementary file 7 [file Data_Sheet_7.PDF]

Supplementary file 8

Table with read counts for immune marker genes, as determined by NGS analysis, for rainbow trout six hours after intraperitoneal injection of recombinant cytokines.

Total RNA samples were isolated, 6 h after injection, from spleen from juvenile rainbow trout injected with IL-2, IL-15-RLI, IL-15La-RLI, or buffer control, five fish per treatment. These RNA samples were pooled per treatment and subjected to Next Generation Sequencing (NGS) analysis. The generated reads from each pooled sample were mapped against the open reading frames of the indicated references with a minimum 99.9% mapping quality without gaps by Geneious 11.1.5 (https://www.geneious.com). The table indicates the number of gene-specific reads found as well as, for homogenization, their number per 10^7 reads.

Target genes / NGS library groups	NM_001134847.1 **Perforin**	FJ184374.1 **IFNγ1**	FJ184375.1 **IFNγ2**	AB574337.1 **IL-4/13A**	HG794522.1 **IL-4/13B1**	HG794523.1 **IL-4/13B2**
Libo3547 **IL-15-RLI** Total reads: 22,986,115	591 reads $257.1/10^7$	18 reads $7.8/10^7$	18 reads $7.8/10^7$	5 reads $2.2/10^7$	2 reads $0.9/10^7$	0 reads 0
Libo3548 **IL15-La-RLI** Total reads: 28,205,720	479 reads $169.8/10^7$	1 reads $0.4/10^7$	4 reads $1.4/10^7$	12 reads $4.3/10^7$	20 reads $7.1/10^7$	8 reads $2.8/10^7$
Libo3549 **IL-2** Total reads: 17,609,524	517 reads $293.6/10^7$	2 reads $1.1/10^7$	16 reads $9.1/10^7$	1 reads $0.6/10^7$	1 reads $0.6/10^7$	0 reads 0
Libo3550 **Buffer control** Total reads: 23,248,192	424 reads $182.4/10^7$	0 reads 0	0 reads 0	0 reads 0	0 reads 0	0 reads 0
