# Supplementary material for: Ancient Cytokine Interleukin 15-Like (IL-15L) Induces a Type 2 Immune Response
Source: Front Immunol. 2020 Oct 29;11:549319. doi: 10.3389/fimmu.2020.549319 (PMC7658486; doi:10.3389/fimmu.2020.549319)
Supplement: Supplementary file 8 [file Data_Sheet_8.PDF]

Supplementary file 9 Table of primer sequences

A. Primer sequences used for molecular cloning of trout *IL-15La* and *IL-15Lb*

Primer name	Primer Sequence (5'-3')	Use	
Trout_IL-15La_CDS_F	GCTACTTCTCTACTGATCTAAAGCTC	amplification of full-length trout *IL-15La* ORF	
Trout_IL-15La_CDS_R	TGTCCCTGTCTATGGATGTGTCC		
Trout_IL-15Lb_CDS_F	CTGCAGATGACATGGACAAGAG	amplification of full-length trout *IL-15Lb* ORF	
Trout_IL-15Lb_CDS_R	TCTGCTCTTATGTGAGTCCTTG		
Trout_IL-15La_5'RACE_R	CAGTTGGCACCTTCTTTCTGTTTGATCAAGGACTTCAGC	5'-RACE amplification of trout *IL-15La*	First PCR
Trout_IL-15La_5'RACE.nes_R	GATAATCAGCCAAAGTTGGGGTGTACAG		Nested PCR
Trout_IL-15Lb_5'RACE_R	TCCAGAATCCCTAGTAATGTTGTTAAGAAATCATTTGCTGCCTG	5'-RACE amplification of trout *IL-15Lb*	First PCR
Trout_IL-15Lb_5'RACE.nes_R	GTCGTAAAGCATGTGAGTGTGGACC		Nested PCR

Table of primers sequences *continued*

B. Primer sequences used for gene expression analysis

Primer name	Primer Sequence (5'-3')	Use	Accession number
Trout_IL-15La_F	CATGTGCAGTAAAGAACTTCCCGG	Semi-quantitative PCR for trout *IL-15La*	MK619679
Trout_IL-15La_R	TTTGGCTTGTATATGGGTGAGGACTTA		
Trout_IL-15Lb_F	AAGGAGGAAGTTCACAGGATGGAATC	Semi-quantitative PCR for trout *IL-15Lb*	MK619680
Trout_IL-15Lb_R	AGTAATGTTGTTAAGAAATCATTTGCTGCCTG		
Trout_CD8α_F	ATGAAAATGGTCCAAAAGTGGATGC	Semi-quantitative PCR for trout *CD8α*	NM_001124263
Trout_CD8α-R	GGTTAGAAAAGTCTGTTGTTGGCTATAGG		
Trout_β-actin_F	GCTGTCTTCCCCTCCATCGTC	Semi-quantitative PCR for trout *β-actin*	AF157514
Trout_β-actin_R	GGCAGGGGTGTTGAAGGTCTC		
Trout_EF1A_F	CCACAGGCCATCTGATCTACA	Semi-quantitative PCR for trout *EF1A*	NM_001124339
Trout_EF1A_R	TGAGCTGTTTCACTCCCAGAGTGTAG		
Trout_IL-15La_qPCR_F	TTGAGGAAGTTCACAAGATGGAATCAT	Real-time qPCR for trout *IL-15La*	MK619679
Trout_IL-15La_qPCR_R	GTTTTGGCTTGTATATGGGTGAGGACTT		
Trout_IL-15Lb_qPCR_F	GGAGGAAGTTCACAGGATGGAATCAT	Real-time qPCR for trout *IL-15Lb*	MK619680
Trout_IL-15Lb_qPCR_R	GTCTTTTCTGGTGTAAGGATGAAGAACG		
Trout_IFNγ (1/2)_qPCR_F	AAACATAGACAAACTGAAAGTCCA	Real-time qPCR for trout *IFNγ (1/2)*	FJ184374<br>FJ184375
Trout_IFNγ (1/2)_qPCR_R	CGTCCAGAACCACACTCATCA		
Trout_PFN1_qPCR_F	CTTTGGCACGCATTACATTACCA	Real-time qPCR for trout *Perforin-1*	NM_001134847
Trout_PFN1_qPCR_R	AGCTGGCCTTGCTCTCTGTCTTA		
Trout_IL-4/13A_qPCR_F	CACCACAAAGTGCAAGGAGTT	Real-time qPCR for trout *IL-4/13A*	AB574337
Trout_IL-4/13A_qPCR_R	TGGTCTTGGCTCTTCACAACG		
Trout_IL-4/13B1_qPCR_F	GAGATTCATCTACTGCAGAGGATCATGA	Real-time qPCR for trout *IL-4/13B1*	HG794522
Trout_IL-4/13B1_qPCR_R	GCAGTTGGAAGGGTGAAGCTTATTGTA		
Trout_IL-4/13B2_qPCR_F	GAGACTCATCTATTGCGTATGATCATCG	Real-time qPCR for trout *IL-4/13B2*	HG794523
Trout_IL-4/13B2_qPCR_R	TGCAGTTGGTTGGATGAAACTTATTGTA		
Trout_EF1A_qPCR_F	CAAGGATATCCGTCGTGGCA	Real-time qPCR for trout *EF1A*	NM_001124339
Trout_EF1A_qPCR_R	ACAGCGAAACGACCAAGAGG		
