# Supplementary material for: Ancient Cytokine Interleukin 15-Like (IL-15L) Induces a Type 2 Immune Response
Source: Front Immunol. 2020 Oct 29;11:549319. doi: 10.3389/fimmu.2020.549319 (PMC7658486; doi:10.3389/fimmu.2020.549319)
Supplement: Supplementary file 9 [file Data_Sheet_9.PDF]

Supplementary file 6

Additional information on RT-qPCR experiments

Table of Contents **Page**

6A: RT-qPCR analysis of immune marker gene expression in trout total splenocytes after incubation with purified recombinant trout IL-2, IL-15-RLI, and IL-15La-RLI produced in insect cells (*table with Ct values used for main text Fig. 10*) 3

6B: RT-qPCR analysis of immune marker gene expression in trout total splenocytes after incubation with recombinant trout cytokine containing supernatants from transfected HEK293T cells 4

6C: RT-qPCR analysis of immune marker gene expression in trout splenocyte subpopulations after incubation with purified recombinant trout IL-2, IL-15-RLI and IL-15La-RLI produced in insect cells (*information supporting main text Fig. 11*) 8

6D: RT-qPCR analysis of immune marker gene expression in trout spleen and head kidney after injection 12

with purified recombinant trout IL-2, IL-15-RLI, and IL-15La-RLI produced in insect cells (*table with Ct values used for main text Fig. 12*)

Supplementary file 6A. RT-qPCR analysis of immune marker gene expression in trout total splenocytes after incubation with purified recombinant trout IL-2, IL-15-RLI and IL-15La-RLI produced in insect cells (*table with the Ct values used for main text Fig. 10*).

Total splenocytes of four individual trout (Nos.1-4) were stimulated for 4 h and 12 h at 15 °C with purified recombinant trout cytokines IL-2, IL-15-RLI and IL-15La-RLI (produced in insect cells; see Supplementary file 4) at 0.2, 1 and 5 nM. After incubation, total RNA was isolated and reverse transcribed into cDNA, followed by quantitative PCR (qPCR) for expression analysis of *IFNγ*, *perforin*, *IL-4/13A*, *IL-4/13B1*, *IL-4/13B2* and *EF1A*. The Ct values were determined as the averages of technical duplicates and are shown in a table; Italic font is used for cases in which the technical duplicate values differed >0.5. The Ct values were used for construction of the graphs shown in main text Fig. 10.

Table with the Ct values used for main text Fig. 10

		4 h										12 h									
		Empty	IL-15-RLI			IL-15La-RLI			IL-2			Empty	IL-15-RLI			IL-15La-RLI			IL-2		
			5 nM	1 nM	0.2 nM	5 nM	1 nM	0.2 nM	5 nM	1 nM	0.2 nM		5 nM	1 nM	0.2 nM	5 nM	1 nM	0.2 nM	5 nM	1 nM	0.2 nM
No.1	EF1A	16.83	16.86	16.65	16.61	16.56	16.51	16.45	16.46	16.62	16.57	17.21	16.89	16.94	16.93	16.98	17.13	16.99	16.92	16.97	17.53
	IFNg1/2	28.39	25.66	25.68	25.69	28.11	27.96	27.76	25.67	26.17	26.37	29.96	26.39	26.39	26.85	29.87	29.80	29.84	26.63	27.23	28.40
	PFN1	23.80	22.83	22.98	22.94	23.77	23.77	23.73	23.10	23.46	23.58	25.00	22.57	22.57	22.75	24.97	25.12	25.00	23.77	*24.09*	25.18
	IL-4/13A	*24.81*	24.86	24.65	24.79	23.14	23.02	23.21	*24.98*	24.85	24.89	23.15	22.77	22.72	22.94	21.47	21.52	21.33	22.95	23.02	23.96
	IL-4/13B1	22.11	21.91	21.87	21.92	21.02	20.97	21.04	21.38	21.83	21.91	22.59	22.06	22.14	22.20	20.68	20.92	20.66	21.87	22.08	22.98
	IL-4/13B2	24.01	23.92	23.84	23.79	22.62	22.69	22.81	23.32	23.60	23.52	25.42	24.82	24.95	24.85	23.49	23.60	23.58	24.82	24.86	25.57
No.2	EF1A	16.86	16.66	16.45	16.37	16.42	16.44	16.56	16.64	16.63	16.77	16.46	16.46	16.92	17.18	17.33	16.96	17.00	17.05	16.82	16.64
	IFNg1/2	32.71	29.63	29.22	29.36	32.56	32.50	*32.28*	28.94	29.70	30.07	33.90	29.55	29.80	30.42	*34.00*	34.40	33.50	30.32	30.20	30.79
	PFN1	24.61	24.08	23.78	23.73	24.27	24.39	24.48	24.20	24.33	24.58	26.19	23.96	24.52	24.92	27.14	26.72	26.87	25.86	25.80	26.02
	IL-4/13A	28.72	28.76	28.46	28.45	25.93	25.92	25.91	28.45	28.34	29.02	26.16	25.97	26.12	26.54	23.54	23.40	23.27	25.87	25.73	25.86
	IL-4/13B1	25.56	25.24	24.86	24.87	23.72	23.75	23.82	24.48	24.66	25.08	25.85	25.26	25.60	25.96	23.34	23.28	23.10	24.74	24.79	24.95
	IL-4/13B2	25.67	25.51	25.05	25.10	23.85	23.74	23.68	24.80	24.95	25.20	26.81	26.26	26.46	26.89	24.15	24.07	23.84	25.71	25.67	26.17
No.3	EF1A	16.90	16.76	16.29	16.50	16.63	16.52	16.68	16.32	16.63	16.49	17.34	16.91	16.68	16.66	16.92	17.11	18.53	17.60	16.59	16.89
	IFNg1/2	*31.30*	27.98	27.76	28.19	31.51	31.03	31.16	28.06	28.70	28.90	33.01	27.65	27.71	27.92	32.53	32.11	33.18	29.47	29.23	29.84
	PFN1	25.61	24.74	24.55	25.00	25.76	25.52	25.86	24.96	25.28	25.27	27.34	24.53	24.46	24.66	27.39	27.25	27.90	26.52	26.05	26.45
	IL-4/13A	28.52	28.78	*28.34*	28.86	25.83	25.77	25.87	27.97	28.39	28.27	26.33	25.97	26.04	25.59	22.61	22.52	22.94	25.78	25.25	25.73
	IL-4/13B1	25.47	25.26	24.84	24.91	23.56	23.36	23.62	23.94	24.35	24.57	25.82	25.70	25.50	25.47	22.94	22.97	23.62	25.22	24.61	25.16
	IL-4/13B2	26.25	26.04	25.65	25.78	23.76	23.84	23.97	24.92	25.41	25.45	27.80	27.49	27.31	26.89	23.95	24.02	24.86	26.92	26.30	26.86
No.4	EF1A	17.79	17.92	17.90	17.68	17.68	17.56	17.74	17.48	17.54	17.71	17.97	17.69	17.60	17.84	17.73	17.73	17.67	17.77	17.70	17.61
	IFNg1/2	32.77	29.86	30.33	31.28	32.78	32.25	*32.65*	29.29	29.96	30.70	34.43	30.04	30.59	32.41	*34.62*	34.08	34.00	30.53	31.92	31.69
	PFN1	25.93	24.89	24.89	25.12	25.42	25.48	25.54	24.59	24.93	25.32	27.41	24.69	24.78	25.29	26.80	26.81	*26.89*	25.93	26.41	26.50
	IL-4/13A	26.90	26.89	26.91	26.76	23.98	23.98	24.09	26.06	26.13	26.82	24.60	24.26	24.18	24.49	21.67	21.54	*22.08*	23.56	23.72	24.07
	IL-4/13B1	23.82	23.79	23.55	23.53	22.34	22.36	22.58	22.71	22.85	23.32	23.90	23.86	23.80	23.98	21.55	21.68	21.77	23.14	23.40	23.56
	IL-4/13B2	25.97	25.94	25.65	25.78	24.42	24.32	24.73	24.89	24.98	25.77	26.93	27.22	27.04	27.28	24.32	24.73	24.61	26.45	26.92	27.08

Supplementary file 6B. RT-qPCR analysis of immune marker gene expression in trout total splenocytes after incubation with recombinant trout cytokine containing supernatants from transfected HEK293T cells.

The immune marker gene expression profiles exhibited by trout total splenocytes after incubation with trout cytokine containing supernatants from transfected HEK293T cells (data presented in this file) agree with the results obtained when using purified (fusion-type) trout cytokines produced in insect cells (main text Fig. 10), providing evidence that the observations cannot be explained by cytokine preparation artefacts.

Two independent experiments, using two trout individuals (Tr.1 and Tr.2) and supernatants from independently transfected HEK293T cells were performed. Trout total splenocytes were incubated for 4 h and 12 h at 15 °C with supernatants from HEK293T cells that had been transfected with DNA plasmids encoding trout IL-2, IL-15, IL-15La or IL-15Lb, with (+) or without (-) trout sIL-15Rα. As negative controls, supernatants from HEK293T cells transfected for sIL-15Rα alone (sIL-15Rα) or with an empty control vector (control) were used. After incubation, total RNA was isolated from the splenocytes and reverse transcribed into cDNA, followed by quantitative PCR (qPCR) for expression analysis of *interferon γ (IFNγ), perforin, IL-4/13A, IL-4/13B1, IL-4/13B2* and *EF1A*. Expression levels were equilibrated against *EF1A* expression and the values for the mock-treated controls were set to 1 in each experimental panel. The Ct values obtained in the experiments are shown in (c); the Ct values were determined as the averages of technical duplicates, and Italic font is used for cases in which the technical duplicate values differed >0.5.

Results of both experiments, (a) for trout individual Tr.1 and (b) for trout individual Tr.2, show that: (1) IL-15 enhances the expression of type 1 immune genes *IFNγ* and *perforin*, and doesn't need the co-presence of sIL-15Rα to do so; (2) IL-15La and IL-15Lb enhance the expression of type 2 immune genes *IL-4/13A*, *IL-4/13B1* and *IL-4/13B2*, but only do so in the co-presence of sIL-15Rα; (3) IL-2 clearly enhances the expression of *IFNγ* and may slightly enhance the expression of the other investigated genes, and IL-2 doesn't need the co-presence of sIL-15Rα to do so.

(Supplementary file 6B)

(a) *Relative expression levels of IFNγ, perforin, IL-4/13A, IL-4/13B1 and IL-4/13B2 in total splenocytes of trout individual Tr.1 after incubation for 4 h and 12 h with recombinant trout cytokine containing supernatants from transfected HEK293T cells*

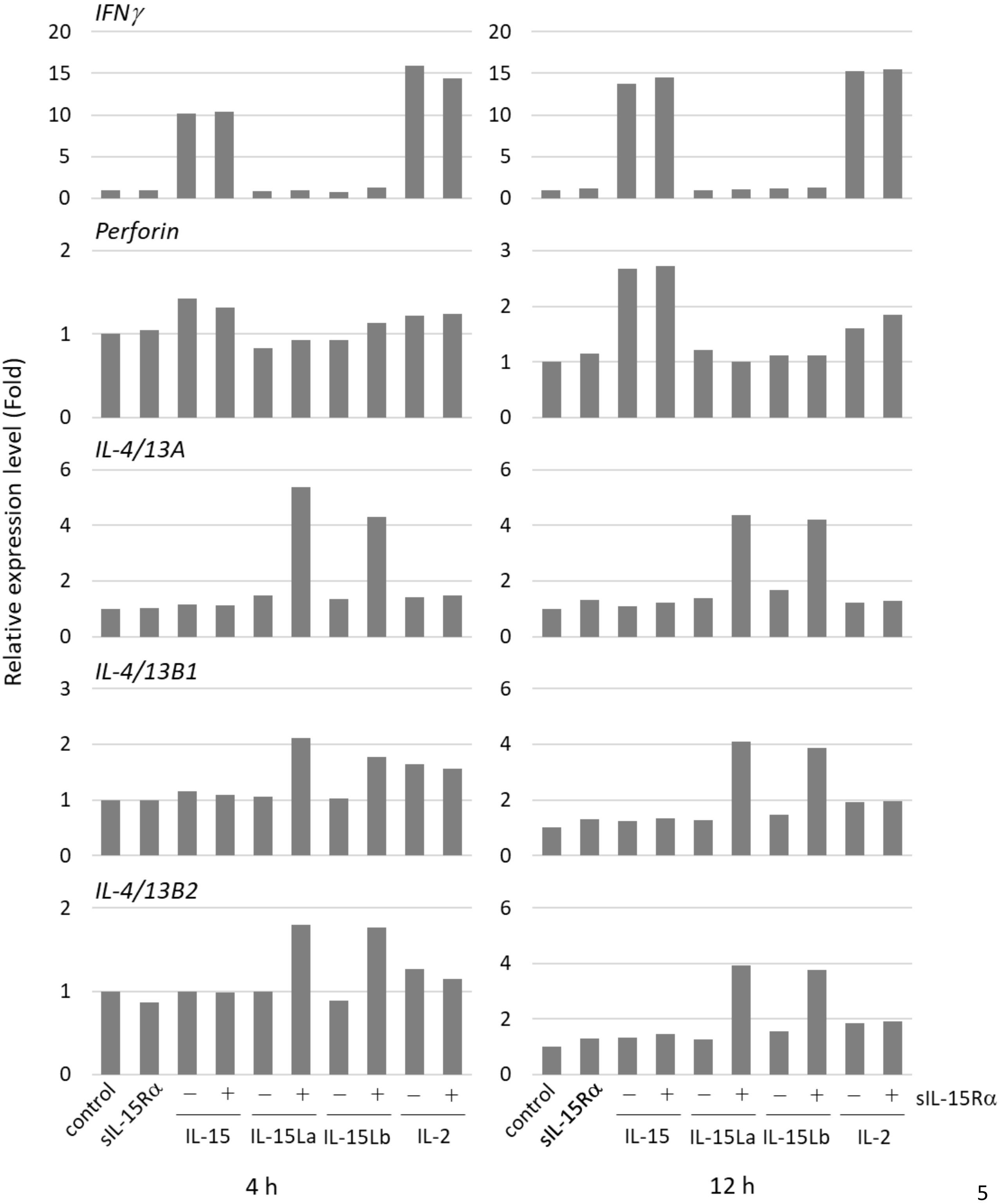

(Supplementary file 6B)

(b) *Relative expression levels of IFNγ, perforin, IL-4/13A, IL-4/13B1 and IL-4/13B2 in total splenocytes of trout individual Tr.2 after incubation for 4 h and 12 h with recombinant trout cytokine containing supernatants from transfected HEK293T cells*

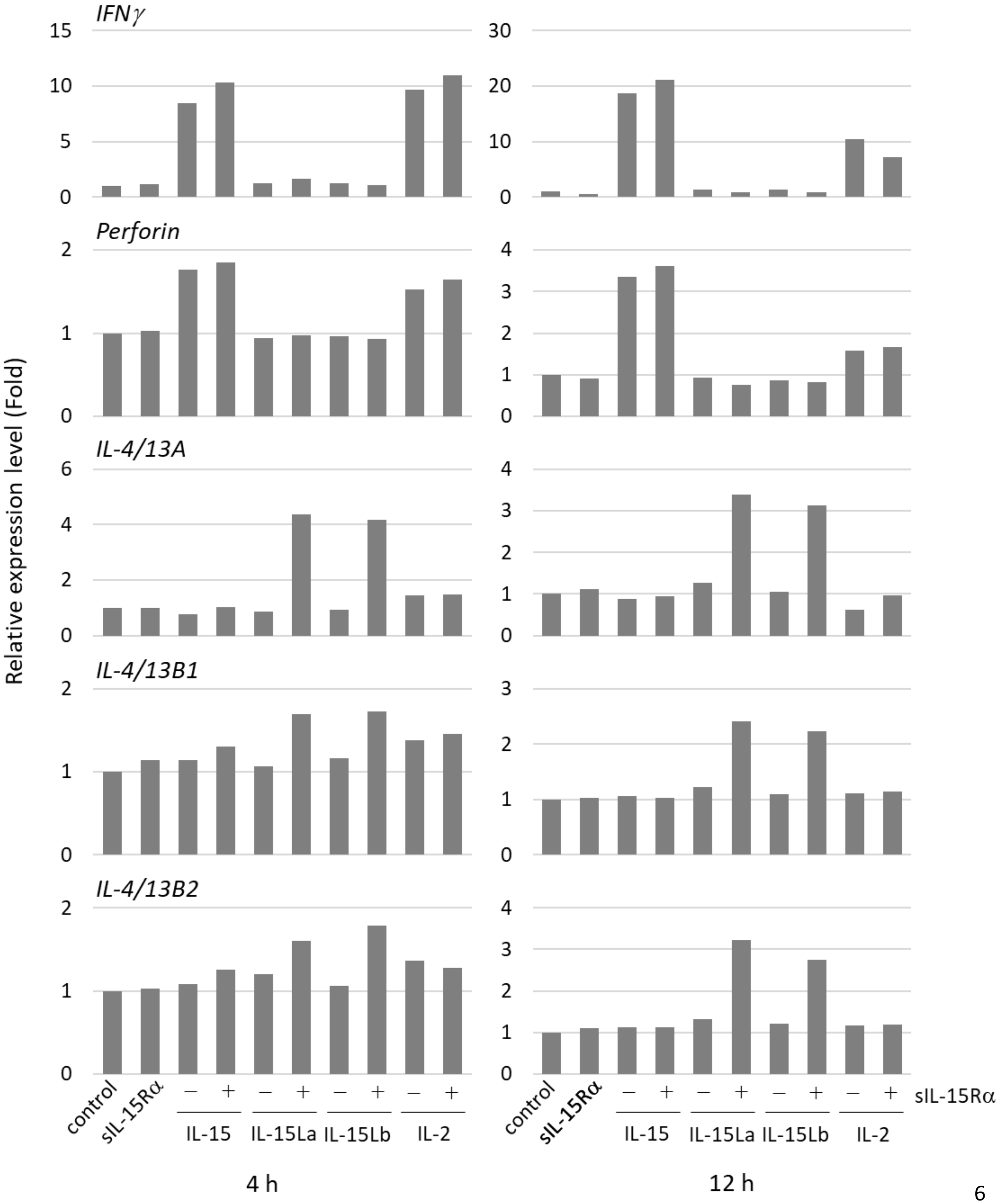

(Supplementary file 6B)

(c) *Table with the Ct values used for the graphs shown in (a) and (b).*
w/o represents without sIL-15Rα, w represents with sIL-15Rα

		4 h										12 h									
		Empty	sIL-15Rα	IL-15		IL-15La		IL-15Lb		IL-2		Empty	sIL-15Rα	IL-15		IL-15La		IL-15Lb		IL-2	
				w/o	w	w/o	w	w/o	w	w/o	w			w/o	w	w/o	w	w/o	w	w/o	w
Tr.1	EF1α	16.34	15.89	15.74	15.74	15.91	16.14	15.54	15.91	16.20	16.13	15.90	16.02	15.61	15.88	15.70	15.77	16.18	15.74	15.77	15.78
	IFNγ1/2	30.90	30.55	26.96	26.92	30.67	30.78	30.41	30.04	26.77	26.85	31.97	31.84	27.89	28.10	31.74	31.75	31.95	31.44	27.90	27.89
	PFN1	24.44	23.93	23.33	23.45	24.28	24.35	23.76	23.83	24.02	23.93	24.79	24.71	23.07	23.32	24.31	24.66	24.90	24.46	23.97	23.78
	IL-4/13A	27.41	26.92	26.62	26.63	26.43	24.79	26.19	24.88	26.76	26.65	23.92	23.65	23.50	23.63	23.25	21.66	23.44	21.69	23.49	23.44
	IL-4/13B1	22.69	22.24	21.88	21.96	22.18	21.41	21.85	21.43	21.83	21.83	22.06	21.79	21.45	21.61	21.50	19.89	21.77	19.94	20.99	20.95
	IL-4/13B2	23.35	23.10	22.76	22.76	22.93	22.31	22.73	22.10	22.87	22.95	23.96	23.71	23.25	23.40	23.43	21.86	23.60	21.88	22.94	22.91
Tr.2	EF1α	17.22	17.29	16.80	17.08	16.63	16.94	16.72	16.86	17.00	16.99	16.91	16.91	16.69	16.89	17.32	16.70	16.75	16.60	16.65	16.79
	IFNγ1/2	*32.76*	32.63	29.26	29.24	31.90	31.77	31.94	32.26	29.26	29.06	33.47	34.48	29.02	29.05	*33.42*	33.37	32.87	*33.30*	29.82	30.51
	PFN1	22.78	22.81	21.54	21.75	22.29	22.55	22.34	22.53	21.95	21.83	23.58	23.72	21.62	21.71	24.10	23.77	23.63	23.56	22.68	22.73
	IL-4/13A	26.17	26.27	26.14	25.97	25.80	23.77	25.76	23.75	25.43	25.37	22.92	22.76	22.88	23.00	23.00	20.95	22.68	20.97	*23.37*	22.85
	IL-4/13B1	22.82	22.69	22.21	22.29	22.14	21.78	22.10	21.67	22.13	22.04	22.26	22.22	21.95	22.20	22.38	20.79	21.98	20.79	21.86	21.95
	IL-4/13B2	26.47	26.51	25.94	26.00	25.62	25.51	25.89	25.28	25.80	25.88	27.12	26.99	26.73	26.94	27.14	25.23	26.68	25.36	26.63	26.76

Supplementary file 6C. RT-qPCR analysis of immune marker gene expression in trout splenocyte subpopulations after incubation with purified recombinant trout IL-2, IL-15-RLI and IL-15La-RLI produced in insect cells (*information supporting main text Fig. 11*).

The table in (a) shows the Ct values used for making main text Fig. 11. In comparison to main text Fig. 11, the graphs in (b) and (c) provide additional information on the gene expressions in $CD4^+$, $CD8^+$ and IgM^+ cells. The graphs in (b) are identical to those in main text Fig. 11, except for being shown at a larger scale, while the graphs (c) are based on the same dataset but using a different approach of observations with high Ct values.

$CD4^+$, $CD8^+$, IgM^+ and $CD4^-CD8^-IgM^-$ (triple negative; TN) lymphocyte populations were isolated from trout spleen using flow sorting (for an example see Supplementary file 3) and stimulated for 12 h at 15 °C with purified recombinant trout cytokines IL-2, IL-15-RLI and IL-15La-RLI (produced in insect cells; see Supplementary file 4) at 0.2 and 5 nM. After incubation, total RNA was isolated and reverse transcribed into cDNA, followed by quantitative PCR (qPCR) for expression analysis of *IFNγ*, *perforin*, *IL-4/13A*, *IL-4/13B1*, *IL-4/13B2* and *EF1A*. The determined Ct values are shown in a table (a), with Ct values ≥35 highlighted by salmon color because the reliability of those values may be considered questionable; the Ct values were determined as the averages of technical duplicates, and Italic font is used for cases in which the technical duplicate values differed >0.5.

Using the data shown in the table (a), expression levels were equilibrated against *EF1A* expression and the values for the mock-treated TN control ("0") were set to 1 in each experimental panel. In the graphs [(b) and (c), and main text Fig. 11], the average values of four experiments are shown together with error bars representing SD. The differences between the graphs shown in (b) and (c) are based on different approaches of the Ct ≥35 values. For the calculations resulting in the (b) and main text Fig. 11 graphs, the Ct values were used as indicated in table (a), with "No Ct" interpreted as zero expression. For the calculations resulting in the (c) graphs also the Ct values indicated in table (a) were used, but now all Ct values ≥35 (and "No Ct" observations) were equalized as 35.

We are showing figures (b) and (c) in order to provide complete information. However, overall, the reliability and functional relevance of the quantitative comparisons between cases of very low expression levels can be questioned, and many of the data shown in (b) and (c) should not be overinterpreted (which is also why we refrained from including statistical analysis in these two figures). The important finding of our RT-qPCR analyses of trout total and subpopulation splenocytes (Supplementary files 6A, 6B and 6C, and main text Figs. 10 and 11) is that in splenocytes from healthy trout most *IFNγ*, *IL-4/13A*, *IL-4/13B1* and *IL-4/13B2* is expressed by $CD4^-CD8^-IgM^-$ lymphocytes, and that expression levels of these cytokine gene transcripts in splenocytes are differentially affected by IL-15(+sIL-15Rα) versus IL-15L+sIL-15Rα.

(Supplementary file 6C)

(a) *Table with the Ct values used for main text Fig. 11*

CD8a

			IL-15-RLI		IL-15La-RLI		IL-2	
		Empty	0.2 nM	5 nM	0.2 nM	5 nM	0.2 nM	5 nM
#1	EF1a	23.84	22.88	23.43	23.7	23.33	23.39	23.33
	IFNg1/2	37.88	34.49	32.95	37.64	34.51	33.34	33.76
	PFN1	28.66	27.55	28.02	28.74	28.39	28.04	27.93
	IL-4/13A	34.6	34.71	34.71	34.83	35.79	34.56	34.64
	IL-4/13B1	37.35	38.62	39.12	35.09	No Ct	37.42	30.24
	IL-4/13B2	No Ct	No Ct	No Ct	No Ct	No Ct	No Ct	33.92
#2	EF1a	22.57	22.72	22.21	23.17	22.98	22.92	22.89
	IFNg1/2	37	34.12	34.28	35.43	34.49	33.42	33.7
	PFN1	28.62	28.41	28.01	29.03	28.94	28.72	28.6
	IL-4/13A	32.88	32.76	31.77	32.93	32.7	33.25	32.93
	IL-4/13B1	36.21	31.6	No Ct	39.31	37.33	33.65	No Ct
	IL-4/13B2	No Ct	No Ct	No Ct	No Ct	No Ct	No Ct	No Ct
#3	EF1a	22.82	23.97	23.74	23.39	23.83	23.72	22.96
	IFNg1/2	34.49	35.35	34.64	34.61	No Ct	35.67	33.08
	PFN1	30.09	30.43	30.16	30.43	30.71	30.37	29.6
	IL-4/13A	35.03	35.88	34.89	34.68	36.35	39.48	35.09
	IL-4/13B1	38.07	No Ct	34.33	31.31	37.23	35.81	No Ct
	IL-4/13B2	No Ct	No Ct	No Ct	35.21	No Ct	No Ct	No Ct
#4	EF1a	23.92	23.83	24.42	23.93	24.19	24.49	23.98
	IFNg1/2	No Ct	33.69	35.42	35.27	35.08	35.37	32.88
	PFN1	29.85	29.78	30.22	29.74	30.23	30.17	29.89
	IL-4/13A	33.88	33.25	33.93	33.3	33.98	35.41	34.2
	IL-4/13B1	37.98	32.48	38.04	33.58	38.32	39.22	39.16
	IL-4/13B2	No Ct	No Ct	No Ct	39.22	No Ct	No Ct	No Ct

CD4

			IL-15-RLI		IL-15La-RLI		IL-2	
		Empty	0.2 nM	5 nM	0.2 nM	5 nM	0.2 nM	5 nM
#1	EF1a	20.37	20.94	20.51	21.05	20.76	20.92	20.83
	IFNg1/2	35.46	33.64	34.51	36.9	34.53	34.41	32.71
	PFN1	28.18	28.73	28.31	28.79	28.6	28.85	28.74
	IL-4/13A	32.13	32.92	32.22	32.71	32.36	32.92	32.74
	IL-4/13B1	28.32	29.61	28.47	28.63	30.6	28.69	27.93
	IL-4/13B2	33.75	34.36	No Ct	34.35	32.78	32.54	31.94
#2	EF1a	20.32	20.21	20.47	20.38	20.47	20.85	20.46
	IFNg1/2	34.6	34.21	32.98	34.31	34.95	36.2	33.75
	PFN1	29.41	28.86	29.48	29.47	29.39	29.54	29.46
	IL-4/13A	29.55	29.71	30.29	29.72	29.81	30.01	30.02
	IL-4/13B1	29.86	28.73	30.92	29.31	28.52	30.53	31.16
	IL-4/13B2	34.22	33.65	33.74	32.85	31.86	34.44	32.48
#3	EF1a	21.99	21.53	21.24	21.51	21.76	21.48	21.54
	IFNg1/2	35.01	34.67	39.47	34.32	34.34	33.69	34.12
	PFN1	31.58	31.09	30.79	30.92	31.06	31.44	31.18
	IL-4/13A	33.92	33.18	33.02	33.56	33.63	34.37	33.92
	IL-4/13B1	No Ct	31.37	33.85	33.42	34.24	29.8	31.17
	IL-4/13B2	35.08	35.44	33.83	No Ct	35.18	34.17	34.67
#4	EF1a	21.71	22.16	22.01	22.62	21.92	22.62	21.66
	IFNg1/2	38.75	35.82	35.74	No Ct	33.49	34.31	35.4
	PFN1	30.95	31.69	31.14	31.51	31.39	31.43	30.87
	IL-4/13A	31.97	31.94	31.95	32.11	31.81	31.73	31.37
	IL-4/13B1	33	34.03	31	30.37	31.75	39.45	29.74
	IL-4/13B2	36.01	36.93	34.13	35.89	37.95	No Ct	36.95

IgM

			IL-15-RLI		IL-15La-RLI		IL-2	
		Empty	0.2 nM	5 nM	0.2 nM	5 nM	0.2 nM	5 nM
#1	EF1a	21.38	21.82	21.46	21.47	21.78	22.27	21.71
	IFNg1/2	36.7	No Ct	No Ct	38.03	38.35	39.69	No Ct
	PFN1	34.17	33.9	33.19	38.19	No Ct	35.96	34.33
	IL-4/13A	35.01	35.5	34.67	34.59	34.77	36.66	35.01
	IL-4/13B1	32.43	No Ct	No Ct	No Ct	30.63	37.65	39.6
	IL-4/13B2	No Ct	No Ct	No Ct	No Ct	34.66	No Ct	No Ct
#2	EF1a	21.85	20.5	21.61	21.6	21.52	21.91	20.99
	IFNg1/2	No Ct	No Ct	No Ct	No Ct	37.78	No Ct	No Ct
	PFN1	37.94	34	33.65	34.51	34.92	35.56	34.21
	IL-4/13A	32.99	31.97	32.1	33.53	32.05	32.09	32.67
	IL-4/13B1	30.01	No Ct	36.61	No Ct	No Ct	No Ct	No Ct
	IL-4/13B2	No Ct	38.93	No Ct	No Ct	No Ct	No Ct	No Ct
#3	EF1a	19.35	20.49	19.63	19.91	19.92	21.42	21.05
	IFNg1/2	38.55	36.8	38.39	36.37	35.85	37.47	38.33
	PFN1	33.76	33.09	33.91	33.76	33.43	33.88	33.4
	IL-4/13A	34.94	34.81	34.63	36.11	35.23	35.36	34.18
	IL-4/13B1	31.6	39.87	No Ct	34.3	No Ct	33.32	No Ct
	IL-4/13B2	No Ct	No Ct	No Ct	No Ct	No Ct	No Ct	No Ct
#4	EF1a	21.62	20.73	22.06	21.92	21.53	21.52	21.47
	IFNg1/2	37.5	No Ct	No Ct	No Ct	38.93	No Ct	No Ct
	PFN1	33.75	32.34	32.95	36.88	33.79	36.11	34.83
	IL-4/13A	32.62	32.91	32.83	33.46	33.51	32.48	33.16
	IL-4/13B1	33.55	39.17	No Ct	37.95	35.92	29.13	39.86
	IL-4/13B2	No Ct	No Ct	No Ct	No Ct	No Ct	No Ct	No Ct

TN

			IL-15-RLI		IL-15La-RLI		IL-2	
		Empty	0.2 nM	5 nM	0.2 nM	5 nM	0.2 nM	5 nM
#1	EF1a	21.47	22.06	22.01	21.78	21.89	23.01	21.86
	IFNg1/2	30.61	29.21	28.69	30.96	30.81	31.6	29.77
	PFN1	27.45	26.7	26.77	27.74	28.17	28.72	27.16
	IL-4/13A	28.08	28.19	27.96	25.6	26.26	29.33	28.04
	IL-4/13B1	20.2	20.35	20.33	18.96	19.44	21.26	19.85
	IL-4/13B2	24.63	25.12	24.95	23.59	23.45	25.27	24.64
#2	EF1a	21.36	20.91	21.08	21.85	21.38	22.26	21.72
	IFNg1/2	31.1	28.86	29.35	31.12	31.44	30.69	29.76
	PFN1	28.83	27.29	27.42	28.58	28.69	28.78	27.94
	IL-4/13A	27.26	26.14	26.59	24.17	24.1	27.11	26.4
	IL-4/13B1	22.02	21.46	21.75	20.87	20.96	21.95	21.25
	IL-4/13B2	25.45	24.74	25.08	24.7	24.51	25.91	24.97
#3	EF1a	21.08	20.81	20.89	21.04	20.92	21.4	21.75
	IFNg1/2	30.36	28.41	28.67	31.04	31.4	31.12	30.62
	PFN1	29.72	28.24	27.83	29.98	29.81	29.76	29.51
	IL-4/13A	31.46	30.32	30.27	27.36	27.1	31.95	32.03
	IL-4/13B1	23.32	22.6	22.5	21.6	21.41	23.14	22.54
	IL-4/13B2	27.03	26.4	26.39	25.65	25.56	27.35	27.29
#4	EF1a	22.74	21.86	21.84	22.43	22.17	22.32	22.14
	IFNg1/2	32.92	31.31	31.05	32.27	32.7	31.63	31.16
	PFN1	31.53	28.85	28.51	30.7	30.87	30.31	30.01
	IL-4/13A	33.27	30.82	30.79	27.74	27.44	32.04	31.83
	IL-4/13B1	23.92	23.02	22.81	22.2	22	23.46	22.88
	IL-4/13B2	29.56	28.89	28.88	28.1	28.29	29.57	28.85

(Supplementary file 6C)

(b) *Relative expression levels of IFNγ, perforin, IL-4/13A, IL-4/13B1 and IL-4/13B2 in CD4⁺, CD8⁺ and IgM⁺ trout spleen morphological lymphocytes after incubation for 12 h with purified recombinant trout cytokines (produced in insect cells) IL-2, IL-15-RLI and IL-15La-RLI at 0.2 and 5 nM. Analysis as done for main text Fig. 11 but shown at a larger scale.*

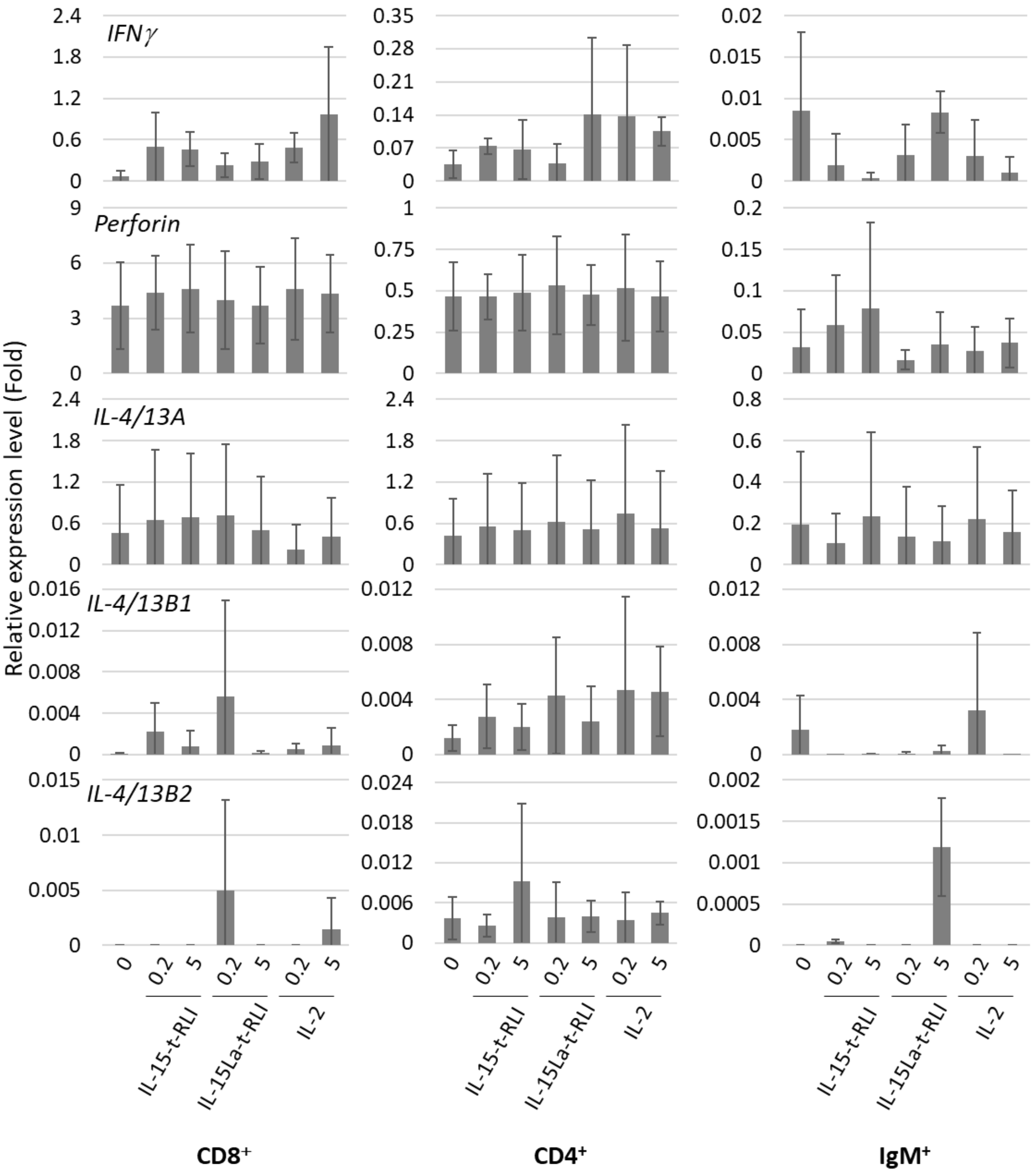

(Supplementary file 6C)

(c) *Relative expression levels of IFNγ, perforin, IL-4/13A, IL-4/13B1 and IL-4/13B2 in CD4*$^{+}$*, CD8*$^{+}$ *and IgM*$^{+}$ *trout spleen morphological lymphocytes after incubation for 12 h with purified recombinant trout cytokines (produced in insect cells) IL-2, IL-15-RLI and IL-15La-RLI at 0.2 and 5 nM. Analysis as done for main text Fig. 11 except for considering all Ct* $\geq$*35 values as 35.*

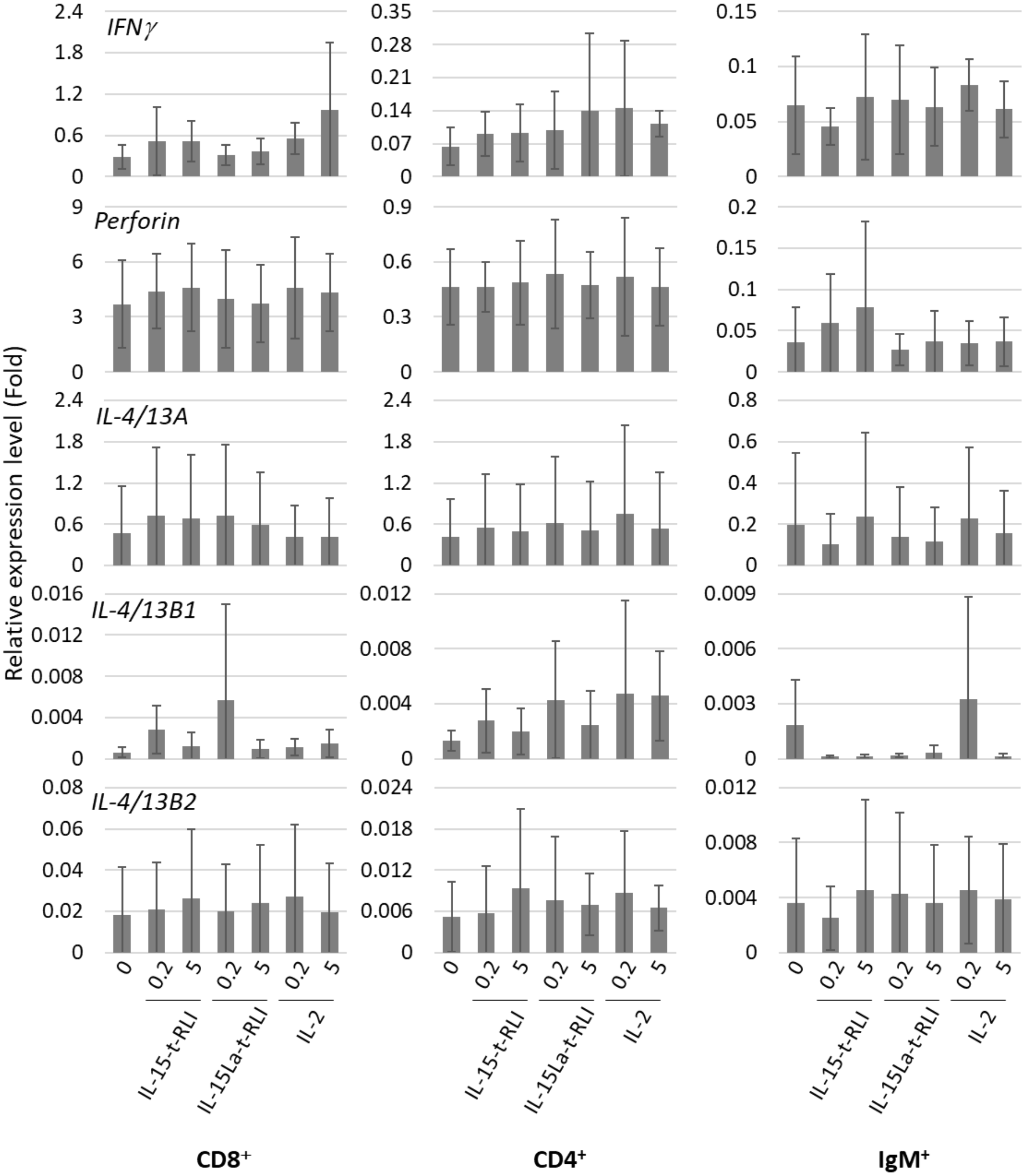

Supplementary file 6D. RT-qPCR analysis of immune marker gene expression in trout spleen and head kidney after injection with purified recombinant trout IL-2, IL-15-RLI, and IL-15La-RLI produced in insect cells (*table with Ct values used for main text Fig. 12*).

Forty rainbow trout juveniles of ~10 grams, ten per cytokine, were injected intraperitoneally with 50 µl recombinant cytokine (1 µM) in protein storage buffer or with buffer control; the cytokines were purified IL-2, IL-15-RLI or IL-15La-RLI that had been produced in insect cells. At 6 h and 12 h after protein injection the spleen and head kidney were harvested from five fish per treatment. Total RNA was isolated and reverse transcribed into cDNA, followed by quantitative PCR (qPCR) for expression analysis of *IFNγ*, *perforin*, *IL-4/13A*, *IL-4/13B1*, *IL-4/13B2* and *EF1A*. The Ct values were determined as the averages of technical duplicates and are shown in the table; Italic font is used for cases in which the technical duplicate values differed >0.5. Data in the same row were obtained from the same trout individual. The values were used for construction of the graphs shown in main text Fig. 12.

Table with the Ct values used for main text Fig. 12

	Spleen 6 h						Head Kidney 6 h					
	EF1-A	IFNg	Perforin	IL4-13A	IL4-13B1	IL4-13B2	EF1-A	IFNg	Perforin	IL4-13A	IL4-13B1	IL4-13B2
IL-15-RLI	16.22	26.97	25.40	*29.14*	30.66	*32.17*	18.31	31.45	28.01	30.18	*34.04*	33.56
	16.40	26.89	25.40	29.20	30.48	32.46	17.85	31.32	27.78	29.84	33.79	*35.68*
	16.16	26.41	25.13	29.12	30.36	32.31	17.48	*30.88*	27.27	30.49	32.60	*33.59*
	16.28	27.15	24.68	28.93	29.72	31.34	17.97	32.61	27.86	30.44	33.30	34.94
	15.62	25.47	25.25	28.55	29.69	31.73	17.73	31.82	28.22	*29.96*	33.45	34.44
IL-15La-RLI	16.47	*29.41*	26.77	27.68	28.56	30.06	18.56	33.43	28.19	28.55	29.48	31.12
	16.05	30.18	25.72	27.36	27.96	29.39	18.10	*33.73*	28.37	29.02	30.11	31.07
	16.84	29.55	26.24	27.17	28.29	29.36	17.91	*34.31*	27.58	29.13	30.11	30.86
	16.08	29.71	25.32	26.82	27.11	28.79	18.17	34.46	27.93	28.71	29.87	30.86
	15.90	29.34	25.20	28.40	28.75	29.94	17.79	34.74	27.69	29.51	*30.71*	32.71
IL-2	15.76	27.68	24.72	*29.64*	*30.87*	31.77	18.17	31.68	28.05	30.17	32.46	*33.87*
	16.05	28.50	*24.74*	29.35	30.70	*32.22*	18.13	*32.75*	27.58	30.07	32.29	*33.38*
	15.62	28.75	25.13	29.07	30.04	*31.90*	17.06	*33.27*	27.78	29.43	31.96	*32.56*
	16.01	*29.78*	25.68	30.20	31.03	*32.70*	18.36	33.31	28.01	30.24	33.56	34.53
	15.41	27.92	24.95	28.73	29.75	31.62	*18.58*	33.37	28.40	30.19	32.29	33.12
Buffer control	16.13	29.75	25.21	29.05	30.67	*31.95*	17.76	34.89	28.40	30.24	33.07	33.44
	16.49	29.90	25.45	28.96	*30.49*	*32.22*	18.45	32.10	27.73	*30.20*	*32.82*	34.72
	17.28	*31.59*	25.79	*30.47*	*32.58*	33.43	18.28	34.82	28.27	*30.52*	32.79	33.90
	15.72	*30.63*	25.49	28.91	30.58	32.25	18.10	*34.85*	28.25	30.04	31.71	*33.75*
	16.20	29.84	25.48	28.80	29.58	*31.85*	17.31	*33.87*	27.54	29.77	*33.12*	*33.96*

	Spleen 12 h						Head Kidney 12 h					
	EF1-A	IFNg	Perforin	IL4-13A	IL4-13B1	IL4-13B2	EF1-A	IFNg	Perforin	IL4-13A	IL4-13B1	IL4-13B2
IL-15-RLI	18.27	*30.21*	27.55	31.06	32.66	34.30	18.14	31.36	27.68	30.00	32.63	*33.34*
	18.15	*28.62*	27.23	*30.21*	32.54	32.41	18.21	30.01	27.62	29.89	32.85	32.78
	18.71	31.58	27.52	31.06	*32.87*	*34.80*	18.19	*31.98*	28.15	29.96	*32.80*	*34.25*
	18.54	29.42	28.11	30.82	*33.06*	*34.52*	17.25	*30.74*	27.92	29.03	*32.60*	*34.01*
	18.03	28.59	27.17	*30.43*	32.17	33.32	18.57	31.04	28.08	29.76	*33.39*	34.84
IL-15La-RLI	17.93	31.94	27.77	28.19	29.56	30.15	16.75	31.42	27.77	27.37	28.93	*30.78*
	17.33	31.09	26.83	27.44	28.96	30.24	16.40	*31.84*	26.71	*27.76*	29.81	30.93
	17.72	*31.25*	26.68	29.13	*31.18*	31.67	16.54	32.71	26.42	*28.10*	*30.86*	*31.91*
	17.64	30.61	26.74	25.65	26.81	28.06	16.56	*31.50*	26.38	26.66	27.54	28.99
	18.37	31.18	27.24	29.34	31.20	*31.96*	17.31	33.69	27.44	29.58	33.29	*32.90*
IL-2	18.42	30.17	27.82	30.33	32.39	*33.95*	16.86	29.77	26.38	28.24	*31.22*	*32.43*
	18.12	*27.56*	27.38	29.57	*30.05*	*31.84*	16.99	28.41	26.47	28.74	29.43	31.11
	17.71	28.07	27.00	29.98	31.19	33.51	17.19	29.88	27.12	28.58	30.68	32.07
	19.58	31.82	27.65	31.25	*33.71*	34.30	18.43	31.77	*28.07*	30.05	*32.31*	*33.38*
	17.76	29.85	27.43	*29.85*	31.88	33.01	18.35	*32.64*	28.72	*30.14*	32.46	*33.11*
Buffer control	18.02	*31.10*	27.72	30.29	*32.13*	*33.12*	18.53	*33.47*	28.75	29.47	*33.54*	34.34
	17.92	31.12	27.08	*29.66*	32.33	*33.74*	18.57	*32.70*	28.45	29.74	*33.36*	*34.62*
	17.80	*31.54*	27.21	29.86	32.08	32.32	18.46	33.02	28.27	29.62	32.55	*32.89*
	18.06	*31.72*	27.31	30.05	*32.06*	*34.03*	17.47	33.05	27.40	29.30	32.44	33.35
	18.40	31.33	27.14	*30.74*	32.03	32.83	18.93	*33.40*	28.59	*30.63*	*34.00*	34.83
